# Rh(iii)-catalyzed regioselective intermolecular *N*-methylene Csp^3^–H bond carbenoid insertion†
†Electronic supplementary information (ESI) available. See DOI: 10.1039/c7sc03802j


**DOI:** 10.1039/c7sc03802j

**Published:** 2017-11-27

**Authors:** Haisheng Xie, Zongren Ye, Zhuofeng Ke, Jianyong Lan, Huanfeng Jiang, Wei Zeng

**Affiliations:** a School of Chemistry and Chemical Engineering , South China University of Technology , No. 381 Wushan Road , Guangzhou , 510641 , P. R. China . Email: jianghf@scut.edu.cn ; Email: zengwei@scut.edu.cn; b School of Materials Science & Engineering , PCFM Lab , Sun Yat-sen University , Guangzhou , 510275 , P. R. China . Email: kezhf3@mail.sysu.edu.cn

## Abstract

A Rh(iii)-catalyzed regioselective intermolecular carbenoid insertion into the *N*-methylene Csp^3^–H bond of acyclic aliphatic amides has been achieved, taking advantage of bidentate–chelation assistance. This methodology has been successfully applied to a broad range of linear and branched-chain *N*-alkylamides, thus providing a practical method for the assembly of diverse beta-amino esters. Mechanism studies and density functional theory (DFT) calculations revealed that a singlet Fischer type carbene insertion *via* an outer-sphere pathway was involved in this *N*-methylene Csp^3^–H bond carbenoid insertion.

## Abstract 2

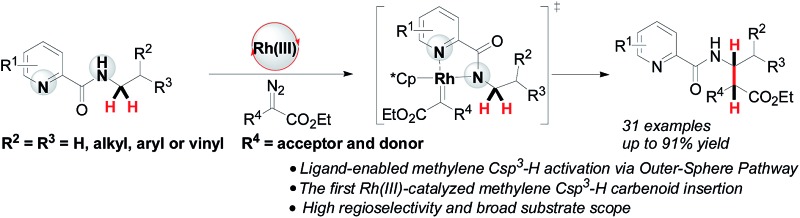
A Rh(iii)-catalyzed regioselective intermolecular carbenoid insertion into the *N*-methylene Csp^3^–H bond has been achieved *via* an outer-sphere pathway.

## Introduction

**Scheme 1:**
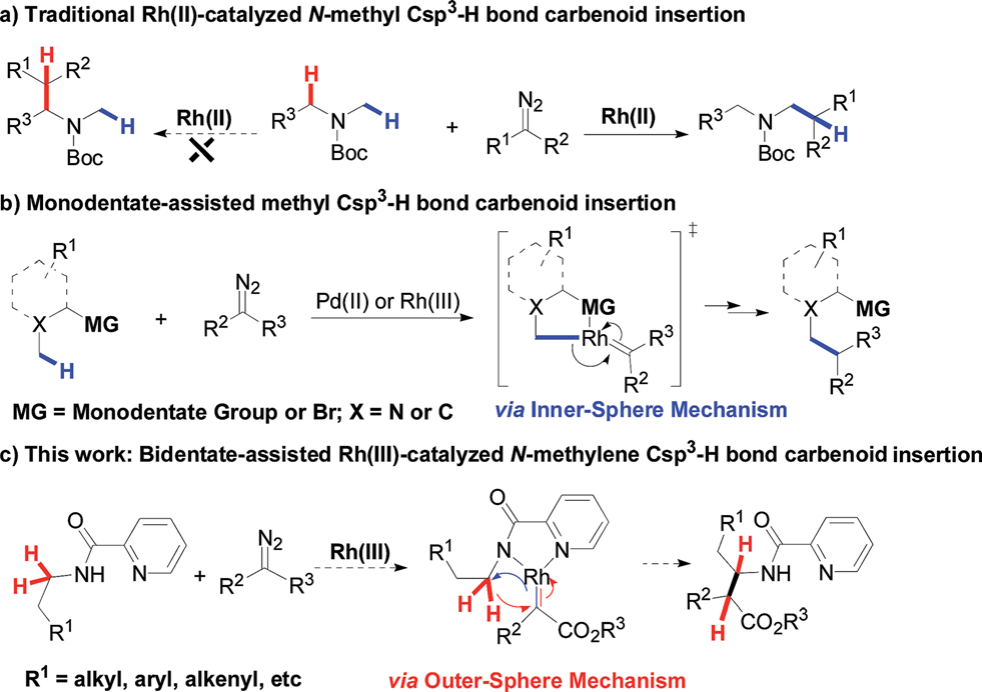
Approaches to versatile Csp^3^–H carbenoid insertion.

Alkyl C–H bond carbenoid functionalization is one of the most challenging topics for atom-economical C–C bond formations.^1^ Over the past few decades, transition-metal catalyzed intra- and intermolecular heteroatom-adjacent Csp^3^–H carbenoid insertions have been well-established for assembling structurally complex molecules, but substrate-specific problems have not yet been overcome.^2^ In terms of the intermolecular version of N-adjacent Csp^3^–H bond carbenoid insertion to acyclic amines, the existing transition metal catalytic systems only tolerate *N*-methyl Csp^3^–H bonds instead of *N*-methylene Csp^3^–H bonds (Scheme 1a).2*a* The intermolecular carbenoid insertion into the *N*-methylene Csp^3^–H bonds of acyclic aliphatic amines is very difficult to achieve because of the delicate balance between steric and electronic factors.^3^

Recently, chelation-assisted intermolecular Csp^2^–H bond carbenoid functionalization has obtained a breakthrough *via* the “Inner-Sphere Pathway^4^ (ISP)”; this strategy provides a powerful approach for site-selective aryl C–H carbenoid insertion. In this regard, Yu,^5^ Glorious,^6^ Rovis,^7^ Li^8^ and others^9^ successively reported that Rh(iii) and Co(iii)-catalyzed ortho aryl Csp^2^–H cross-coupling reactions with diazo compounds could conveniently install C–C bonds into arenes by employing oximes, hydroxamic acids, pyridines or quaternary ammoniums as directing groups. In sharp contrast, ligand-directed alkyl Csp^3^–H carbenoid functionalization remains almost undeveloped, owing to such bonds possessing a relatively smaller s-orbital contribution and larger bond dissociation energy.^10^ To date, only Martin and Zhou have ever reported that transition metal-catalyzed intermolecular Csp^3^–H bond alkylation with diazo compounds could occur through the ISP using aryl bromides, arylamine *N*-oxides and quinoline as reaction platforms, but these tactics were limited to only primary methyl Csp^3^–H bonds (Scheme 1b).^11^ Therefore, developing various types of methylene Csp^3^–H carbenoid insertion remains challenging yet highly desirable. Very recently, we have accomplished a novel Ir(iii)-catalyzed bidentate-assisted regioselective methylene Csp^3^–H nitrene insertion,^12^ in which the “Outer-Sphere Pathway (OSP)”^4^,^12^ is involved in the transformation. Undoubtedly, the chelation-assisted OSP has already brought us a rising innovative concept, thus providing a promising approach to achieve versatile methylene Csp^3^–H functionalizations. Herein, we report an unprecedented Rh(iii)-catalyzed bidentate-assisted regioselective intermolecular carbenoid insertion of *N*-methylene Csp^3^–H bonds *via* the OSP (Scheme 1c). This protocol constitutes a unique tool to rapidly build up complex linear beta-amino acid derivatives, which are among the most important precursors of beta-peptides and beta-lactams^13^ and feature in a large number of naturally occurring and unnatural compounds.^14^

## Results and discussion

We initiated our study by investigating the cross-coupling reaction of *N*-butyl-pyridine-2-carboxylic acid amide (**1a**) and α-diazo-β-ketoester (**2a**) in the presence of metal catalysts including [Cp*IrCl_2_]_2_, Cp*Co(CO)I_2_, Cp*Co(MeCN)_3_SbF_6_, RhCl_3_, Rh_2_(OAc)_4_ and [Cp*RhCl_2_]_2_ (5 mol%) and Ag_2_CO_3_ (20 mol%) in CH_3_CN at 100 °C for 24 h (see Table S-1 in ESI†). To our delight, screening of the catalysts quickly revealed that [Cp*RhCl_2_]_2_ could provide the coupling product **3a** with a promising 47% yield (Table 1, entry 2), in which the C–H carbenoid insertion occurred highly regioselectively at the *N*-methylene C–H bond. Unfortunately, other transition metal salts such as [Cp*IrCl_2_]_2_, Cp*Co(CO)I_2_, Rh_2_(OAc)_4_, *etc.* were not efficient at all. Then, various types of silver additive were evaluated (entries 3–7) and it was found that employing AgOAc as an additive could moderately improve the yield of **3a** from 47% to 65% (compare entry 2 with 7, also see Table S-2 in ESI†). The reaction conversion could be further promoted when the transformation was conducted in TFE, which delivered an 89% yield of **3a** (entry 8); however, switching to other solvents such as 1,4-dioxane or DMSO led to a significantly lower coupling efficiency (see Table S-3 in ESI†). Also, lowering or increasing the reaction temperature resulted in worse results (entries 9 and 10).

**Table 1:** Optimization of reaction conditions^*a*^

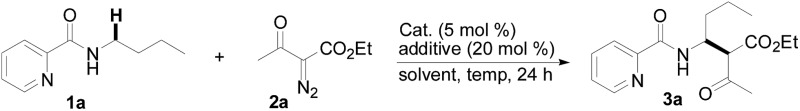				
Entry	Catalyst	Additive	Solvent	Yield^*b*^ (%)
1	Rh_2_(OAc)_4_	Ag_2_CO_3_	CH_3_CN	0
2	[Cp*RhCl_2_]_2_	Ag_2_CO_3_	CH_3_CN	47
3	[Cp*RhCl_2_]_2_	AgClO_4_	CH_3_CN	—
4	[Cp*RhCl_2_]_2_	AgSbF_6_	CH_3_CN	28
5	[Cp*RhCl_2_]_2_	AgBF_4_	CH_3_CN	15
6	[Cp*RhCl_2_]_2_	AgNTf_2_	CH_3_CN	63
7	[Cp*RhCl_2_]_2_	AgOAc	CH_3_CN	65
8	[Cp*RhCl_2_]_2_	AgOAc	TFE^*c*^	89
9	[Cp*RhCl_2_]_2_	AgOAc	TFE	69^*d*^
10	[Cp*RhCl_2_]_2_	AgOAc	TFE	71^*e*^

^*a*^Unless otherwise noted, all of the reactions were carried out using *N*-butyl-pyridine-2-carboxylic acid amide (**1a**) (0.10 mmol) and diazo compound (**2a**) (0.20 mmol) with a metal catalyst (5.0 mol%) in the presence of a silver salt (20 mol%) in solvent (1.0 mL) at 100 °C for 24 h under Ar in a sealed reaction tube, followed by flash chromatography on SiO_2_.

^*b*^Isolated yield.

^*c*^TFE refers to 2,2,2-trifluoroethanol.

^*d*^The reaction temperature is 80 °C.

^*e*^The reaction temperature is 110 °C.

With this protocol in hand, the scope of the Rh(iii)-catalyzed *N*-methylene C–H carbenoid insertion of *N*-butyl-pyridine-2-carboxylic acid amide (**1a**) was first investigated with a range of diazo compounds **2** (Table 2). As illustrated for **3a–3i**, diacceptor- and donor/acceptor-substituted diazo compounds underwent smooth cross-coupling reactions with *N*-methylene C–H bonds to furnish beta-amino esters (**3a–3i**, 43–91% yields). Among them, alpha-diazo-beta-ketoesters participated in the transformation to produce alpha-acyl-beta-amino esters (**3a** and **3b**, 89% and 43% yield, respectively). Moreover, various alpha-diazo-beta-arylesters are also applicable to the present transformation, leading to the formation of the corresponding alpha-aryl and beta-amino esters, in which the substituent on the aryl ring had an important effect on the yield of the reaction. These alpha-diazo-beta-arylesters with electron-deficient phenyl rings gave the products in moderate to excellent yields (**3c–3h**, 50–91% yields). On the contrary, compared with diazo-phenyl-acetic acid methyl ester (**3i**, 51% yield), when an electron-richer aryl group-containing diazo ester such as diazo-(4-methoxy-phenyl)-acetic acid methyl ester was used, an unexpected alkene **3k** (*Z*/*E* = 3 : 1) was formed in 67% yield, and no desired beta-amino ester **3j** was observed.

**Table 2:** Substrate scope^*a*^ ^,^^*b*^ ^,^^*c*^

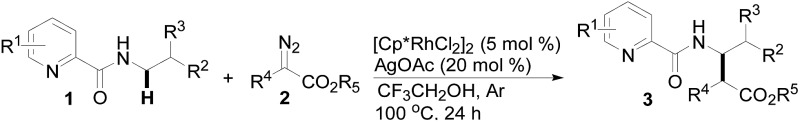
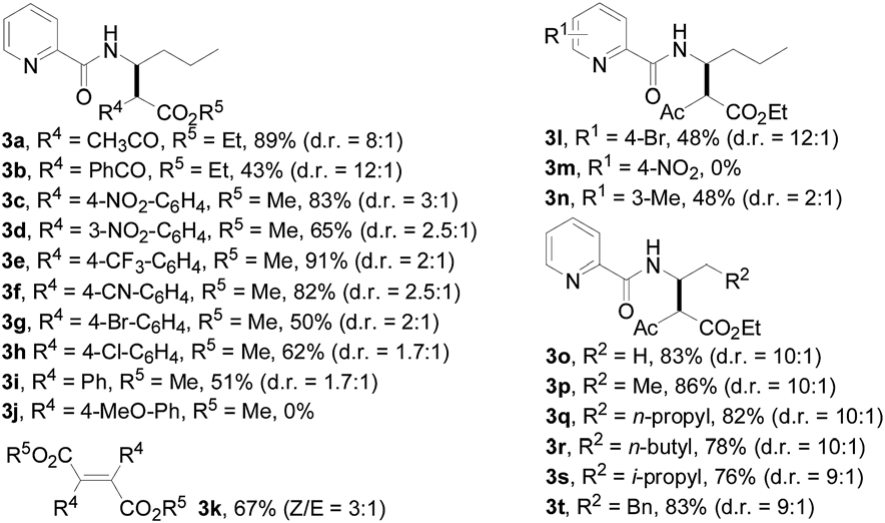
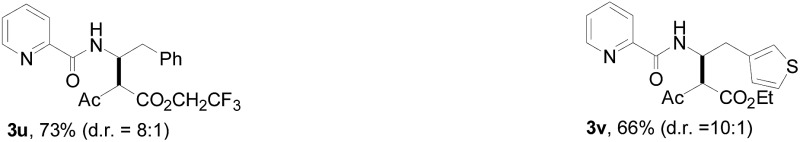
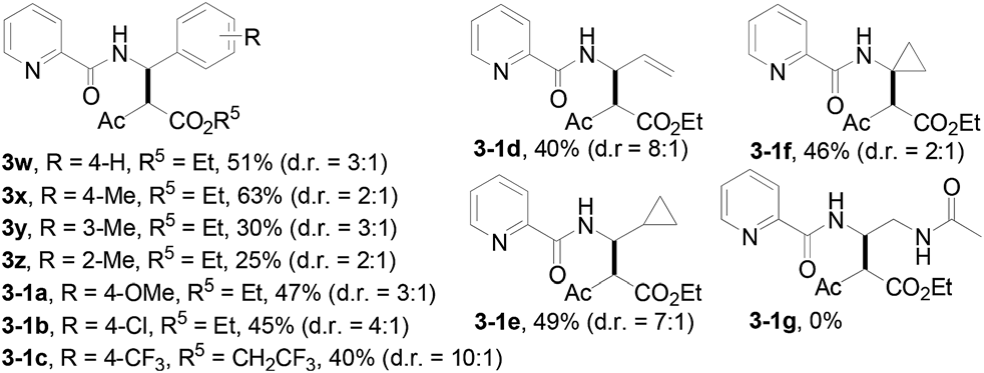

^*a*^All of the reactions were carried out using amides (**1**) (0.10 mmol) and diazo compounds (**2**) (0.20 mmol) with [Cp*RhCl_2_]_2_ (5.0 mol%) in the presence of AgOAc (20 mol%) in 2,2,2-trifluoroethanol (1.0 mL) at 100 °C for 24 h under Ar in a sealed reaction tube, followed by flash chromatography on SiO_2_.

^*b*^Isolated yield.

^*c*^d.r. values were determined by ^1^H NMR spectroscopy, please see ESI.

Subsequently, we prepared the 3- or 4-substituted pyridine-2-carboxylic acid butylamides, and investigated the substitution effect of pyridine moieties on *N*-methylene Csp^3^–H bond carbenoid insertion with 2-diazo-3-oxo-butyric acid methyl ester **2a**. It was found that introducing a methyl group or bromo group into the 3- or 4-position of the pyridine ring could lead to moderate yields of **3l** and **3n** (48%), and a pyridine ring with a strong electron-withdrawing group (–NO_2_) was not tolerated for this transformation (**3m**).

The scope of the present procedure with regard to different types of *N*-amido alkane has been further evaluated. Compared with the *N-n*-butyl-substituted amide (**1a**), the shorter or longer straight-chain alkylamine-based amides could be smoothly regioselectively installed a Csp^3^–Csp^3^ bond into the alpha-position of the alkylamine moiety in 78–86% yields (**3o–3r**). Branched-chain 3-methyl-butylamine-based amides and phenylpropylamine- or phenylethylamine-based amides could also tolerate this reaction system and afforded structurally complex beta-amino acid derivatives **3s** (76%), **3t** (83%) and **3u** (73%). Meanwhile, we also observed that the intermolecular *N*-methylene Csp^3^–H bond carbenoid insertion of 2-thiophen-3-yl-ethylamine-based amides also proceeded well to give a 66% yield of **3v**, and thiophenyl C–H carbenoid insertion did not occur.^15^ However, *N*-benzyl-substituted amides made the transformation a little sluggish, possibly due to steric hindrance from the phenyl ring suppressing the *N*-methylene Csp^3^–H bond insertion (**3w–3z**and **3-1a–3-1c**). To our surprise, the present protocol was also applicable to an alkenyl functional group-containing amidoalkane, in which the carbon–carbon double bond could be kept intact (**3-1d**, 40% yield).^16^ More importantly, in addition to the *N*-allylamide, the *N*-cyclopropylmethyleneamine-based amide was also amenable to the reaction, furnishing the desired beta-cyclopropyl-beta-amino ester **3-1e** in a 49% yield. This transformation was not only limited to the *N*-methylene Csp^3^–H bond;^17^*N*-cyclopropylamide could also couple with diazoester **2a** through an *N*-methyne Csp^3^–H bond carbenoid insertion to provide the target product **3-1f** (46%). Unfortunately, pyridine-2-carboxylic acid (2-acetylamino-ethyl)-amide was not tolerated for this transformation, possibly due to the coordination between Rh(iii) and 1,2-bisamide “N” inhibiting the Csp^3^–H bond carbenoid insertion (**3-1g**, 0%). Finally, the post-synthetic utility of this transformation revealed that 2-pyridyl carboxyamide **3e** could be smoothly converted into a N–H free beta-amino acid (**4a**) in a 67% yield *via* a one-pot process (see ESI† for more details).

**Scheme 2:**
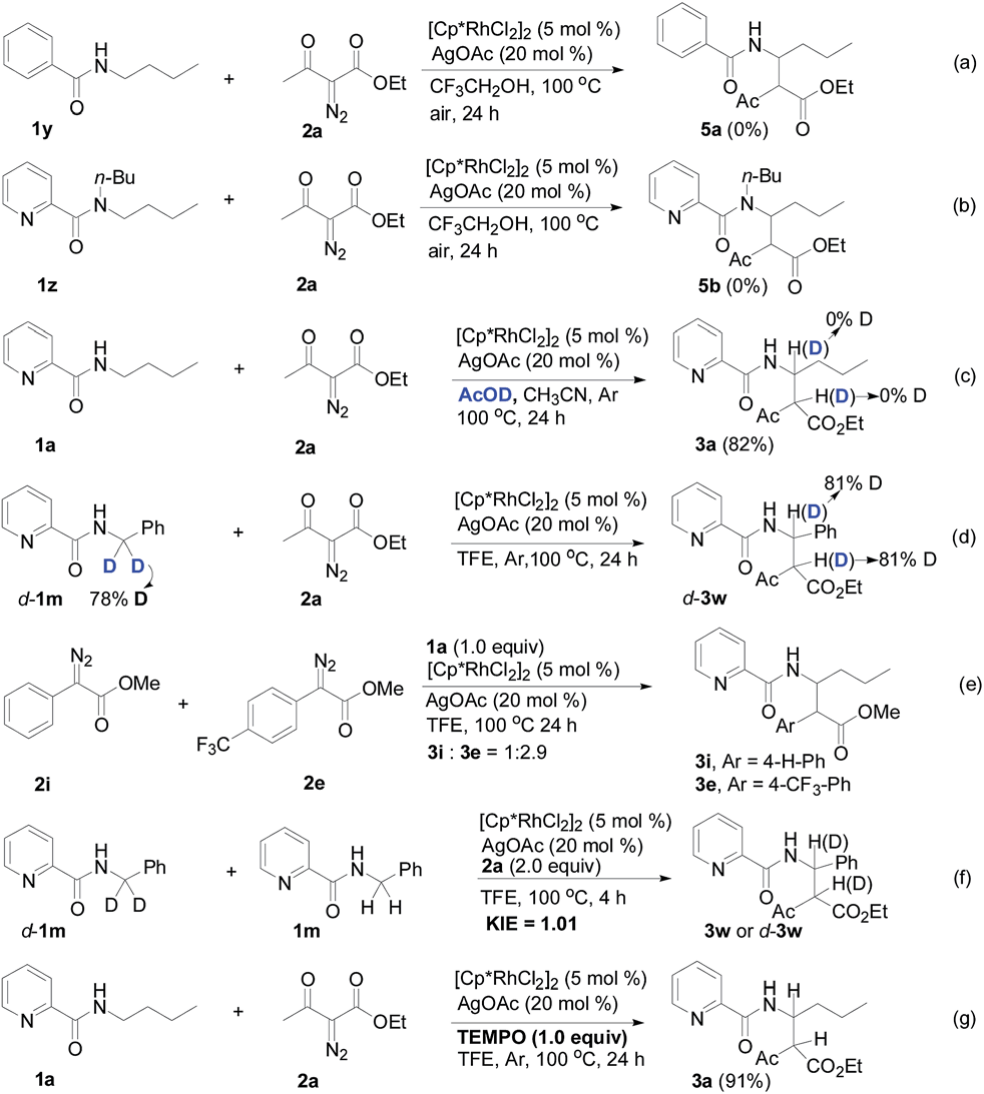
Preliminary mechanism studies.

**Fig. 1:**
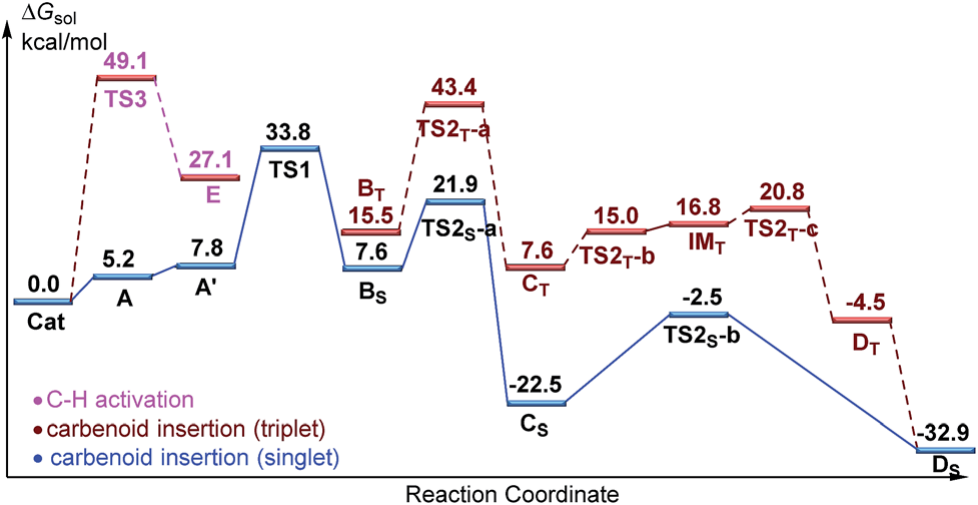
The free energy profiles for the Rh(iii)-catalyzed regioselective *N*-methylene Csp^3^–H bond carbenoid functionalization. The free energies are reported in kcal mol^–1^ at the M06-L/BSII/SMD(2,2,2-trifluoroethanol)//M06-L/BSI level of theory.

Designed control experiments, as well as DFT studies (see ESI† for more details), were performed to elucidate the plausible reaction mechanism (Scheme 2). Treatment of *N*-butyl-benzamide (**1y**) or *N*,*N*-dibutyl-benzamide (**1z**) with alpha-diazo ester (**2a**) under our standard conditions did not provide the corresponding target products **5a** or **5b** (Scheme 2a and b),^18^ and thus demonstrated that the pyridyl group and amide “N” played a significant bichelate-directing role in enabling the *N*-methylene C–H carbenoid insertion. Meanwhile, when **1a** was subjected to 1.0 equiv. of AcOD in the presence of diazo compound **2a**, no H/D exchange was detected at the alpha- or beta-position of the beta-aminoester **3a** (Scheme 2c). Although this experiment implied that an irreversible concerted metalation–deprotonation (CMD) process followed by metal protonation was possibly involved in this transformation,^19^ our DFT study further excluded an inner-sphere mechanism *via* bidentate-assisted *N*-methylene Csp^3^–H bond activation, which is required to overcome an activation free energy of 49.1 kcal mol^–1^ (**TS3**, Fig. S-5, ESI†) due to the three-membered ring strain. Moreover, treatment of d-**1m** (78% D) with **2a** afforded the deuterated product d-**3w**, in which 81% D was inserted at both the alpha- and beta-positions of the beta-amino ester d-**3w** (Scheme 2d); this result clearly indicated that a two-electron carbenoid insertion into the Csp^3^–H bond occurred in the presence of bidentate–chelation assistance. DFT calculations (Fig. 1, detailed pathways are shown in Fig. S-5, ESI†) were carried out to further confirm the carbenoid insertion process. In the Rh-carbenoid formation stage, the Rh-carbenoid is formed *via* transition state **TS1**, with a calculated activation free energy of 33.8 kcal mol^–1^ (**Cat** → **TS1**). Subsequently, we further evaluated both the singlet and the triplet carbenoid insertion pathways. The corresponding DFT results suggest that the carbenoid insertion proceeds in a singlet Fischer type carbene manner (21.9 kcal mol^–1^, **TS2_S_-a**; –2.5 kcal mol^–1^, **TS2_S_-b**), and the triplet pathway through radical recombination is less feasible due to the high activation free energy (43.4 kcal mol^–1^, **TS2_T_-a**). The singlet carbene pathway was further confirmed by the control experiment (Scheme 2g), in which using TEMPO (1.0 equiv.) did not significantly decrease the reaction yield (91% of **3a**).

The *N*-methylene C–H carbenoid insertion between alpha-aryl-alpha-diazo esters differing in electronic effects indicates that an electron-deficient diazo compound tended to form a rhodium carbene at a relatively higher rate (Scheme 2e). Moreover, a competitive cross-coupling of an equimolar mixture of d-**1m** and **1m** with alpha-diazo ester **2a** also gave a *k*_H_/*k*_D_ value of 1.01 on the basis of the ^1^H NMR spectrum (Scheme 2f), suggesting that C–H bond carbenoid insertion did not involve the rate-limiting step of this transformation. These results are further supported by our DFT data, which demonstrated that the formation of Rh-carbenoid *via***TS1** (Δ*G*^‡^ = 33.8 kcal mol^–1^) should be the rate-determining step.

**Scheme 3:**
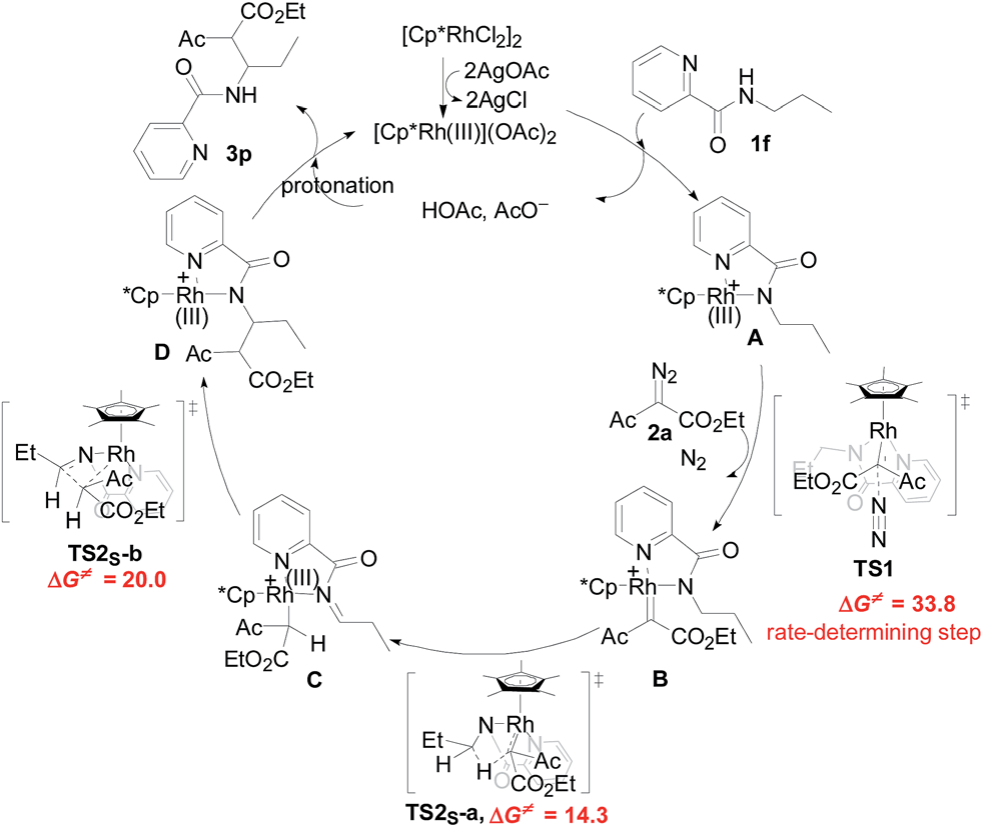
Proposed reaction mechanism.

A mechanism rationale coherent with these results and the DFT studies is proposed in Scheme 3. The initial coordination of the pyridyl nitrogen and amide nitrogen of substrate **1f** to an active Rh(iii) catalyst affords complex **A**. Subsequent interaction of complex **A** with diazo compound **2a** is followed by denitrogenation to generate Rh carbene species **B**. The rhodium complex **B** undergoes a bichelate-assisted singlet Fischer type carbenoid insertion into the *N*-methylene Csp^3^–H bond *via* an outer-sphere pathway, successively producing the corresponding imine intermediate **C** and Rh(iii) complex **D**. Further protonization of complex **D** furnishes the desired beta-amino ester **3p** with the regeneration of the Rh(iii) catalyst.

## Conclusions

In summary, we have developed the first Rh(iii)-catalyzed intermolecular *N*-methylene Csp^3^–H bond carbenoid insertion of acyclic aliphatic amides with high regioselectivity. In these systems, bidentate–chelation acts as a unique platform to enable the cross-coupling of *N*-methylene Csp^3^–H bonds with diazo compounds through the “Outer-Sphere Pathway”. This strategy could have broad implications on future research directions on selective Csp^3^–H functionalization. Moreover, this reaction tolerates a broad scope of substrates and provides an effective approach to diverse beta-amino esters. Further efforts to achieving an asymmetric version of this transformation are underway.

## Conflicts of interest

There are no conflicts to declare.

## Supplementary Material

Supplementary information
