# Supplementary material for: Rh(iii)-catalyzed regioselective intermolecular N-methylene Csp3–H bond carbenoid insertion
Source: Chem Sci. 2017 Nov 27;9(4):985–9. doi: 10.1039/c7sc03802j (PMC5874982; doi:10.1039/c7sc03802j)

Electronic Supplementary Material (ESI) for Chemical Science.
This journal is © The Royal Society of Chemistry 2017

Supporting Information

Rh(III)-Catalyzed Regioselective Intermolecular *N*-Methylene Csp^3-H Bond Carbenoid Insertion

Haisheng Xie,[a] Zongren Ye,[b] Zhuofeng Ke*[b] Jianyong Lan,[a] Huanfeng Jiang,*[a] and Wei Zeng*[a]

[a] School of Chemistry and Chemical Engineering, South China University of Technology, Guangzhou 510641, China

[b] School of Materials Science & Engineering, PCFM Lab, Sun Yat-sen University, Guangzhou 510275, China

Table of Contents

Supporting Information......1

I. General Methods......3

II. Experimental Procedures for the Preparation of Starting Materials 3

1. Preparation of picolinamides. 3
2. Preparation of diazo compounds 7
3. Procedure for the preparation of the *d*-**1m**......8

III. Experimental Procedure for Optimizing the Reaction Conditions......8

IV. Rh(III)-Catalyzed Regioselective Intermolecular *N*-Methylene Csp^3-H Bond Carbenoid Insertion......10

1. General procedure for the Rh(III)-catalyzed regioselective intermolecular *N*-methylene Csp^3-H bond carbenoid insertion......10
2. Synthetic application of this transformation 10
3. Spectroscopic data of the reaction products 11

V. Control Experiments for Mechanism Studies......19

1. Procedure for the Rh(III)-catalyzed Csp^3-H bond carbenoid insertion of *N*-butylbenzamide (**1w**) with ethyl 2-diazo-3-oxobutanoate **(2a)** 19
2. Procedure for the Rh(III)-catalyzed Csp^3-H bond carbenoid insertion of *N,N*-dibutylpicolinamide

(**1t**) with ethyl 2-diazo-3-oxobutanoate (**2a**) **19**

3. Rh(III)-catalyzed Csp^3-H carbenoid insertion of **1a** with **2a** in different deuterated solvent system **19**

4. Rh(III)-catalyzed Csp^3-H carbenoid insertion of *d*-**1m** with **2a**...**21**

5. Kinetic isotope effect for this transformation..**22**

6. Competition experiment for different diazo compounds differing in electron effects **23**

7. The effect of TEMPO on this transformation..**24**

VI. Computational details...25

VII. Reference 41

VIII. Spectral Copies of ^{1}H and ^{13}C NMR of Compounds Obtained in This Study........................42

I. General Methods

All reactions were carried out in flame-dried sealed tubes with magnetic stirring. Unless otherwise noted, all experiments were performed under argon atmosphere. All reagents were purchased from TCI, Acros or Strem. Solvents were treated with 4 Å molecular sieves or sodium and distilled prior to use. Purifications of reaction products were carried out by flash chromatography using Qingdao Haiyang Chemical Co. Ltd silica gel (300-400 mesh). Infrared spectra (IR) were recorded on a Brucker TENSOR 27 FTIR spectrophotometer and are reported as wavelength numbers (cm^{-1}). Infrared spectra were recorded by preparing a KBr pellet containing the title compounds. ^{1}H NMR and ^{13}C NMR spectra were recorded with tetramethylsilane (TMS) as internal standard at ambient temperature unless otherwise indicated on a Bruker Avance DPX 600 fourier Transform spectrometer operating at 400 MHz for ^{1}H NMR and 100 MHz for ^{13}C NMR. Chemical shifts are reported in parts per million (ppm) and coupling constants are reported as Hertz (Hz). Splitting patterns are designated as singlet (s), broad singlet (bs), doublet (d), triplet (t). Splitting patterns that could not be interpreted or easily visualized are designated as multiple (m). Low resolution mass spectra were recorded using a Waters HPLC/ZQ4000 Mass Spectrometer. High resolution mass spectra (HR-MS) were recorded on an IF-TOF spectrometer (Micromass). Gas chromatograph mass spectra were obtained with a SHIMADZU model GCMS-QP5000 spectrometer.

II. Experimental procedures for the preparation of starting materials

1. General procedure for the preparation of the picolinamides from amines.[1]

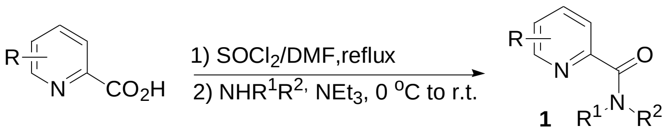

All of the picolinamides including **1a-1x** were obtained according to the following procedure.[1] To a solution of the picolinic acid (5.0 mmol) in DCM (20 mL) at room temperature was added $SOCl_2$ (4 mL) and one drop of dry DMF. The reaction was allowed to stir at 80 ℃ for 4 hours. The solvent was then removed under reduced pressure to afford the corresponding crude acid chloride. Then DCM (20 mL) was added and the solution was cooled to 0 ℃ followed by dropwise addition of NEt_3 (1.5 mL) and amine (10.0 mmol, 2.0 eq.). The reaction mixture was stirred at r.t. overnight, extracted by DCM. The organic layer was dried over Na_2SO_4 and the solvent was evaporated, then purified through flash chromatography on silica gel with ethyl acetate/petroleum (v/v = 1/2) as the eluent to afford the desired products.

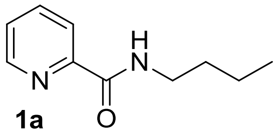

1a

***N*-Butylpicolinamide (1a)**[1]**:** **^{1}H NMR** (400 MHz, $CDCl_3$) δ 8.54 (d, *J* = 4.5 Hz, 1H), 8.20 (d, *J* = 7.8 Hz, 1H), 8.06 (s, 1H), 7.84 (t, *J* = 7.7 Hz, 1H), 7.46 – 7.39 (m, 1H), 3.48 (q, *J* = 13.4, 6.8 Hz, 2H), 1.70 – 1.57 (m, 2H), 1.50 – 1.38 (m, 2H), 0.96 (t, *J* = 7.3 Hz, 3H); **^{13}C NMR** (100 MHz, $CDCl_3$) δ 164.2, 150.1, 148.0, 137.3, 126.0, 122.2, 39.1, 31.7, 20.2, 13.8. **MS (ESI):** m/z= 178.1 $[M]^{+}$.

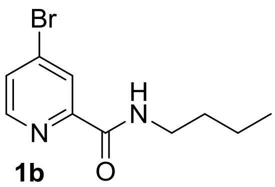

1b

4-Bromo-*N*-butylpicolinamide (1b)[1]**:** ^{1}**H NMR** (400 MHz, $CDCl_3$) δ 8.28 (d, *J* = 6.3 Hz, 2H), 7.96 (s, 1H), 7.51 (d, *J* = 5.1 Hz, 1H), 3.40 (q, *J* = 6.7 Hz, 2H), 1.60 – 1.48 (m, 2H), 1.40 – 1.29 (m, 2H), 0.88 (t, *J* = 7.3 Hz, 3H); ^{13}**C NMR** (100 MHz, $CDCl_3$) δ 162.9, 151.2, 148.7, 134.3, 129.1, 125.7, 39.2, 31.6, 20.1, 13.7. **MS (ESI):** m/z= 256.0 $[M]^+$.

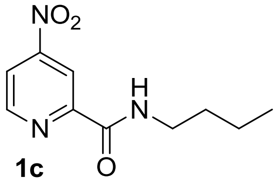

1c

***N*-Butyl-4-nitropicolinamide (1c)**[1]**:** ^{1}**H NMR** (400 MHz, $CDCl_3$) δ 8.79 (d, *J* = 3.8 Hz, 2H), 8.12 – 8.07 (m, 1H), 7.93 (s, 1H), 3.44 (dd, *J* = 13.6, 6.8 Hz, 2H), 1.61 – 1.52 (m, 2H), 1.41 – 1.31 (m, 2H), 0.89 (t, *J* = 7.3 Hz, 3H); ^{13}**C NMR** (100 MHz, $CDCl_3$) δ 162.1, 155.1, 153.4, 150.4, 118.4, 115.2, 39.5, 31.6, 20.1, 13.7. **MS (ESI):** m/z= 223.1 $[M]^+$.

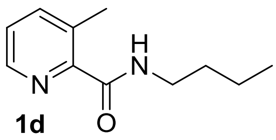

1d

***N*-Butyl-3-methylpicolinamide (1d)**[1]**:** ^{1}**H NMR** (400 MHz, $CDCl_3$) δ 8.36 (d, *J* = 3.7 Hz, 1H), 8.13 (s, 1H), 7.56 (d, *J* = 7.7 Hz, 1H), 7.30 – 7.25 (m, 1H), 3.42 (q, *J* = 6.7 Hz, 2H), 2.73 (s, 3H), 1.68 – 1.54 (m, 2H), 1.43 (dt, *J* = 14.8, 7.4 Hz, 2H), 0.95 (t, *J* = 7.3 Hz, 3H); ^{13}**C NMR** (100 MHz, $CDCl_3$) δ 166.0, 147.5, 145.4, 140.8, 135.3, 125.5, 39.0, 31.8, 20.6, 20.2, 13.8. **MS (ESI):** m/z= 192.1 $[M]^+$.

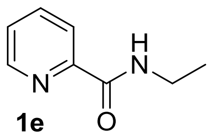

1e

***N*-Ethylpicolinamide (1e)**[1]**:** ^{1}**H NMR** (400 MHz, $CDCl_3$) δ 8.52 (d, *J* = 4.6 Hz, 1H), 8.18 (d, *J* = 7.8 Hz, 1H), 8.04 (s, 1H), 7.82 (t, *J* = 7.7 Hz, 1H), 7.44 – 7.36 (m, 1H), 3.54 – 3.45 (m, 2H), 1.25 (t, *J* = 7.3 Hz, 3H); ^{13}**C NMR** (100 MHz, $CDCl_3$) δ 164.2, 150.1, 148.0, 137.3, 126.0, 122.1, 34.3, 14.8. **MS (ESI):** m/z= 150.1 $[M]^+$.

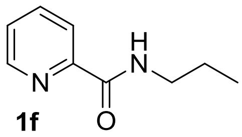

1f

***N*-Propylpicolinamide (1f)**[1]**:** ^{1}**H NMR** (400 MHz, $CDCl_3$) δ 8.53 (d, *J* = 4.3 Hz, 1H), 8.19 (d, *J* = 7.8 Hz, 1H), 8.09 (s, 1H), 7.86 – 7.80 (m, 1H), 7.44 – 7.37 (m, 1H), 3.43 (dd, *J* = 13.4, 7.0 Hz, 2H), 1.72 – 1.60 (m, 2H), 0.99 (t, *J* = 7.4 Hz, 3H); ^{13}**C NMR** (100 MHz, $CDCl_3$) δ 164.3, 150.1, 148.0, 137.3, 126.0, 122.2, 41.1, 22.9, 11.5. **MS (ESI):** m/z= 164.1 $[M]^+$.

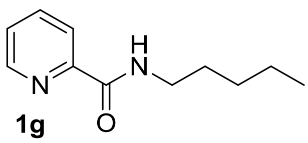

1g

***N*-Pentylpicolinamide (1g)**[1]**:** ^{1}**H NMR** (400 MHz, $CDCl_3$) δ 8.54 (d, *J* = 4.7 Hz, 1H), 8.20 (d, *J* = 7.8 Hz, 1H), 8.07 (s, 1H), 7.84 (t, *J* = 7.6 Hz, 1H), 7.45 – 7.38 (m, 1H), 3.47 (q, *J* = 6.1 Hz, 2H), 1.70 – 1.58 (m, 2H), 1.45 – 1.29 (m, 4H), 0.91 (t, *J* = 5.8 Hz, 3H); ^{13}**C NMR** (100 MHz, $CDCl_3$) δ 164.2, 150.1, 148.0, 137.3, 126.0, 122.2, 39.4, 29.3, 29.1, 22.4, 14.0. **MS (ESI):** m/z= 192.1 $[M]^+$.

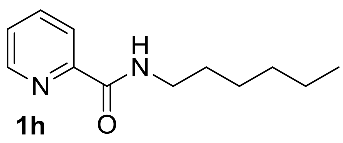

1h

***N*-Hexylpicolinamide (1h)**[1]**:** ^{1}**H NMR** (400 MHz, $CDCl_3$) δ 8.53 (d, *J* = 4.6 Hz, 1H), 8.19 (d, *J* = 7.8 Hz, 1H), 8.07 (s, 1H), 7.82 (t, *J* = 7.7 Hz, 1H), 7.43 – 7.36 (m, 1H), 3.45 (q, *J* = 6.7

Hz, 2H), 1.67 – 1.56 (m, 2H), 1.44 – 1.35 (m, 2H), 1.33 – 1.26 (m, 4H), 0.87 (t, *J* = 6.2 Hz, 3H); **^{13}C NMR** (100 MHz, $CDCl_3$) δ 164.2, 150.1, 148.0, 137.3, 126.0, 122.2, 39.5, 31.5, 29.6, 26.7, 22.5, 14.0. **MS (ESI):** m/z= 206.1 $[M]^+$.

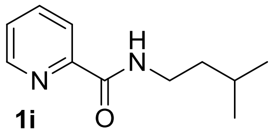

1i

***N*-Isopentylpicolinamide (1i)**[1]: **^{1}H NMR** (400 MHz, $CDCl_3$) δ 8.50 (d, *J* = 4.4 Hz, 1H), 8.17 (d, *J* = 7.8 Hz, 1H), 8.03 (s, 1H), 7.80 (t, *J* = 8.3 Hz, 1H), 7.42 – 7.34 (m, 1H), 3.46 (dd, *J* = 14.0, 6.7 Hz, 2H), 1.72 – 1.61 (m, 1H), 1.51 (dd, *J* = 14.6, 7.0 Hz, 2H), 0.93 (s, 3H), 0.92 (s, 3H); **^{13}C NMR** (100 MHz, $CDCl_3$) δ 164.2, 150.1, 148.0, 137.3, 126.0, 122.1, 38.5, 37. 7, 25.8, 22.4. **MS (ESI):** m/z= 192.1 $[M]^+$.

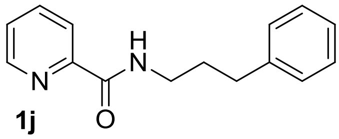

1j

***N*-(3-Phenylpropyl)picolinamide (1j)**[1]**:** **^{1}H NMR** (400 MHz, $CDCl_3$) δ 8.55 (d, *J* = 4.6 Hz, 1H), 8.22 (d, *J* = 7.8 Hz, 1H), 8.17 (s, 1H), 7.90 – 7.83 (m, 1H), 7.43 (dd, *J* = 7.5, 4.8 Hz, 1H), 7.30 (t, *J* = 7.5 Hz, 2H), 7.25 – 7.19 (m, 3H), 3.53 (dd, *J* = 13.5, 6.8 Hz, 2H), 2.77 – 2.71 (m, 2H), 2.05 – 1.96 (m, 2H); **^{13}C NMR** (100 MHz, $CDCl_3$) δ 164.5, 149. 9, 148.0, 141.4, 137.5, 128.4, 128.4, 126.2, 126.0, 122.3, 39.1, 33.3, 31.2; **MS (ESI)**: m/z= 240.1 $[M]^+$.

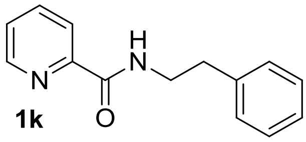

1k

***N*-Phenethylpicolinamide (1k)**[1]**:** **^{1}H NMR** (400 MHz, $CDCl_3$) δ 8.51 (d, *J* = 4.6 Hz, 1H), 8.21 (d, *J* = 7.8 Hz, 1H), 8.18 (s, 1H), 7.83 (td, *J* = 7.7, 1.6 Hz, 1H), 7.44 – 7.36 (m, 1H), 7.36 – 7.29 (m, 2H), 7.25 (dd, *J* = 14.2, 7.1 Hz, 3H), 3.75 (dd, *J* = 13.7, 7.0 Hz, 2H), 2.96 (t, *J* = 7.3 Hz, 2H); **^{13}C NMR** (100 MHz, $CDCl_3$) δ 164.3, 149.9, 148.1, 140.0, 137.3, 128.8, 128.6, 126.5, 126.1, 122.2, 40.8, 36.0. **MS (ESI):** m/z= 226.1 $[M]^+$.

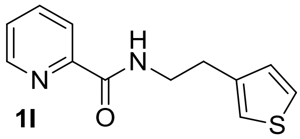

1l

***N*-(2-(Tthiophen-3-yl)ethyl)picolinamide (1l)**[1]**:** **^{1}H NMR** (400 MHz, $CDCl_3$) δ 8.54 (d, *J* = 4.5 Hz, 1H), 8.25 (s, 1H), 8.22 (d, *J* = 7.8 Hz, 1H), 7.85 (t, *J* = 7.7 Hz, 1H), 7.47 – 7.36 (m, 1H), 7.18 (d, *J* = 5.1 Hz, 1H), 7.01 – 6.95 (m, 1H), 6.91 (s, 1H), 3.78 (q, *J* = 6.7 Hz, 2H), 3.18 (t, *J* = 6.9 Hz, 2H); **^{13}C NMR** (100 MHz, $CDCl_3$) δ 164.4, 149.9, 148.1, 141.3, 137.3, 127.0, 126.2, 125.3, 123. 9, 122.2, 40.9, 30.1; **MS (ESI):** m/z= 232.1 $[M]^+$.

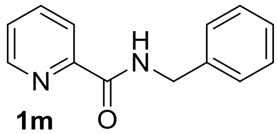

1m

***N*-Benzylpicolinamide (1m)**[1]**:** **^{1}H NMR** (400 MHz, $CDCl_3$) δ 8.52 (d, *J* = 4.7 Hz, 1H), 8.39 (s, 1H), 8.23 (d, *J* = 7.8 Hz, 1H), 7.84 (t, *J* = 7.7 Hz, 1H), 7.45 – 7.31 (m, 5H), 7.30 – 7.25 (m, 1H), 4.67 (d, *J* = 6.1 Hz, 2H); **^{13}C NMR** (100 MHz, $CDCl_3$) δ 164.3, 149.8, 148.1, 138.3, 137.4, 128.7, 127.9, 127.5, 126.2, 122.4, 43.5. **MS (ESI):** m/z= 212.1 $[M]^+$.

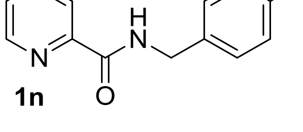

1n

***N*-(4-Methylbenzyl)picolinamide (1n)**[1]**:** **^{1}H NMR** (400 MHz, $CDCl_3$) δ 8.50 (d, *J* = 4.2 Hz, 1H), 8.36 (s, 1H), 8.23 (d, *J* = 7.8 Hz, 1H), 7.84 (td, *J* = 7.7, 1.5 Hz, 1H), 7.40 (dd, *J* = 7.4, 4.9 Hz, 1H), 7.26 (d, *J* = 7.9 Hz, 2H), 7.15 (d, *J* = 7.8 Hz, 2H), 4.62 (d, *J* = 6.0 Hz, 2H), 2.33 (s, 3H); **^{13}C NMR** (100 MHz, $CDCl_3$) δ 164.3, 149.9, 148.1, 137.4, 137.1, 135.2, 129.4, 127.9, 126.2, 122.4, 43.3, 21.1. **MS (ESI):** m/z= 226.1 $[M]^+$.

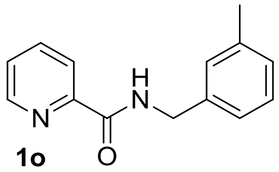

1o

***N*-(3-Methylbenzyl)picolinamide (1o)**[1]**:** **^{1}H NMR** (400 MHz, $CDCl_3$) δ 8.54 (d, *J* = 4.7 Hz, 1H), 8.26 (d, *J* = 7.8 Hz, 2H), 7.87 (td, *J* = 7.7, 1.4 Hz, 1H), 7.47 – 7.41 (m, 1H), 7.38 – 7.32 (m, 1H), 7.26 – 7.17 (m, 3H), 4.69 (d, *J* = 5.8 Hz, 2H), 2.40 (s, 3H); **^{13}C NMR** (100 MHz, $CDCl_3$) δ 164.1, 149.9, 148.1, 137.4, 136.5, 135.9, 130.5, 128.6, 127.7, 126.2, 122.3, 41.7, 19.1. **MS (ESI):** m/z= 226.1 $[M]^+$.

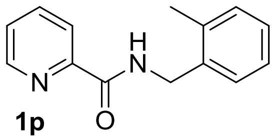

1p

***N*-(2-Methylbenzyl)picolinamide (1p)**[1]**:** **^{1}H NMR** (400 MHz, $CDCl_3$) δ 8.55 (d, *J* = 4.5 Hz, 1H), 8.38 (s, 1H), 8.26 (d, *J* = 7.8 Hz, 1H), 7.87 (t, *J* = 7.0 Hz, 1H), 7.47 – 7.41 (m, 1H), 7.26 (dd, *J* = 12.4, 4.9 Hz, 1H), 7.19 (d, *J* = 9.0 Hz, 2H), 7.12 (d, *J* = 7.4 Hz, 1H), 4.66 (d, *J* = 6.1 Hz, 2H), 2.37 (s, 3H); **^{13}C NMR** (100 MHz, $CDCl_3$) δ 164.2, 149.9, 148.1, 138.4, 138.2, 137.4, 128.6, 128.2, 126.2, 124.9, 122.4, 43.5, 21.4. **MS (ESI):** m/z= 226.1 $[M]^+$.

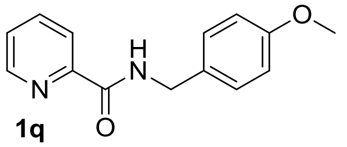

1q

***N*-(4-Methoxybenzyl)picolinamide (1q)**[1]**:** **^{1}H NMR** (400 MHz, $CDCl_3$) δ 8.53 (d, *J* = 4.6 Hz, 1H), 8.35 (s, 1H), 8.25 (d, *J* = 7.8 Hz, 1H), 7.87 (t, *J* = 7.7 Hz, 1H), 7.46 – 7.40 (m, 1H), 7.31 (d, *J* = 8.5 Hz, 2H), 6.89 (d, *J* = 8.5 Hz, 2H), 4.62 (d, *J* = 6.0 Hz, 2H), 3.81 (s, 3H); **^{13}C NMR** (100 MHz, $CDCl_3$) δ 164.2, 159.0, 149.9, 148.1, 137.4, 130.3, 129.2, 126.2, 122.4, 114.1, 55.3, 43.0. **MS (ESI):** m/z= 242.1 $[M]^+$.

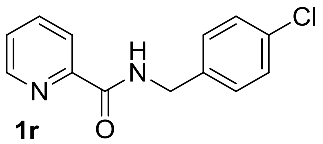

1r

***N*-(4-Chlorobenzyl)picolinamide (1r)**[1]**:** **^{1}H NMR** (400 MHz, $CDCl_3$) δ 8.54 (d, *J* = 4.6 Hz, 1H), 8.42 (s, 1H), 8.24 (d, *J* = 7.8 Hz, 1H), 7.87 (td, *J* = 7.7, 1.6 Hz, 1H), 7.47 – 7.40 (m, 1H), 7.31 (s, 4H), 4.65 (d, *J* = 6.2 Hz, 2H); **^{13}C NMR** (100 MHz, $CDCl_3$) δ 164.3, 149.7, 148.1, 137.4, 136.7, 133.3, 129.2, 128.8, 126.3, 122.4, 42.8. **MS (ESI):** m/z= 246.1 $[M]^+$.

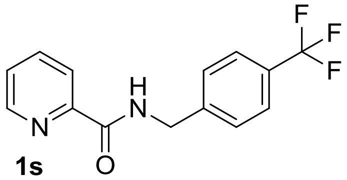

1s

***N*-(4-(Trifluoromethyl)benzyl)picolinamide (1s)**[1]**:** **^{1}H NMR** (400 MHz, $CDCl_3$) δ 8.54 (d, *J* = 4.5 Hz, 2H), 8.24 (d, *J* = 7.8 Hz, 1H), 7.87 (t, *J* = 7.7 Hz, 1H), 7.59 (d, *J* = 8.0 Hz, 2H), 7.52 – 7.40 (m, 3H), 4.74 (d, *J* = 6.3 Hz, 2H); **^{13}C NMR** (100 MHz, $CDCl_3$) δ 164.5, 149.6, 148.2, 142.4, 137.5, 127.9, 126.4, 125.6, 122.4, 42.9. **MS (ESI):** m/z= 280.1 $[M]^+$.

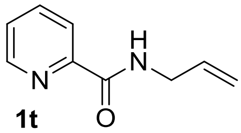

1t

***N*-Allylpicolinamide (1t)**[1,4]**:** **^{1}H NMR** (400 MHz, $CDCl_3$) δ 8.51 (d, *J* = 2.5 Hz, 1H), 8.16 (d, *J* = 7.7 Hz, 2H), 7.80 (t, *J* = 7.7 Hz, 1H), 7.43 – 7.34 (m, 1H), 5.91 (qd, *J* = 10.6, 6.4 Hz, 1H), 5.23 (d, *J* = 17.1 Hz, 1H), 5.13 (d, *J* = 10.2 Hz, 1H), 4.07 (t, *J* = 5.1 Hz, 2H); **^{13}C NMR** (100 MHz, $CDCl_3$) δ 164.2, 149.9, 148.1, 137.3, 134.1, 126.2, 122.3, 116.4, 41. 8. **MS (ESI):** m/z= 162.1 $[M]^+$.

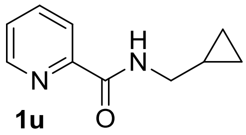

1u

***N*-(Cyclopropylmethyl)picolinamide (1u)**[1]**:** **^{1}H NMR** (400 MHz, $CDCl_3$) δ 8.56 (d, *J* = 4.6 Hz, 1H), 8.20 (d, *J* = 7.8 Hz, 1H), 8.16 (s, 1H), 7.84 (t, *J* = 7.7 Hz, 1H), 7.42 (dd, *J* = 6.8, 5.4 Hz, 1H), 3.34 (t, *J*

= 6.4 Hz, 2H), 1.14 – 1.03 (m, 1H), 0.56 (d, *J* = 8.0 Hz, 2H), 0.30 (d, *J* = 4.7 Hz, 2H); **^{13}C NMR** (100 MHz, $CDCl_3$) δ 164.2, 150.1, 148.0, 137.3, 126.0, 122.2, 44.2, 10.8, 3.5. **MS (ESI):** m/z= 176.1 $[M]^+$.

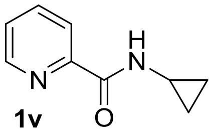
1v

***N*-Cyclopropylpicolinamide (1v)**[1]**: ^{1}H NMR** (400 MHz, $CDCl_3$) δ 8.47 (d, *J* = 4.7 Hz, 1H), 8.16 (d, *J* = 7.8 Hz, 1H), 8.05 (s, 1H), 7.80 (t, *J* = 7.7 Hz, 1H), 7.42 – 7.36 (m, 1H), 2.96 – 2.87 (m, 1H), 0.88 – 0.78 (m, 2H), 0.67 – 0.60 (m, 2H); **^{13}C NMR** (100 MHz, $CDCl_3$) δ 165.7, 149.8, 148.0, 137.3, 126.2, 122.0, 22.5, 6.5. **MS (ESI):** m/z= 162.1 $[M]^+$.

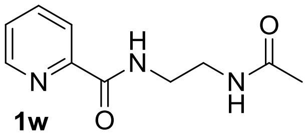
1w

***N*-(2-acetamidoethyl)picolinamide (1w)**[4]**: ^{1}H NMR** (400 MHz, $CDCl_3$) δ 8.55 (d, *J* = 4.3 Hz, 1H), 8.42 (s, 1H), 8.17 (d, *J* = 7.7 Hz, 1H), 7.86 (t, *J* = 7.7 Hz, 1H), 7.50 – 7.38 (m, 1H), 6.51 (s, 1H), 3.62 (dd, *J* = 11.6, 5.8 Hz, 2H), 3.49 (dd, *J* = 11.0, 5.3 Hz, 2H), 1.97 (s, 3H); **^{13}C NMR** (100 MHz, $CDCl_3$) δ 170.8, 165.5, 149.4, 148.1, 137.6, 126.4, 122.3, 40.6, 39.4, 23.2. **MS (ESI):** m/z= 207.1 $[M]^+$.

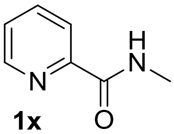
1x

***N*-Methylpicolinamide (1x)**[1]**: ^{1}H NMR** (400 MHz, $CDCl_3$) δ 8.53 (d, *J* = 4.7 Hz, 1H), 8.19 (d, *J* = 7.8 Hz, 1H), 8.05 (s, 1H), 7.84 (td, *J* = 7.7, 1.6 Hz, 1H), 7.41 (dd, *J* = 7.4, 4.8 Hz, 1H), 3.03 (d, *J* = 5.1 Hz, 3H); **^{13}C NMR** (100 MHz, $CDCl_3$) δ 165.0, 150.0, 148.0, 137.3, 126.1, 122.1, 26.1. **MS (ESI):** m/z= 136.1 $[M]^+$.

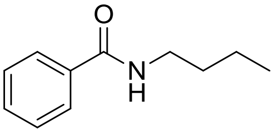
1y

***N*-Butylbenzamide (1w)**[2]**: ^{1}H NMR** (400 MHz, $CDCl_3$) δ 7.77 (dd, *J* = 5.2, 3.3 Hz, 2H), 7.51 – 7.42 (m, 1H), 7.41 – 7.34 (m, 2H), 6.71 (s, 1H), 3.46 – 3.36 (m, 2H), 1.66 – 1.51 (m, 2H), 1.43 – 1.31 (m, 2H), 0.92 (t, *J* = 7.3 Hz, 3H); **^{13}C NMR** (100 MHz, $CDCl_3$) δ 167.7, 134.9 131.2, 128.4, 126.9, 39.8, 31.7, 20.2, 13.8. **MS (ESI):** m/z= 177.1 $[M]^+$.

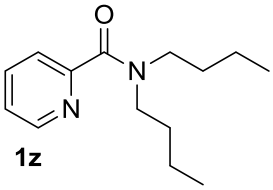
1z

***N,N*-Dibutylbenzamide (1x)**[1]**: ^{1}H NMR** (400 MHz, $CDCl_3$) δ 8.56 (d, *J* = 4.3 Hz, 1H), 7.81 – 7.73 (m, 1H), 7.54 (d, *J* = 7.8 Hz, 1H), 7.34 – 7.27 (m, 1H), 3.53 – 3.47 (m, 2H), 3.36 – 3.30 (m, 2H), 1.67 (dt, *J* = 15.3, 7.6 Hz, 2H), 1.52 (dt, *J* = 15.1, 7.5 Hz, 2H), 1.41 (dq, *J* = 14.8, 7.4 Hz, 2H), 1.18 – 1.08 (m, 2H), 0.96 (t, *J* = 7.3 Hz, 3H), 0.77 (t, *J* = 7.4 Hz, 3H); **^{13}C NMR** (100 MHz, $CDCl_3$) δ 168.9, 155.3, 148.2, 136.8, 124.0, 123.1, 48.5, 45.5, 30.9, 29.7, 20.3, 19.8, 13.9, 13.6. **MS (ESI):** m/z= 234.2 $[M]^+$.

2. Procedure for the preparation of diazo compounds [3]

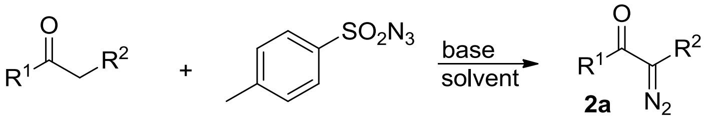

To a solution of *β*-ketoester or *β*-diketone (15 mmol, 1.0 equiv.) and 4-methylbenzenesulfonyl azide (18 mmol, 1.2 equiv.) in CH_3CN (20 mL) at 0 °C was added

DBU (21 mmol, 1.4 equiv.). The resulting solution was stirred at 0 °C for 3 h and slowly brought to r.t. Upon completion as indicated by thin layer chromatography (TLC), the reaction was quenched with water, extracted with ethyl acetate, and dried over anhydrous Na_2SO_4. The reaction mixture was concentrated under reduced pressure, and the crude products were purified by column chromatography.

3. Procedure for the preparation of *d*-1m.[4,5]

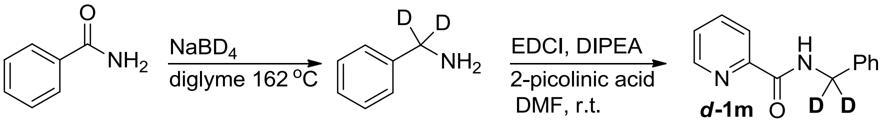

Benzamide (0.726 g, 6 mmol) was readily reduced to benzylamine $NaBD_4$ (26.5 mmol, 1 g) alone in diglyme for 1.5 h at 162 °C (reflux). The deuterated benzylamine was the only observed product. The product was characterized by GC/MS versus authentic benzylamine and the crude products were purified by column chromatography.

***d*-1m** (0.69 g, 5 mmol), 2-picolinic acid (0.74 g, 6 mmol), EDCI (1.16 g, 6 mmol), HOBt (0.92 g, 6 mmol) and DIPEA (2.2 mL, 12.5 mmol) were dissolved in 15 mL of anhydrous DMF. The mixture was stirred at r.t. for 24 h. Water was then added and the mixture was extracted with EtOAc. The combined organic layers was washed with H_2O and brine, dried over anhydrous Na_2SO_4, filtered, and concentrated in vacuo. The residue was purified by silica gel flash chromatography (EtOAc/Hex: 1/10) to give the desired product ***d*-1m** (78% D).

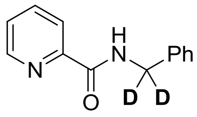

***d*-1m**

***N*-Benzylpicolinamide (*d*-1m)**: **^{1}H NMR** (400 MHz, $CDCl_3$) δ 8.53 (d, *J* = 4.6 Hz, 1H), 8.39 (s, 1H), 8.24 (d, *J* = 7.8 Hz, 1H), 7.86 (t, *J* = 8.3 Hz, 1H), 7.46 – 7.24 (m, 6H), 4.68 (d, *J* = 6.2 Hz, 0.45H); **^{13}C NMR** (100 MHz, $CDCl_3$) δ 164.3, 149.9, 148.1, 138.2, 137.3, 128.7, 127.9, 127.5, 126.2, 122.4, 43.5. **IR** (KBr): 1735, 1519, 1461, 1291, 742, 695 cm^{-1}.

III. Experimental procedure for the optimization study

1. Table S-1 .The effect of transition metal catalysts on the Csp^3-H bond carbenoid insertion[a]

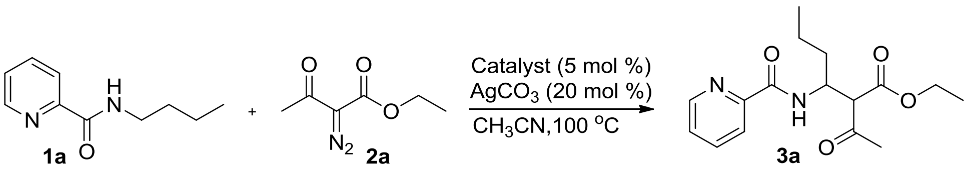

entry	catalyst	yield(%)[b]
1	$[Cp^*IrCl_2]_2$	0
2	$Cp^*Co(CO)I_2$	0
3	$Cp^*Co(MeCN)_3SbF_6$	0
4	$RhCl_3$	0
5	$Rh_2(OAc)_4$	0
6	$[Cp^*RhCl_2]_2$	47

[a]Unless otherwise noted, all the reactions were carried out using

N-butyl-pyridine-2-carboxylic acid amide (**1a**) (0.10 mmol) and diazo compound (**2a**) (0.20 mmol) with metal catalysts (5.0 mol %) in the presence of $AgCO_3$ (20 mol %) in CH_3CN (1.0 mL) at 100 °C for 24 h under Ar in a sealed reaction tube, followed by flash chromatography on SiO_2. [b] Isolated yield.

2. Table S-2. The effect of Ag salts on the Rh(III)-catalyzed Csp^3-H bond carbenoid insertion[a]

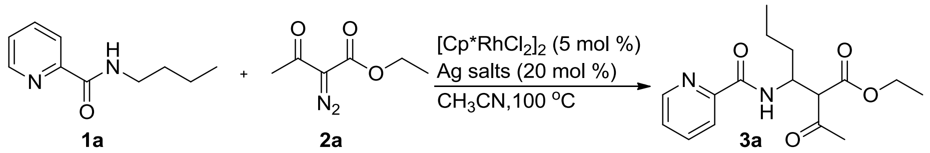

entry	Ag salts (20 mol %)	yield(%)[b]
1	$AgClO_4$	0
2	$AgSbF_6$	28
3	$AgBF_4$	15
4	$AgNTf_2$	63
5	Ag_2CO_3	47
6	AgOAc	65

[a]Unless otherwise noted, all the reactions were carried out using *N*-butyl-pyridine-2-carboxylic acid amide (**1a**) (0.10 mmol) and diazo compound (**2a**) (0.20 mmol) with $[Cp^*RhCl_2]_2$ (5 mol %) in the presence of Ag salts (20 mol %) in CH_3CN (1.0 mL) at 100 °C for 24 h under Ar in a sealed reaction tube, followed by flash chromatography on SiO_2. [b]Isolated yield.

3. Table S-3. The effect of solvents on the Rh(III)-catalyzed Csp^3-H bond carbenoid insertion[a]

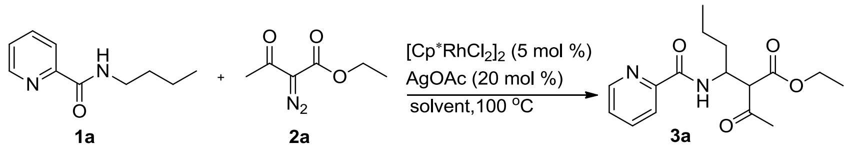

entry	solvent	yield(%)[b]
1	DMF	trace
2	DMSO	0
3	1,4-dioxane	26
4	TFE	89

[a]Unless otherwise noted, all the reactions were carried out using *N*-butyl-pyridine-2-carboxylic acid amide (**1a**) (0.10 mmol) and diazo compound (**2a**) (0.20 mmol) with $[Cp^*RhCl_2]_2$ (5 mol %) in the presence of AgOAc (20 mol %) in solvents (1.0 mL) at 100 °C for 24 h under Ar in a sealed reaction tube, followed by flash chromatography on SiO_2. [b] Isolated yield.

4. Table S-4. The effect of temperature on the the Rh(III)-catalyzed Csp^3-H bond carbenoid insertion[a]

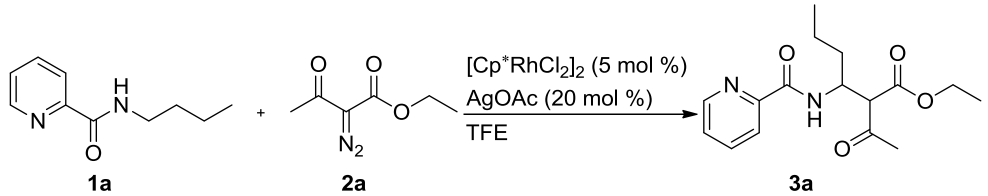

entry	temperature	yield(%)[b]
1	80 °C	63
2	100 °C	89
3	110 °C	71

[a]Unless otherwise noted, all the reactions were carried out using *N*-butyl-pyridine-2-carboxylic acid amide (**1a**) (0.10 mmol) and diazo compound (**2a**) (0.20 mmol) with $[Cp^*RhCl_2]_2$ (5 mol %) in the presence of AgOAc (20 mol %) in TFE (1.0 mL) at deferent reaction temperature for 24 h under Ar in a sealed reaction tube, followed by flash chromatography on SiO_2. [b] Isolated yield.

IV. Experimental procedure for the Rh(III)-catalyzed *N*-methylene Csp^3-H bond carbenoid insertion

1. Procedure for the Rh(III)-catalyzed *N*-methylene C-H bond carbenoid insertion of picolinamides with diazo compounds.

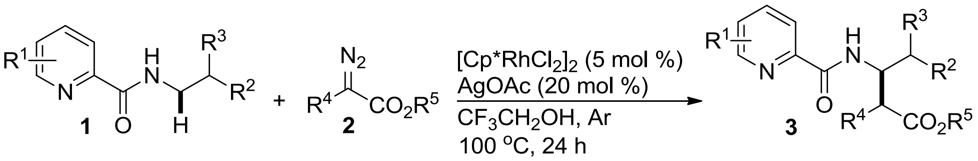

All of the products (**3a** ~ **3-1f**) were obtained according to the following procedure. To a screw capped vial with a spinvane triangular-shaped Teflon stir bar were added picolinamide (0.20 mmol), diazo compounds (0.4 mmol), $[Cp^*RhCl_2]_2$ (7.0 mg, 0.005 mmol, 5 mol %), AgOAc (6 mg, 0.02 mmol, 20 mol %) and 2,2,2-trifluoroethanol (2.0 mL) under Ar atmosphere conditions. The reaction mixture was stirred at 100 °C for 24 h, filtered through a pad of celite and then washed with ethyl acetate (3 ×10 mL). Organic solvents were removed under reduced pressure and the residue was purified by chromatography on silica gel with acetone/petroleum as the eluent to give the desired products.

2. Synthetic application of this transformation

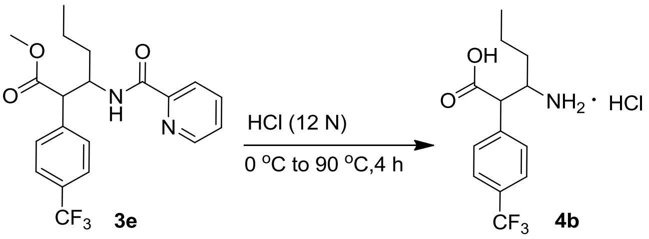

3-Amino-2-(4-(trifluoromethyl)phenyl)hexanoic acid hydrochloride. The product **3e** (78.8 mg, 0.2 mmol) was added to a 25 mL jacketed reactor containing water (0.5 mL) to give a slurry. Hydrochloric acid (12 N, 0.5 mL, 0.34 mmol) was added dropwise at 0 °C. The

reaction mixture was heated at 90 °C for 4 h. The cooled solution was concentrated *in vacuo* to give crude product. The residue was purified by chromatography on silica gel with dichloromethane/ methanol as the eluent to give the desired product.

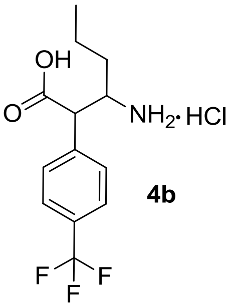

4b

3-Amino-2-(4-(trifluoromethyl)phenyl)hexanoic acid hydrochloride (4b). **^{1}H NMR** (400 MHz, DMSO/CF_3CO_2H, 20:1) δ 9.83 (s, 1H), 7.81 (q, *J* = 8.4 Hz, 4H), 5.28 (s, 1H), 2.92 – 2.82 (m, 1H), 2.75 – 2.64 (m, 1H), 1.68 – 1.57 (m, 2H), 1.31 – 1.17 (m, 2H), 0.82 (t, *J* = 7.3 Hz, 3H); **^{13}C NMR** (100 MHz, DMSO/CF_3CO_2H, 20:1) δ 169.0, 136.2, 130.2, 126.4, 117.3, 114.5, 62.2, 46.0, 27.7, 19.6, 13.6; **^{19}F NMR** (376 MHz, MeOD) δ -66.0. **HR-MS (ESI)** calcd for $[M + 1 - HCl]^+$: $C_{13}H_{17}F_3NO_2$: 276.1179, found: 276.1182; **IR** (KBr): 1683, 1328, 1192， 1017， 823, 764， 623 cm^{-1}.

3. Spectroscopic data of all the isolated products

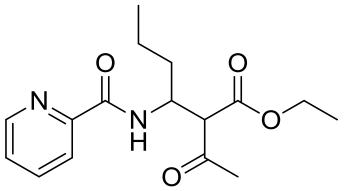

3a

Ethyl 2-acetyl-3-(picolinamido)hexanoate (3a): (8:1 crude dr), yellow oil, 89% yield. **^{1}H NMR** (400 MHz, $CDCl_3$) δ 12.26 (s, 1H), 8.43 (d, *J* = 4.7 Hz, 1H), 7.67 (t, *J* = 7.7 Hz, 1H), 7.54 (d, *J* = 7.8 Hz, 1H), 7.25 – 7.17 (m, 1H), 4.40 – 4.18 (m, 2H), 3.73 – 3.30 (m, 2H), 1.95 (s, 3H), 1.69 – 1.53 (m, 2H), 1.46 – 1.34 (m, 2H), 1.33 – 1.24 (m, 3H), 0.96 (t, *J* = 7.3 Hz, 3H); **^{13}C NMR** (100 MHz, $CDCl_3$) δ 174.5, 170.8, 169.6, 154.7, 148.1, 136.2, 124.1, 122.5, 108.6, 61.0, 50.0, 29.4, 20.5, 18.5, 14.2, 13.9. **HR-MS (ESI)** calcd for $[M + 1]^+$: $C_{16}H_{23}N_2O_4$: 307.1652, found: 307.1655; **IR** (KBr): 3476, 2958, 2928, 2871, 1738, 1652, 1587, 1566, 1376, 855, 748 cm^{-1}.

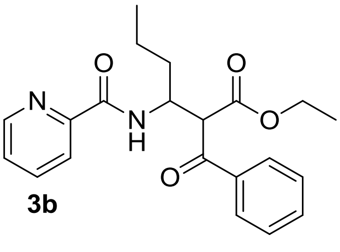

3b

Ethyl 2-benzoyl-3-(picolinamido)hexanoate (3b): (12:1 crude dr), yellow oil, 43% yield. **^{1}H NMR** (400 MHz, $CDCl_3$) δ 12.81 (s, 1H), 8.58 – 8.41 (m, 1H), 8.05 (t, *J* = 8.9 Hz, 1H), 7.79 – 7.71 (m, 1H), 7.69 – 7.56 (m, 2H), 7.54 – 7.30 (m, 3H), 7.25 – 7.18 (m, 1H), 4.42 – 4.04 (m, 2H), 3.92 – 3.80 (m, 1H), 2.91 – 2.79 (m, 1H), 1.68 – 1.53 (m, 1H), 1.51 – 1.40 (m, 1H), 1.34 – 1.27 (m, 1H), 1.23 – 1.15 (m, 3H), 1.01 – 0.91 (m, 1H), 0.85 (t, *J* = 7.3 Hz, 2H), 0.60 (t, *J* = 7.3 Hz, 1H); **^{13}C NMR** (100 MHz, $CDCl_3$) δ 171.9, 169.0, 168.3, 154.3, 148.4, 147.6, 136.2, 134.1, 133.3, 130.6, 129.0, 128.7, 128.3, 128.2, 124.3, 123.9, 109.5, 62.5, 61.2, 50.9, 31.8, 29.2, 20.5, 19.8, 13.9, 13.3. **HR-MS (ESI)** calcd for $[M + 1]^+$: $C_{21}H_{25}N_2O_4$: 369.1809, found: 369.1806; **IR** (KBr): 3626, 2959, 2931, 2871, 1747, 1700, 1643, 1597, 1567, 1447, 1262, 1181, 1138 cm^{-1}.

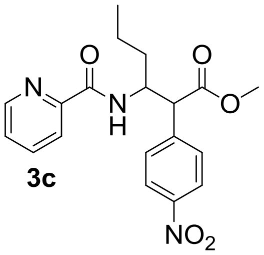

3c

Methyl 2-(4-nitrophenyl)-3-(picolinamido)hexanoate (3c)： (3:1 crude dr), yellow oil, 83% yield. **^{1}H NMR** (400 MHz, $CDCl_3$) δ 8.57 – 8.45 (m, 1H), 8.16 (d, *J* = 8.4 Hz, 2H), 7.84 – 7.71 (m, 1H), 7.61 (t, *J* = 8.4 Hz, 3H), 7.36 – 7.27 (m, 1H), 5.52 (s, 1H), 3.75 (s, 3H), 3.62 – 3.44 (m, 1H), 3.41 – 3.28 (m, 1H), 1.33 – 1.13 (m, 2H), 1.09 – 0.91 (m, 2H), 0.69 – 0.55 (m, 3H); **^{13}C NMR** (100 MHz, $CDCl_3$) δ 169.4,

169.2, 153.5, 148.3, 147.8, 142.2, 137.1, 130.3, 124.9, 123.9, 123.6, 63.0, 52.8, 49.3, 31.3, 19.7, 13.4. **HR-MS (ESI)** calcd for $[M + 1]^+$: $C_{19}H_{22}N_3O_5$: 372.1554, found: 372.1559; **IR** (KBr): 3430, 2955, 2868, 1746, 1639, 1523, 1420, 1345, 1209, 845, 743, 622 cm^{-1}.

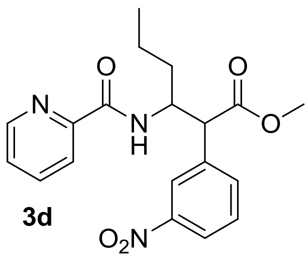

3d

Methyl 2-(3-nitrophenyl)-3-(picolinamido)hexanoate (3d): (2.5:1 crude dr), yellow oil, 65% yield. **^{1}H NMR** (400 MHz, $CDCl_3$) δ 8.61 (s, 1H), 8.36 (d, *J* = 23.0 Hz, 1H), 8.23 (d, *J* = 7.6 Hz, 1H), 7.93 – 7.80 (m, 2H), 7.72 (d, *J* = 7.7 Hz, 1H), 7.59 (t, *J* = 7.8 Hz, 1H), 7.44 – 7.36 (m, 1H), 5.60 (s, 1H), 3.84 (s, 3H), 3.70 – 3.58 (m, 1H), 3.48 – 3.37 (m, 1H), 1.42 – 1.24 (m, 2H), 1.19 – 1.00 (m, 2H), 0.79 – 0.65 (m, 3H); **^{13}C NMR** (100 MHz, $CDCl_3$) δ 169.4, 169.2, 153.6, 148.3, 137.1, 135.5, 129.6, 124.9, 124.3, 124.0, 123.3, 63.0, 52.9, 49.3, 31.3, 19.7, 13.4. **HR-MS (ESI)** calcd for $[M + 1]^+$: $C_{19}H_{22}N_3O_5$: 372.1554, found: 372.1561; **IR** (KBr): 3698, 2955, 1736, 1638, 1578, 1425, 1347, 1211, 811, 738, 684 cm^{-1}.

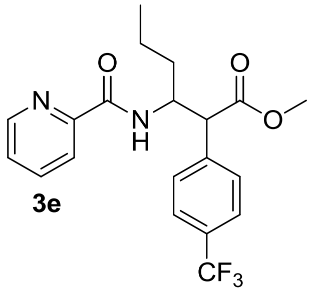

3e

Methyl3-(picolinamido)-2-(4-(trifluoromethyl)phenyl)hexanoate (3e): (2:1 crude dr), yellow oil, 91% yield. **^{1}H NMR** (400 MHz, $CDCl_3$) δ 8.65 – 8.53 (m, 1H), 7.87 – 7.78 (m, 1H), 7.72 – 7.62 (m, 3H), 7.61 – 7.53 (m, 2H), 7.41 – 7.33 (m, 1H), 5.77 (s, 1H), 3.82 (s, 3H), 3.56 – 3.45 (m, 1H), 3.43 – 3.31 (m, 1H), 1.53 – 1.40 (m, 1H), 1.20 – 1.06 (m, 1H), 1.03 – 0.85 (m, 2H), 0.77 – 0.58 (m, 3H); **^{13}C NMR** (100 MHz, $CDCl_3$) δ 169.8, 169.5, 153.9, 148.3, 147.9, 138.7, 137.4, 137.0, 129.9, 129.6, 125.5, 124.7, 123.8, 64.1, 62.7, 52.6, 48.4, 45.8, 31.4, 29.9, 20.2, 19.6, 13.5, 13.3. **HR-MS (ESI)** calcd for $[M + 1]^+$: $C_{20}H_{22}F_3N_2O_3$: 395.1577, found: 395.1549; **IR** (KBr): 3490, 2956, 1746, 1637, 1412, 1323, 1167, 1118, 750 cm^{-1}.

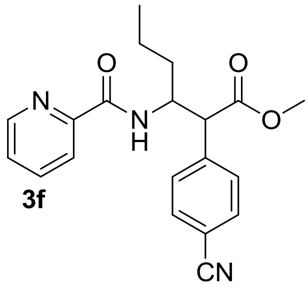

3f

Methyl 2-(4-cyanophenyl)-3-(picolinamido)hexanoate (3f): (2.5:1 crude dr), yellow oil, 82% yield. **^{1}H NMR** (400 MHz, $CDCl_3$) δ 8.56 (d, *J* = 10.4 Hz, 1H), 7.88 – 7.75 (m, 1H), 7.66 (d, *J* = 7.4 Hz, 3H), 7.57 (d, *J* = 7.6 Hz, 2H), 7.40 – 7.31 (m, 1H), 5.57 (s, 1H), 3.78 (s, 3H), 3.68 – 3.48 (m, 1H), 3.43 – 3.30 (m, 1H), 1.58 – 1.42 (m, 1H), 1.34 – 1.17 (m, 1H), 1.11 – 0.94 (m, 2H), 0.75 – 0.60 (m, 3H); **^{13}C NMR** (100 MHz, $CDCl_3$) δ 169.4, 169.3, 153.6, 153.5, 148.3, 147.8, 140.4, 140.2, 137.5, 137.1, 132.3, 132.3, 130.1, 130.0, 124.9, 123.9, 118.5, 118.3, 112.4, 112.2, 64.0, 63.1, 52.8, 52.7, 49.2, 46.0, 31.3, 29.9, 20.2, 19.6, 13.6, 13.4. **HR-MS (ESI)** calcd for $[M + 1]^+$: $C_{20}H_{22}N_3O_3$: 352.1656, found: 352.1659; **IR** (KBr): 3697, 2955, 1746, 1644, 1463, 1022, 675 cm^{-1}.

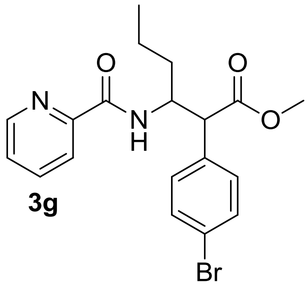

3g

Methyl 2-(4-bromophenyl)-3-(picolinamido)hexanoate (3g): (2:1 crude dr), yellow oil, 50% yield. **^{1}H NMR** (400 MHz, $CDCl_3$) δ 8.50 (s, 1H), 7.78 – 7.66 (m, 1H), 7.58 (d, *J* = 7.5 Hz, 1H), 7.44 (d, *J* = 7.8 Hz, 2H), 7.25 (d, *J* = 8.3 Hz, 3H), 5.68 (s, 1H), 3.70 (d, *J* = 12.3 Hz, 3H), 3.44 – 3.32 (m, 1H), 3.32 – 3.19 (m, 1H), 1.59

– 1.44 (m, 1H), 1.35 (dd, *J* = 19.3, 9.7 Hz, 1H), 1.03 (dt, *J* = 18.9, 9.9 Hz, 1H), 0.93 – 0.76 (m, 1H), 0.67 (t, *J* = 6.5 Hz, 1H), 0.53 (t, *J* = 7.2 Hz, 2H); **^{13}C NMR** (100 MHz, $CDCl_3$) δ 170.1, 169.6, 154.0, 148.3, 147.9, 137.4, 136.9, 133.6, 131.8, 131.3, 130.9, 124.8, 124.6, 123.8, 122.8, 64.0, 62.3, 52.6, 52.4, 47.8, 45.6, 31.5, 29.9, 20.2, 19.7, 13.6, 13.3. **HR-MS (ESI)** calcd for $[M + 1]^+$: $C_{19}H_{22}BrN_2O_3$: 405.0808, found: 405.0811; **IR** (KBr): 3495, 2951, 1741, 1634, 1405, 1171, 1102, 741 cm^{-1}.

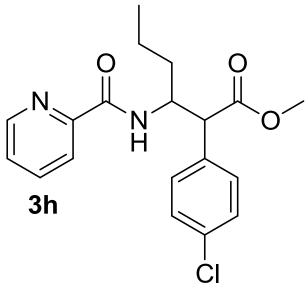

3h

Methyl 2-(4-chlorophenyl)-3-(picolinamido)hexanoate (3h): (1.7:1 crude dr), yellow oil, 62% yield. **^{1}H NMR** (400 MHz, $CDCl_3$) δ 8.67 – 8.56 (m, 1H), 7.88 – 7.78 (m, 1H), 7.68 (d, *J* = 7.7 Hz, 1H), 7.46 – 7.34 (m, 5H), 5.81 (s, 1H), 3.88 – 3.76 (m, 3H), 3.51 – 3.41 (m, 1H), 3.40 – 3.31 (m, 1H), 1.69 – 1.54 (m, 1H), 1.51 – 1.38 (m, 1H), 1.18 – 1.06 (m, 1H), 1.02 – 0.86 (m, 1H), 0.76 (t, *J* = 6.9 Hz, 1H), 0.62 (t, *J* = 7.2 Hz, 2H); **^{13}C NMR** (100 MHz, $CDCl_3$) δ 170.2, 169.6, 154.1, 148.3, 147.9, 137.4, 136.9, 134.6, 133.0, 131.0, 130.6, 128.9, 124.6, 123.8, 64.0, 62.2, 52.5, 52.4, 47.8, 45.6, 31.5, 29.9, 20.3, 19.7, 13.6, 13.3. **HR-MS (ESI)** calcd for $[M + 1]^+$: $C_{19}H_{22}ClN_2O_3$: 361.1313, found: 361.1315; **IR** (KBr): 2956, 1745, 1637, 1410, 1316, 1177, 1099, 1006 cm^{-1}.

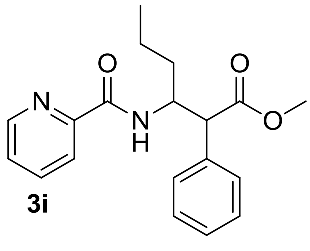

3i

Methyl 2-phenyl-3-(picolinamido)hexanoate (3i): (1.7:1 crude dr), yellow oil, 51% yield. **^{1}H NMR** (400 MHz, $CDCl_3$) δ 8.61 (d, *J* = 4.7 Hz, 1H), 7.87 – 7.77 (m, 1H), 7.68 (d, *J* = 7.7 Hz, 1H), 7.48 – 7.32 (m, 6H), 6.03 (s, 1H), 3.85 – 3.78 (m, 3H), 3.44 – 3.37 (m, 1H), 3.36 – 3.29 (m, 1H), 1.92 – 1.76 (m, 1H), 1.66 – 1.51 (m, 1H), 1.17 – 1.01 (m, 1H), 0.99 – 0.90 (m, 1H), 0.72 (t, *J* = 7.1 Hz, 1H), 0.56 (t, *J* = 7.2 Hz, 2H); **^{13}C NMR** (100 MHz, $CDCl_3$) δ 170.8, 169.8, 154.4, 148.3, 148.0, 137.3, 136.8, 134.2, 129.7, 129.2, 128.7, 128.7, 124.7, 124.5, 123.7, 64.8, 62.5, 52.4, 52.3, 47.1, 45.5, 31.6, 29.9, 20.3, 19.7, 13.6, 13.2. **HR-MS (ESI)** calcd for [M + H]: $C_{19}H_{23}N_2O_3$: 327.1703, found: 327.1706; **IR** (KBr): 2946, 1744, 1633, 1578, 1409, 1104, 742 cm^{-1}.

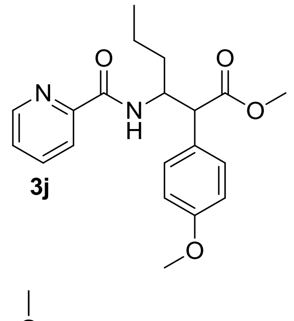

3j

Methyl 2-(4-methoxyphenyl)-3-(picolinamido)hexanoate (3j): 0% yield.

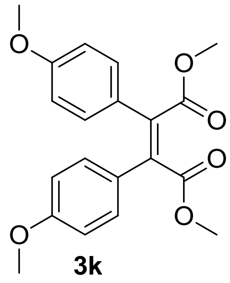

3k

Dimethyl 2,3-bis(4-methoxyphenyl)maleate (3k)[6]: ^{1}H NMR (400 MHz, $CDCl_3$) δ 6.95 (d, *J* = 8.9 Hz, 4H), 6.65 (d, *J* = 8.9 Hz, 4H), 3.74 (s, 6H), 3.68 (s, 6H).

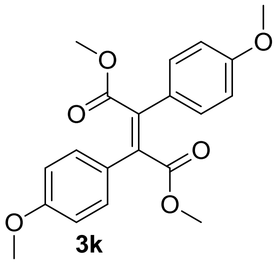

3k

Dimethyl 2,3-bis(4-methoxyphenyl)fumarate (3k)[6]**:** ^{1}**H NMR** (400 MHz, $CDCl_3$) δ 7.31 (d, *J* = 8.8 Hz, 4H), 6.89 (d, *J* = 8.8 Hz, 4H), 3.82 (s, 6H), 3.58 (s, 6H).

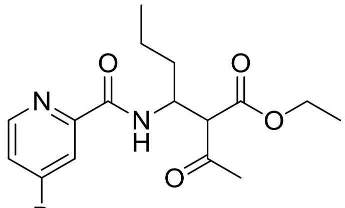

3l

Ethyl 2-acetyl-3-(4-bromopicolinamido)hexanoate (3l): (12:1 crude dr), yellow oil, 48% yield. ^{1}**H NMR** (400 MHz, $CDCl_3$) δ 12.22 (s, 1H), 8.18 (d, *J* = 5.2 Hz, 1H), 7.65 (s, 1H), 7.33 (d, *J* = 5.2 Hz, 1H), 4.24 – 4.09 (m, 2H), 3.58 – 3.45 (m, 2H), 1.90 (s, 3H), 1.58 – 1.48 (m, 2H), 1.37 – 1.28 (m, 2H), 1.23 (t, *J* = 7.1 Hz, 3H), 0.89 (t, *J* = 7.3 Hz, 3H); ^{13}**C NMR** (100 MHz, $CDCl_3$) δ 175.0, 170.6, 168.3, 155.8, 148.9, 132.9, 127.4, 126.1, 108.2, 61.1, 49.7, 29.4, 20.5, 18.6, 14.3, 13.9. **HR-MS (ESI)** calcd for $[M + 1]^+$: $C_{16}H_{22}BrN_2O_4$: 384.0752, found: 384.0755; **IR** (KBr): 3441, 2980, 1741, 1682, 1630, 1570, 1490, 1329, 1013, 660, 587 cm^{-1}.

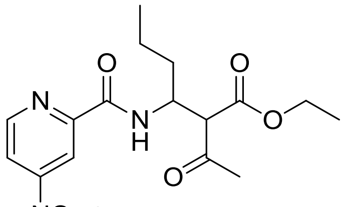

3m

Ethyl 2-acetyl-3-(4-nitropicolinamido)hexanoate (3m): 0% yield.

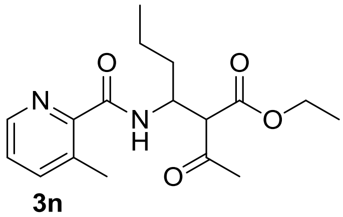

3n

Ethyl 2-acetyl-3-(3-methylpicolinamido)hexanoate (3n): (2:1 crude dr), yellow oil, 48% yield. ^{1}**H NMR** (400 MHz, $CDCl_3$) δ 12.26 (s, 1H), 8.16 (d, *J* = 4.4 Hz, 1H), 7.38 (d, *J* = 7.7 Hz, 1H), 7.04 – 6.99 (m, 1H), 4.27 – 4.15 (m, 1H), 4.09 – 3.99 (m, 1H), 3.64 – 3.55 (m, 1H), 3.55 – 3.48 (m, 1H), 2.31 (s, 1H), 2.28 (s, 2H), 2.08 (s, 1H), 2.03 (s, 2H), 1.60 – 1.51 (m, 1H), 1.39 – 1.30 (m, 1H), 1.27 – 1.22 (m, 2H), 1.21 – 1.18 (m, 1H), 1.06 – 0.95 (m, 1H), 0.89 (t, *J* = 7.3 Hz, 2H), 0.84 – 0.74 (m, 1H), 0.65 (t, *J* = 7.2 Hz, 1H); ^{13}**C NMR** (100 MHz, $CDCl_3$) δ 176.6, 176.1, 170.8, 170.0, 169.5, 154.5, 153.6, 146.5, 145.5, 138.3, 138.2, 130.8, 130.4, 123.7, 123.4, 107.5, 104.2, 61.0, 51.1, 49.0, 30.1, 29.7, 20.5, 19.8, 19.2, 18.2, 17.5, 14.2, 14.1, 14.0, 13.5. **HR-MS (ESI)** calcd for $[M + 1]^+$: $C_{17}H_{25}N_2O_4$: 321.1795, found: 321.1779; **IR** (KBr): 3439, 2960, 2932, 2872, 1735, 1655, 1574, 1450, 1338, 1211, 799, 742 cm^{-1}.

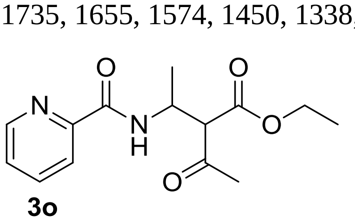

3o

Ethyl 2-acetyl-3-(picolinamido)butanoate (3o): (10:1 crude dr), yellow oil, 83% yield. ^{1}**H NMR** (400 MHz, $CDCl_3$) δ 12.27 (s, 1H), 8.46 (d, *J* = 4.7 Hz, 1H), 7.73 – 7.66 (m, 1H), 7.56 (d, *J* = 7.8 Hz, 1H), 7.27 – 7.21 (m, 1H), 4.30 – 4.21 (m, 2H), 3.85 – 3.74 (m, 1H), 3.71 – 3.61 (m, 1H), 1.97 (s, 3H), 1.36 – 1.29 (m, 3H), 1.25 (t, *J* = 7.3 Hz, 3H); ^{13}**C NMR** (100 MHz, $CDCl_3$) δ 174.9, 171.2, 169.8, 155.1, 148. 5, 136.6, 124.4, 122.8, 108.5, 61.3, 44.5, 18.9, 14.5, 12.8; **HR-MS (ESI)** calcd for $[M + 1]^+$: $C_{14}H_{19}N_2O_4$:

279.1339, found: 279.1346; **IR** (KBr): 2981, 2936, 1737, 1651, 1469, 1396, 1227, 749 cm^{-1}.

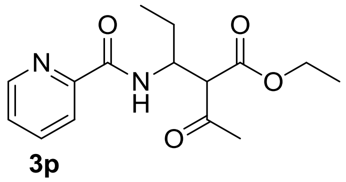

3p

Ethyl 2-acetyl-3-(picolinamido)pentanoate (3p): (10:1 crude dr), yellow oil, 86% yield. **^{1}H NMR** (400 MHz, $CDCl_3$) δ 12.27 (s, 1H), 8.45 (d, *J* = 4.5 Hz, 1H), 7.68 (t, *J* = 7.7 Hz, 1H), 7.55 (d, *J* = 7.8 Hz, 1H), 7.26 – 7.21 (m, 1H), 4.25 (q, *J* = 7.1 Hz, 2H), 3.68 – 3.59 (m, 1H), 3.58 – 3.49 (m, 1H), 1.97 (s, 3H), 1.74 – 1.63 (m, 2H), 1.35 – 1.27 (m, 3H), 0.99 (t, *J* = 7.4 Hz, 3H); **^{13}C NMR** (100 MHz, $CDCl_3$) δ 174.5, 170.8, 169.6, 154.8, 148.1, 136.2, 124.1, 122.5, 108.7, 61.0, 51.4, 20.6, 18.5, 14.2, 11.6. **HR-MS (ESI)** calcd for $[M + 1]^+$: $C_{15}H_{21}N_2O_4$: 293.1536, found: 293.1502; **IR** (KBr): 3499, 2972, 2934, 2875, 1738, 1651, 1587, 1567, 1339, 1285, 748 cm^{-1}.

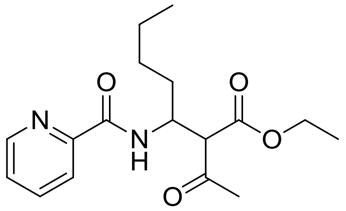

3q

Ethyl 2-acetyl-3-(picolinamido)heptanoate (3q): (10:1 crude dr), yellow oil, 82% yield. **^{1}H NMR** (400 MHz, $CDCl_3$) δ 12.26 (s, 1H), 8.43 (d, J = 5.5 Hz, 1H), 7.67 (m, 1H), 7.53 (d, *J* = 7.7 Hz, 1H), 7.21 (dd, *J* = 7.5, 4.8 Hz, 1H), 4.27 – 4.19 (m, 2H), 3.70 – 3.61 (m, 1H), 3.59 – 3.48 (m, 1H), 1.94 (s, 3H), 1.69 – 1.59 (m, 2H), 1.40 – 1.33 (m, 4H), 1.31 – 1.24 (m, 3H), 0.91 (t, *J* = 6.7 Hz, 3H); **^{13}C NMR** (100 MHz, $CDCl_3$) δ 174.5, 170.8, 169.5, 154.7, 148.1, 136.2, 124.1, 122.5, 108.7, 61.0, 49.8, 29.4, 27.0, 22.5, 18.5, 14.2, 14.0. **HR-MS (ESI)** calcd for $[M + 1]^+$: $C_{17}H_{25}N_2O_4$: 321.1809, found: 321.1813; **IR** (KBr): 2957, 2932, 2869, 1738, 1652, 1587, 1567, 1395, 1376, 855 cm^{-1}.

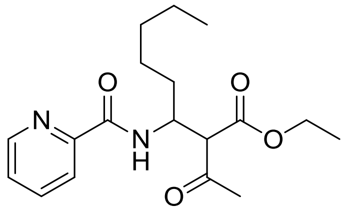

3r

Ethyl 2-acetyl-3-(picolinamido)octanoate (3r): (10:1 crude dr), yellow oil, 78% yield. **^{1}H NMR** (400 MHz, $CDCl_3$) δ 12.18 (s, 1H), 8.35 (d, *J* = 4.4 Hz, 1H), 7.60 (dd, *J* = 10.8, 4.6 Hz, 1H), 7.46 (d, *J* = 7.8 Hz, 1H), 7.17 – 7.11 (m, 1H), 4.16 (q, *J* = 7.1 Hz, 2H), 3.63 – 3.53 (m, 1H), 3.46 (dt, *J* = 13.2, 7.7 Hz, 1H), 1.87 (s, 3H), 1.61 – 1.51 (m, 2H), 1.34 – 1.18 (m, 9H), 0.82 (t, *J* = 6.4 Hz, 3H); **^{13}C NMR** (100 MHz, **$CDCl_3$**) δ 174.5, 170.8, 169.5, 154.7, 148.1, 136.2, 124.1, 122.5, 108.7, 61.0, 49.8, 31.7, 27.3, 26.9, 22.6, 18.6, 14.2, 14.1. **HR-MS (ESI)** calcd for $[M + 1]^+$: $C_{18}H_{27}N_2O_4$: 335.1965, found: 335.1985; **IR** (KBr): 3440, 2955, 2931, 2858, 1738, 1653, 1587, 1440, 1396, 1377, 1173, 787 cm^{-1}.

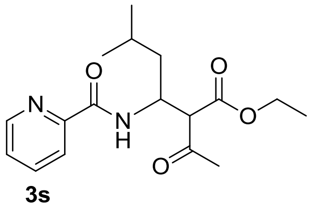

3s

Ethyl 2-acetyl-5-methyl-3-(picolinamido)hexanoate (3s): (9:1 crude dr), yellow oil, 76% yield. **^{1}H NMR** (400 MHz, $CDCl_3$) δ 12.18 (s, 1H), 8.38 – 8.32 (m, 1H), 7.60 (td, *J* = 7.7, 1.7 Hz, 1H), 7.47 (d, *J* = 7.8 Hz, 1H), 7.17 – 7.11 (m, 1H), 4.21 – 4.10 (m, 2H), 3.68 – 3.58 (m, 1H), 3.54 – 3.43 (m, 1H), 1.87 (s, 3H), 1.64 – 1.53 (m, 1H), 1.50 – 1.43 (m, 2H), 1.22 (t, *J* = 7.1 Hz, 3H), 0.89 (d, *J* = 1.3 Hz, 3H), 0.88 (d, *J* = 1.3 Hz, 3H); **^{13}C NMR** (100 MHz, $CDCl_3$) δ 174.5, 170.8, 169.5, 154.7, 148.0, 136.2, 124.1, 122.5, 108.7, 61.0, 48.3, 36.0, 26.5, 22.6, 18.6, 14.2. **HR-MS (ESI)** calcd for $[M + 1]^+$: $C_{17}H_{25}N_2O_4$: 321.1809, found: 321.1812; **IR** (KBr): 3440, 2981, 2954, 2870, 1739, 1651, 1587, 1566, 1524, 1437, 1380, 1339, 748 cm^{-1}.

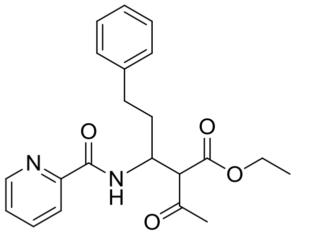

3t

Ethyl 2-acetyl-5-phenyl-3-(picolinamido)pentanoate (3t)： (9:1 crude dr), yellow oil, 83% yield. **^{1}H NMR** (400 MHz, $CDCl_3$) δ 12.28 (s, 1H), 8.45 (d, *J* = 4.7 Hz, 1H), 7.69 (td, *J* = 7.7, 1.7 Hz, 1H), 7.57 (d, *J* = 7.8 Hz, 1H), 7.31 (t, *J* = 6.7 Hz, 2H), 7.27 – 7.19 (m, 4H), 4.23 (q, *J* = 7.1 Hz, 2H), 3.80 – 3.70 (m, 1H), 3.70 – 3.57 (m, 1H), 2.73 (dd, *J* = 8.7, 7.0 Hz, 2H), 2.08 – 1.98 (m, 2H), 1.95 (s, 3H), 1.27 (t, *J* = 7.1 Hz, 3H); **^{13}C NMR** (100 MHz, $CDCl_3$) δ 174.5, 170.8, 169.6, 154.6, 148.1, 141.6, 136.3, 128.4, 128.3, 125.9, 124.2, 122.6, 108.7, 61.1, 49.5, 33.5, 28.8, 18.6, 14.2. **HR-MS (ESI)** calcd for $[M + 1]^+$: $C_{21}H_{25}N_2O_4$: 369.1180, found: 369.1811; **IR** (KBr): 3397, 2981, 2930, 2856, 1736, 1650, 1587, 1567, 1495, 1395, 1338, 808, 748 cm^{-1}.

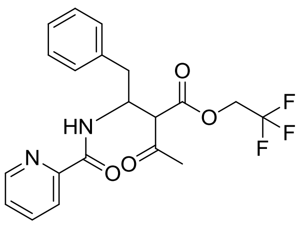

3u

2,2,2-trifluoroethyl2-acetyl-4-phenyl-3-(picolinamido)butanoate (3u): (10:1 crude dr), yellow oil, 73% yield. **^{1}H NMR** (400 MHz, $CDCl_3$) δ 11.67 (s, 1H), 8.34 (d, *J* = 4.7 Hz, 1H), 7.63 (dd, *J* = 12.5, 5.8 Hz, 2H), 7.25 – 7.14 (m, 6H), 4.52 (dq, *J* = 12.6, 8.4 Hz, 1H), 4.45 – 4.34 (m, 1H), 3.92 – 3.80 (m, 1H), 3.75 – 3.65 (m, 1H), 3.02 – 2.87 (m, 2H), 1.87 (s, 3H); **^{13}C NMR** (100 MHz, $CDCl_3$) δ 176.2, 169.1, 169.0, 153.8, 147.9, 138.9, 136.6, 128.8, 128.6, 126.4, 124.6, 123.4, 108.4, 60.7(q, *J* = 36.9 Hz), 51.9, 33.5, 18.6; **^{19}F NMR** (376 MHz, $CDCl_3$) δ -73.4. **HR-MS (ESI)** calcd for $[M + 1]^+$: $C_{20}H_{20}F_3N_2O_4$:409.1370, found: 409.1344; **IR** (KBr): 3437, 2952, 2923, 2851, 1734, 1709, 1637, 1587, 1566, 1525, 1167, 810, 701 cm^{-1}.

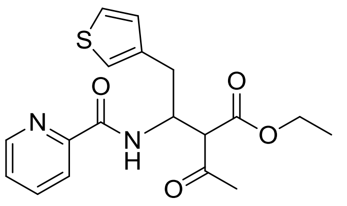

3v

Ethyl2-acetyl-3-(picolinamido)-4-(thiophen-3-yl)butanoate (3v): (10:1 crude dr), yellow oil, 66% yield. **^{1}H NMR** (400 MHz, $CDCl_3$) δ 12.27 (s, 1H), 8.44 (d, *J* = 4.4 Hz, 1H), 7.70 (t, *J* = 7.7 Hz, 1H), 7.60 (d, *J* = 7.8 Hz, 1H), 7.24 (d, *J* = 6.2 Hz, 1H), 7.15 (d, *J* = 4.9 Hz, 1H), 6.99 – 6.93 (m, 1H), 6.93 – 6.90 (m, 1H), 4.24 (q, *J* = 7.1 Hz, 2H), 3.96 – 3.89 (m, 1H), 3.88 – 3.80 (m, 1H), 3.32 – 3.13 (m, 2H), 1.92 (s, 3H), 1.29 (t, *J* = 7.1 Hz, 3H); **^{13}C NMR** (100 MHz, $CDCl_3$) δ 174.5, 170.7, 169.6, 154.2, 148.1, 141.3, 136.4, 127.0, 125.2, 124.4, 123.7, 122.8, 108.9, 61.2, 51.7, 29.7, 27.7, 18.4, 14.2. **HR-MS (ESI)** calcd for $[M + 1]^+$: $C_{18}H_{21}N_2O_4S$: 361.1217, found: 361.1226; **IR** (KBr): 3392, 2982, 2923, 2851, 1736, 1651, 1587, 1414, 1394, 852, 814 cm^{-1}.

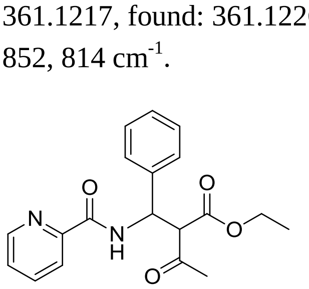

3w

Ethyl 3-oxo-2-(phenyl(picolinamido)methyl)butanoate (3w): (3:1 crude dr), yellow oil, 51% yield. **^{1}H NMR** (400 MHz, $CDCl_3$) δ 13.00 (s, 1H), 8.42 (d, *J* = 4.2 Hz, 1H), 8.14 (d, *J* = 7.8 Hz, 2H), 7.80 – 7.72 (m, 1H), 7.39 – 7.31 (m, 2H), 7.27 – 7.18 (m, 2H), 7.04 (dd, *J* = 7.2, 1.5 Hz, 1H), 4.52 (dd, *J* = 14.7, 6.3 Hz, 1H), 4.40 (dd, *J* = 14.7, 5.5 Hz, 1H), 4.07 (q, *J* = 7.1 Hz, 2H), 2.09 (s, 1H), 1.70 (s, 2H), 1.09 (t, *J* = 7.1 Hz, 2H), 1.03 (t, *J* = 7.1 Hz, 1H); **^{13}C NMR** (100 MHz, $CDCl_3$) δ 174.3, 172.2, 164.0, 149.9, 147.9, 137.8, 137.4, 134.5, 132.1, 128.9, 128.2, 127.7, 126.2, 122.3, 101.9, 60.9, 41.6, 19.8, 14.1. **HR-MS (ESI)** calcd for $[M + Na]^+$: $C_{19}H_{20}N_2O_4$ Na^+: 363.1315, found: 363.1316; **IR** (KBr): 3387, 3060, 2981, 2924, 1740, 1712, 1671, 1570, 1433, 1398, 820, 751 cm^{-1}.

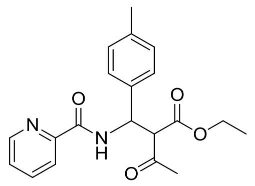

3x

Ethyl 3-oxo-2-(picolinamido(p-tolyl)methyl)butanoate(3x): (2:1 crude dr), yellow oil, 63% yield. **^{1}H NMR** (400 MHz, $CDCl_3$) δ 13.06 (s, 1H), 8.48 (d, *J* = 4.0 Hz, 1H), 8.25 – 8.18 (m, 1H), 8.18 – 8.11 (m, 1H), 7.87 – 7.80 (m, 1H), 7.43 – 7.37 (m, 1H), 7.35 – 7.28 (m, 1H), 7.12 (d, *J* = 7.9 Hz, 1H), 6.92 (s, 1H), 4.59 – 4.51 (m, 1H), 4.46 – 4.38 (m, 1H), 4.20 – 4.06 (m, 2H), 2.34 (s, 1H), 2.33 (s, 2H), 2.16 (s, 1H), 1.77 (s, 2H), 1.18 (t, *J* = 7.1 Hz, 2H), 1.09 (t, *J* = 7.1 Hz, 1H); **^{13}C NMR** (100 MHz, $CDCl_3$) δ 174.3, 172.3, 163.9, 149.9, 147.9, 137.4, 137.4, 134.7, 134.4, 133.5, 132.7, 129.0, 128.9, 126.1, 122.3, 102.0, 100.0, 60.9, 41.4, 21.0, 19.8, 14.2. **HR-MS (ESI)** calcd for $[M + 1]^+$: $C_{20}H_{23}N_2O_4$: 355.1652, found: 355.1658; **IR** (KBr): 3457, 2985, 2916, 2844, 1743, 1646, 1569, 1554, 1519, 1332, 818, 805 cm^{-1}.

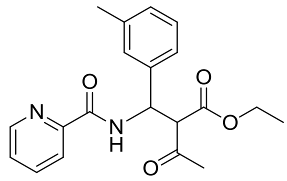

3y

Ethyl 3-oxo-2-(picolinamido(m-tolyl)methyl)butanoate (3y): (3:1 crude dr), yellow oil, 30% yield. **^{1}H NMR** (400 MHz, $CDCl_3$) δ 13.09 (s, 1H), 8.51 (d, *J* = 4.1 Hz, 1H), 8.27 – 8.16 (m, 2H), 7.90 – 7.82 (m, 1H), 7.42 (dd, *J* = 7.4, 4.7 Hz, 1H), 7.30 – 7.22 (m, 1H), 7.11 (d, *J* = 7.6 Hz, 1H), 7.01 (d, *J* = 7.7 Hz, 1H), 4.57 (dd, *J* = 14.6, 6.3 Hz, 1H), 4.44 (dd, *J* = 14.6, 5.4 Hz, 1H), 4.17 (q, *J* = 7.1 Hz, 2H), 2.36 (s, 3H), 2.17 (s, 1H), 1.79 (s, 2H), 1.19 (t, *J* = 7.1 Hz, 2H), 1.12 (t, *J* = 7.1 Hz, 1H); **^{13}C NMR** (100 MHz, $CDCl_3$) δ 201.9, 174.4, 172.4, 168.9, 163.9, 149.9, 149.6, 148.1, 147.9, 137.9, 137.5, 137.4, 131.9, 131.5, 130.0, 129.7, 128.8, 128.6, 126.3, 126.2, 122.3, 101.7, 61.5, 60.8, 41.7, 41.6, 21.2, 19.8, 14.2, 13.9. **HR-MS (ESI)** calcd for $[M + 1]^+$: $C_{20}H_{23}N_2O_4$: 355.1652, found: 355.1658; **IR** (KBr): 3389, 3057, 2983, 2921, 1743, 1714, 1675, 1569, 1465, 1434, 1332, 1150, 750 cm^{-1}.

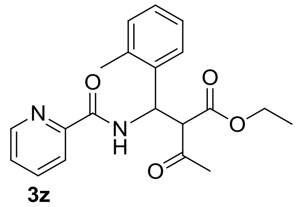

3z

Ethyl 3-oxo-2-(picolinamido(o-tolyl)methyl)butanoate (3z): (2:1 crude dr), yellow oil, 25% yield. **^{1}H NMR** (400 MHz, $CDCl_3$) δ 13.05 (s, 1H), 8.42 (t, *J* = 5.7 Hz, 1H), 8.21 (d, *J* = 7.8 Hz, 1H), 7.83 (t, *J* = 7.0 Hz, 1H), 7.42 – 7.34 (m, 1H), 7.24 – 7.16 (m, 2H), 7.16 – 7.09 (m, 1H), 6.98 (d, *J* = 7.0 Hz, 1H), 5.30 (d, *J* = 13.9 Hz, 1H), 4.54 – 4.35 (m, 1H), 4.25 – 4.04 (m, 2H), 2.41 (s, 3H), 1.77 (s, 2H), 1.42 (s, 1H), 1.18 (t, *J* = 7.1 Hz, 3H); **^{13}C NMR** (100 MHz, $CDCl_3$) δ 176.4, 174.2, 172.4, 170.6, 169.7, 163.7, 154.7, 149.8, 148.2, 147.8, 138.2, 137.8, 137.4, 136.3, 135.9, 135.4, 134.2, 132.0, 130.4, 130.3, 129.8, 128.1, 127.9, 126.1, 125.9, 124.1, 122.3, 106. 8, 102.5, 61.0, 61.0, 48.4, 38.9, 19.9, 19.8, 19.5, 17.9, 14.2, 14.1; **HR-MS (ESI)** calcd for $[M + 1]^+$: $C_{20}H_{23}N_2O_4$: 355.1652, found: 355.1656; **IR** (KBr): 3609, 3009, 2945, 2831, 1744, 1695, 1678, 1569, 1554, 1418, 1385, 1365, 1332, 745 cm^{-1}.

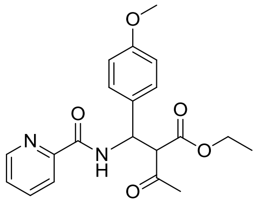

3-1a

Ethyl2-((4-methoxyphenyl)(picolinamido)methyl)-3-oxobutanoate (3-1a): (3:1 crude dr), yellow oil, 47% yield. **^{1}H NMR** (400 MHz, $CDCl_3$) δ 13.04 (s, 1H), 8.46 (d, *J* = 4.1 Hz, 1H), 8.23 – 8.17 (m, 1H), 8.12 (s, 1H), 7.82 (t, *J* = 7.5 Hz, 1H), 7.43 – 7.29 (m, 2H), 6.91 – 6.78 (m, 1H), 6.65 (d, *J* = 2.5 Hz, 1H), 4.51 (dd, J = 14.5, 6.2 Hz, 1H), 4.38 (dd, *J* = 14.4, 5.2 Hz, 1H), 4.13 (q, *J* = 7.1 Hz, 2H), 3.78 (s, 3H), 2.15 (s, 1H), 1.78 (s, 2H), 1.16 (t, *J* = 7.1 Hz, 2H), 1.08 (t, *J* = 7.1 Hz, 1H); **^{13}C NMR** (100 MHz,

$CDCl_3$) δ 174.3, 172.2, 163.9, 159.0, 149.9, 147.9, 137.4, 135.9, 130.4, 130.0, 126.1, 122.3, 117.7, 113.3, 101.9, 60.9, 55.3, 41.2, 19. 8, 14.1; **HR-MS (ESI)** calcd for $[M + 1]^+$: $C_{20}H_{23}N_2O_5$: 371.1601, found: 371.1605; **IR** (KBr): 3388, 2925, 1738, 1672, 1643, 1570, 1490, 1353, 1332, 867, 820 cm^{-1}.

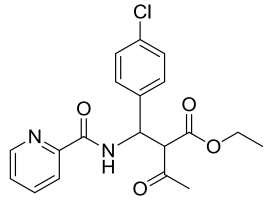

3-1b

Ethyl2-((4-chlorophenyl)(picolinamido)methyl)-3-oxobutanoate (3-1b): (4:1 crude dr), yellow oil, 45% yield. **^{1}H NMR** (400 MHz, $CDCl_3$) δ 11.72 (s, 1H), 8.41 (d, *J* = 4.4 Hz, 1H), 7.77 – 7.66 (m, 2H), 7.39 (d, *J* = 8.2 Hz, 2H), 7.31 (d, *J* = 8.3 Hz, 2H), 7.25 (d, *J* = 5.6 Hz, 1H), 5.20 (d, *J* = 13.9 Hz, 1H), 4.60 – 4.50 (m, 1H), 4.49 – 4.42 (m, 1H), 4.38 (d, *J* = 13.9 Hz, 1H), 1.82 (s, 1H), 1.49 (s, 2H), 1.29 – 1.25 (m, 2H), 1.20 (t, *J* = 7.1 Hz, 1H); **^{13}C NMR** (100 MHz, $CDCl_3$) δ 177.2, 169.1, 168.8, 153.5, 147.9, 136.6, 134. 8, 133.9, 131.6, 128.6, 124.6, 123.4, 106.9, 52.0, 29.7, 18.3, 14.1; **HR-MS (ESI)** calcd for $[M + 1]^+$: $C_{19}H_{20}ClN_2O_4$: 375.1110, found: 371.1146; **IR** (KBr): 3476, 2989, 2945, 2888, 1785, 1743, 1663, 1568, 1535, 964 cm^{-1}.

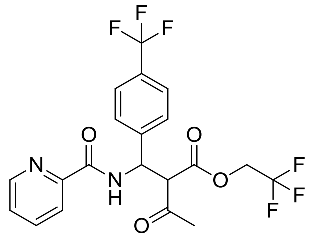

3-1c

2,2,2-Trifluoroethyl3-oxo-2-(picolinamido(4-(trifluoromethyl) phenyl)methyl)butanoate (3-1c): (10:1 crude dr), yellow oil, 40% yield. **^{1}H NMR** (400 MHz, $CDCl_3$) δ 11.74 (s, 1H), 8.42 (d, *J* = 4.7 Hz, 1H), 7.78 – 7.67 (m, 2H), 7.64 – 7.56 (m, 4H), 7.31 – 7.24 (m, 1H), 5.17 (d, *J* = 13.9 Hz, 1H), 4.57 (d, *J* = 14.0 Hz, 1H), 4.53 – 4.36 (m, 2H), 1.53 (s, 3H); **^{13}C NMR** (100 MHz, $CDCl_3$) δ 177.1, 169.2, 168.8, 153.3, 147.9, 136.6, 130.4, 125.5, 125.4, 125.4, 124.8, 123.5, 107.2, 107.1, 60.0, 52.5, 18.3; **^{19}F NMR** (376 MHz, $CDCl_3$) δ -62.6, -73.4. **HR-MS (ESI)** calcd for $[M + 1]^+$: $C_{20}H_{17}F_6N_2O_4$: 463.1087, found: 463.1060; **IR** (KBr): 3503, 3008, 2830, 1740, 1647, 1587, 1552, 1500, 1482, 1385, 971, 807 cm^{-1}.

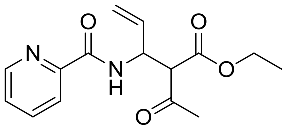

3-1d

Ethyl 2-acetyl-3-(picolinamido)pent-4-enoate (3-1d): (8:1 crude dr), yellow oil, 40% yield. **^{1}H NMR** (400 MHz, $CDCl_3$) δ 12.15 (s, 1H), 8.37 (d, *J* = 4.6 Hz, 1H), 7.61 (t, *J* = 7.1 Hz, 1H), 7.49 (d, *J* = 7.8 Hz, 1H), 7.16 (dd, *J* = 7.1, 5.1 Hz, 1H), 5.92 (td, *J* = 17.1, 8.5 Hz, 1H), 5.13 (dd, *J* = 20.2, 13.6 Hz, 2H), 4.34 (dd, *J* = 14.2, 6.6 Hz, 1H), 4.17 (q, *J* = 7.1 Hz, 2H), 4.00 (dd, *J* = 14.2, 7.5 Hz, 1H), 1.86 (s, 3H), 1.23 – 1.20 (m, 3H); **^{13}C NMR** (100 MHz, $CDCl_3$) δ 175.0, 170.7, 169.5, 154.4, 148.2, 136.3, 132.7, 124.2, 122.6, 119.2, 108.1, 61.0, 52.3, 18.7, 14.2. **HR-MS (ESI)** calcd for $[M + Na]^+$: $C_{15}H_{18}N_2O_4$ Na: 313.1188, found: 313.1199 ; **IR** (KBr): 3469, 2983, 2903, 1742, 1652, 1568, 1470, 1439, 1391, 1184, 881 cm^{-1}.

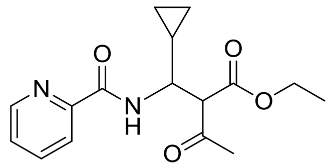

3-1e

Ethyl2-(cyclopropyl(picolinamido)methyl)-3-oxobutanoate (3-1e): (7:1 crude dr), yellow oil, 49% yield. **^{1}H NMR** (400 MHz, $CDCl_3$) δ 11.66 (s, 1H), 8.34 (d, *J* = 4.7 Hz, 1H), 7.66 – 7.60 (m, 1H), 7.56 (d, *J* = 7.8 Hz, 1H), 7.19 – 7.13 (m, 1H), 4.60 – 4.49 (m, 1H), 4.47 – 4.35 (m, 1H), 3.67 (dd, *J* = 13.9, 7.4 Hz, 1H), 3.28 (dd, *J* = 13.9, 7.1 Hz, 1H), 1.96 (s, 3H), 1.26 – 1.16 (m, 3H), 0.85 – 0.74 (m, 1H), 0.55 – 0.39 (m, 2H), 0.28 – 0.16 (m, 2H); **^{13}C NMR** (100 MHz, $CDCl_3$) δ 174.4, 172.4, 163.9, 149.9, 147.9, 137.9, 137.4, 131.9, 131.5, 129.7, 128.6, 126.2, 122.3, 101.7, 60.8, 41.6, 21.2, 19.8, 14.2. **HR-MS (ESI)** calcd for $[M + 1]^+$: $C_{16}H_{21}N_2O_4$:

305.1496, found: 305.1506; **IR** (KBr): 3451, 3004, 2921, 2850, 1737, 1651, 1587, 1526, 1380, 804 cm^{-1}.

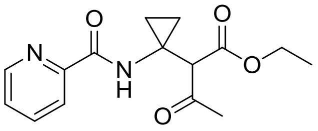

3-1f

Ethyl 3-oxo-2-(1-(picolinamido)cyclopropyl)butanoate (3-1f): (2:1 crude dr), yellow oil, 46% yield. **^{1}H NMR** (400 MHz, $CDCl_3$) δ 12.11 (s, 1H), 8.35 (d, *J* = 4.7 Hz, 1H), 7.61 (t, *J* = 7.7 Hz, 1H), 7.51 – 7.45 (m, 1H), 7.18 – 7.13 (m, 1H), 4.31 – 4.20 (m, 1H), 4.19 – 4.09 (m, 1H), 3.24 – 3.14 (m, 1H), 2.02 (s, 1H), 1.85 (s, 2H), 1.29 – 1.23 (m, 1H), 1.20 (t, *J* = 7.2 Hz, 2H), 0.83 – 0.71 (m, 2H), 0.66 – 0.56 (m, 2H); **^{13}C NMR** (100 MHz, $CDCl_3$) δ 174.8, 171.2, 170.9, 154.7, 148.1, 136.3, 124.2, 122.5, 107.6, 61.0, 31.7, 18.5, 14.2, 5.7, 5.5. **HR-MS (ESI)** calcd for $[M + 1]^+$: $C_{15}H_{19}N_2O_4$: 291.1339, found: 291.1338; **IR** (KBr): 3499, 3010, 2985, 2919, 1736, 1653, 1587, 1437, 1366, 1292, 847, 750 cm^{-1}.

V. Control experiments for mechanism studies

1. Procedure for Rh(III)-catalyzed Csp^3-H bond carbenoid insertion of *N*-butylbenzamide (1w) with ethyl 2-diazo-3-oxobutanoate (2a)

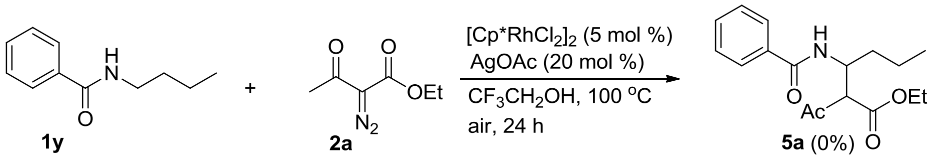

To a screw capped vial with a spinvane triangular-shaped Teflon stir bar were added *N*-butylbenzamide (**1y**, 0.1 mmol), ethyl 2-diazo-3-oxobutanoate (**2a**, 0.2 mmol), $[Cp^*RhCl_2]_2$ (7 mg, 0.005 mmol, 5 mol %), AgOAc (6 mg, 0.02 mmol, 20 mol %) and 2,2,2-trifluoroethanol, (1.0 mL) under Ar atmosphere conditions. The reaction mixture was stirred at 100 °C for 24 h, the reaction mixture was detected by TLC and ^{1}H NMR method, and no desired **5a** was found.

2. Procedure for Rh(III)-catalyzed *N*-methylene Csp^3-H bond carbenoid insertion of *N,N*-dibutylpicolinamide (1x) with ethyl 2-diazo-3-oxobutanoate (2a)

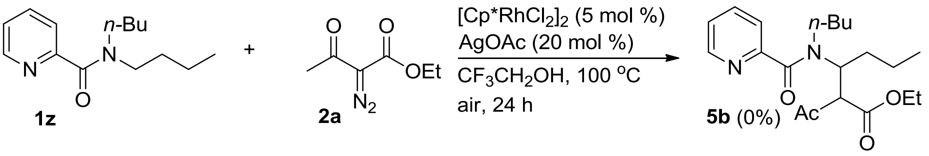

To a screw capped vial with a spinvane triangular-shaped Teflon stir bar were added *N,N*-dibutylpicolinamide (**1z**, 0.1 mmol), ethyl 2-diazo-3-oxobutanoate (**2a**, 0.2 mmol), $[Cp^*RhCl_2]_2$ (7 mg, 0.005 mmol, 5 mol %), AgOAc (6 mg, 0.02 mmol, 20 mol %) and 2,2,2-trifluoroethanol (1.0 mL) under Ar atmosphere. The reaction mixture was stirred at 100 °C for 24 h, the reaction mixture was detected by TLC and ^{1}H NMR method, and no desired **5b** was found.

3. Rh(III)-catalyzed Csp^3-H carbenoid insertion of 1a with 2a in different deuterated solvent system

3-1 Rh(III)-catalyzed Csp^3-H carbenoid insertion of **1a** with **2a** in AcOD/CH_3CN system

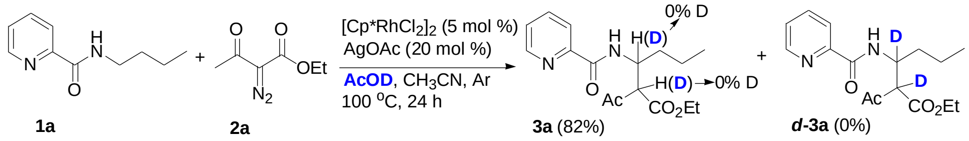

To a screw capped vial with a spinvane triangular-shaped Teflon stir bar were added *N*-butylbenzamide (**1a**, 0.1 mmol), ethyl 2-diazo-3-oxobutanoate (**2a**, 0.2 mmol), AcOD (2.0 equiv), $[Cp^*RhCl_2]_2$ (7 mg, 0.005 mmol, 5 mol %), AgOAc (6 mg, 0.02 mmol, 20 mol %) and CH_3CN (1.0 mL) under Ar atmosphere conditions. The reaction mixture was stirred at 100 ^oC for 24 h, filtered through a pad of celite and then washed with ethyl acetate (3 × 10 mL). The combined organic layers were removed under reduced pressure and the residue was purified by chromatography on silica gel. The product **3a** (no D was incorporated) was obtained in 82% yield and *d*-**3a** deuterium was not observed by ^1H NMR method.

3-2 Rh(III)-catalyzed Csp^3-H carbenoid insertion of **1a** with **2a** in solvent CF_3CD_2OD

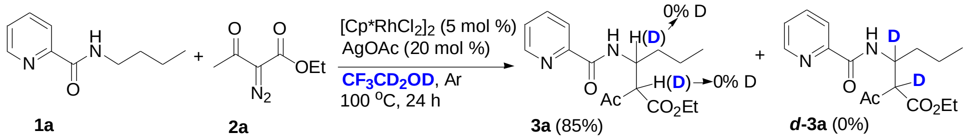

To a screw capped vial with a spinvane triangular-shaped Teflon stir bar were added *N*-butylpicolinamide (**1a**, 0.1 mmol), ethyl 2-diazo-3-oxobutanoate (**2a**, 0.2 mmol), $[Cp^*RhCl_2]_2$ (7 mg, 0.005 mmol, 5 mol %), AgOAc (6 mg, 0.02 mmol, 20 mol %) and CF_3CD_2OD (1.0 mL) under Ar atmosphere conditions. The reaction mixture was stirred at 100 ^oC for 24 h, filtered through a pad of celite and then washed with ethyl acetate (3 × 10 mL). The combined organic layers were removed under reduced pressure and the residue was purified by chromatography on silica gel. The product **3a** (no D was incorporated) was obtained in 85% yield and *d*-**3a** deuterium was not observed by ^1H NMR method.

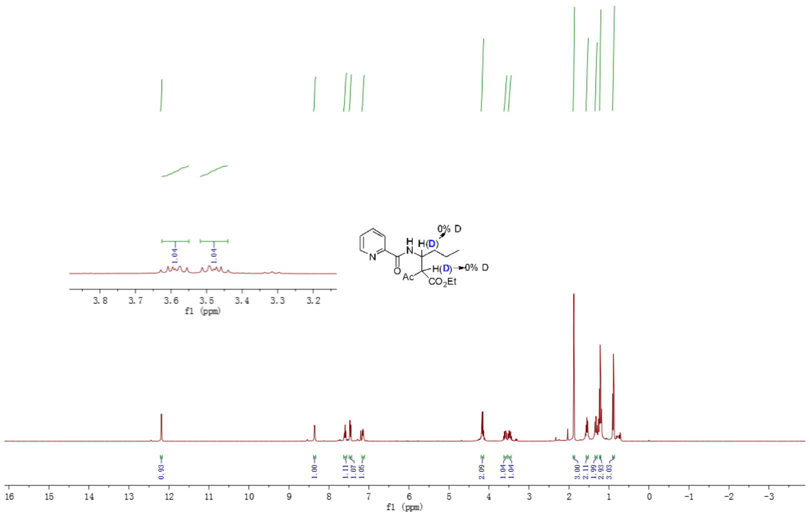

Figure S-1. ^{1}H NMR spectrum for **3a** derived from CF_3CD_2OD solvent system

4. Rh(III)-catalyzed Csp^3-H carbenoid insertion of *d*-1m with 2a

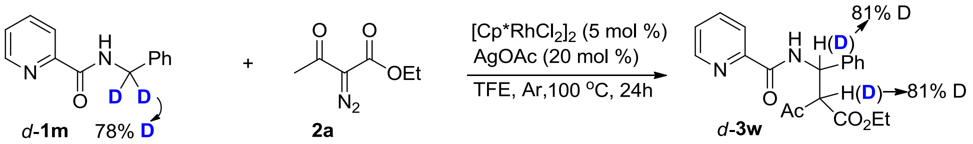

To a screw capped vial with a spinvane triangular-shaped Teflon stir bar were added *N*-benzylpicolinamide (*d*-**1m**, 78% D, 0.1 mmol), ethyl 2-diazo-3-oxobutanoate (**2a**, 0.2 mmol), [Cp*RhCl$_2$]$_2$ (7 mg, 0.005 mmol, 5 mol %), AgOAc (6 mg, 0.02 mmol, 20 mol %) and 2,2,2-trifluoroethanol, (1.0 mL) under Ar atmosphere. The reaction mixture was stirred at 100 °C for 24 h, filtered through a pad of celite and then washed with ethyl acetate (3 ×10 mL). The combined organic layers were removed under reduced pressure and the residue was purified by chromatography on silica gel. The deuterated product *d*-**3w** (81% D) was obtained.

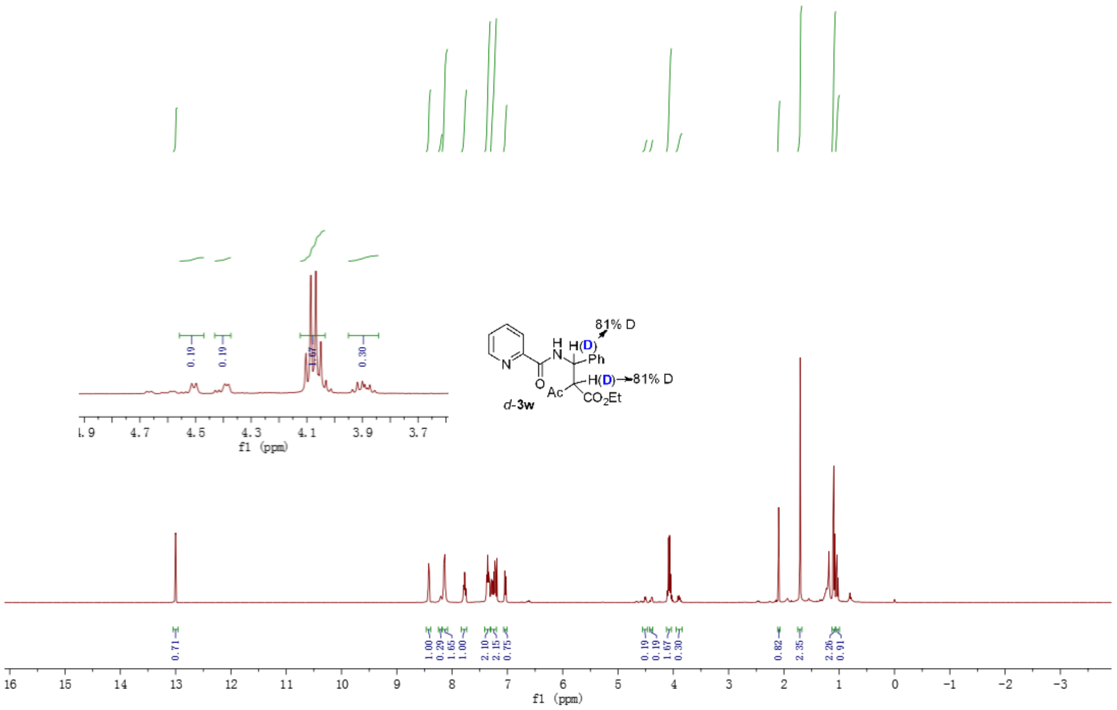

Figure S-2. ^{1}**H NMR** spectrum for *d*-**3w**

5. Kinetic isotope effect for this transformation

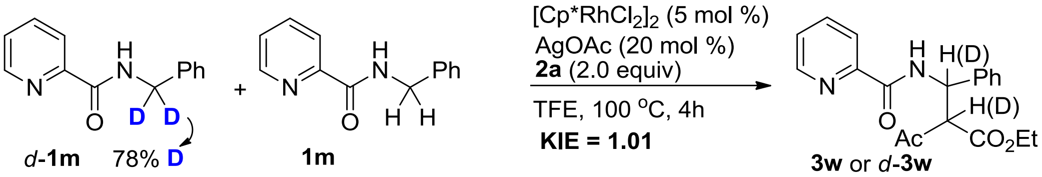

To a screw capped vial with a spinvane triangular-shaped Teflon stir bar were added *N*-benzylpicolinamide [**1m**: 7.6 mg, 0.036 mmol and *d*-**1m** (78% D)**:** 13.5 mg, 0.064 mmol],

ethyl 2-diazo-3-oxobutanoate **2a** (31.2 mg, 0.2 mmol), $[Cp^*RhCl_2]_2$ (7 mg, 0.005 mmol, 5 mol %), AgOAc (6 mg, 0.02 mmol, 20 mol %) and 2,2,2-trifluoroethanol (1.0 mL) under Ar atmosphere conditions. The reaction mixture was stirred at 100 °C for 4 h, and then was cooled to room temperature. Then the organic solvents were removed under reduced pressure and the residue was purified by chromatography on silica gel to give the desired products **3w** and *d*-**3w**. **^{1}H NMR** (400 MHz, $CDCl_3$) δ 13.00 (s, 1H), 8.41 (d, *J* = 3.3 Hz, 1H), 8.13 (d, *J* = 7.5 Hz, 1H), 7.80 – 7.72 (m, 1H), 7.40 – 7.17 (m, 5H), 7.03 (dd, *J* = 7.2, 1.5 Hz, 1H), 4.52 (dd, *J* = 14.7, 6.3 Hz, 0.47H), 4.40 (dd, *J* = 14.6, 5.4 Hz, 0.47H), 4.07 (dd, *J* = 14.2, 7.1 Hz, 2H), 2.09 (s, 1H), 1.70 (s, 2H), 1.09 (t, *J* = 7.1 Hz, 2H), 1.03 (t, *J* = 7.1 Hz, 1H).

The reaction progress in the early stage (4 hours) indicated a kinetic isotope effect (KIE) of 1.01.

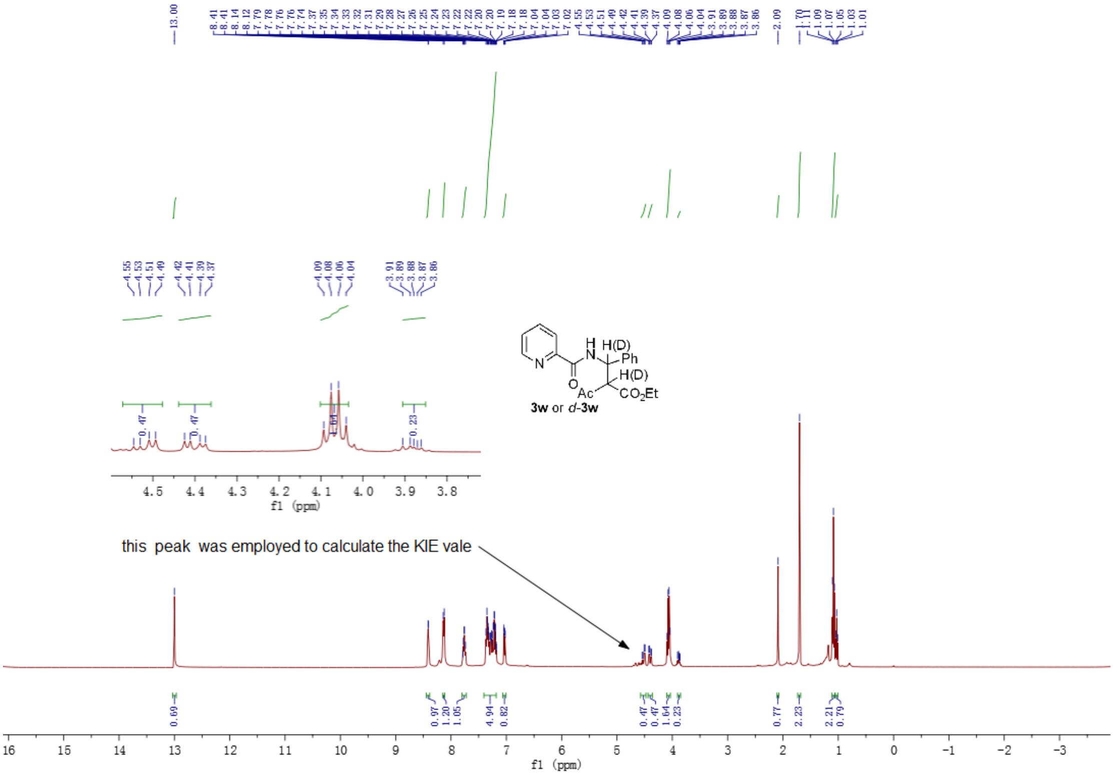

Figure S-3. ^{1}H NMR spectrum for competitive KIE analysis

6. Competition experiment for different dizao compounds differing in electron effects

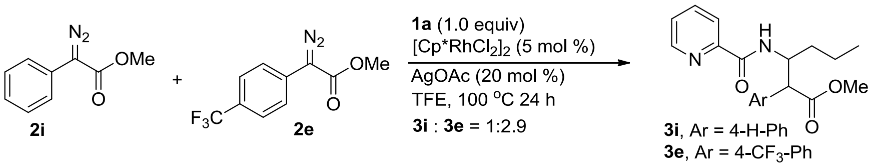

To a screw capped vial with a spinvane triangular-shaped Teflon stir bar were added *N*-butylpicolinamide (**1a**, 0.1 mmol), **2i** (17.6 mg, 0.1 mmol), **2e** (24.4 mg, 0.1 mmol), $[Cp^*RhCl_2]_2$ (7 mg, 0.005 mmol, 5 mol %), AgOAc (6 mg, 0.02 mmol, 20 mol %) and 2,2,2-trifluoroethanol (1.0 mL) under Ar atmosphere. The reaction mixture was stirred at 100 °C for 24 h, and then was cooled to room temperature. The solvent was removed under reduced pressure conditions and the ratio of **3i**/**3e** was analyzed by the crude ^{1}H NMR spectrum.

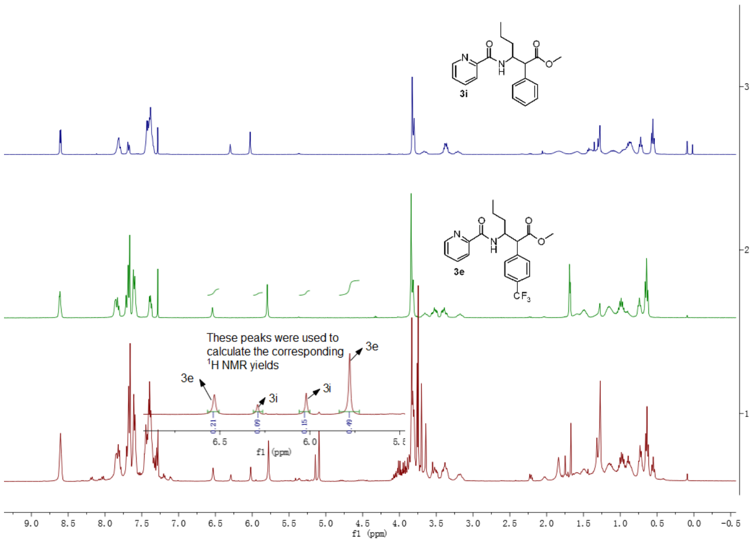

Figure S-4. The [1]**H NMR** spectrum of competition experiment of different diazo compounds **2i** and **2e**.

7. The effect of TEMPO on this transformation

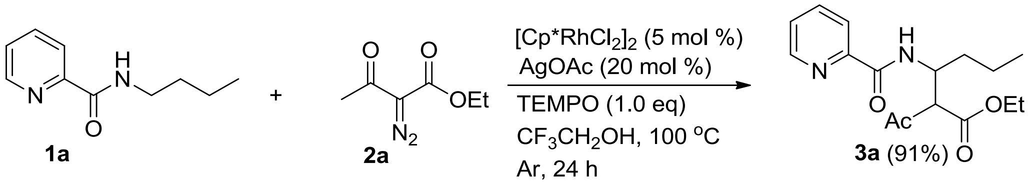

To a screw capped vial with a spinvane triangular-shaped Teflon stir bar were added *N*-butylpicolinamide (**1a**, 0.1 mmol), ethyl 2-diazo-3-oxobutanoate (**2a**, 0.2 mmol), $[Cp^*RhCl_2]_2$ (7 mg, 0.005 mmol, 5 mol %), AgOAc (6 mg, 0.02 mmol, 20 mol %) and TEMPO (15.6 mg, 1.0 eq) under Ar atmosphere conditions. The reaction mixture was stirred at 100 °C for 24 h, filtered through a pad of celite and then washed with ethyl acetate (3 × 10 mL). The combined organic layers were removed under reduced pressure and the residue was purified by chromatography on silica gel. The product **3a** was obtained in 91% yield.

VI. Computational details

All calculations were performed using Gaussian 09 D.01. Program.[7] Geometry optimizations were carried out at the M06-L[8]/BSI level (BSI designates the basis set combination of SDD[9] for metal atom and 6-31 G (d, p) for nonmetal atoms). Frequency analysis calculations were performed to characterize the structures to be the minima (no imaginary frequency) or transition states (one imaginary frequency). Transition states were verified by intrinsic reaction coordinate (IRC) calculations. With M06-L/BSI geometries, the energy results were further refined by calculating single point energy at the M06-L/BSII level with larger basis set (BSII designates SDD for metal atom and 6-311++g (d, p) for nonmetal atoms). The solvation effect of 2,2,2-trifluoroethanol was simulated by the SMD continuum solvent mode.[10] Since the calculation is based on ideal gas phase model, the ignorance of the suppression effect of solvent on the translational and rotational freedoms of reactants will cause the overestimation of the entropic contribution. Therefore, we adopt the MHP scheme proposed by Martin, Hay and Pratt, which is an approximate method to calculate entropy more accurately.[11] Based on this method, a correction of 4.3 kcal/mol is applied when a component of the reaction changes (i.e., a reaction from m components to n components, the correction is (n-m) × 4.3 kcal/mol). The 3D optimized structure figures in this paper were displayed by CYLview visualization program.[12] All of the thermodynamic data were obtained at 398.15 K, and additional computational information and the cartesian coordinates of the optimized structure are given in the supporting information.

The possible reaction pathways were proposed (see Figure S-5) and their corresponding free energy profiles were also provided by DFT calculations (see Figure S-6). Our DFT study excluded the inner sphere mechanism *via* bidentate-assisted *N*-methylene Csp^3-H bond activation, which is required to overcome an activation free energy of 49.1 kcal/mol (TS3, Fgiure S-5 and S-6) due to the three-membered ring strain. DFT calculations (Fgiure S-6, detailed pathways is shown in Figure S-5, SI) were carried out to further confirm the carbenoid insertion process. The whole process consists of two stages, the Rh-carbenoid formation and the carbene insertion into *N*-methylene Csp^3-H bond. In the Rh-carbenoid formation stage, the Rh-carbenoid is formed via transition state TS1, with the release of a molecule of N_2, upoun the approching of the nitrogen source, ethyl diazoacetoacetate, to the vacant site of the Rh center. The activation free energy of the formation of Rh-carbenoid is 33.8 kcal/mol (**Cat→TS1**). For the second stage, both the singlet and the triplet carbenoid instion pathways were evaluated. The singlet carbenoid firstly abstracts the hydride of the *N*-methylene (14.3 kcal/mol, Bs→**TS2$_S$-a**) to form intermediate **C$_S$** (-22.5 kcal/mol). Then, the alkyl ligand in **IM$_S$** nucleophilically attacks Csp^2 of imine via **TS2$_S$-b** ($\Delta G^‡$ = 20.0 kcal/mol) to produce product **D$_S$** (-32.9 kcal/mol). On the other hand, the triplet carbenoid firstly abstracts the H-atom of the *N*-methylene (43.4 kcal/mol, **TS2$_T$-a**) to form a radical intermediate (**C$_T$**, 7.6 kcal/mol). Then, the intermediate dissociate from Rh via **TS2$_T$-b** to form diradical intermediate **IM$_T$**, which subsequently recombines via **TS2$_T$-c** (20.8 kcal/mol) to produce carbenoid insertion product **D$_T$** (-4.5 kcal/mol). DFT results suggest that the carbenoid insertion proceeds in a singlet Fischer type carbene manner ($\Delta G^‡$ = 21.9 kcal/mol). The triplet pathway through radical recombination is less feasible due to the high activation free energy ($\Delta G^‡$ = 43.4 kcal/mol). Noteworthily, the rate-determining step is suggested to be the release of N_2 to

form Rh-carbenoid via **TS1** ($\Delta G^{\ddagger}$ = 33.8 kcal/mol), which is in good agreement with the controle expriment in Eq. 4e, Scheme 2. The competitive *N*-methylene C-H carbenoid insertion between *alpha*-aryl-*alpha*-diazo esters differing in electronic effects indicates that an electron-deficient diazo compound tended to form rhodium carbene at a relatively higher rate. This is further supported by our observed KIE effect (k_H/k_D = 1.01), suggesting that C-H bond carbenoid insertion did not involve the rate-limiting step of this transformation.

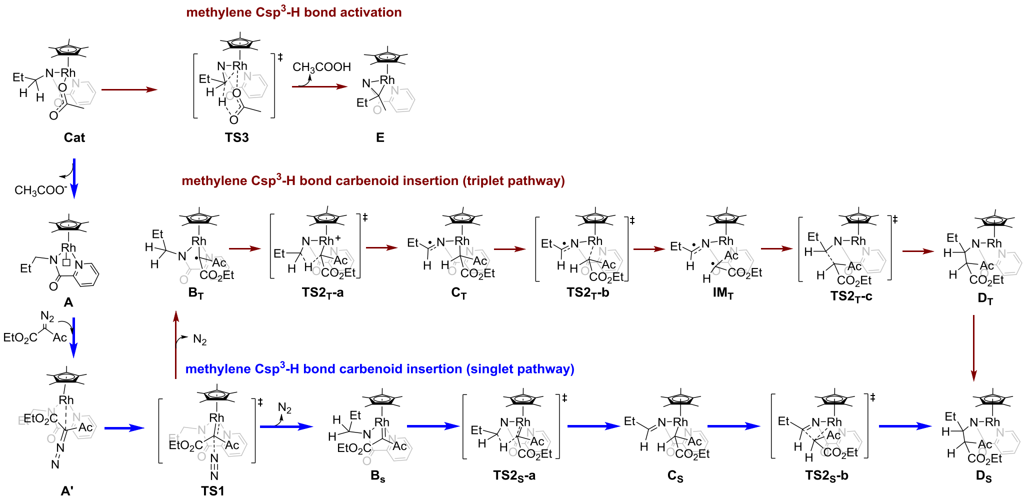

Figure S-5. The detailed reaction pathways for the Rh(III)-catalyzed regioselective methylene Csp^3-H bond carbenoid functionalization.

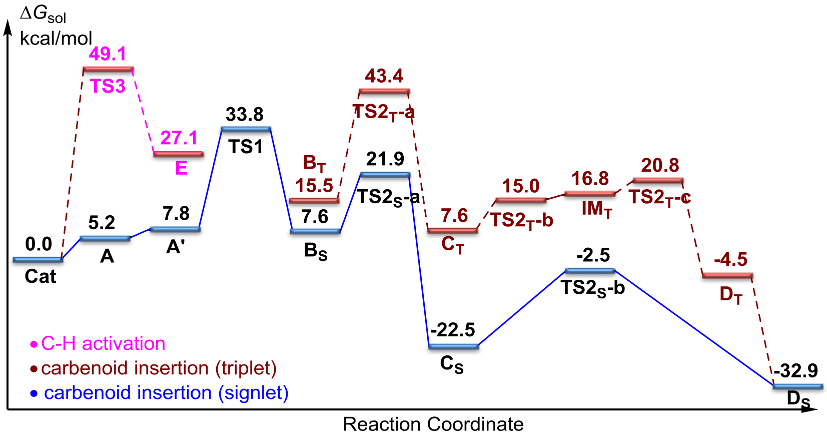

Figure S-6. The free energy profiles for the Rh(III)-catalyzed regioselective methylene Csp^3-H bond carbenoid functionalization. The free energies are reported in kcal/mol at the M06-L/BSII/SMD (dichloroethane)//M06-L/BSI level of theory.

Table S-5. The absolute energies of all optimized structures

Species	$Zero_c$	H_c	G_c	deltaS	Gc(398 K)	E_0	H	G	SP+SOL	Final
	**(BSI)**	**(BSI)**	**(BSI)**	**(BSI)**	**(BSI)**	**(BSI)**	**(BSI)**	**(BSI)**	**(BSII)**	**Free energy**
N_2	0.005541	0.008846	-0.012916	7.29901E-05	-0.020215011	-109.509194	-109.505889	-109.527652	-109.539279	-109.5526415
CH_3COO^-	0.048337	0.053741	0.020761	0.000110615	0.009699454	-228.431995	-228.426591	-228.459571	-228.66656	-228.650008
CH_3COOH	0.062356	0.067803	0.035392	0.000108707	0.024521297	-229.004367	-228.99892	-229.031331	-229.13597	-229.1045962
$C_6H_8O_3N_2$	0.143583	0.156288	0.10503	0.00017192	0.087837983	-568.414153	-568.401448	-568.452706	-568.701463	-568.6067725
A	0.415835	0.441676	0.363049	0.000263716	0.336677375	-1034.434754	-1034.408913	-1034.48754	-1035.132475	-1034.788945
A'	0.562332	0.600963	0.494178	0.000358159	0.458362136	-1602.86996	-1602.831328	-1602.938114	-1603.856727	-1603.391512
B_S	0.54985	0.586936	0.483656	0.000346403	0.449015718	-1493.349458	-1493.312372	-1493.415653	-1494.295145	-1493.839277
B_T	0.55047	0.587088	0.484651	0.000343575	0.450293462	-1493.343601	-1493.306983	-1493.40942	-1494.283766	-1493.82662
D_S	0.553568	0.589932	0.486452	0.000347074	0.451744638	-1493.408221	-1493.371858	-1493.475337	-1494.362303	-1493.903706
D_T	0.552977	0.589237	0.486382	0.000344977	0.451884264	-1493.375609	-1493.339349	-1493.442203	-1494.317223	-1493.858486
Cat	0.466872	0.498313	0.406948	0.00030644	0.376304029	-1263.065794	-1263.034353	-1263.125718	-1263.830369	-1263.447212
E	0.401337	0.427188	0.348588	0.000263626	0.322225431	-1034.006609	-1033.980758	-1034.059357	-1034.628586	-1034.299508
C_S	0.549382	0.586636	0.481921	0.000351216	0.446799417	-1493.355194	-1493.31794	-1493.422656	-1494.340821	-1493.887169
C_T	0.549948	0.586984	0.483337	0.000347634	0.448573626	-1493.347373	-1493.310337	-1493.413984	-1494.294638	-1493.839212
IM_T	0.549096	0.586027	0.482791	0.000346255	0.448165476	-1493.346187	-1493.309255	-1493.412492	-1494.279539	-1493.824521
TS1	0.558835	0.597214	0.493246	0.00034871	0.458374962	-1602.837712	-1602.799333	-1602.903301	-1603.81542	-1603.350193
$TS2_S$-a	0.547188	0.583315	0.482463	0.000338259	0.448637073	-1493.334839	-1493.298712	-1493.399565	-1494.271918	-1493.816428
$TS2_S$-b	0.549798	0.585514	0.486351	0.000332594	0.453091567	-1493.349249	-1493.313533	-1493.412697	-1494.315319	-1493.855375
$TS2_T$-a	0.543221	0.580018	0.476412	0.000347496	0.441662377	-1493.293676	-1493.25688	-1493.360485	-1494.230708	-1493.782193
$TS2_T$-b	0.548069	0.584784	0.481516	0.000346363	0.446879743	-1493.342212	-1493.305497	-1493.408765	-1494.281204	-1493.827472
$TS2_T$-c	0.548825	0.585249	0.482502	0.000344615	0.448040487	-1493.333172	-1493.296749	-1493.399496	-1494.27301	-1493.818117
TS3	0.459871	0.491293	0.39964	0.000307406	0.368899433	-1262.992242	-1262.96082	-1263.052473	-1263.744783	-1263.369031

- The **$Zero_C$, H_C and G_C** designate the thermal correction to Energy, Enthalpy and Gibbs Free Energy.
- The **deltaS** is equal to $\frac{G_c - H_c}{298.15K}$. The **Gc(398 K)** is equal to $(\mathbf{H_c} - \mathbf{deltaS} \cdot 398.15K)$.
- The **E, H** and **G** designate the Electronic Energy with ZPE correction, Enthalpy and Gibbs Free Energy in gas phase, respectively.
- The **SP + Sol G** designates the sum of single point energy at BSII level and the solvation free energy.
- The Final Free energy results are given with the correction of MHP scheme.

The Cartesian coordinates (xyz) for all optimized structures are presented

A

C	-2.587366	0.254679	-0.157037
C	-3.962749	0.096465	-0.096254
C	-4.477636	-1.150296	0.245424
C	-3.596645	-2.194471	0.511478
C	-2.229806	-1.959476	0.426575
H	-4.586931	0.955529	-0.319553
H	-5.550021	-1.307182	0.303590
H	-3.954921	-3.181366	0.782282
H	-1.502459	-2.742707	0.622832
N	-1.736491	-0.756631	0.099747
C	-1.962043	1.565024	-0.508645
N	-0.582500	1.549660	-0.494164
C	0.039167	2.833940	-0.808283
H	1.086415	2.637981	-1.059975
H	-0.448498	3.251681	-1.696597
C	-0.052824	3.839698	0.337874
C	0.633977	3.384631	1.611549
H	0.240020	2.422045	1.959567
H	1.715868	3.272761	1.466540
O	-2.654139	2.537194	-0.781967
Rh	0.322063	-0.182825	-0.098801
C	2.021039	-0.849478	-1.220041
C	1.431398	-1.995827	-0.547586
C	1.495947	-1.759195	0.856153
C	2.470609	0.083229	-0.223591
C	2.116811	-0.466828	1.060475
H	0.386214	4.781012	-0.011042
H	-1.109463	4.050422	0.528291
H	0.495983	4.105501	2.420709
C	2.399779	0.145366	2.379494
H	1.638292	-0.110256	3.120295
H	3.358648	-0.228158	2.760011
H	2.471116	1.233162	2.319751
C	1.039942	-2.659099	1.948113
H	1.900644	-3.101123	2.461993
H	0.456609	-2.123824	2.703286
H	0.432855	-3.486702	1.574631
C	0.877420	-3.189456	-1.236993
H	1.686696	-3.814510	-1.630123
H	0.284462	-3.814508	-0.565842
H	0.244239	-2.911239	-2.084290
C	2.171552	-0.697681	-2.686600
H	3.130718	-1.121156	-3.008607
H	1.385868	-1.222002	-3.234402
H	2.160286	0.351222	-2.991330
C	3.267017	1.313921	-0.458917
H	4.336267	1.091449	-0.368750
H	3.106126	1.723998	-1.458377
H	3.034658	2.095444	0.268685

A'

C	0.685364	-2.292439	-1.057886
C	0.722525	-3.648259	-1.341079
C	1.811322	-4.392069	-0.902960
C	2.825060	-3.750584	-0.196430
C	2.717156	-2.387980	0.049078
H	-0.116627	-4.081878	-1.874391
H	1.868220	-5.457402	-1.101862
H	3.691580	-4.292710	0.165479
H	3.482259	-1.847724	0.598716
N	1.665846	-1.671416	-0.375105
C	-0.465986	-1.438212	-1.473392
N	-0.336584	-0.118216	-1.099696
C	-1.400394	0.763555	-1.555118
H	-2.368046	0.254300	-1.442438
H	-1.413414	1.634387	-0.891145
C	-1.227131	1.202158	-3.004630
C	-2.240142	2.259368	-3.402496
H	-3.264055	1.883874	-3.311084
H	-2.163181	3.151007	-2.767668
O	-1.423859	-1.906424	-2.078288
Rh	1.289453	0.435087	-0.089553
C	1.067099	2.482799	0.582585
C	1.993841	2.417047	-0.503367
C	3.115305	1.575559	-0.093676
C	1.607999	1.679428	1.660875

C	2.880848	1.151874	1.250452
C	4.298474	1.271832	-0.936581
H	4.989849	2.122391	-0.946712
H	4.852283	0.405181	-0.569547
H	4.013518	1.075241	-1.973530
C	1.883933	3.118935	-1.804772
H	2.450621	4.057200	-1.771392
H	2.293331	2.523794	-2.624867
H	0.848357	3.368708	-2.046328
C	-0.174050	3.293124	0.673136
H	-0.979810	2.734770	1.161173
H	0.012027	4.192982	1.270851
H	-0.521439	3.623906	-0.309195
C	0.977072	1.511784	2.990327
H	1.400775	0.670173	3.542534
H	1.150454	2.416416	3.586744
H	-0.104586	1.373358	2.908881
C	3.772567	0.325532	2.107259
H	4.590764	-0.121748	1.538480
H	4.227547	0.942640	2.889371
H	3.224409	-0.477096	2.611561
H	-0.204199	1.578982	-3.138289
H	-2.102096	2.579857	-4.437470
H	-1.316263	0.321762	-3.649784
C	-2.619334	-1.393000	1.024766
C	-1.479340	-2.240346	1.421370
C	-2.873734	0.006655	1.351045
N	-3.495771	-1.994011	0.232514
O	-1.349702	-3.363660	0.955356
O	-2.067847	0.734695	1.912825
O	-4.099994	0.379724	0.950153
N	-4.196418	-2.561758	-0.452547
C	-4.502125	1.741348	1.248862
H	-5.577172	1.666700	1.420866
H	-4.011851	2.050070	2.175285
C	-0.501653	-1.665265	2.406773
H	-0.996197	-1.279926	3.302293
H	0.214280	-2.445847	2.669484
H	0.019530	-0.811718	1.958819
C	-4.181299	2.665100	0.104190
H	-4.579505	3.662648	0.305657
H	-3.100720	2.755783	-0.038977
H	-4.624591	2.306457	-0.828169

B$_S$

C	2.463559	1.286107	-0.543870
C	3.706320	1.725348	-0.982475
C	4.682967	0.787386	-1.286080
C	4.381031	-0.568171	-1.159089
C	3.116294	-0.938081	-0.725650
H	3.852073	2.796198	-1.084150
H	5.663515	1.101481	-1.629635
H	5.108276	-1.335264	-1.401366
H	2.823361	-1.980591	-0.632095
N	2.183102	-0.022509	-0.417182
C	1.341336	2.237789	-0.297139
N	0.239437	1.654691	0.248426
C	-1.027306	2.374134	0.136288
H	-1.243775	2.541294	-0.931524
H	-1.809353	1.695408	0.499659
C	-1.123032	3.693417	0.896931
C	-2.567084	4.155971	0.975355
H	-2.999089	4.286225	-0.022322
H	-3.195533	3.430730	1.508965
O	1.447204	3.424248	-0.595112
Rh	0.231334	-0.416379	0.345549
C	-1.371035	-0.946516	1.818173
C	-0.384313	-0.192120	2.560683
C	0.855512	-0.876088	2.498670
C	-0.736928	-2.157023	1.366831
C	0.648477	-2.093868	1.739619
C	2.130251	-0.429718	3.119912
H	2.173544	-0.718537	4.175804
H	3.001957	-0.870703	2.629813
H	2.235198	0.658372	3.078878
C	-0.631768	1.093665	3.253689
H	-0.744713	0.920561	4.330097
H	0.197511	1.792595	3.117453
H	-1.545682	1.577425	2.901515
C	-2.827185	-0.649463	1.783778
H	-3.340776	-1.211011	1.001464
H	-3.288687	-0.920341	2.740454
H	-3.027823	0.413285	1.618905
C	-1.407033	-3.320746	0.733060
H	-0.783336	-3.800252	-0.024846
H	-1.629973	-4.071285	1.500199

H	-2.350535	-3.051759	0.256032
C	1.643447	-3.181430	1.547644
H	2.669677	-2.805710	1.540965
H	1.577007	-3.903209	2.370126
H	1.469505	-3.737036	0.621839
H	-0.707148	3.566674	1.903826
H	-2.656623	5.112029	1.495520
H	-0.496793	4.438270	0.401471
C	-0.460369	-0.632364	-1.432200
C	0.319245	-0.266447	-2.647889
C	-1.822203	-1.132741	-1.710519
O	0.491465	0.925928	-2.847329
O	-1.991888	-2.254313	-2.157215
O	-2.764898	-0.241127	-1.425660
C	-4.134370	-0.620080	-1.765635
H	-4.152356	-0.917127	-2.817993
H	-4.397972	-1.502040	-1.171051
C	0.818701	-1.365137	-3.528133
H	-0.027260	-1.878505	-3.997121
H	1.477561	-0.959223	-4.296917
H	1.343475	-2.125903	-2.940556
C	-5.001873	0.568600	-1.471386
H	-6.042067	0.337739	-1.709592
H	-4.951171	0.847162	-0.415196
H	-4.700708	1.432413	-2.067913

B_T

C	1.173495	2.128619	1.157588
C	1.932196	3.247483	1.468745
C	3.317973	3.151323	1.408141
C	3.893191	1.940644	1.035401
C	3.070776	0.864049	0.721687
H	1.411921	4.157396	1.749594
H	3.940678	4.007591	1.645863
H	4.968983	1.822796	0.971440
H	3.468100	-0.083877	0.377428
N	1.737055	0.960134	0.791646
C	-0.310100	2.142217	1.235869
N	-0.881197	0.914266	0.954458
C	-2.323318	0.837602	1.083612
H	-2.769627	1.679175	0.533495
H	-2.647208	-0.084640	0.587865
C	-2.829061	0.871625	2.526211
C	-4.315942	0.573225	2.574547
H	-4.888444	1.305655	1.996585
H	-4.542143	-0.416961	2.159328
O	-0.947771	3.143600	1.539869
Rh	0.308662	-0.542524	0.235659
C	-0.785126	-2.380005	-0.287558
C	-0.584535	-2.381769	1.137029
C	0.827568	-2.382706	1.387027
C	0.519698	-2.463998	-0.914477
C	1.507422	-2.452583	0.109017
C	1.488567	-2.407894	2.717012
H	1.741802	-3.436039	3.001170
H	2.422155	-1.838888	2.718307
H	0.846234	-2.000081	3.500384
C	-1.660006	-2.444774	2.157157
H	-1.937275	-3.490852	2.332811
H	-1.348447	-2.025126	3.115632
H	-2.567680	-1.928339	1.833129
C	-2.089704	-2.487650	-0.987491
H	-2.051812	-2.036253	-1.979675
H	-2.370768	-3.541656	-1.101469
H	-2.895033	-1.996947	-0.432990
C	0.757161	-2.650764	-2.367348
H	1.762015	-2.341821	-2.657151
H	0.643186	-3.711592	-2.619442
H	0.035568	-2.095764	-2.973755
C	2.969713	-2.625378	-0.083124
H	3.535206	-2.326275	0.802719
H	3.197105	-3.683092	-0.259358
H	3.332867	-2.057924	-0.942103
H	-2.268426	0.140884	3.122089
H	-4.695987	0.594175	3.597913
H	-2.613453	1.855380	2.952194
C	0.347472	0.423271	-1.603820
C	1.584572	0.491187	-2.372181
C	-0.823082	1.240837	-1.962942
O	2.603250	-0.089133	-1.971918
O	-0.742683	2.433798	-2.198021
O	-1.974251	0.543460	-1.968378
C	-3.161021	1.321756	-2.292667
H	-3.152801	2.231476	-1.683752
H	-3.085182	1.635113	-3.338486
C	1.591608	1.254189	-3.669808

H	1.523381	2.327864	-3.475023
H	2.513135	1.037785	-4.209940
H	0.728106	1.004883	-4.293780
C	-4.362914	0.457962	-2.036576
H	-5.271858	1.001852	-2.302545
H	-4.334343	-0.456753	-2.634083
H	-4.438724	0.179030	-0.980503

D$_S$

C	1.423533	2.504733	0.429902
C	1.773509	3.843894	0.488911
C	3.046108	4.221572	0.072533
C	3.921982	3.242668	-0.387132
C	3.501251	1.918543	-0.410179
H	1.032928	4.548535	0.852647
H	3.349898	5.262954	0.100994
H	4.920912	3.492499	-0.726800
H	4.152040	1.122529	-0.759760
N	2.275236	1.555717	-0.006865
C	0.060603	2.036614	0.822968
O	-0.760777	2.802266	1.307189
Rh	1.423296	-0.420611	0.056581
C	0.895405	-2.363793	-0.771715
C	1.047104	-2.468835	0.639394
C	2.413094	-2.084408	0.965844
C	2.169184	-1.909251	-1.326796
C	3.110315	-1.798620	-0.257619
C	2.995574	-2.069997	2.328462
H	3.353682	-3.072519	2.593657
H	3.847517	-1.390764	2.402942
H	2.258746	-1.780946	3.081818
C	0.025907	-2.971478	1.593008
H	0.169934	-4.045896	1.757046
H	0.091343	-2.484827	2.569063
H	-0.989295	-2.838042	1.209068
C	-0.314155	-2.744797	-1.543680
H	-0.267907	-2.399491	-2.578034
H	-0.394595	-3.838072	-1.567702
H	-1.244854	-2.367327	-1.102536
C	2.433665	-1.672975	-2.766916
H	3.277052	-0.996151	-2.921016
H	2.673705	-2.616590	-3.271184
H	1.563743	-1.248141	-3.274558
C	4.554372	-1.463066	-0.366552
H	4.888921	-0.810196	0.444852
H	5.158488	-2.375115	-0.310285
H	4.795402	-0.983131	-1.318170
N	-0.143741	0.699195	0.556514
C	-1.481036	0.151213	0.790733
H	-2.979511	1.709898	0.448133
H	-1.432225	-0.880872	0.423304
C	-1.835929	0.120680	2.280539
C	-3.109759	-0.645135	2.594676
H	-3.998531	-0.125988	2.221530
H	-3.106246	-1.648947	2.155012
H	-0.984125	-0.332200	2.806364
H	-3.238889	-0.752850	3.673949
H	-1.908213	1.149363	2.645384
C	-2.571019	0.841386	-0.073231
C	-1.967306	1.374275	-1.389985
C	-3.664258	-0.170197	-0.350534
O	-1.798011	2.565463	-1.539455
O	-3.460478	-1.337428	-0.642932
O	-4.873299	0.381488	-0.254903
C	-5.991624	-0.519370	-0.484025
H	-5.901301	-0.926078	-1.496037
H	-5.893528	-1.358204	0.213510
C	-1.531460	0.372034	-2.423232
H	-2.284062	-0.396804	-2.612833
H	-1.263686	0.889048	-3.344838
H	-0.645244	-0.146476	-2.035545
C	-7.252622	0.269027	-0.279911
H	-8.121854	-0.371246	-0.445435
H	-7.311437	0.664572	0.736620
H	-7.311725	1.107979	-0.976789

D$_T$

C	1.495046	2.528240	0.427704
C	1.943534	3.841211	0.408275
C	3.183278	4.119028	-0.155264
C	3.925059	3.072445	-0.697960
C	3.407249	1.785128	-0.655862
H	1.301719	4.606621	0.832125
H	3.564862	5.134773	-0.179226

H	4.892467	3.244245	-1.156385
H	3.943998	0.939145	-1.076091
N	2.216270	1.515548	-0.099277
C	0.149895	2.159994	0.953541
O	-0.709977	3.023802	1.148381
Rh	1.291376	-0.383854	0.132975
C	0.773876	-2.373456	-0.992722
C	0.729711	-2.560078	0.430232
C	2.066105	-2.393332	0.950389
C	2.085077	-1.900458	-1.323035
C	2.895522	-1.946381	-0.119821
C	2.477367	-2.598936	2.362207
H	2.693439	-3.657804	2.551340
H	3.379617	-2.036910	2.615372
H	1.688693	-2.309033	3.062366
C	-0.426236	-3.086910	1.204271
H	-0.361626	-4.178543	1.281691
H	-0.444055	-2.696490	2.227129
H	-1.381420	-2.846722	0.729928
C	-0.355398	-2.569041	-1.932733
H	-0.282345	-1.907597	-2.799254
H	-0.356414	-3.599470	-2.309736
H	-1.320727	-2.398841	-1.448570
C	2.564924	-1.567810	-2.691013
H	3.430721	-0.900765	-2.672880
H	2.868621	-2.471722	-3.231323
H	1.783281	-1.082303	-3.281571
C	4.360395	-1.697917	-0.040226
H	4.638991	-1.135840	0.856188
H	4.907331	-2.646752	-0.005949
H	4.732706	-1.154431	-0.912079
N	-0.021442	0.814693	1.121040
C	-1.391291	0.331841	1.244866
H	-2.804330	1.748781	0.354601
H	-1.328051	-0.755284	1.149481
C	-1.999188	0.643941	2.618706
C	-3.346000	-0.023052	2.837988
H	-4.119892	0.385938	2.178823
H	-3.298036	-1.104206	2.664558
H	-1.281057	0.298945	3.371865
H	-3.692229	0.133035	3.862006
H	-2.074814	1.729820	2.731605
C	-2.292057	0.818134	0.088400
C	-1.558797	1.072391	-1.225077
C	-3.353773	-0.237253	-0.221311
O	-0.496259	0.529390	-1.522846
O	-3.137981	-1.433044	-0.223866
O	-4.527287	0.326430	-0.506227
C	-5.613288	-0.604950	-0.792441
H	-5.298959	-1.248435	-1.619440
H	-5.741411	-1.241631	0.088732
C	-2.213067	2.040649	-2.154350
H	-2.062164	3.047154	-1.745165
H	-1.777504	1.991872	-3.151438
H	-3.294893	1.884546	-2.189286
C	-6.833171	0.209697	-1.108749
H	-7.675144	-0.453261	-1.318588
H	-7.109495	0.851757	-0.269727
H	-6.674176	0.839803	-1.986951

Cat

C	-1.781489	1.900350	-0.260503
C	-2.840541	2.796314	-0.150141
C	-4.044446	2.358061	0.380686
C	-4.155161	1.032541	0.798592
C	-3.056413	0.196549	0.661202
H	-2.665374	3.813243	-0.487453
H	-4.886870	3.037053	0.478159
H	-5.073475	0.648448	1.229866
H	-3.081222	-0.840204	0.989323
N	-1.903029	0.618812	0.127780
C	-0.459484	2.330089	-0.806246
N	0.462937	1.354310	-0.759284
C	1.783771	1.698615	-1.252508
H	2.347140	0.768821	-1.393918
H	1.683782	2.180368	-2.238689
C	2.577247	2.619588	-0.332739
C	3.964926	2.886371	-0.886628
H	4.534604	1.954141	-0.988780
H	3.921453	3.353256	-1.877415
O	-0.311827	3.482998	-1.241197
Rh	-0.094193	-0.533317	-0.031611
C	0.460038	-1.724521	-1.732589
C	-0.815784	-2.153026	-1.235736
C	-0.639203	-2.634533	0.120646
C	1.436685	-1.922647	-0.680750

C 0.746313 -2.488475 0.448317
H 2.018217 3.553933 -0.216578
H 2.651676 2.146878 0.651720
H 4.542569 3.549681 -0.236909
C 1.371451 -2.886835 1.734742
H 0.688473 -2.730114 2.574192
H 1.623470 -3.954489 1.709974
H 2.277370 -2.311127 1.926723
C -1.676813 -3.252763 0.990423
H -1.579372 -4.345227 1.010887
H -1.597498 -2.901370 2.024088
H -2.688121 -3.032099 0.635994
C -2.101633 -2.132954 -1.981941
H -2.287090 -3.099287 -2.465425
H -2.949332 -1.932861 -1.319047
H -2.102669 -1.365756 -2.760065
C 0.741671 -1.193218 -3.092229
H 1.032607 -1.999964 -3.775545
H -0.131789 -0.693283 -3.518472
H 1.556031 -0.464504 -3.074143
C 2.906547 -1.713151 -0.768835
H 3.432939 -2.674860 -0.738435
H 3.188064 -1.218675 -1.702011
H 3.252861 -1.101726 0.069523
C 1.330990 0.462896 2.351673
O 2.360858 0.057532 1.801225
O 0.119317 0.322283 1.910502
C 1.409890 1.245977 3.645585
H 0.504047 1.141713 4.245204
H 2.286316 0.944900 4.221681
H 1.522358 2.306665 3.398223

E

C 2.540672 0.049748 -0.473119
C 3.848214 0.515792 -0.465399
C 4.176476 1.619032 0.311117
C 3.170109 2.199694 1.086279
C 1.891058 1.669630 1.054864
H 4.570233 -0.013167 -1.080375
H 5.186645 2.016052 0.323464
H 3.374985 3.052450 1.725796
H 1.088735 2.076149 1.665826
N 1.557468 0.612675 0.282863
C 2.142965 -1.182255 -1.203151
N 0.766562 -1.433014 -1.211893
C 0.262540 -2.105351 -0.111716
H -0.640653 -2.694740 -0.314861
C 1.130198 -2.684468 0.974010
C 0.344779 -2.924571 2.250506
H -0.055108 -1.977866 2.632726
H -0.506526 -3.592411 2.075260
O 2.973207 -1.924436 -1.709714
Rh -0.366278 -0.088785 -0.089238
C -2.008105 0.902175 -1.319784
C -1.621122 1.799824 -0.301952
C -1.855574 1.161307 0.991215
C -2.429576 -0.326558 -0.673101
C -2.422006 -0.120052 0.757165
H 1.579739 -3.624531 0.618710
H 1.973796 -2.011693 1.175559
H 0.961935 -3.377338 3.031200
C -2.941676 -1.066969 1.781762
H -2.456686 -0.922299 2.751449
H -4.021308 -0.936485 1.933712
H -2.782794 -2.109865 1.490094
C -1.691488 1.821008 2.317287
H -2.619991 2.307287 2.644574
H -1.406511 1.104829 3.094697
H -0.925294 2.602878 2.290470
C -1.077841 3.175382 -0.469502
H -1.814929 3.938578 -0.189635
H -0.196819 3.345371 0.162392
H -0.776692 3.368979 -1.502218
C -1.962062 1.111119 -2.792139
H -2.965303 1.268793 -3.208635
H -1.355435 1.979734 -3.061094
H -1.533980 0.243237 -3.304038
C -2.976065 -1.515689 -1.385270
H -4.010792 -1.346466 -1.708734
H -2.389081 -1.753812 -2.278028
H -2.978521 -2.403292 -0.746098

C_S

C -2.370416 -0.375320 1.377321

C	-3.230054	-0.634602	2.438629
C	-2.721123	-1.255092	3.573910
C	-1.369737	-1.585782	3.608576
C	-0.567821	-1.301130	2.506762
H	-4.269902	-0.337914	2.347565
H	-3.364542	-1.470083	4.420784
H	-0.927642	-2.054902	4.480938
H	0.500678	-1.499452	2.492819
N	-1.063525	-0.713514	1.409308
C	-2.855615	0.345169	0.169910
O	-4.025812	0.608045	-0.018083
Rh	-0.019312	-0.477221	-0.462783
C	1.796358	-1.229406	-1.447381
C	0.796547	-0.854675	-2.412980
C	-0.345083	-1.736859	-2.223875
C	1.243937	-2.243894	-0.600964
C	-0.079546	-2.578034	-1.108374
C	-1.578123	-1.732341	-3.051994
H	-1.456467	-2.393943	-3.916794
H	-2.450235	-2.081098	-2.493170
H	-1.802916	-0.735390	-3.439288
C	0.977809	0.113372	-3.527159
H	1.474416	-0.362919	-4.380611
H	0.021740	0.498749	-3.891416
H	1.594285	0.965454	-3.228714
C	3.188917	-0.720357	-1.403736
H	3.658996	-0.908900	-0.435780
H	3.783075	-1.242766	-2.162734
H	3.255115	0.349177	-1.615239
C	1.955771	-2.937519	0.504154
H	1.259189	-3.445302	1.176380
H	2.629645	-3.704163	0.104299
H	2.553649	-2.240096	1.097260
C	-0.978304	-3.620219	-0.547845
H	-2.028423	-3.417356	-0.773833
H	-0.738848	-4.605390	-0.963297
H	-0.877566	-3.697492	0.538587
N	-1.800233	0.686605	-0.718140
C	-1.849290	1.797334	-1.391559
H	1.218720	1.931689	-0.572811
H	-0.969191	1.988153	-2.013907
C	-2.937398	2.799532	-1.464795
C	-2.446794	4.142258	-1.975209
H	-1.704771	4.563533	-1.294193
H	-1.992072	4.055890	-2.966813
H	-3.716023	2.382111	-2.119808
H	-3.274223	4.848613	-2.055537
H	-3.424579	2.881753	-0.488125
C	0.969026	1.363439	0.328191
C	-0.004040	2.138965	1.130795
C	2.191998	0.914697	1.045348
O	-0.819057	2.888965	0.584240
O	2.246433	0.047679	1.908948
O	3.278217	1.593547	0.629802
C	4.529810	1.239461	1.268681
H	4.476473	1.548151	2.317608
H	4.629664	0.147885	1.264550
C	-0.006744	2.042024	2.635243
H	0.580219	2.878746	3.030286
H	-1.025540	2.167206	3.008580
H	0.439604	1.124722	3.018062
C	5.632907	1.927773	0.516342
H	6.597576	1.704503	0.977308
H	5.670573	1.593846	-0.524112
H	5.498448	3.011629	0.521006

C_T

C	-1.106921	-1.486025	1.684422
C	-0.224631	-0.714458	2.499695
C	1.113227	-1.155340	2.222101
C	-0.334202	-2.515058	1.025752
C	1.048315	-2.311156	1.372114
C	2.348083	-0.596662	2.834361
H	2.556792	-1.080615	3.795553
H	3.226274	-0.752655	2.201879
H	2.258900	0.475267	3.034243
C	-0.602599	0.385498	3.419598
H	-0.620269	0.032369	4.458039
H	0.106430	1.218838	3.383653
H	-1.600039	0.776484	3.200554
C	-2.590061	-1.418885	1.686778
H	-3.007387	-1.855851	0.776223
H	-2.992477	-1.983372	2.536261
H	-2.957756	-0.392215	1.779591
C	-0.908061	-3.664525	0.279330
H	-0.138423	-4.228693	-0.252867

H	-1.393282	-4.359001	0.975694
H	-1.666750	-3.344396	-0.442126
C	2.194848	-3.169975	0.970209
H	3.127449	-2.601407	0.915527
H	2.354788	-3.976231	1.695524
H	2.032046	-3.643791	-0.002380
C	2.406312	1.634661	-0.244117
C	3.589983	2.277714	-0.591210
C	4.502930	1.612539	-1.400313
C	4.199590	0.326024	-1.833139
C	2.999377	-0.255463	-1.440189
H	3.758233	3.284114	-0.222469
H	5.432603	2.089919	-1.692337
H	4.876049	-0.230126	-2.472228
H	2.720703	-1.253982	-1.765498
N	2.118718	0.382202	-0.657755
C	1.402489	2.338143	0.591169
O	1.655687	3.345936	1.224110
Rh	0.339135	-0.595454	0.094546
N	0.147230	1.687508	0.636516
C	-0.957493	2.350886	0.654139
H	-0.767035	0.961695	-1.740588
H	-1.862775	1.742785	0.771530
C	-1.153984	3.812294	0.475600
C	-2.456718	4.134104	-0.241529
H	-2.481863	3.694132	-1.242539
H	-3.320519	3.753563	0.310533
H	-1.155515	4.253287	1.483920
H	-2.578753	5.213139	-0.346114
H	-0.286015	4.259445	-0.017472
C	-0.857591	-0.122553	-1.653235
C	-0.046942	-0.772129	-2.728597
C	-2.252812	-0.547926	-1.437181
O	0.822798	-0.113738	-3.291773
O	-2.716532	-1.664500	-1.609780
O	-3.011088	0.490535	-0.991052
C	-4.426163	0.204187	-0.860726
H	-4.820089	-0.022674	-1.856287
H	-4.554465	-0.697839	-0.254944
C	-0.247412	-2.224598	-3.061611
H	-1.271821	-2.425441	-3.383344
H	0.461330	-2.517790	-3.836470
H	-0.100694	-2.847435	-2.170394
C	-5.085243	1.405530	-0.245011
H	-6.156405	1.226187	-0.130294
H	-4.676451	1.618183	0.748657
H	-4.958375	2.294730	-0.867449

IM_T

C	0.153033	-2.557937	0.493951
C	1.289439	-2.426494	1.383566
C	2.454886	-2.235515	0.579260
C	0.629824	-2.533129	-0.858831
C	2.035436	-2.248188	-0.807843
C	3.850572	-2.064852	1.059548
H	4.404237	-1.343261	0.451201
H	3.884451	-1.720490	2.095770
H	4.400245	-3.012298	1.011357
C	1.233650	-2.488357	2.866805
H	2.067735	-1.956126	3.330329
H	0.305656	-2.057636	3.254595
H	1.272755	-3.527014	3.215993
C	-1.243592	-2.858717	0.911794
H	-1.973770	-2.454285	0.204555
H	-1.402539	-3.942332	0.965809
H	-1.464212	-2.461943	1.907843
C	-0.171417	-2.775656	-2.083843
H	0.280556	-2.305985	-2.962210
H	-0.228988	-3.851370	-2.294027
H	-1.194024	-2.405286	-1.982069
C	2.937449	-2.171446	-1.989800
H	3.900970	-1.719106	-1.740034
H	3.150562	-3.172886	-2.381058
H	2.489311	-1.599050	-2.809123
C	1.733514	2.266282	0.463527
C	2.382458	3.489160	0.373168
C	3.399723	3.640289	-0.562160
C	3.733016	2.551788	-1.362590
C	3.057296	1.349426	-1.192736
H	2.062702	4.294386	1.025905
H	3.918311	4.587182	-0.668935
H	4.512438	2.623513	-2.113287
H	3.297467	0.473994	-1.789144
N	2.069841	1.198776	-0.295282
C	0.594094	2.099197	1.395826
O	0.314700	2.912527	2.248830

Rh	1.040780	-0.593579	0.188831
N	-0.121456	0.861812	1.200716
C	-1.349720	0.767332	1.617028
H	-1.809642	-0.212684	1.441662
C	-2.199544	1.802119	2.261865
C	-3.668076	1.418399	2.295829
H	-4.091422	1.330216	1.290399
H	-3.820271	0.460868	2.804300
H	-1.810442	1.969539	3.275623
H	-4.251081	2.169255	2.831458
H	-2.031758	2.769611	1.771013
H	-3.039165	2.497607	-0.840546
C	-2.540148	1.627586	-1.262368
C	-1.161396	1.824343	-1.714779
C	-3.248656	0.349633	-1.249449
O	-0.540909	2.771637	-1.225180
O	-2.702912	-0.745097	-1.322519
O	-4.569080	0.531462	-1.103592
C	-5.369527	-0.680727	-1.039626
H	-6.358928	-0.360737	-1.368664
H	-4.963989	-1.397705	-1.757739
C	-0.567236	0.916646	-2.748161
H	-1.012681	1.142236	-3.724457
H	0.510341	1.078535	-2.812490
H	-0.790870	-0.129978	-2.532161
C	-5.385632	-1.239743	0.359557
H	-5.994320	-2.146517	0.390286
H	-4.374688	-1.501796	0.686842
H	-5.809832	-0.524366	1.068025

TS1

C	1.868837	2.049694	-0.077965
C	2.876317	2.957210	-0.382794
C	4.126850	2.478385	-0.747628
C	4.325678	1.101189	-0.820432
C	3.269568	0.254298	-0.519586
H	2.632554	4.013705	-0.334108
H	4.933758	3.163815	-0.986985
H	5.279341	0.682246	-1.121661
H	3.368692	-0.825547	-0.594353
N	2.069216	0.719901	-0.141649
C	0.485137	2.511783	0.238112
N	-0.359811	1.504887	0.566765
C	-1.766695	1.853692	0.726365
H	-2.150059	2.296011	-0.208232
H	-2.317501	0.912698	0.863859
C	-2.088192	2.811998	1.870557
C	-3.584050	2.865905	2.121732
H	-4.127460	3.193513	1.228761
H	-3.983361	1.881675	2.401418
O	0.176458	3.699738	0.113443
Rh	0.348886	-0.466411	0.404304
C	-0.957268	-1.901824	1.404680
C	-0.425259	-1.020836	2.411257
C	0.998965	-1.122633	2.395203
C	0.155689	-2.659425	0.859880
C	1.353219	-2.161964	1.442078
C	1.941140	-0.372929	3.267180
H	2.056518	-0.868266	4.237817
H	2.935780	-0.297708	2.820567
H	1.585680	0.643585	3.457220
C	-1.222262	-0.182379	3.336262
H	-1.473916	-0.773490	4.224795
H	-0.669659	0.696357	3.672956
H	-2.165364	0.148215	2.894955
C	-2.398020	-2.173182	1.154610
H	-2.555165	-2.610274	0.164907
H	-2.793485	-2.882090	1.891488
H	-3.000622	-1.261331	1.230407
C	0.030143	-3.847203	-0.021302
H	0.992047	-4.163477	-0.430429
H	-0.357708	-4.685604	0.569679
H	-0.666650	-3.685411	-0.846573
C	2.721282	-2.702445	1.232304
H	3.494527	-1.956112	1.429176
H	2.903326	-3.535444	1.921245
H	2.865979	-3.091581	0.220297
H	-1.558883	2.495676	2.776389
H	-3.834418	3.560505	2.926372
H	-1.697431	3.800570	1.619274
C	-0.208362	-0.432479	-1.626901
C	0.917161	-0.697818	-2.585913
C	-1.563639	-0.994178	-1.876008
N	-0.455408	1.328630	-2.098148
O	1.532873	0.219013	-3.099861
O	-1.697068	-2.181092	-2.122205

O	-2.543188	-0.112038	-1.728361
N	-0.747629	2.315475	-2.522309
C	-3.904666	-0.606954	-1.924693
H	-4.041883	-0.769005	-2.997796
H	-3.991211	-1.576444	-1.425811
C	1.287166	-2.139139	-2.780648
H	2.056653	-2.199264	-3.549915
H	1.678807	-2.547929	-1.843236
H	0.417903	-2.744803	-3.046689
C	-4.834121	0.431275	-1.366659
H	-5.869485	0.126279	-1.531843
H	-4.686282	0.560784	-0.290859
H	-4.685786	1.399159	-1.851669

TS2$_S$-a

C	-2.450831	-1.515302	0.201040
C	-3.634563	-2.208773	-0.013743
C	-4.507145	-1.762778	-0.997505
C	-4.159015	-0.643138	-1.750317
C	-2.960713	0.003604	-1.480939
H	-3.822385	-3.091703	0.588839
H	-5.438611	-2.286052	-1.188834
H	-4.798662	-0.274633	-2.544764
H	-2.640142	0.875222	-2.045761
N	-2.129163	-0.420611	-0.516906
C	-1.396763	-2.038223	1.123595
O	-1.508999	-3.133252	1.653615
Rh	-0.360400	0.560631	0.169922
C	0.964541	2.244985	0.711154
C	0.241047	1.829177	1.878796
C	-1.163400	2.018139	1.626865
C	0.005457	2.732636	-0.263029
C	-1.290087	2.614371	0.319712
C	-2.271316	1.743018	2.580376
H	-2.483343	2.616960	3.206714
H	-3.197352	1.486365	2.057892
H	-2.025557	0.913817	3.248790
C	0.822123	1.296495	3.137472
H	0.710941	2.029175	3.944149
H	0.315362	0.380537	3.457274
H	1.888207	1.081913	3.036779
C	2.441027	2.363375	0.593132
H	2.759120	2.384089	-0.452003
H	2.772419	3.303322	1.048805
H	2.965576	1.549868	1.101168
C	0.338332	3.335856	-1.580904
H	-0.518181	3.316774	-2.260121
H	0.636641	4.384170	-1.464078
H	1.161051	2.809530	-2.072099
C	-2.569191	3.065785	-0.282450
H	-3.407450	2.419284	-0.009558
H	-2.810682	4.069130	0.087023
H	-2.517726	3.133365	-1.371548
N	-0.316800	-1.188667	1.236914
C	1.010737	-1.693699	1.238012
H	1.308827	-1.596145	0.096225
H	1.698186	-0.935312	1.644228
C	1.356634	-3.076792	1.729692
C	2.836999	-3.354193	1.530350
H	3.100320	-3.352557	0.467238
H	3.461294	-2.601269	2.025043
H	1.080449	-3.143601	2.789304
H	3.112153	-4.329823	1.934885
H	0.739434	-3.821814	1.221341
C	0.889921	-0.482701	-1.067645
C	0.207062	-1.454014	-1.973557
C	2.219456	0.015507	-1.465701
O	-0.192758	-2.530736	-1.561847
O	2.323264	0.777080	-2.415663
O	3.224423	-0.429744	-0.715592
C	4.557753	0.015655	-1.109256
H	4.741097	-0.334561	-2.129180
H	4.560592	1.110230	-1.131870
C	-0.009875	-0.975982	-3.384499
H	0.931592	-1.027660	-3.940323
H	-0.750010	-1.616469	-3.866244
H	-0.320437	0.072425	-3.418155
C	5.524857	-0.551546	-0.111137
H	6.541902	-0.246931	-0.366239
H	5.308690	-0.193422	0.899236
H	5.490710	-1.643233	-0.101873

TS2$_S$-b

C	-2.559911	1.382795	0.529715

C	-3.626008	2.010488	1.162637
C	-4.334949	1.315358	2.130155
C	-3.962778	0.005650	2.433402
C	-2.908854	-0.568625	1.740643
H	-3.843867	3.038148	0.891960
H	-5.160852	1.786368	2.653344
H	-4.476572	-0.566327	3.197591
H	-2.581763	-1.585943	1.938784
N	-2.225605	0.104578	0.799701
C	-1.705430	2.133891	-0.425681
O	-1.969834	3.264989	-0.787724
Rh	-0.566872	-0.632929	-0.282773
C	0.973273	-2.033064	-0.964471
C	0.286979	-1.495337	-2.087432
C	-1.124128	-1.799081	-1.920300
C	0.011757	-2.753496	-0.127379
C	-1.264022	-2.641949	-0.733635
C	-2.208866	-1.444939	-2.865320
H	-2.323783	-2.230838	-3.621800
H	-3.169932	-1.338101	-2.356644
H	-1.995156	-0.512294	-3.392230
C	0.886389	-0.767758	-3.233334
H	0.998413	-1.449974	-4.083664
H	0.259734	0.063225	-3.567869
H	1.880441	-0.378936	-2.998939
C	2.426244	-1.936543	-0.699949
H	2.644654	-1.987560	0.369123
H	2.946559	-2.772444	-1.183496
H	2.860398	-1.008123	-1.083913
C	0.349842	-3.503438	1.108938
H	-0.497546	-3.565554	1.795753
H	0.648022	-4.528886	0.862231
H	1.183721	-3.046340	1.647478
C	-2.531587	-3.270767	-0.288497
H	-3.385806	-2.596259	-0.392430
H	-2.741341	-4.149404	-0.908949
H	-2.482999	-3.613758	0.747016
N	-0.600540	1.375634	-0.861525
C	0.529218	1.948716	-1.257615
H	1.803329	2.852833	0.548327
H	1.309935	1.272363	-1.604037
C	0.704145	3.390859	-1.596202
C	2.113526	3.733810	-2.040310
H	2.854575	3.522084	-1.266228
H	2.406031	3.151503	-2.919725
H	-0.023535	3.613561	-2.388527
H	2.180766	4.790129	-2.305929
H	0.360052	4.008469	-0.758836
C	1.809455	1.812850	0.850322
C	0.747362	1.415877	1.712560
C	3.036348	1.108555	0.536091
O	-0.271404	2.128748	1.833823
O	3.698079	1.291442	-0.481113
O	3.403404	0.193486	1.478350
C	4.684991	-0.432703	1.273988
H	4.740930	-0.829407	0.253765
H	5.462879	0.333909	1.352275
C	0.769135	0.060206	2.407081
H	1.514257	0.049096	3.208742
H	-0.214013	-0.148025	2.835965
H	1.083564	-0.759594	1.743283
C	4.827056	-1.501355	2.323223
H	5.790086	-2.007334	2.226890
H	4.764644	-1.077379	3.328189
H	4.039351	-2.258229	2.231602

$TS2_T$-a

C	-0.843587	-2.336498	0.669223
C	-0.028166	-1.906075	1.768811
C	1.355735	-2.082925	1.400480
C	0.032054	-2.820022	-0.370755
C	1.387685	-2.653553	0.085086
C	2.523992	-1.768922	2.262035
H	2.747462	-2.604977	2.934792
H	3.424513	-1.577614	1.672940
H	2.334484	-0.891699	2.886559
C	-0.504872	-1.435127	3.091084
H	-1.559684	-1.154219	3.071669
H	-0.395479	-2.238338	3.829539
H	0.077019	-0.583055	3.452187
C	-2.323023	-2.480977	0.694131
H	-2.734473	-2.569085	-0.313502
H	-2.593986	-3.388849	1.244968
H	-2.813177	-1.639509	1.192920
C	-0.406618	-3.440514	-1.642886
H	0.405711	-3.504228	-2.369558

H	-0.762946	-4.461856	-1.460354
H	-1.229595	-2.879654	-2.093557
C	2.610953	-3.082856	-0.639686
H	3.468065	-2.441688	-0.417216
H	2.887842	-4.099686	-0.338783
H	2.465945	-3.100707	-1.722560
C	2.530850	1.495952	0.120513
C	3.679612	2.227032	-0.149439
C	4.464550	1.868976	-1.237708
C	4.071211	0.792308	-2.028541
C	2.918995	0.095363	-1.689742
H	3.905551	3.074955	0.489017
H	5.363437	2.427835	-1.477804
H	4.642014	0.491590	-2.899809
H	2.567464	-0.747603	-2.278627
N	2.170912	0.437630	-0.630508
C	1.573376	1.933967	1.174145
O	1.785750	2.925963	1.862555
Rh	0.417796	-0.613298	0.008268
N	0.469822	1.125028	1.306177
C	-0.808047	1.654659	1.510493
H	-1.293972	1.312813	0.382636
H	-1.436677	1.007693	2.142120
C	-1.072688	3.126110	1.677743
C	-2.553938	3.424036	1.510540
H	-2.896314	3.185315	0.497236
H	-3.165335	2.837660	2.205165
H	-0.713281	3.446289	2.663867
H	-2.766846	4.478815	1.693684
H	-0.474572	3.691575	0.955872
C	-1.070761	0.566091	-0.833544
C	-0.653665	1.737349	-1.652717
C	-2.350204	-0.079170	-1.213329
O	0.318996	2.428946	-1.368920
O	-2.462608	-0.926392	-2.085056
O	-3.379765	0.382314	-0.481530
C	-4.689907	-0.144239	-0.825449
H	-4.918589	0.165411	-1.850828
H	-4.637048	-1.237896	-0.823827
C	-1.543441	2.089514	-2.822580
H	-2.569048	2.264651	-2.479546
H	-1.166738	2.986534	-3.312054
H	-1.585667	1.260843	-3.535312
C	-5.665721	0.396485	0.179851
H	-6.673257	0.041911	-0.047526
H	-5.413195	0.069225	1.192235
H	-5.680822	1.488931	0.168773

TS2$_T$-b

C	-1.779381	0.223600	1.910323
C	-0.579970	0.860158	2.355244
C	0.360208	-0.178581	2.788112
C	-1.499078	-1.178415	1.839618
C	-0.202018	-1.422949	2.472600
C	1.684237	0.088905	3.403528
H	1.577414	0.361642	4.459825
H	2.345241	-0.779745	3.355719
H	2.196255	0.925172	2.916285
C	-0.416639	2.314530	2.621795
H	-0.953099	2.926649	1.891852
H	-0.809888	2.568401	3.613574
H	0.633520	2.619357	2.614796
C	-3.069023	0.889208	1.598572
H	-3.700626	0.266126	0.960846
H	-3.631611	1.078733	2.520704
H	-2.933802	1.854361	1.102217
C	-2.455126	-2.234330	1.410395
H	-1.945698	-3.175477	1.185217
H	-3.185960	-2.446560	2.199393
H	-3.008712	-1.928007	0.517213
C	0.400853	-2.765570	2.667974
H	1.470226	-2.708581	2.883494
H	-0.074274	-3.290962	3.504202
H	0.269014	-3.400710	1.785565
C	2.119973	-1.586446	-1.029286
C	2.839645	-2.562008	-1.710449
C	2.166710	-3.683795	-2.180278
C	0.796581	-3.786590	-1.956970
C	0.144582	-2.775429	-1.258098
H	3.904028	-2.411859	-1.858596
H	2.698952	-4.461812	-2.717500
H	0.229256	-4.636911	-2.319465
H	-0.925168	-2.798282	-1.070325
N	0.796368	-1.700087	-0.797951
C	2.795449	-0.344419	-0.566931
O	3.998087	-0.192580	-0.648683

Rh -0.062905 -0.183140 0.472317
N 1.893304 0.617519 -0.027650
C 2.249693 1.867076 0.092208
H -1.485856 2.719838 -0.740687
H 1.490149 2.503501 0.552374
C 3.525022 2.524387 -0.276240
C 3.438877 4.037534 -0.209994
H 2.696525 4.412866 -0.916389
H 3.159925 4.378902 0.791950
H 4.304561 2.128746 0.391053
H 4.402917 4.487863 -0.451326
H 3.836940 2.169469 -1.266108
C -1.135440 1.804004 -1.211896
C 0.114998 1.916895 -1.908481
C -2.108667 0.754268 -1.410103
O 0.851878 2.898531 -1.699105
O -1.830845 -0.460616 -1.367746
O -3.357041 1.196310 -1.571411
C -4.398112 0.198829 -1.722339
H -4.254219 -0.299128 -2.686657
H -4.270759 -0.563037 -0.943552
C 0.535152 0.887550 -2.928745
H 0.378555 1.310124 -3.926563
H 1.612293 0.711330 -2.845728
H -0.009213 -0.056116 -2.863670
C -5.717192 0.910240 -1.626233
H -6.536151 0.198696 -1.751399
H -5.832339 1.395197 -0.653400
H -5.810006 1.674424 -2.400391

TS2$_T$-c

C 0.072613 -2.455237 0.693575
C 1.239019 -2.277961 1.535774
C 2.380599 -2.220456 0.686793
C 0.513034 -2.619656 -0.661754
C 1.930848 -2.388641 -0.681103
C 3.793553 -2.033797 1.105334
H 4.349828 -1.416492 0.393223
H 3.872252 -1.562282 2.087340
H 4.311545 -2.998080 1.161065
C 1.215344 -2.149320 3.015235
H 2.117561 -1.669318 3.400744
H 0.354162 -1.567173 3.357055
H 1.140502 -3.134405 3.490948
C -1.315819 -2.666032 1.185512
H -2.059880 -2.360787 0.444428
H -1.482428 -3.727271 1.405099
H -1.506755 -2.120777 2.115342
C -0.339425 -2.976487 -1.823155
H 0.077878 -2.608378 -2.764577
H -0.415600 -4.067287 -1.913855
H -1.353965 -2.584159 -1.716527
C 2.806582 -2.495977 -1.881138
H 3.797120 -2.068523 -1.704399
H 2.961377 -3.545190 -2.157755
H 2.369951 -1.997090 -2.752371
C 1.875663 2.244410 0.323778
C 2.628587 3.409100 0.264355
C 3.778456 3.424920 -0.514280
C 4.136661 2.269581 -1.205467
C 3.346146 1.135410 -1.082956
H 2.286519 4.271646 0.826413
H 4.385111 4.321539 -0.586136
H 5.018652 2.240386 -1.835342
H 3.588916 0.209833 -1.598604
N 2.232228 1.118202 -0.332728
C 0.608922 2.207087 1.098809
O 0.249839 3.125428 1.809108
Rh 1.011760 -0.550750 0.079809
N -0.094553 0.981710 0.930938
C -1.411464 0.921654 1.174662
H -1.806767 -0.091427 1.070321
C -2.184398 1.832880 2.083298
C -3.632531 1.408470 2.236019
H -4.201868 1.559546 1.315189
H -3.714914 0.349798 2.508679
H -1.666573 1.808385 3.052068
H -4.121506 1.986683 3.021929
H -2.092355 2.875077 1.766361
H -2.683560 2.373862 -0.452947
C -2.269436 1.430320 -0.803133
C -1.063049 1.588785 -1.645902
C -3.223923 0.326132 -1.001417
O -0.328134 2.551700 -1.441020
O -2.897546 -0.844640 -1.151407
O -4.492042 0.756110 -0.964549

C	-5.514481	-0.271727	-1.091033
H	-6.387004	0.271385	-1.456039
H	-5.189331	-0.986683	-1.850642
C	-0.772944	0.607170	-2.747754
H	-1.423932	0.816597	-3.604778
H	0.263624	0.723795	-3.071545
H	-0.978063	-0.420552	-2.444578
C	-5.770858	-0.941599	0.234209
H	-6.564010	-1.685308	0.126061
H	-4.874942	-1.457055	0.592170
H	-6.087599	-0.218891	0.990169

TS3

C	-1.414834	2.206920	-0.143370
C	-2.279700	3.282562	0.094440
C	-3.488352	3.065646	0.723965
C	-3.822847	1.761300	1.119428
C	-2.922351	0.742156	0.869456
H	-1.943817	4.259280	-0.240185
H	-4.170847	3.889123	0.914411
H	-4.761348	1.540577	1.616612
H	-3.123124	-0.283618	1.172152
N	-1.746491	0.947957	0.251466
C	-0.127107	2.416031	-0.822425
N	0.599727	1.260030	-0.946793
C	1.956437	1.214324	-1.046545
H	2.327196	0.462090	-1.758717
C	2.828849	2.427802	-0.862412
C	4.302376	2.069924	-0.966631
H	4.600843	1.346877	-0.198862
H	4.527682	1.620322	-1.940897
O	0.194834	3.532278	-1.268417
Rh	-0.263108	-0.489677	-0.038338
C	0.027838	-1.813115	-1.797210
C	-1.288416	-1.927925	-1.205378
C	-1.161496	-2.521534	0.116859
C	0.971034	-2.170140	-0.792100
C	0.228875	-2.604871	0.383142
H	2.559539	3.206878	-1.589043
H	2.619894	2.890132	0.114014
H	4.942227	2.950292	-0.859234
C	0.856708	-3.080224	1.645360
H	0.252192	-2.827016	2.521783
H	0.982644	-4.170113	1.636326
H	1.851062	-2.644397	1.782425
C	-2.269709	-2.978427	0.998056
H	-2.446062	-4.057634	0.900703
H	-2.061222	-2.779186	2.054219
H	-3.213618	-2.484040	0.747805
C	-2.568920	-1.661263	-1.915160
H	-2.920727	-2.557881	-2.440361
H	-3.359903	-1.356446	-1.223087
H	-2.454243	-0.866113	-2.656688
C	0.300811	-1.311385	-3.169652
H	-0.109952	-1.985765	-3.930860
H	-0.144711	-0.323094	-3.326732
H	1.372671	-1.210427	-3.355286
C	2.453029	-2.230904	-0.925928
H	2.804952	-3.269750	-0.930132
H	2.792421	-1.763181	-1.853109
H	2.962964	-1.716432	-0.104349
C	1.738895	0.289826	2.282491
O	0.543878	0.321039	1.962147
O	2.739961	0.320397	1.438274
H	2.390104	0.519364	0.454784
C	2.162607	0.200364	3.716306
H	1.294675	0.122626	4.369377
H	2.814316	-0.666271	3.856631
H	2.749639	1.081261	3.986627

VII. Reference

(1) Zhou, J.; Li, B.; Qian, Z. -C.; Shi, B. -F. *Adv. Synth. Catal.* **2014**, *356*, 1038.

(2) Andrea, O. P.; Alejandra, H. -S.; Diego, G. -S. *Green Chem*. **2015**, *17*, 3157.

(3) Jiang, Y.; Khong, V. Z. Y.; Lourdusamy, E.; Park, C. M. *Chem. Commun.* **2012**, *48*, 3133.

(4) Yang, C.M .; Pittman, C. U., Jr. *Synth. Comm*. **1998**, *28*, 2027–2041.

(5) Zhao, Y. S.; Chen, G. *Org. Lett.* **2011**, *13*, 4850-4853.

(6) Zhu, C. H.; Xu, G, Y.; Ding, D.; Qiu, L.; Sun, J. G. *Org. Lett*. **2015**, *17*, 4244-4247.

(7) Frisch, M. J. T., G. W.; Schlegel, H. B.; Scuseria, G. E.; Robb, M. A.; Cheeseman, J. R.; Scalmani, G.; Barone, V.; Mennucci, B.; Petersson, G. A.; Nakatsuji, H.; Caricato, M.; Li, X.; Hratchian, H. P.; Izmaylov, A. F.; Bloino, J.; Zheng, G.; Sonnenberg, J. L.; Hada, M.; Ehara, M.; Toyota, K.; Fukuda, R.; Hasegawa, J.; Ishida, M.; Nakajima,T.; Honda, Y.; Kitao, O.; Nakai, H.; Vreven, T.; Montgomery, J. A., Jr.; Peralta, J. E.; Ogliaro, F.; Bearpark, M.; Heyd, J. J.; Brothers, E.; Kudin,K. N.; Staroverov, V. N.; Keith, T.; Kobayashi, R.; Normand, J.;Raghavachari, K.; Rendell, A.; Burant, J. C.; Iyengar, S. S.; Tomasi, J.; Cossi, M.; Rega, N.; Millam, J. M.; Klene, M.; Knox, J. E.; Cross, J. B.; Bakken, V.; Adamo, C.; Jaramillo, J.; Gomperts, R.; Stratmann, R. E.;Yazyev, O.; Austin, A. J.; Cammi, R.; Pomelli, C.; Ochterski, J. W.; Martin, R. L.; Morokuma, K.; Zakrzewski, V. G.; Voth, G. A.; Salvador,; P.; Dannenberg, J. J. D., S.; Daniels, A. D.; Farkas, O.; Foresman, J. B.; Ortiz, J. V.; Cioslowski, J.; Fox, D. J. Gaussian 09, Revision D.01; Gaussian, Inc.: Wallingford CT **2013**.

(8) Zhao, Y.; Truhlar, D. G. *Theor. Chem. Acc.* **2008**, *120*, 215-241.

(9) Dolg, M.; Wedig, U.; Stoll, H.; Preuss, H. *J. Chem. Phys.* **1987**, *86*, 866-872.

(10) Marenich, A. V.; Cramer, C. J.; Truhlar, D. G. *J. Phys. Chem. B* **2009**, *113*, 6378-6396.

(11) Martin, R. L.; Hay, P. J.; Pratt, L. R. *J. Phys. Chem. A* **1998**, *102*, 3565-3573.

(12) C. Y. Legault *CYLview, 1.0b* **2009**, *Université de Sherbrooke*, http://www.cylview.org.

Appendix II: Spectral copies of ^{1}H and ^{13}C NMR of compounds obtained in this study

^{1}H NMR spectrum (400 MHz, $CDCl_3$) of **1a**

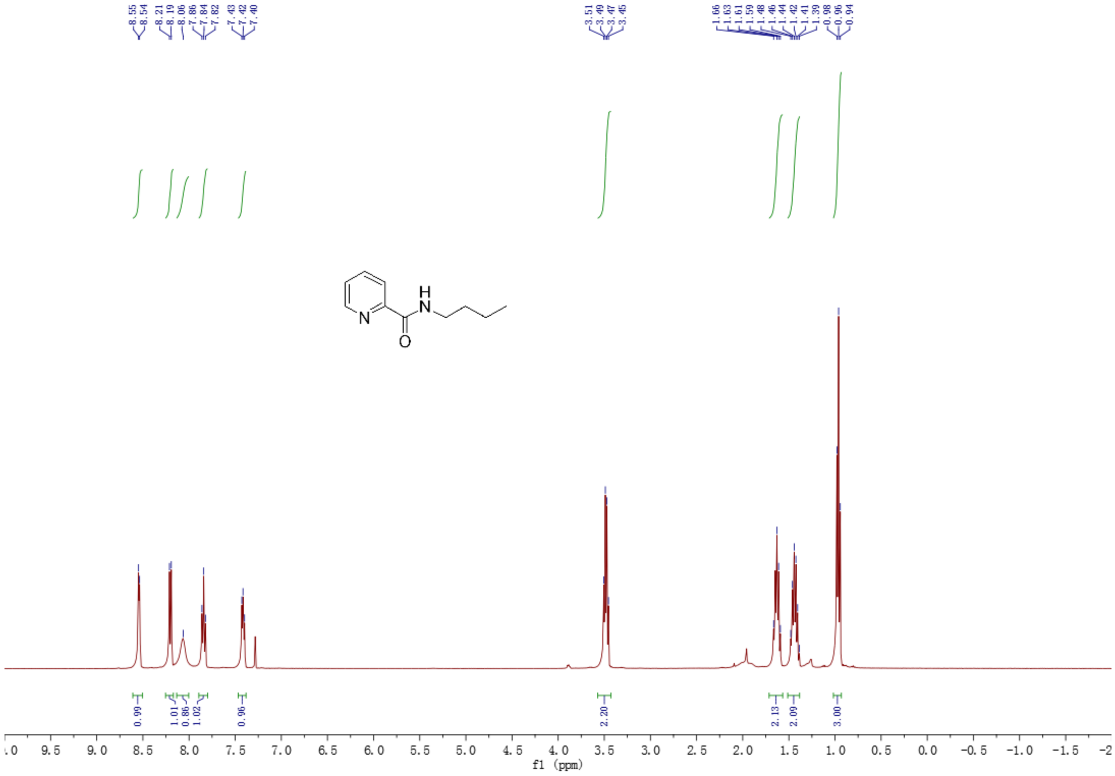

^{13}C NMR spectrum (100 MHz, $CDCl_3$) of **1a**

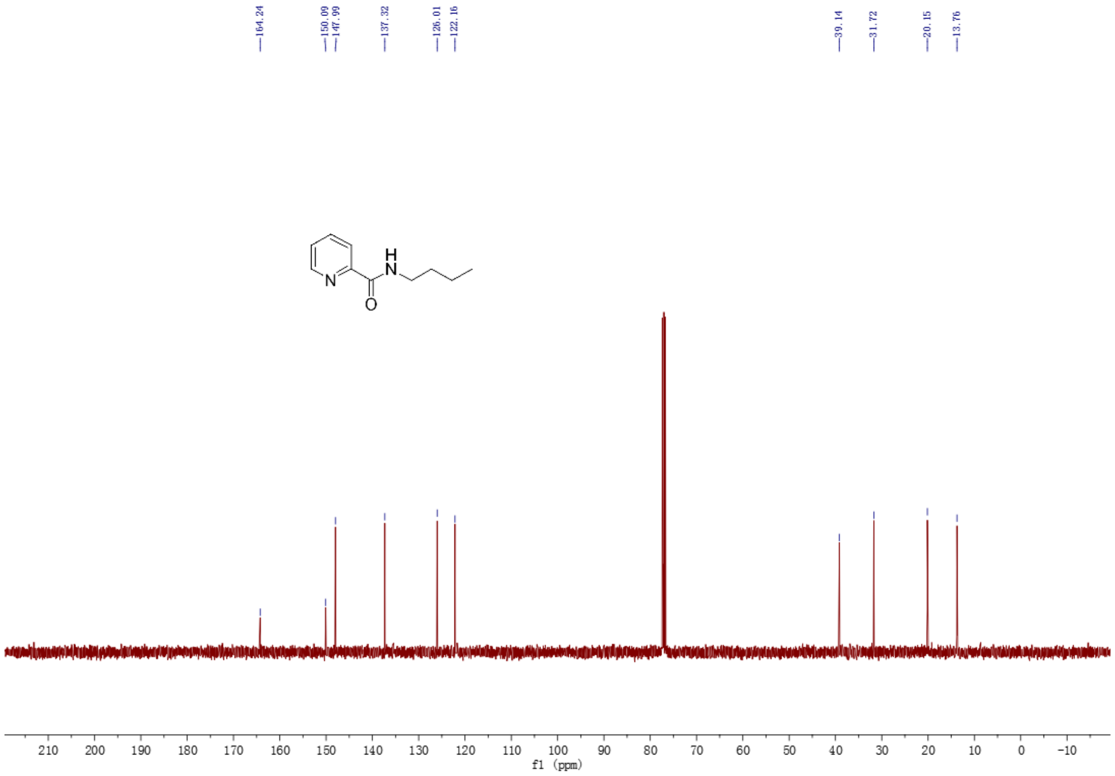

^{1}H NMR spectrum (400 MHz, $CDCl_3$) of **1b**

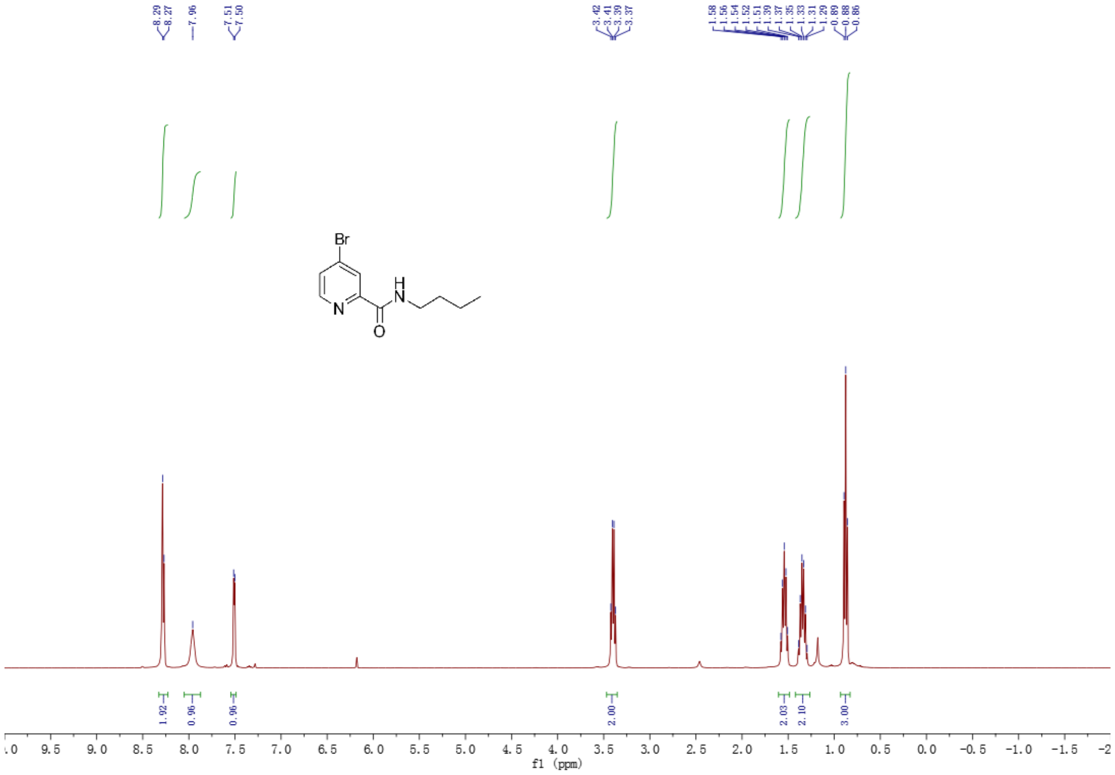

^{13}C NMR spectrum (100 MHz, $CDCl_3$) of **1b**

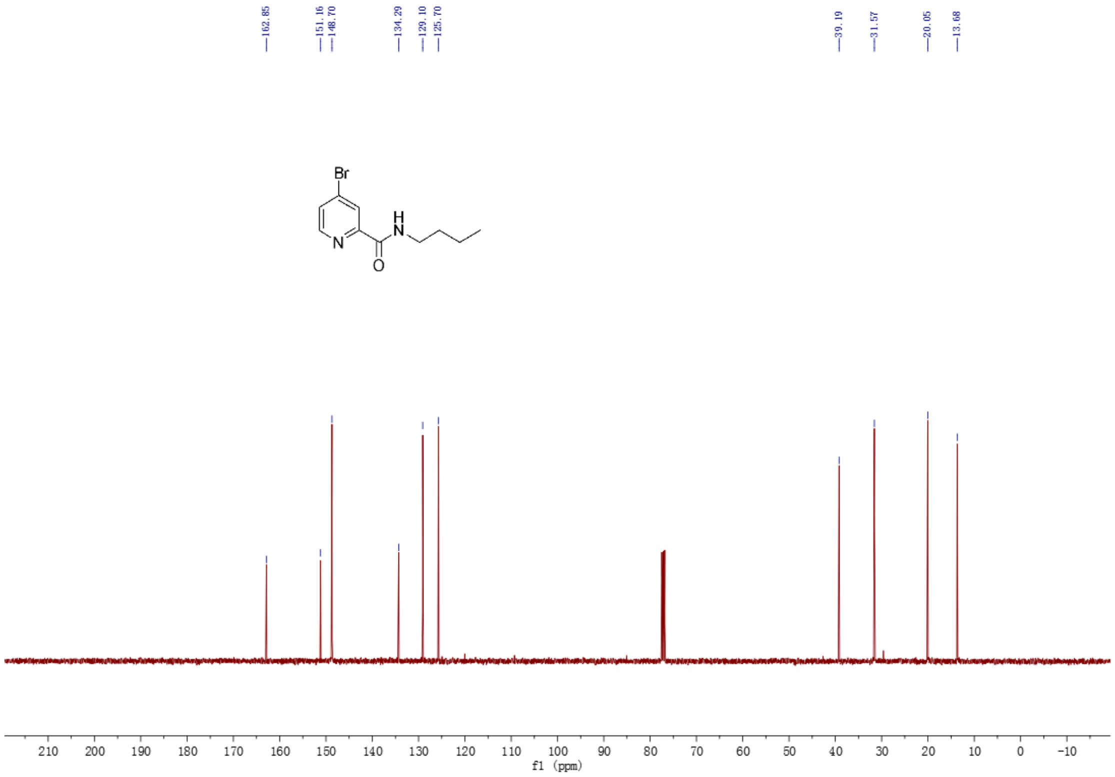

^{1}H NMR spectrum (400 MHz, $CDCl_3$) of **1c**

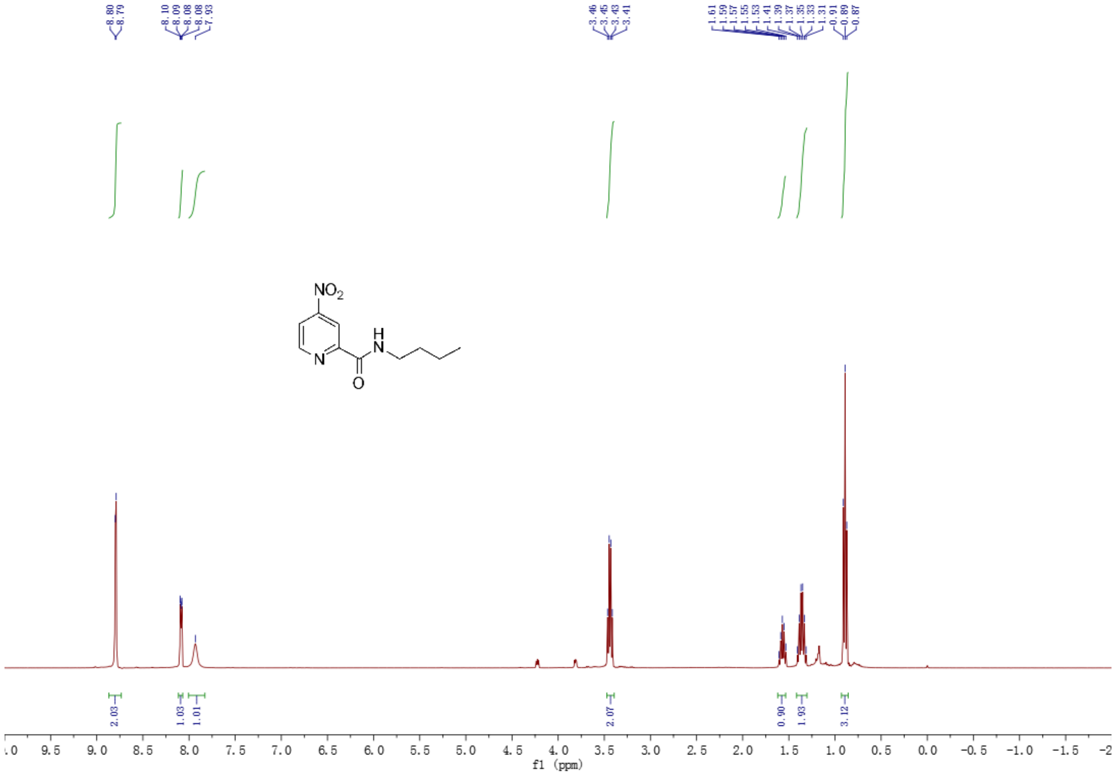

^{13}C NMR spectrum (100 MHz, $CDCl_3$) of **1c**

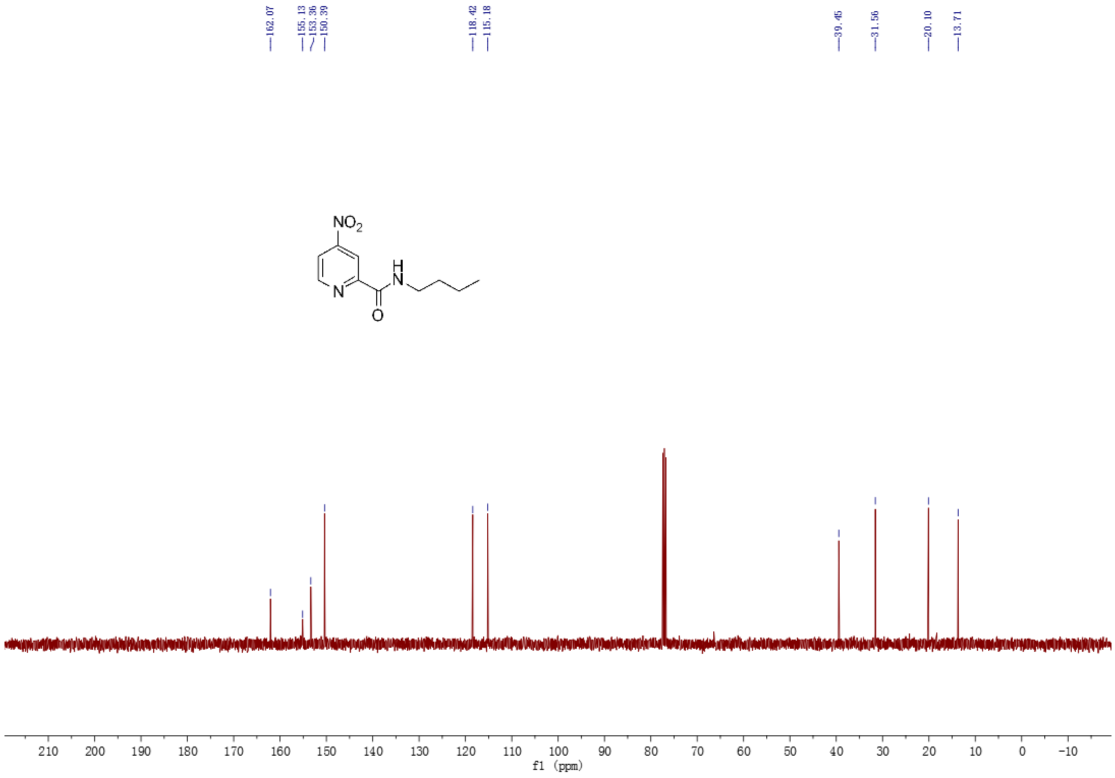

^{1}H NMR spectrum (400 MHz, $CDCl_3$) of **1d**

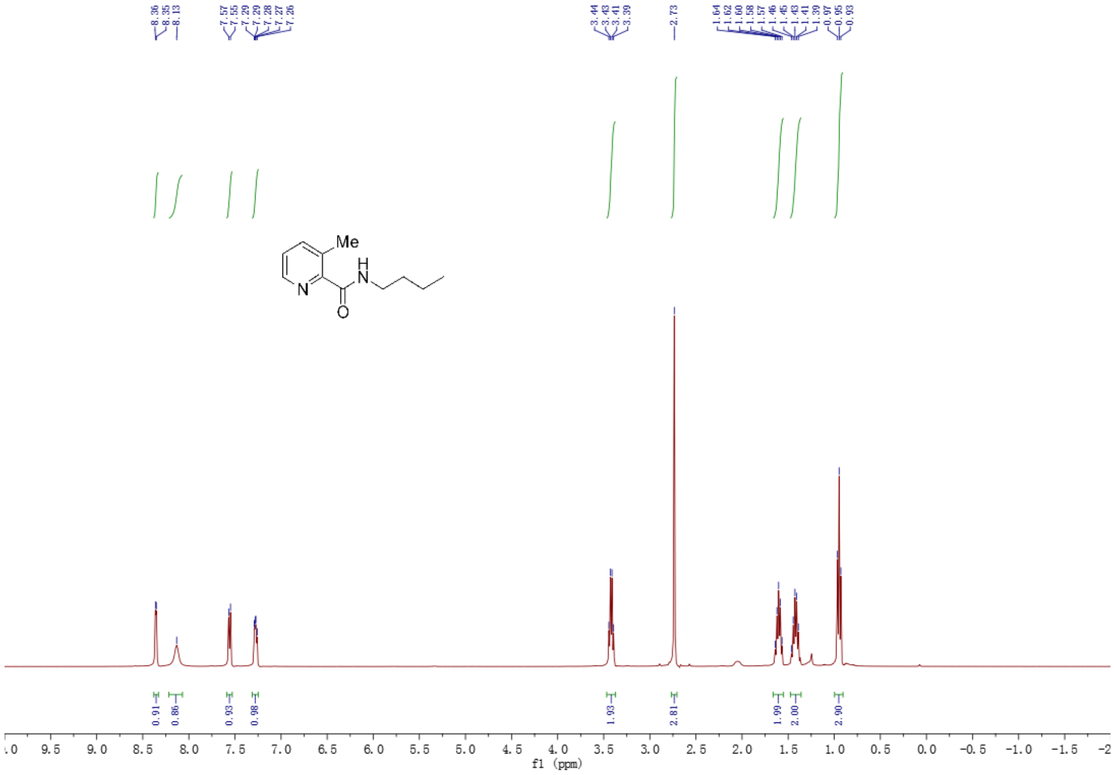

^{13}C NMR spectrum (100 MHz, $CDCl_3$) of **1d**

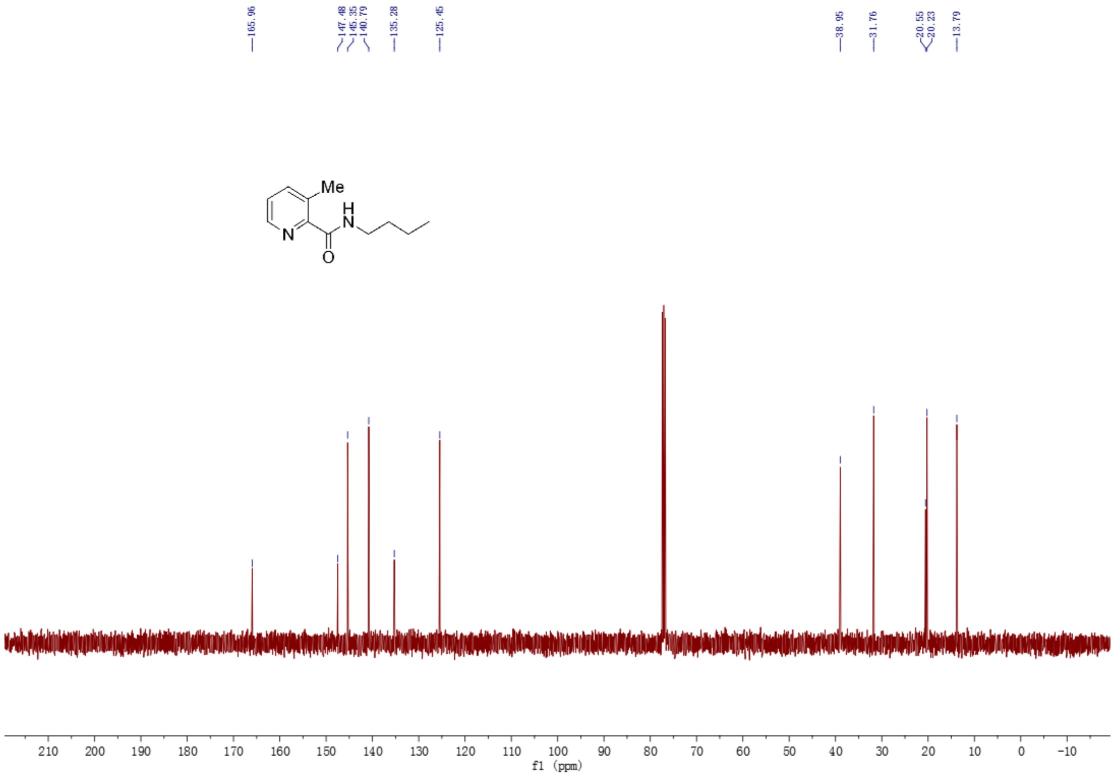

^{1}H NMR spectrum (400 MHz, $CDCl_3$) of **1e**

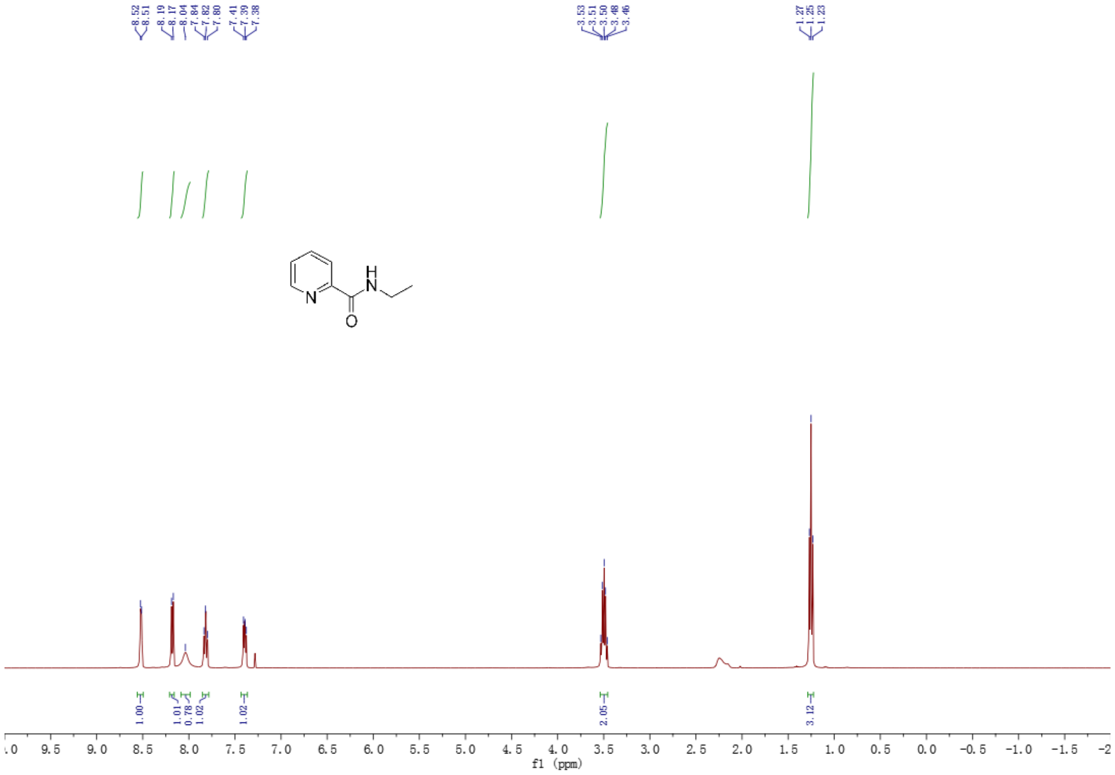

^{13}C NMR spectrum (100 MHz, $CDCl_3$) of **1e**

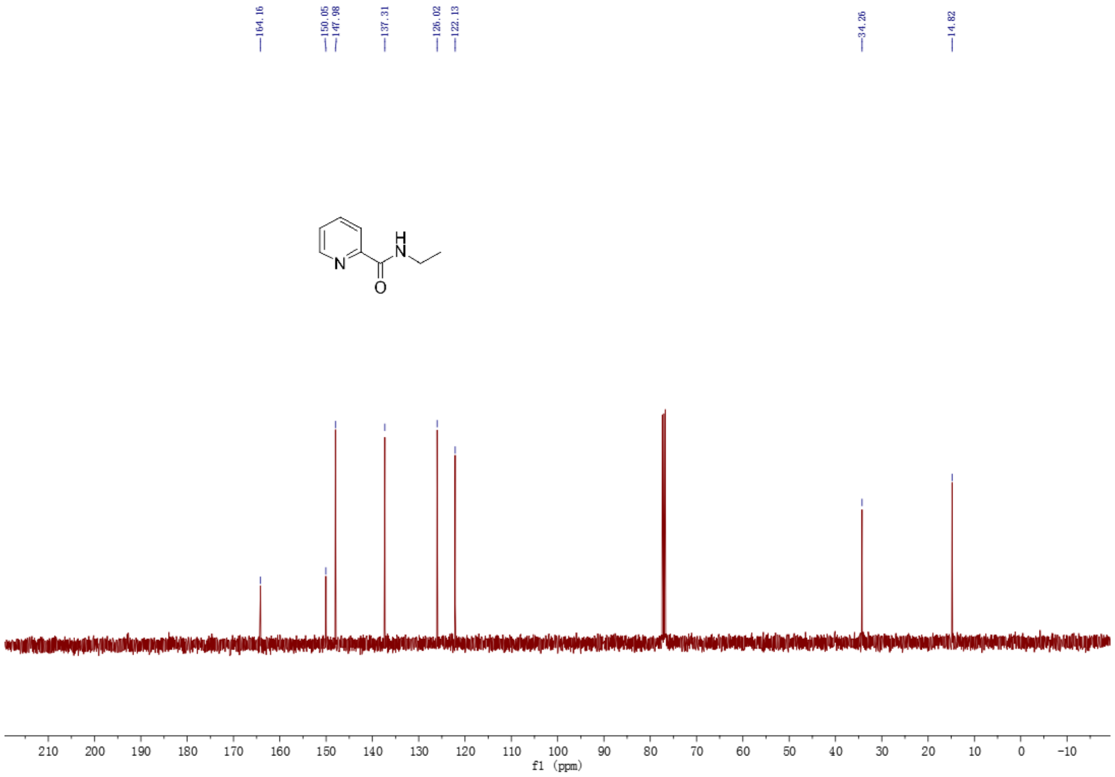

^{1}H NMR spectrum (400 MHz, $CDCl_3$) of **1f**

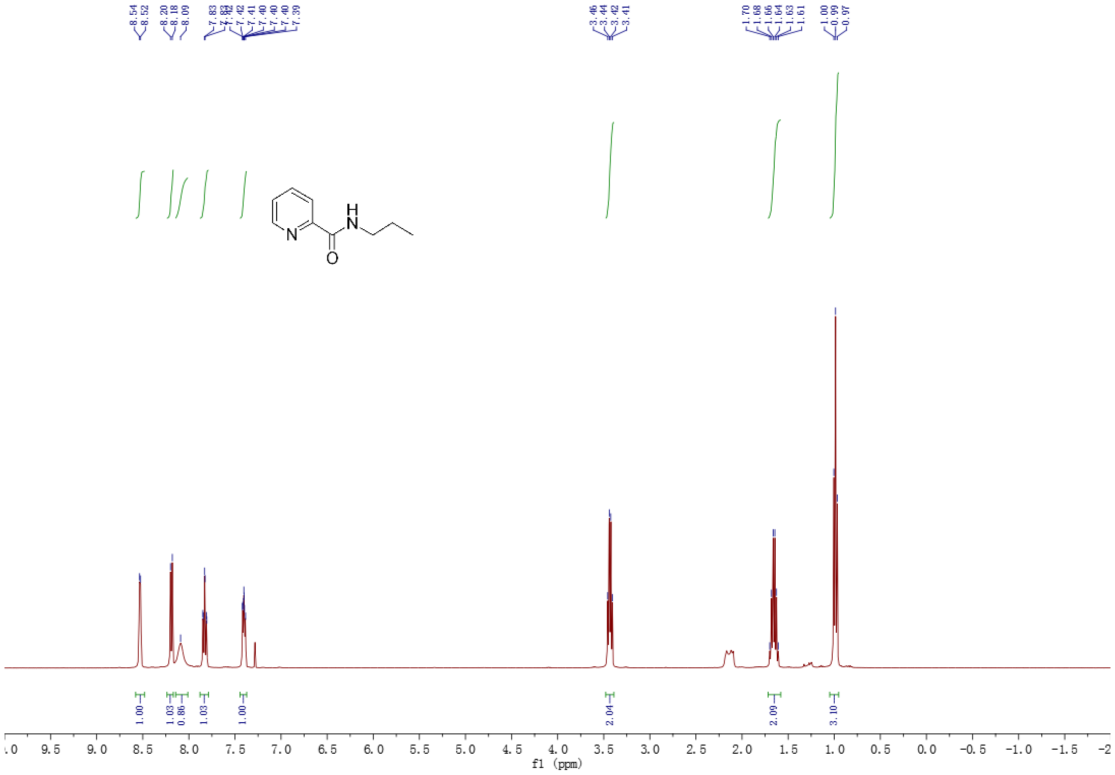

^{13}C NMR spectrum (100 MHz, $CDCl_3$) of **1f**

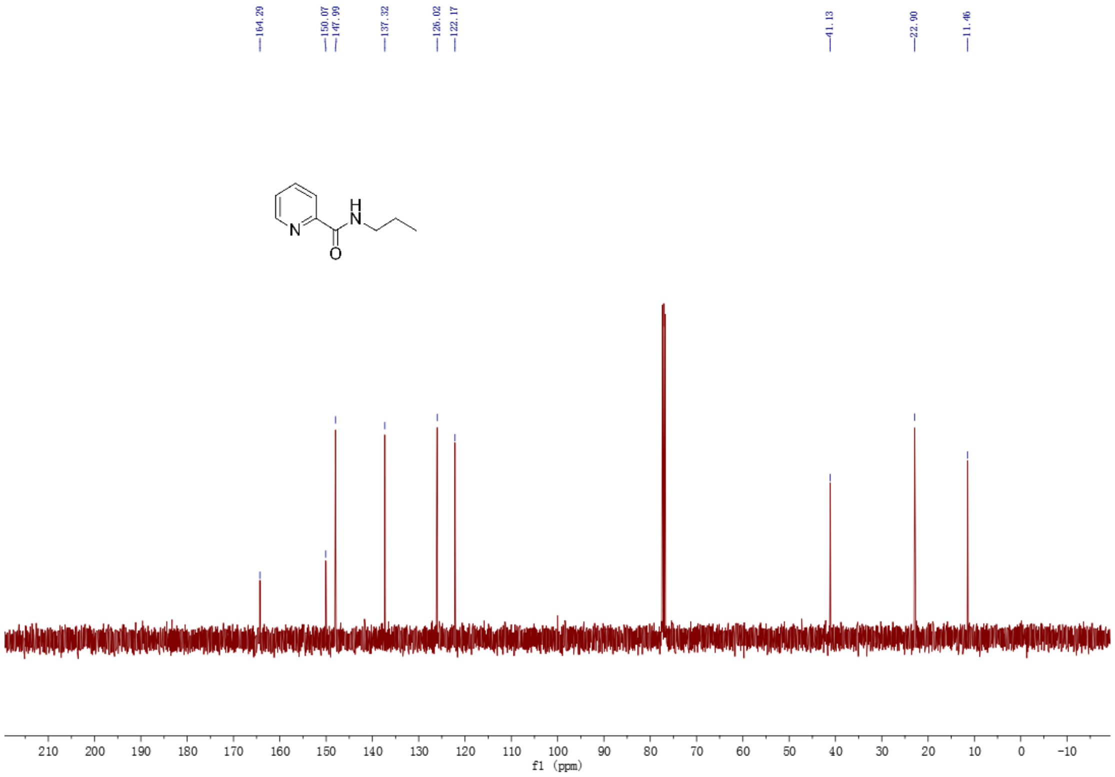

^{1}H NMR spectrum (400 MHz, $CDCl_3$) of **1g**

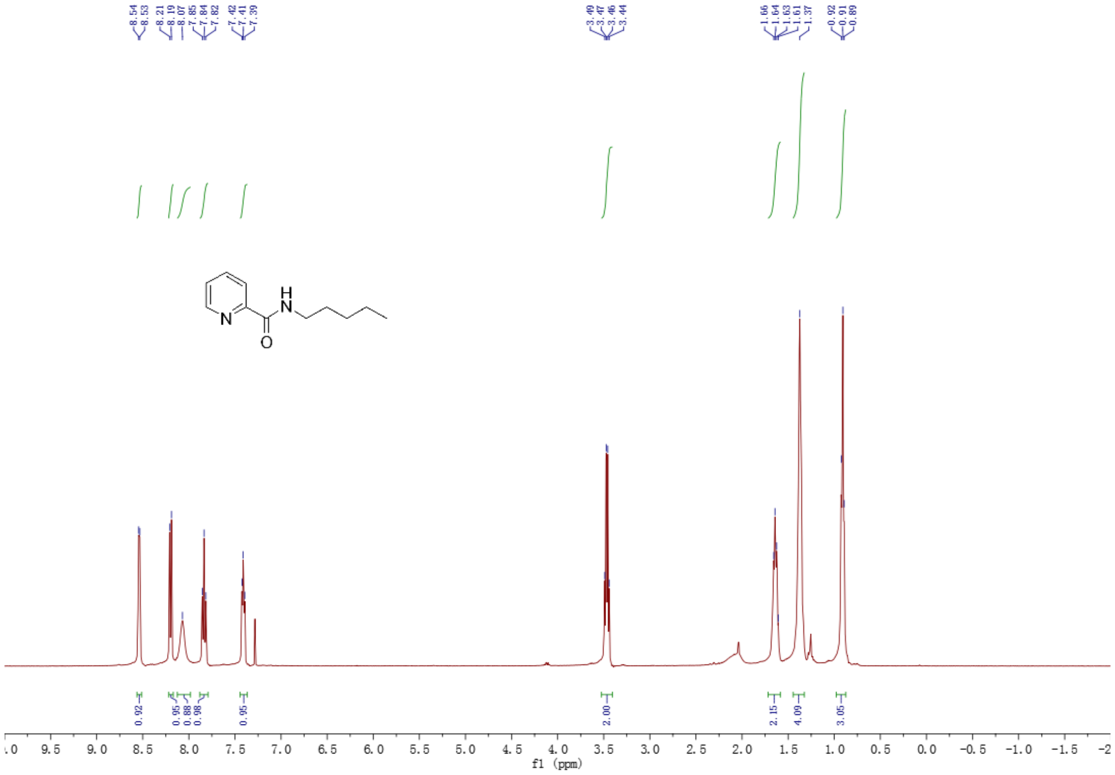

^{13}C NMR spectrum (100 MHz, $CDCl_3$) of **1g**

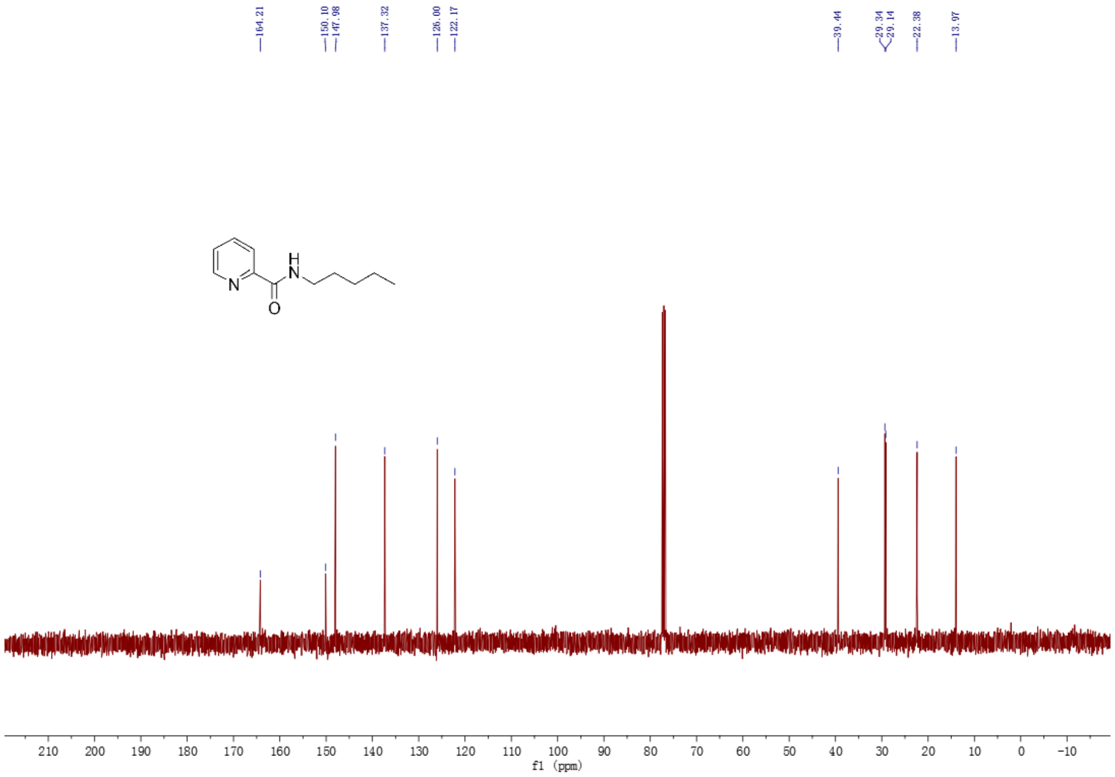

^{1}H NMR spectrum (400 MHz, $CDCl_3$) of **1h**

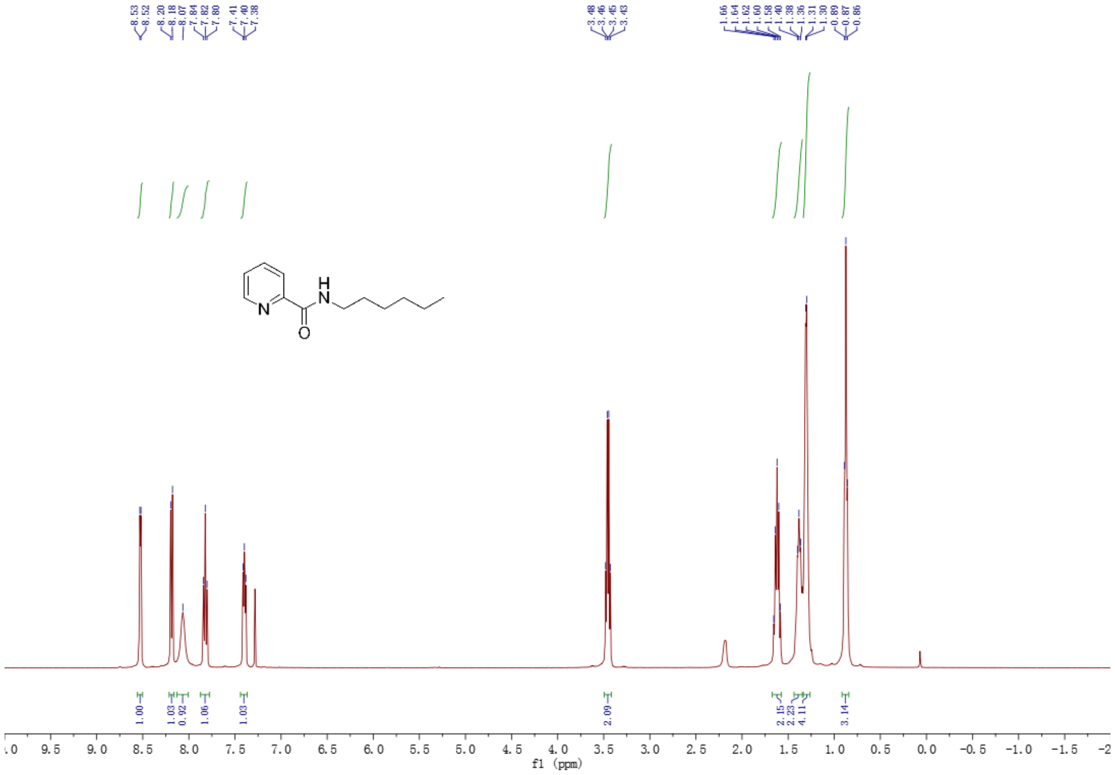

^{13}C NMR spectrum (100 MHz, $CDCl_3$) of **1h**

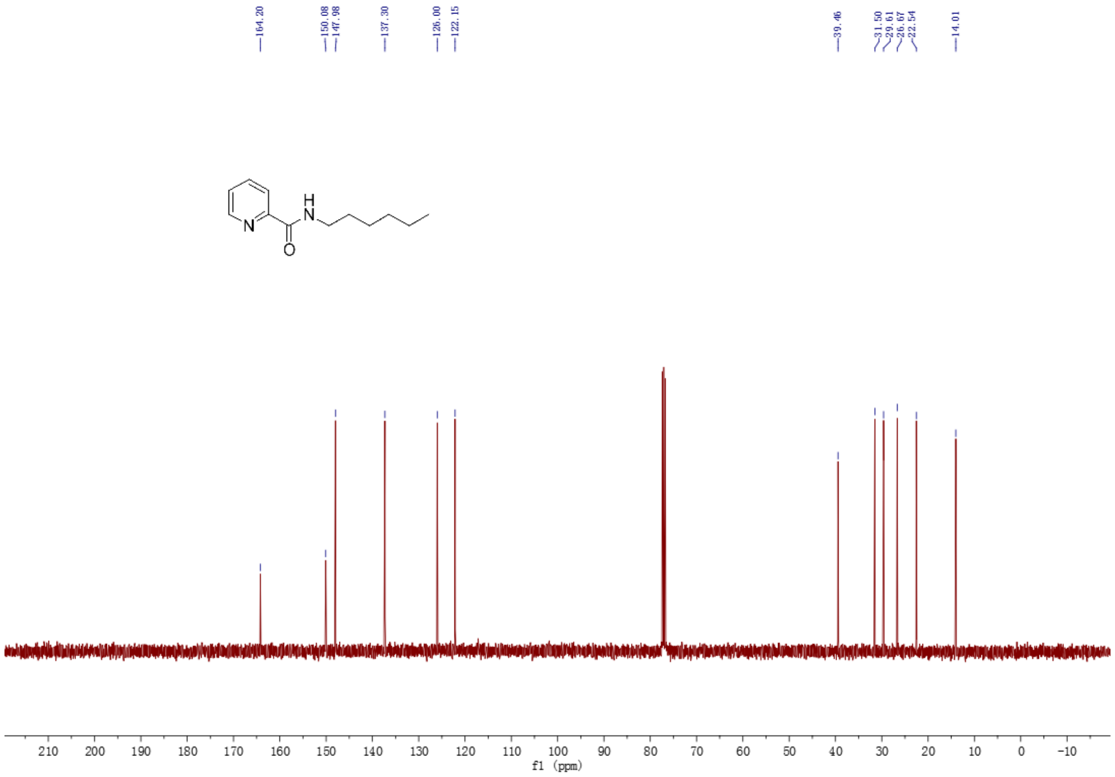

^{1}H NMR spectrum (400 MHz, $CDCl_3$) of **1i**

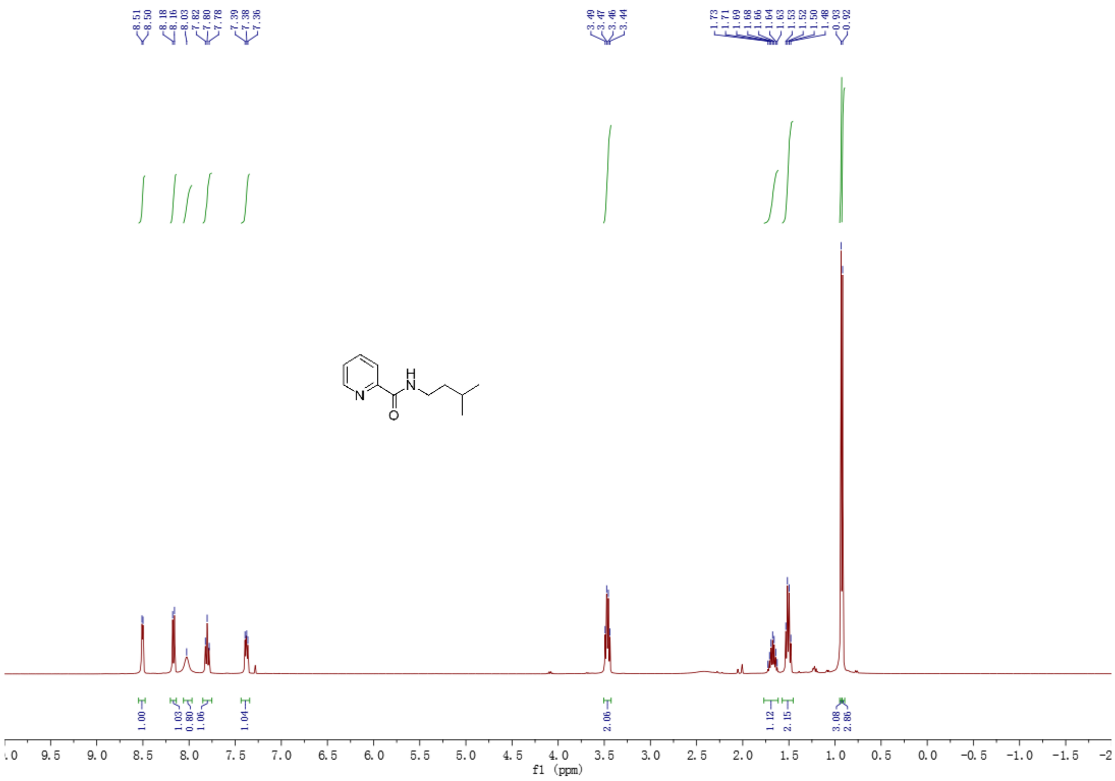

^{13}C NMR spectrum (100 MHz, $CDCl_3$) of **1i**

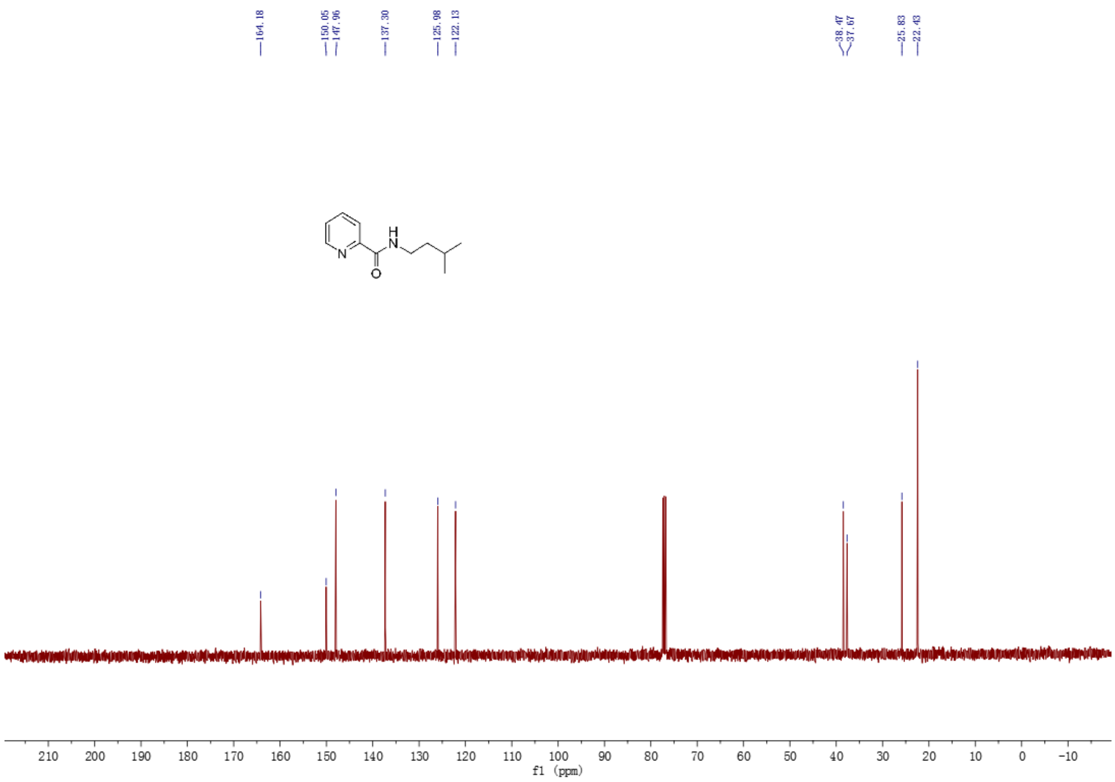

^{1}H NMR spectrum (400 MHz, $CDCl_3$) of **1j**

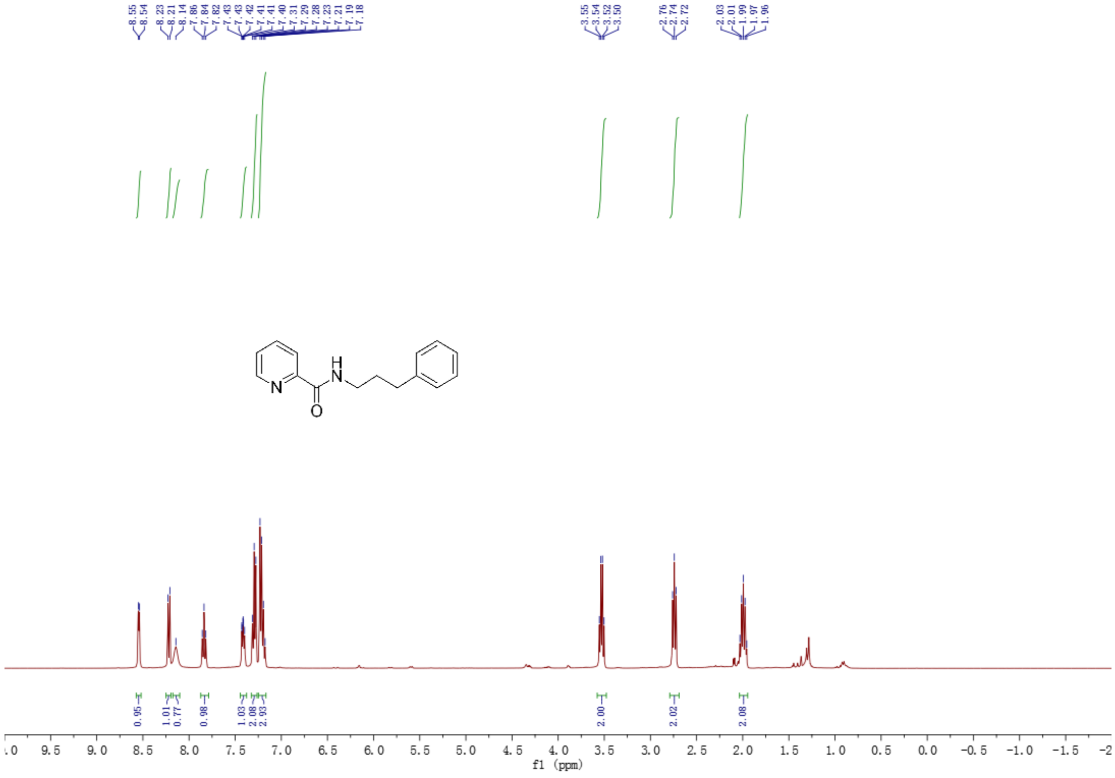

^{13}C NMR spectrum (100 MHz, $CDCl_3$) of **1j**

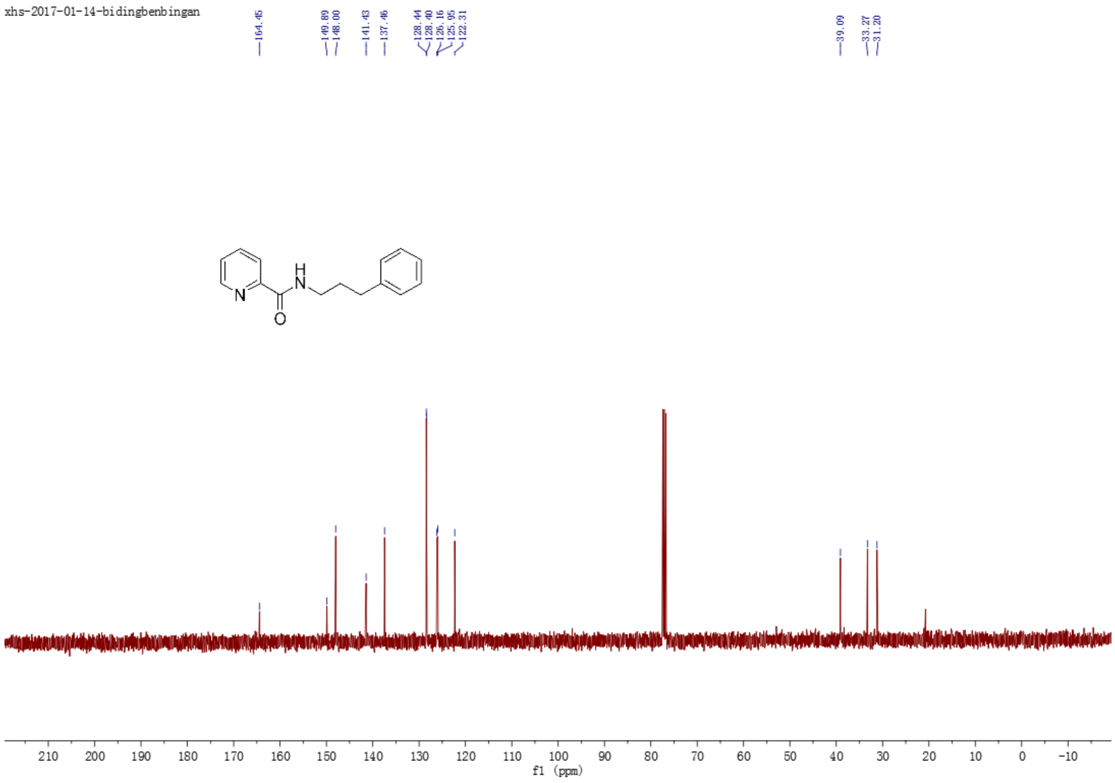

^{1}H NMR spectrum (400 MHz, $CDCl_3$) of **1k**

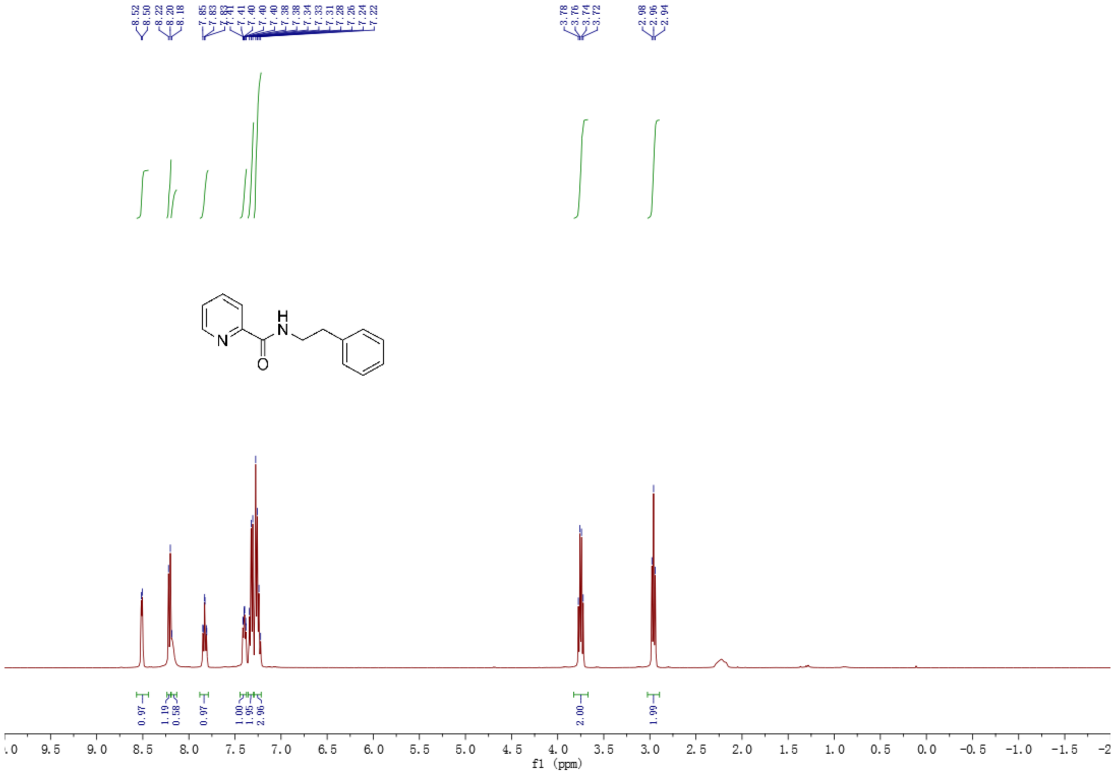

^{13}C NMR spectrum (100 MHz, $CDCl_3$) of **1k**

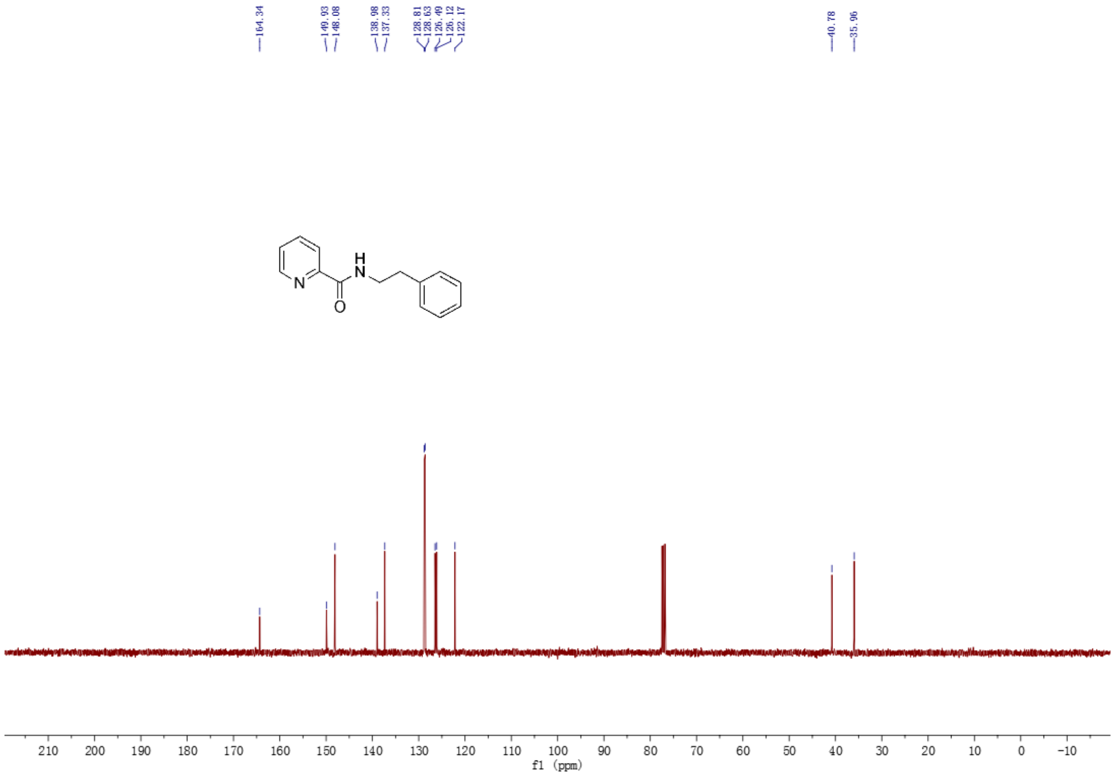

^{1}H NMR spectrum (400 MHz, $CDCl_3$) of **1l**

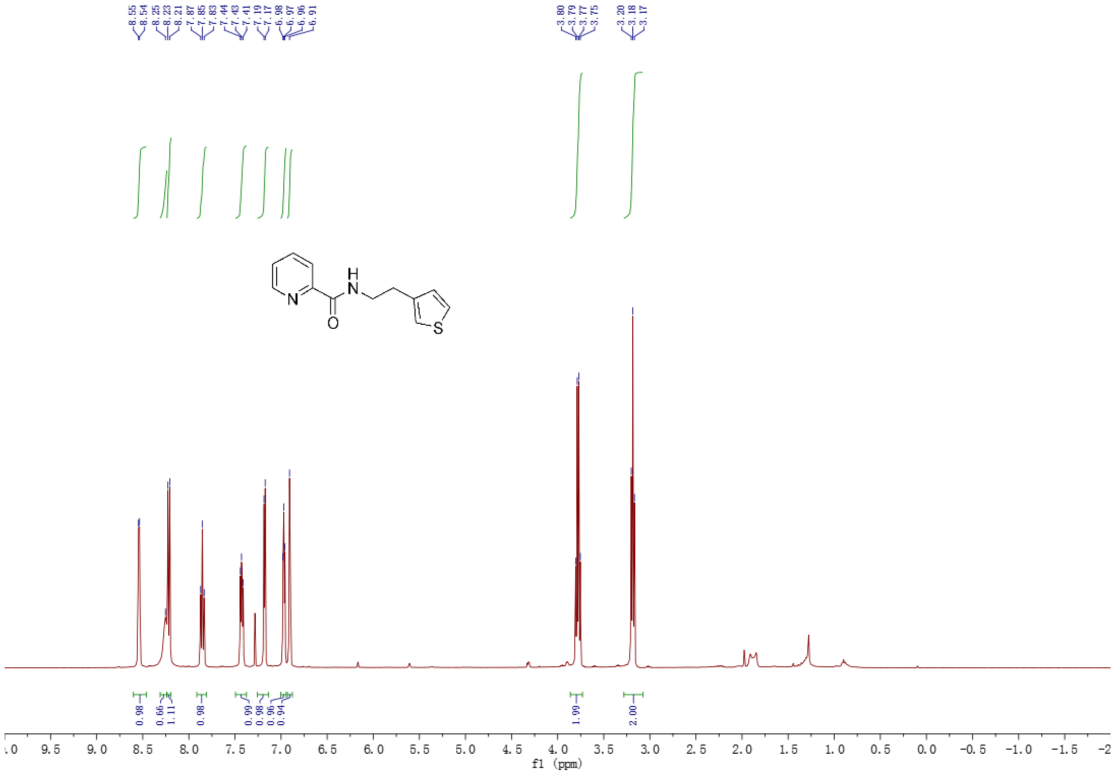

^{13}C NMR spectrum (100 MHz, $CDCl_3$) of **1l**

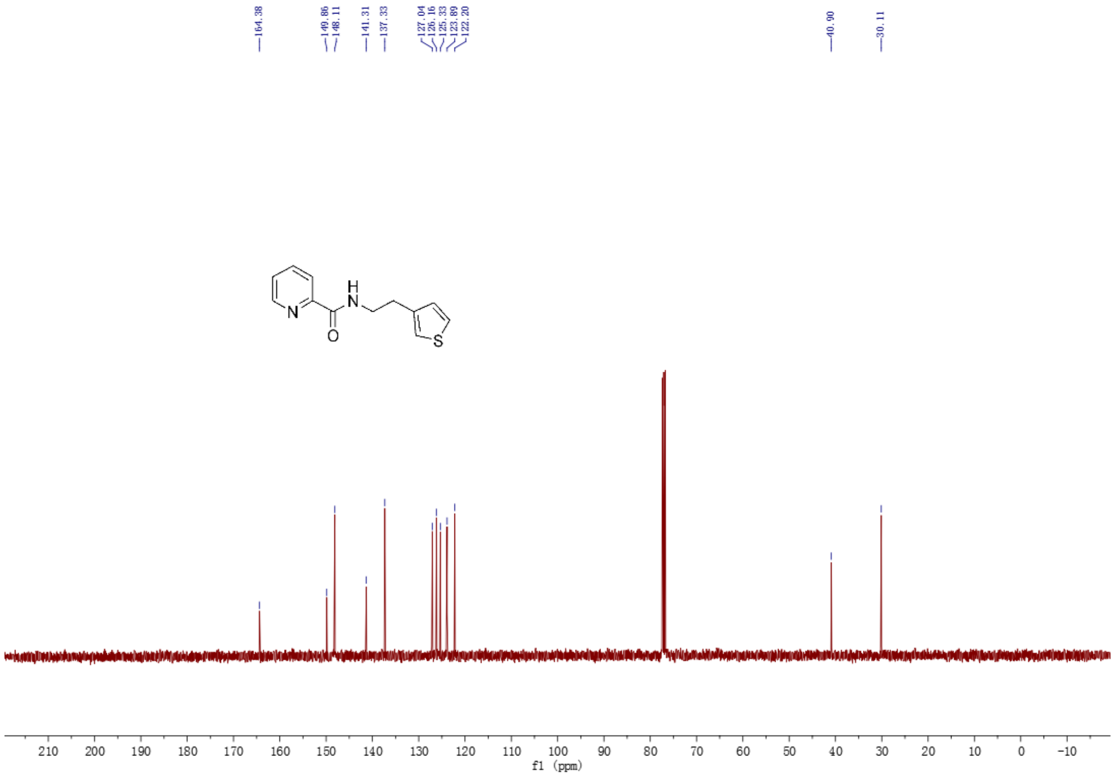

^{1}H NMR spectrum (400 MHz, $CDCl_3$) of **1m**

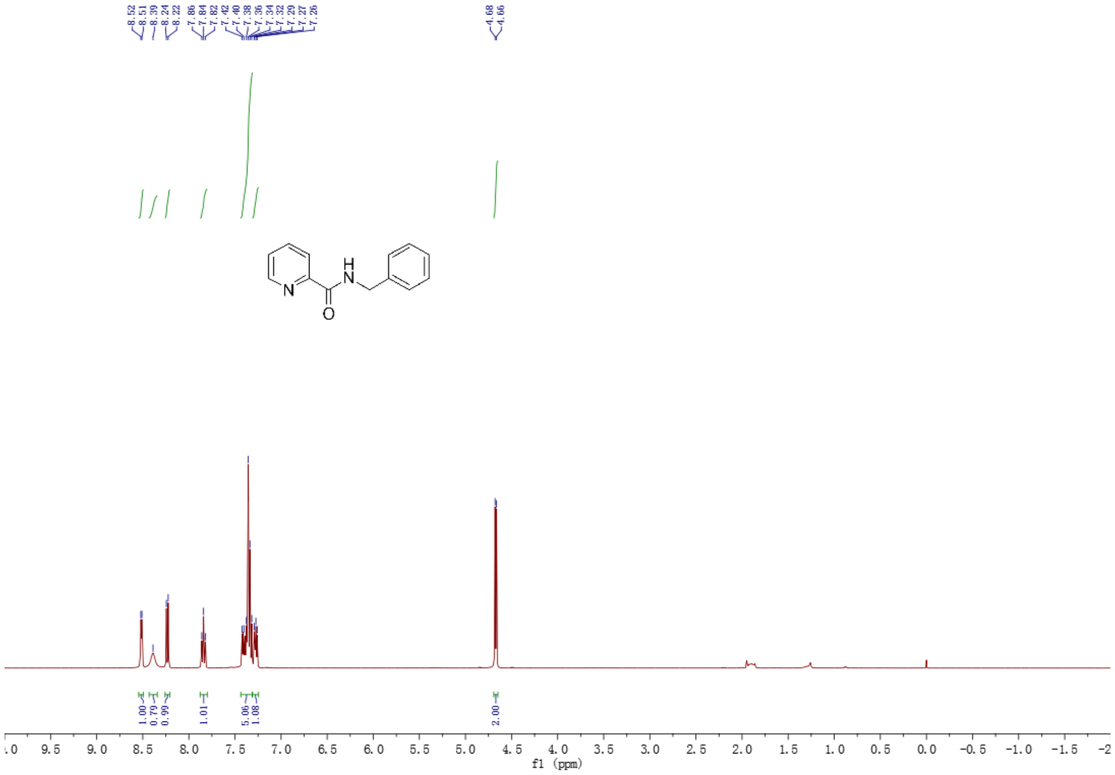

^{13}C NMR spectrum (100 MHz, $CDCl_3$) of **1m**

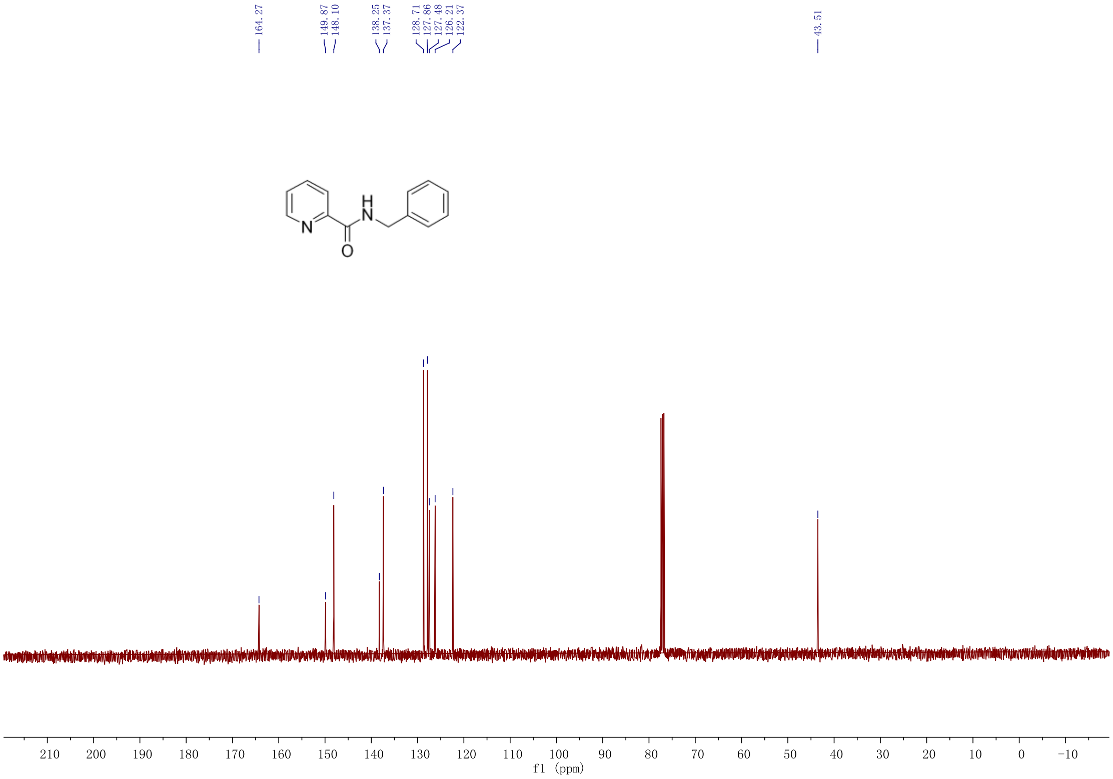

^{1}H NMR spectrum (400 MHz, $CDCl_3$) of **1n**

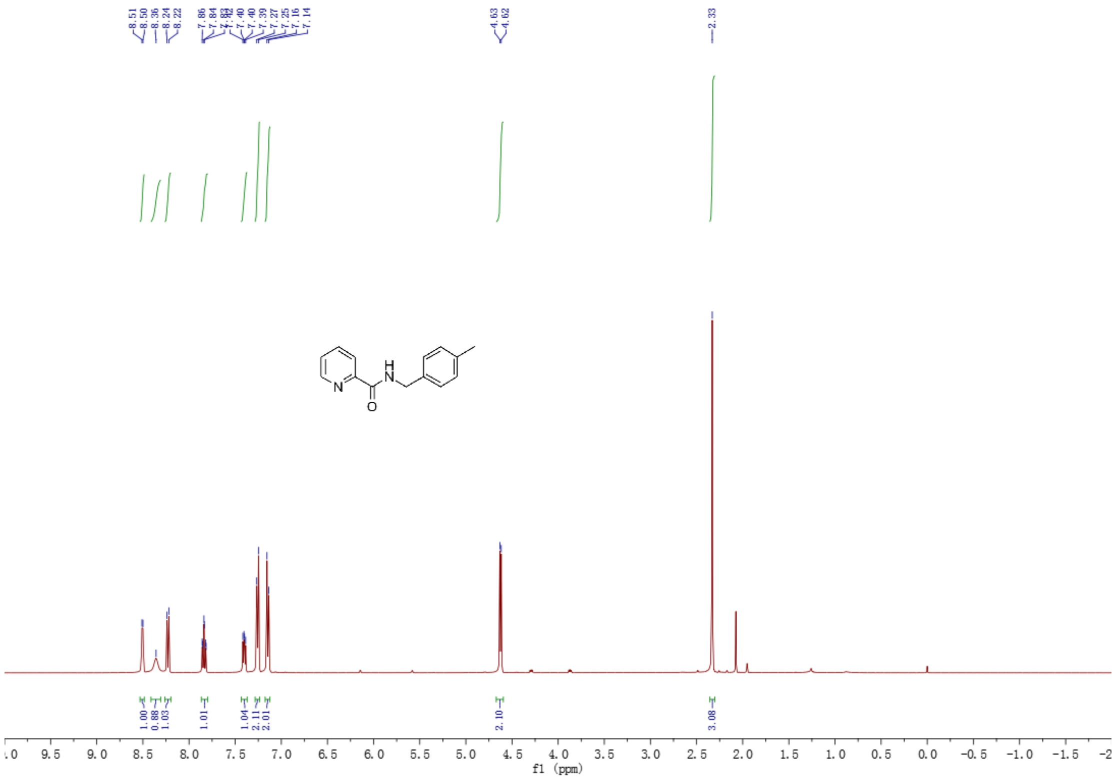

^{13}C NMR spectrum (100 MHz, $CDCl_3$) of **1n**

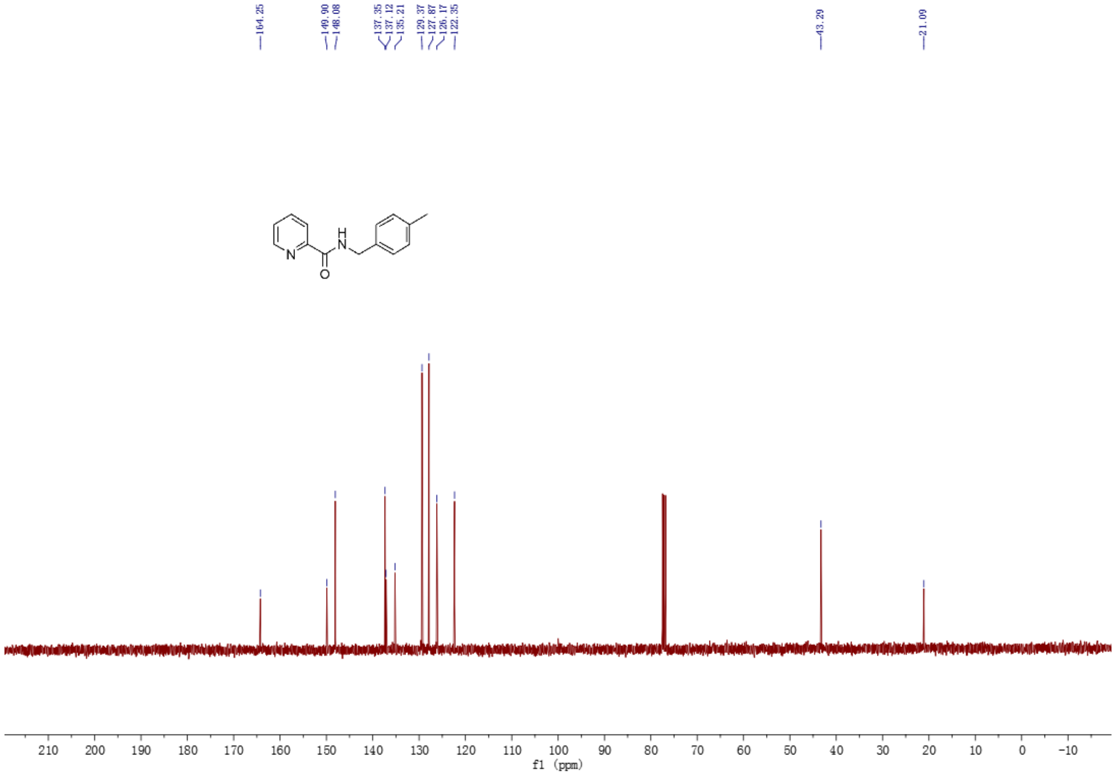

^{1}H NMR spectrum (400 MHz, $CDCl_3$) of **1o**

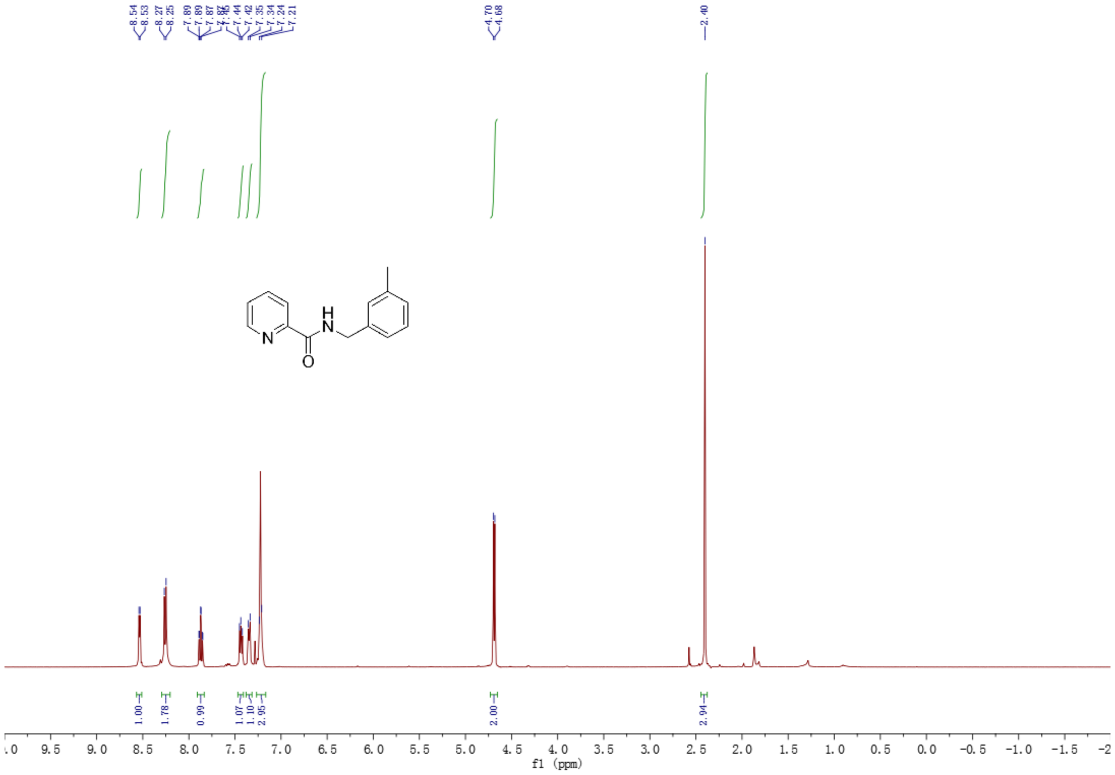

^{13}C NMR spectrum (100 MHz, $CDCl_3$) of **1o**

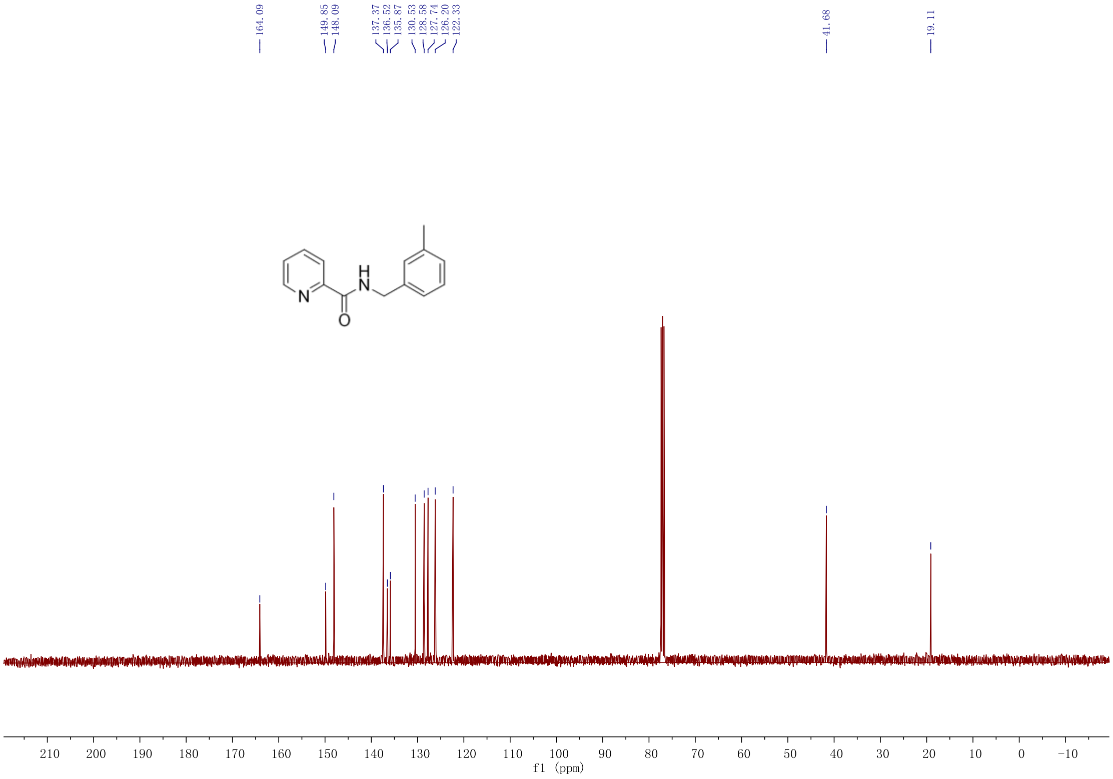

^{1}H NMR spectrum (400 MHz, $CDCl_3$) of **1p**

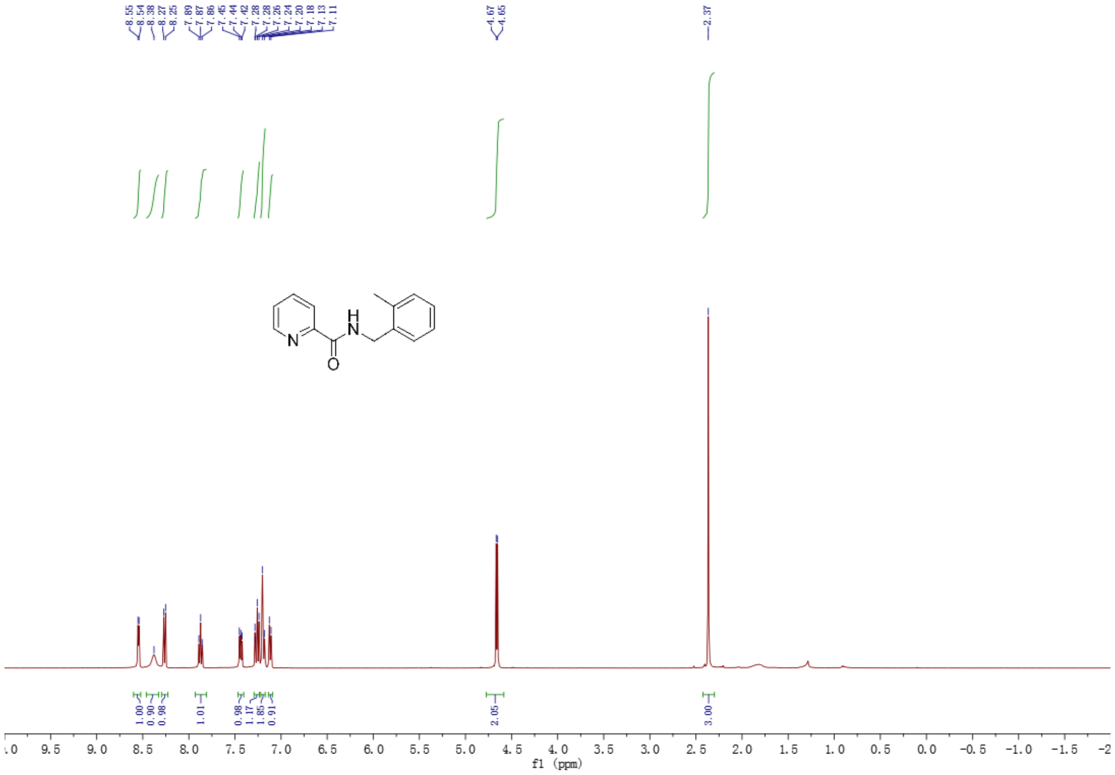

^{13}C NMR spectrum (100 MHz, $CDCl_3$) of **1p**

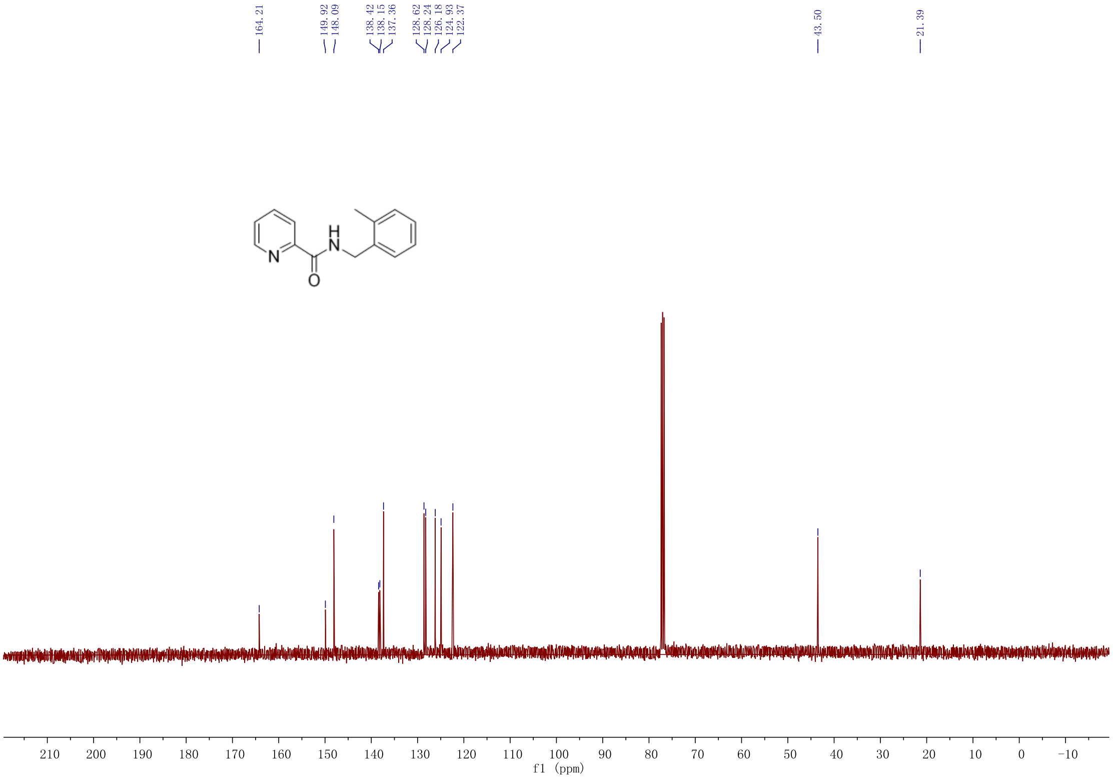

^{1}H NMR spectrum (400 MHz, $CDCl_3$) of **1q**

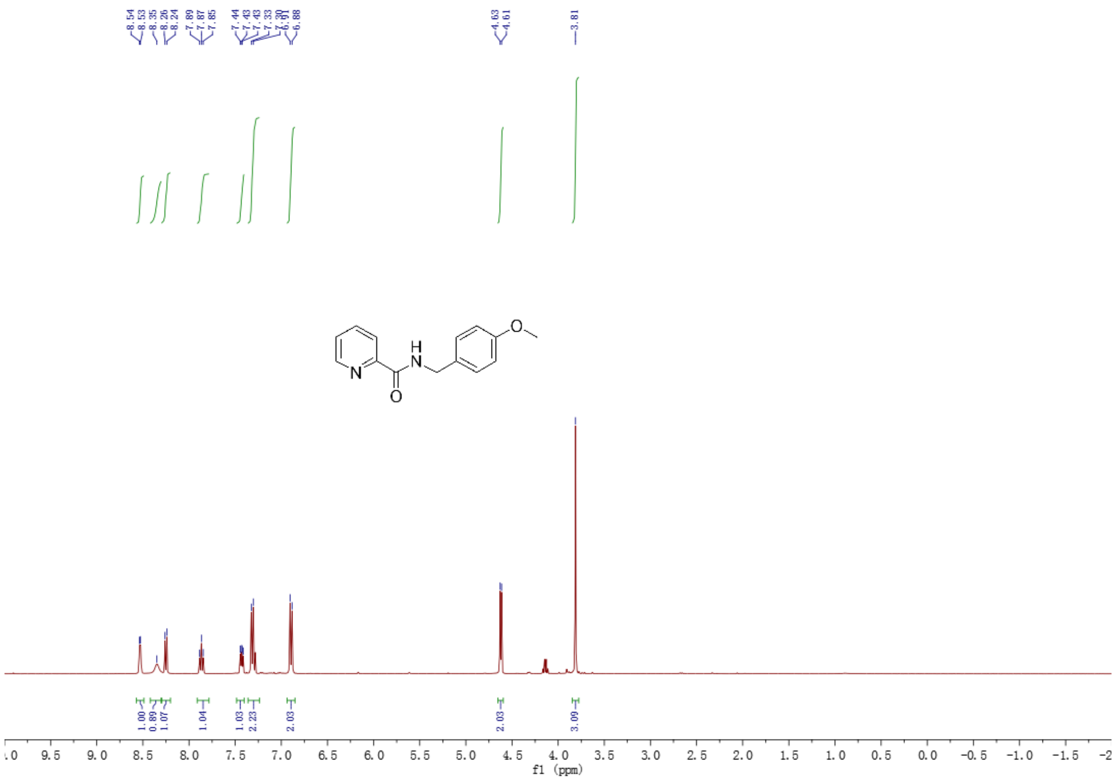

^{13}C NMR spectrum (100 MHz, $CDCl_3$) of **1q**

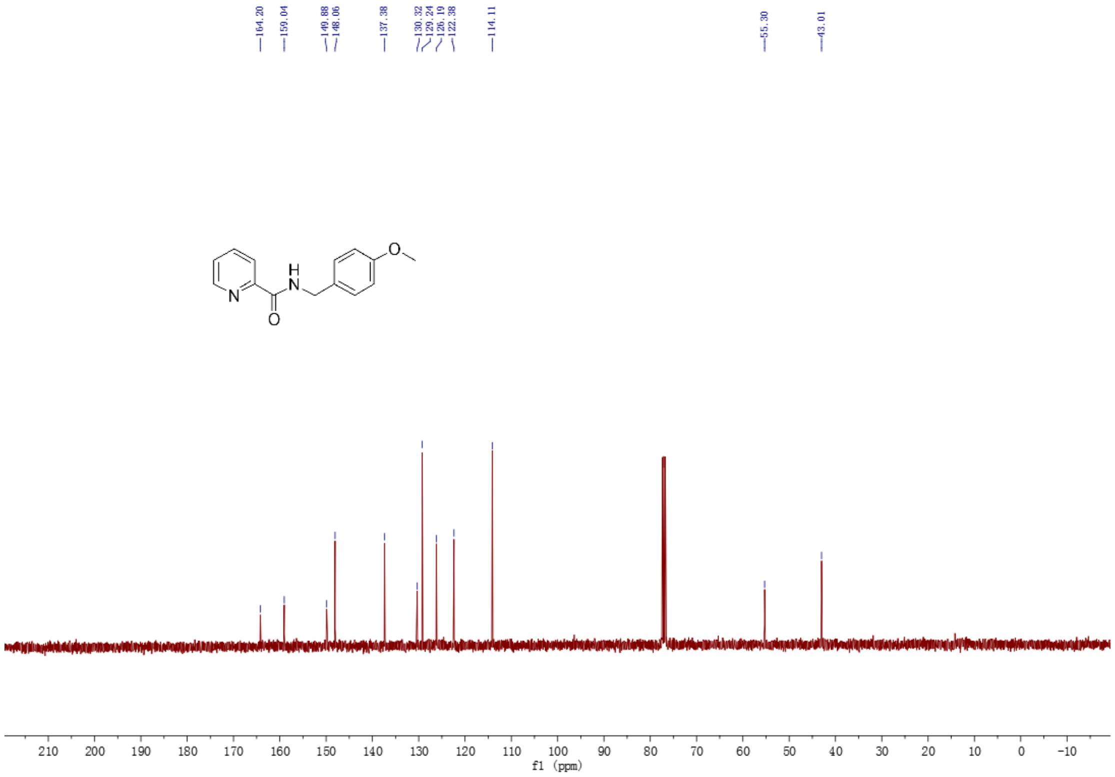

^{1}H NMR spectrum (400 MHz, $CDCl_3$) of **1r**

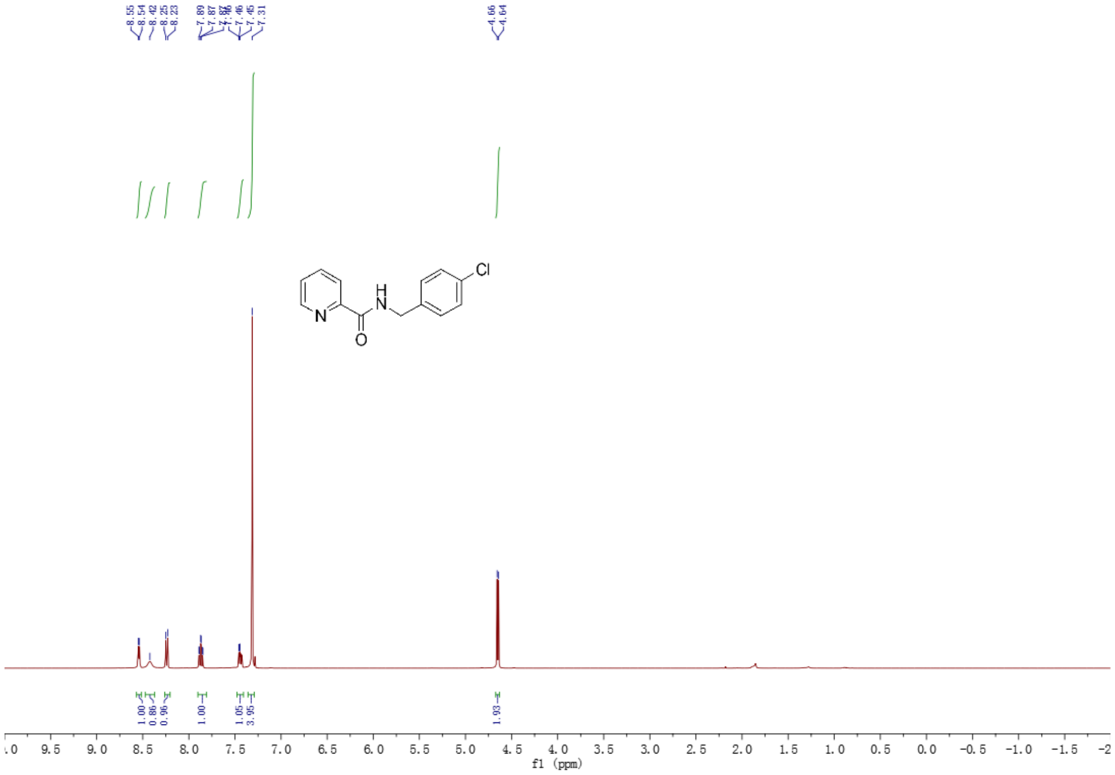

^{13}C NMR spectrum (100 MHz, $CDCl_3$) of **1r**

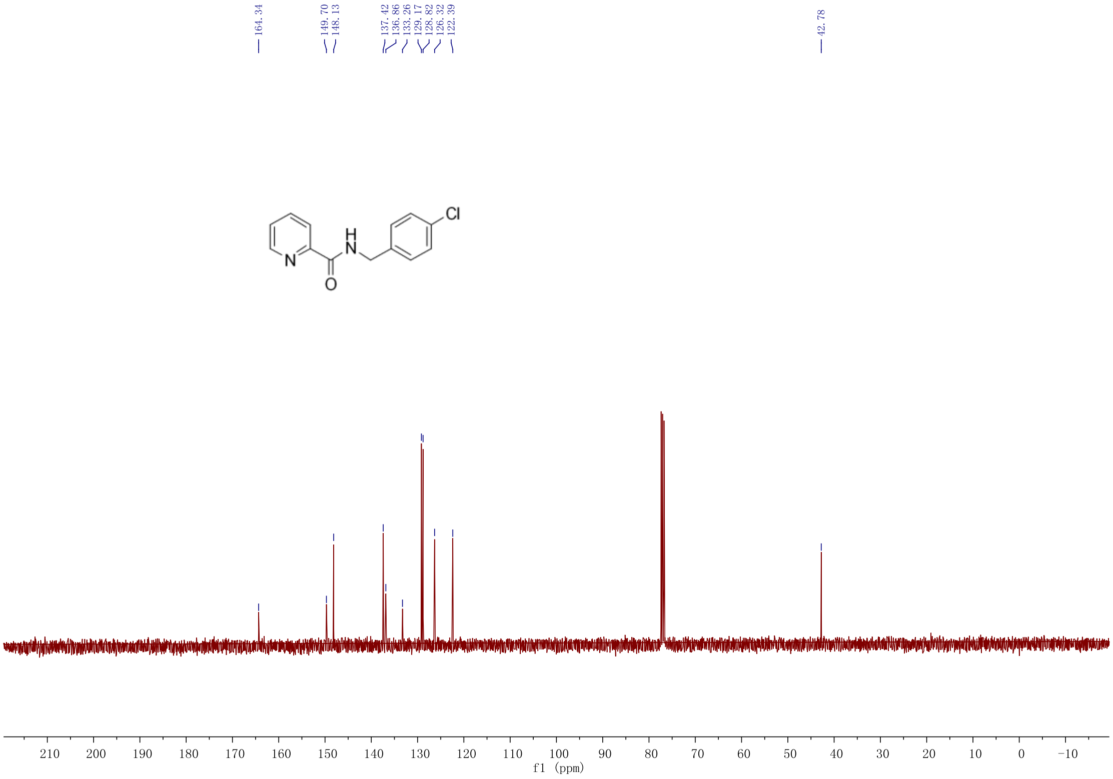

^{1}H NMR spectrum (401 MHz, $CDCl_3$) of **1s**

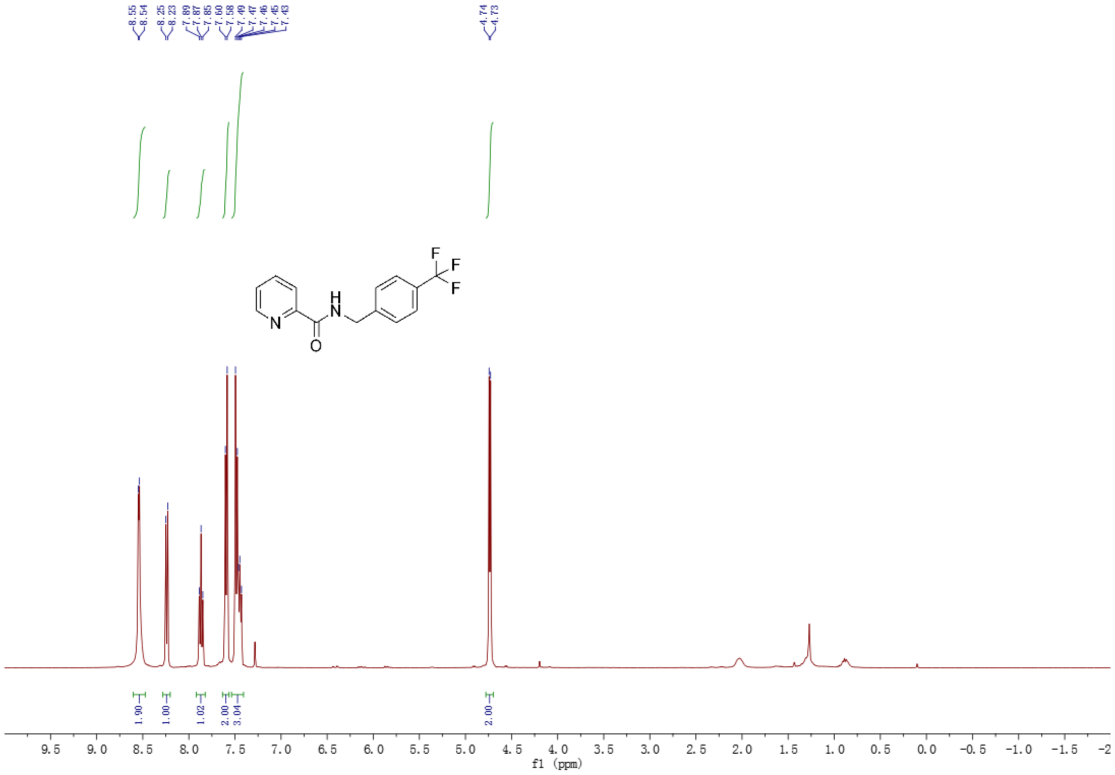

^{13}C NMR spectrum (100 MHz, $CDCl_3$) of **1s**

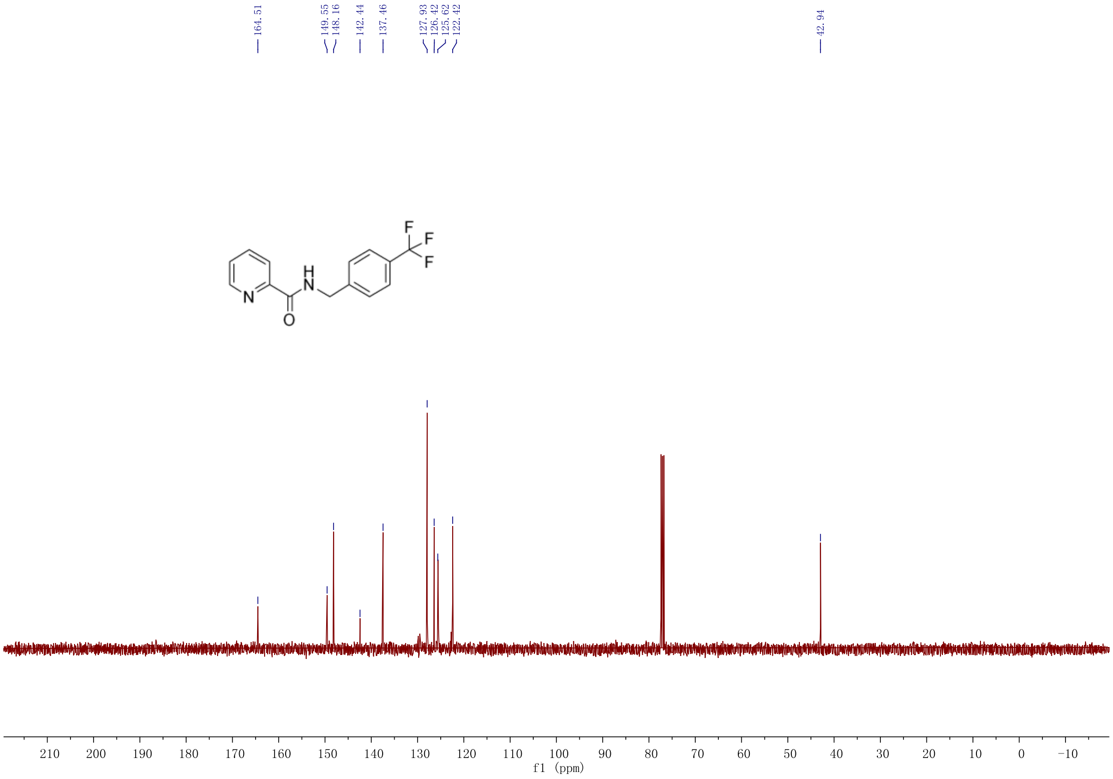

^{1}H NMR spectrum (400 MHz, $CDCl_3$) of **1t**

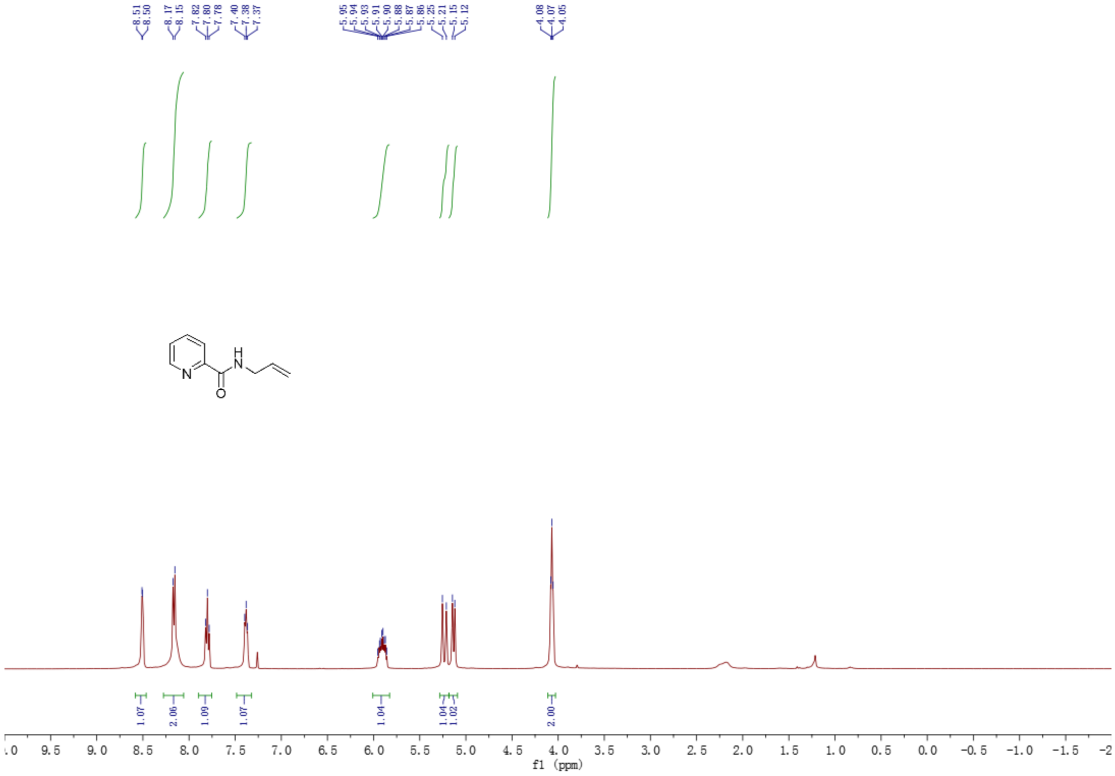

^{13}C NMR spectrum (100 MHz, $CDCl_3$) of **1t**

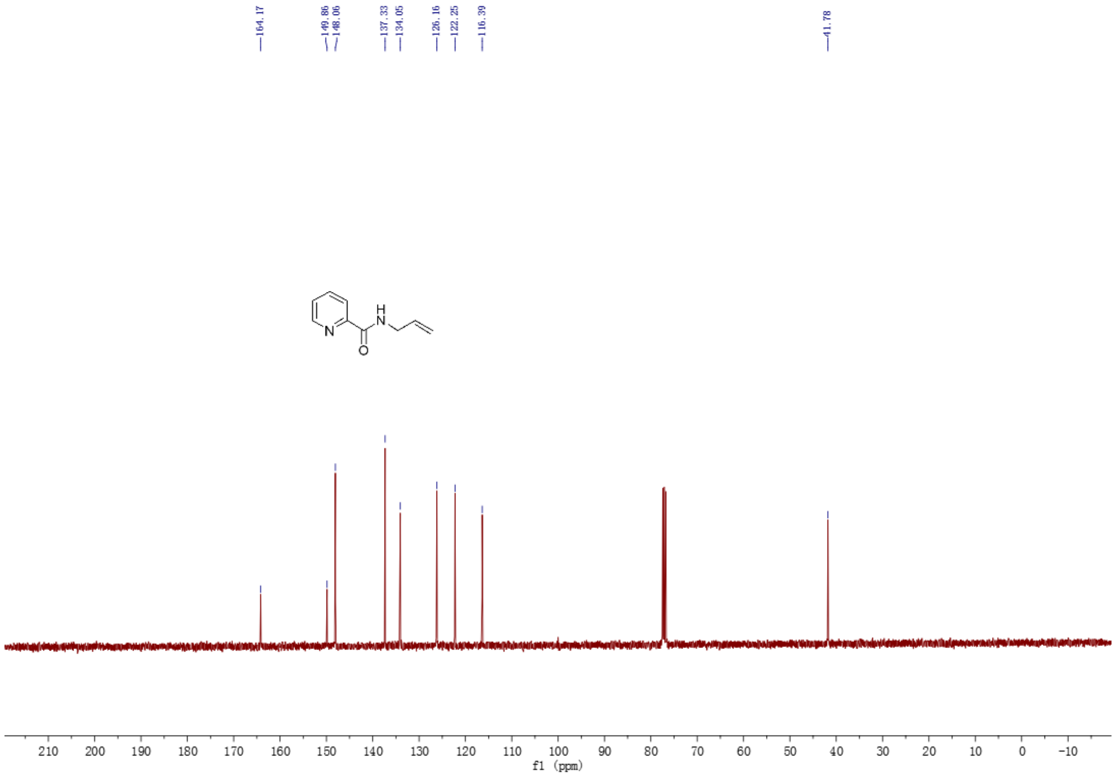

^{1}H NMR spectrum (400 MHz, $CDCl_3$) of **1u**

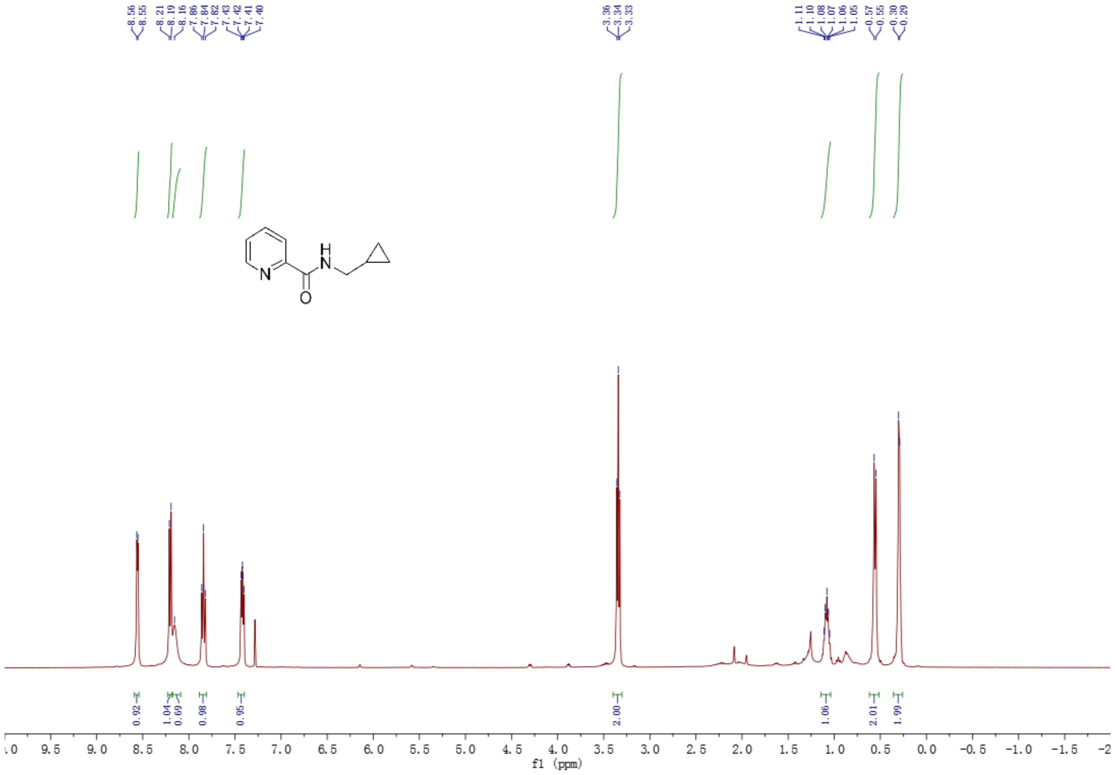

^{13}C NMR spectrum (100 MHz, $CDCl_3$) of **1u**

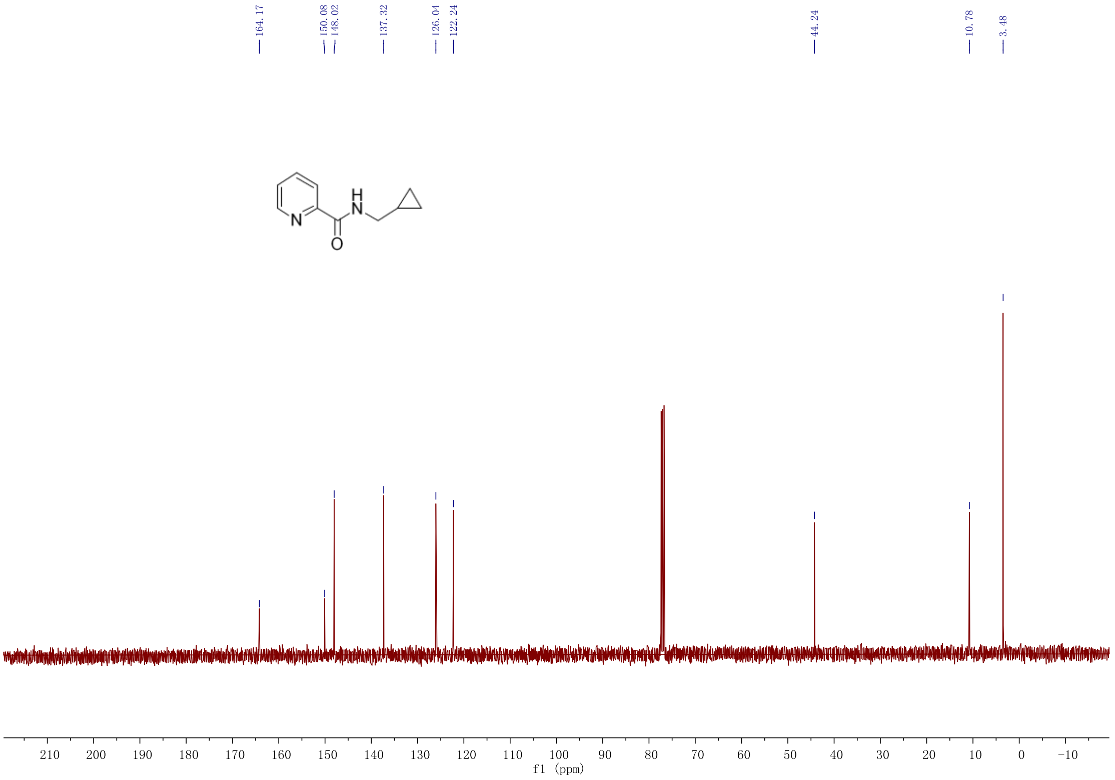

^{1}H NMR spectrum (400 MHz, $CDCl_3$) of **1v**

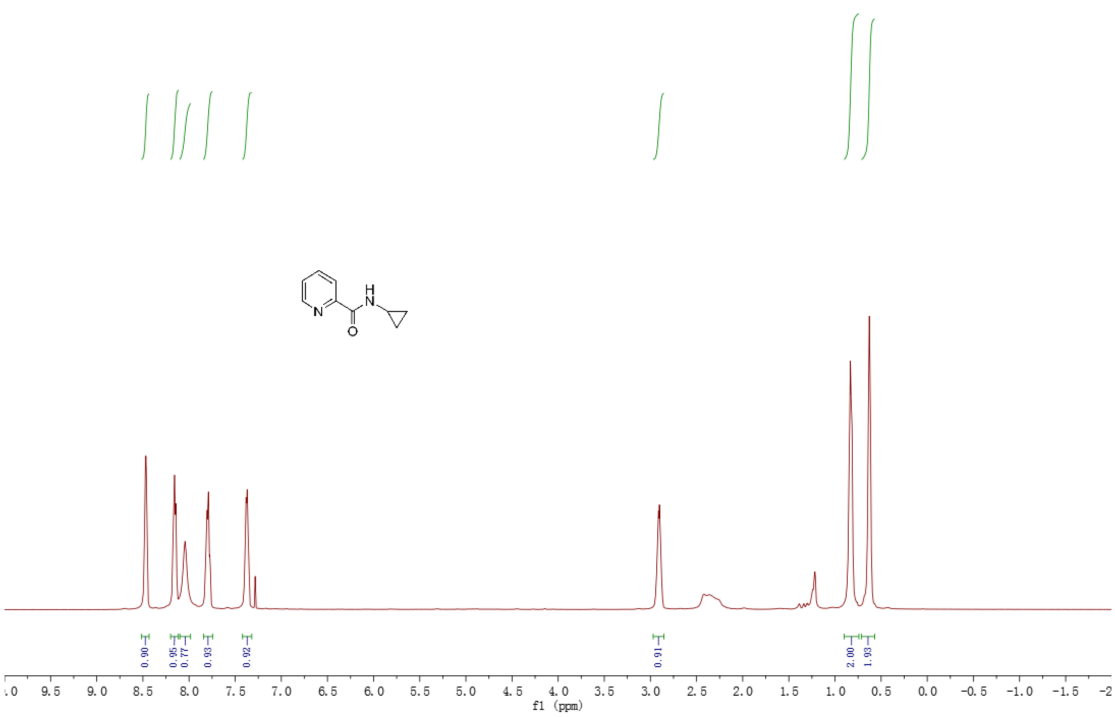

^{13}C NMR spectrum (100 MHz, $CDCl_3$) of **1v**

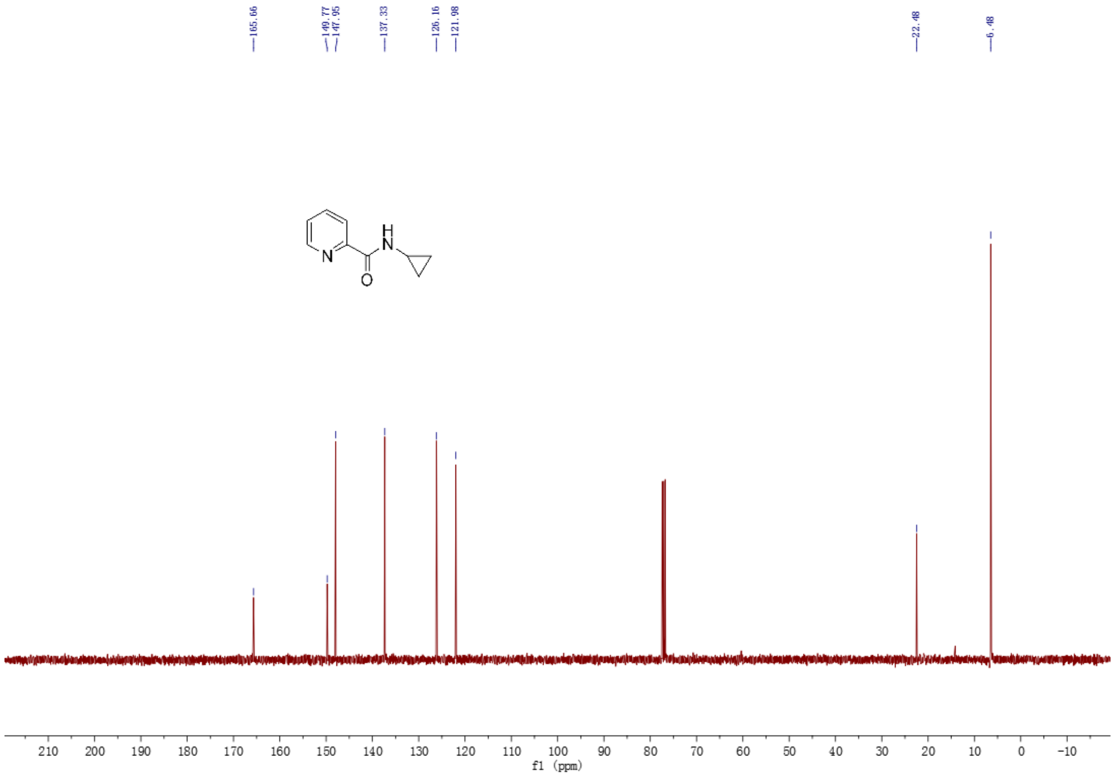

^{1}H NMR spectrum (400 MHz, $CDCl_3$) of **1w**

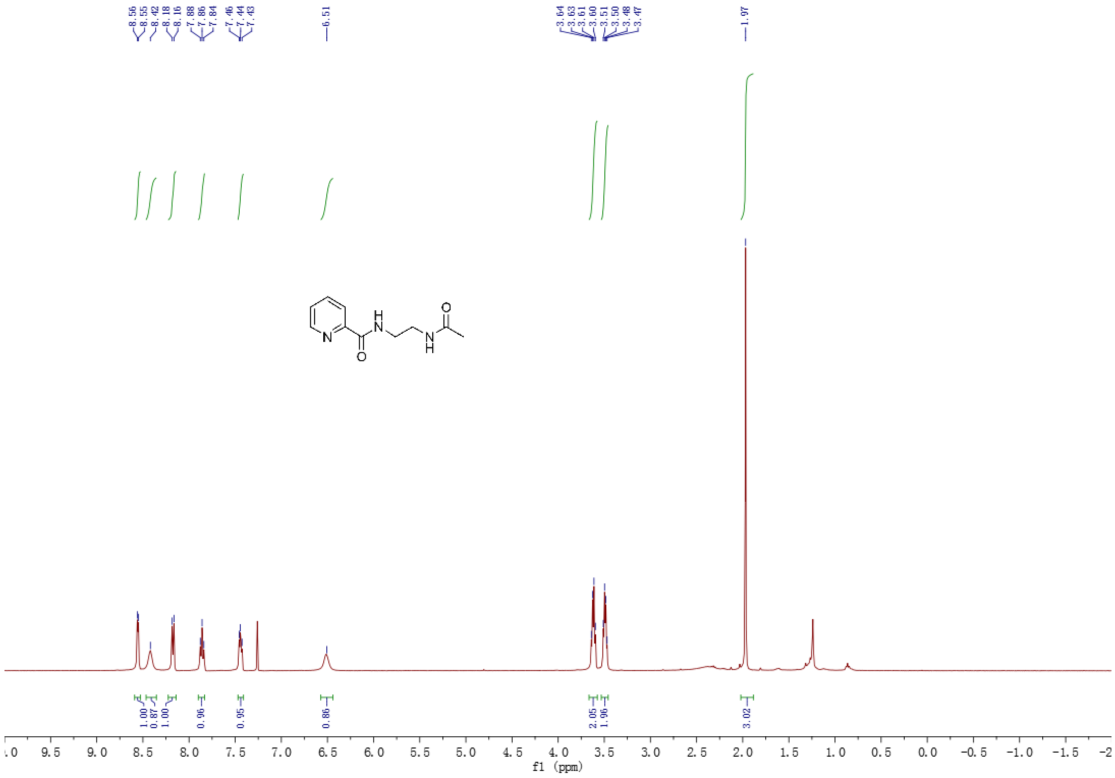

^{13}C NMR spectrum (100 MHz, $CDCl_3$) of **1w**

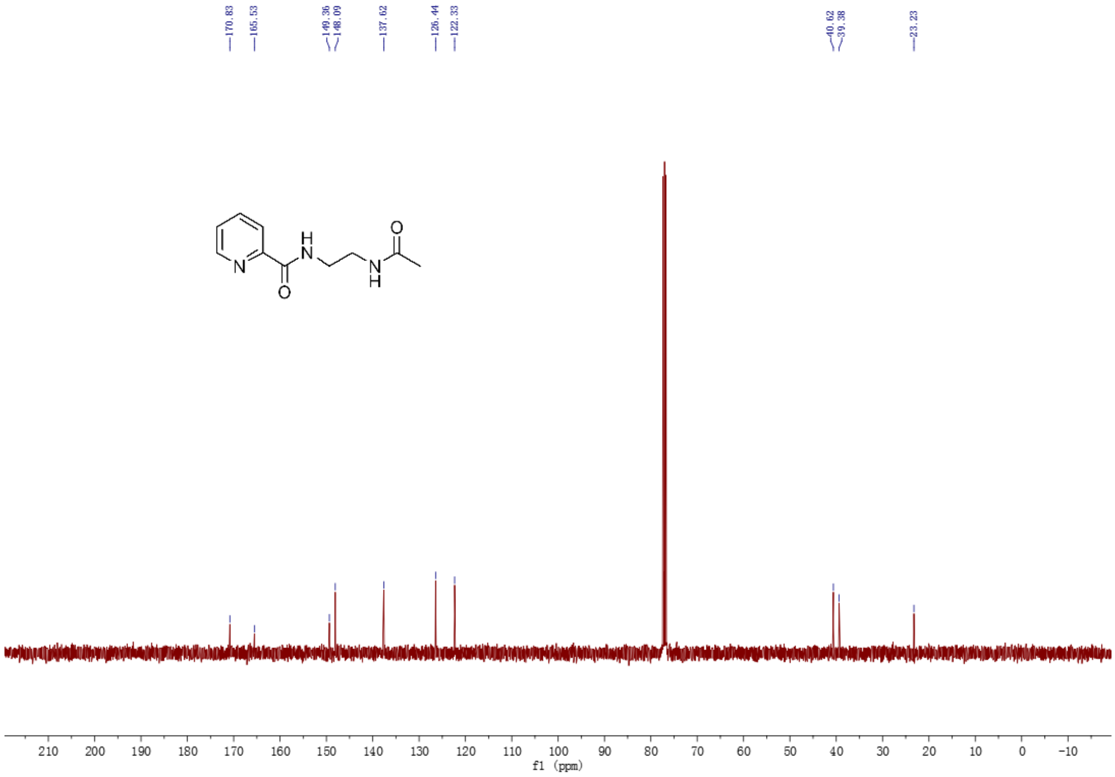

^{1}H NMR spectrum (400 MHz, $CDCl_3$) of **1x**

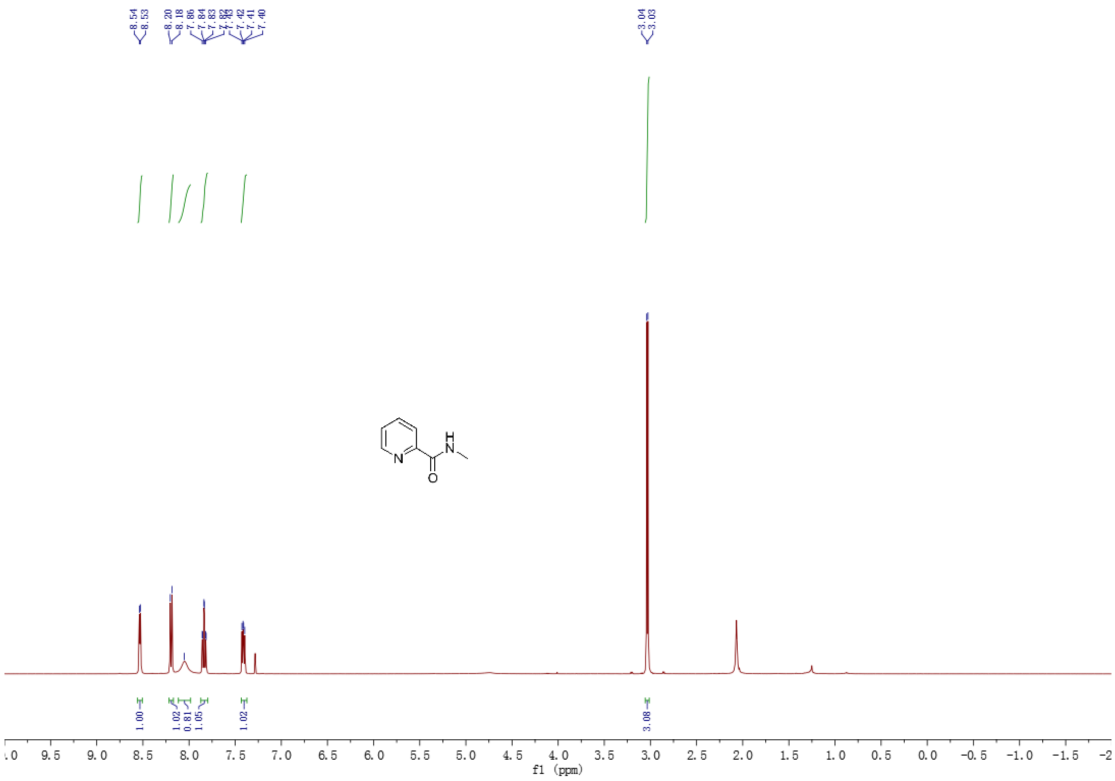

^{13}C NMR spectrum (100 MHz, $CDCl_3$) of **1x**

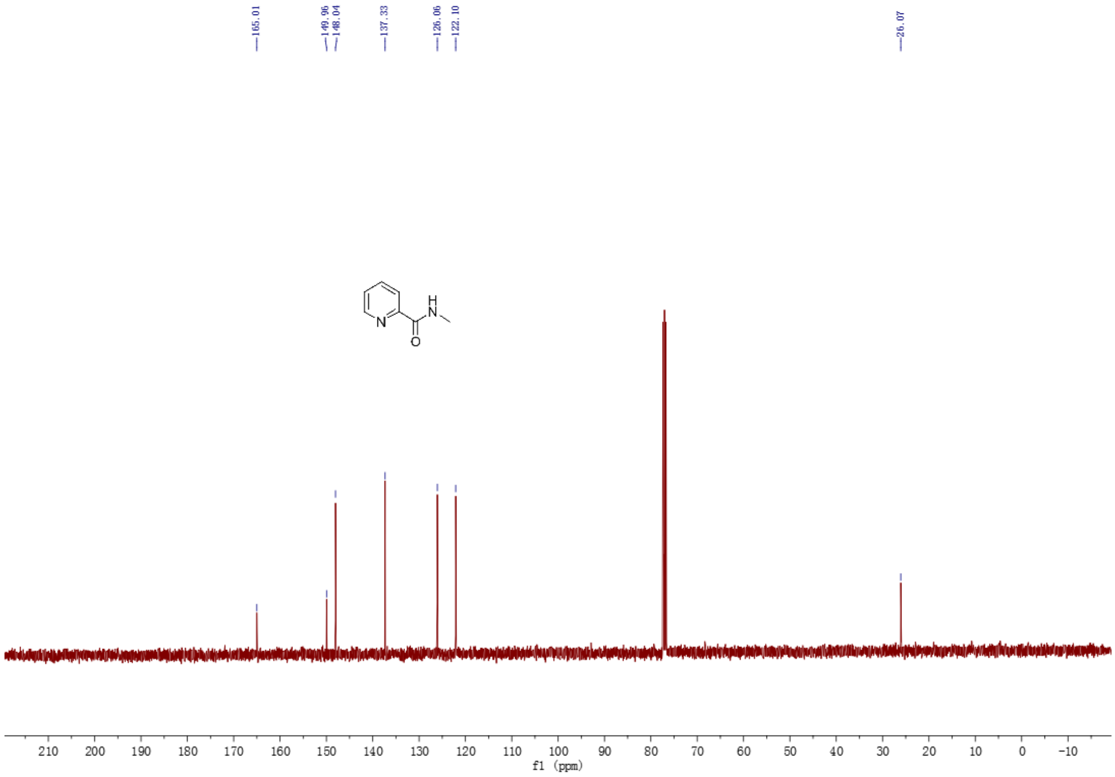

^{1}H NMR spectrum (400 MHz, $CDCl_3$) of **1y**

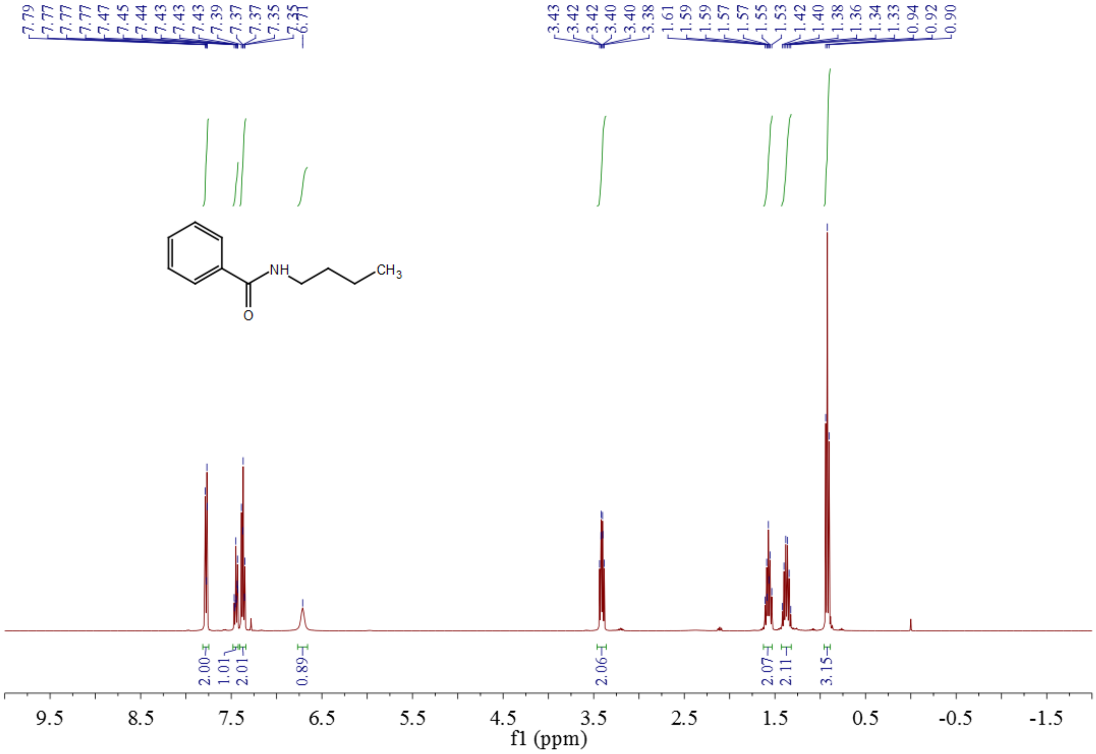

^{13}C NMR spectrum (100 MHz, $CDCl_3$) of **1y**

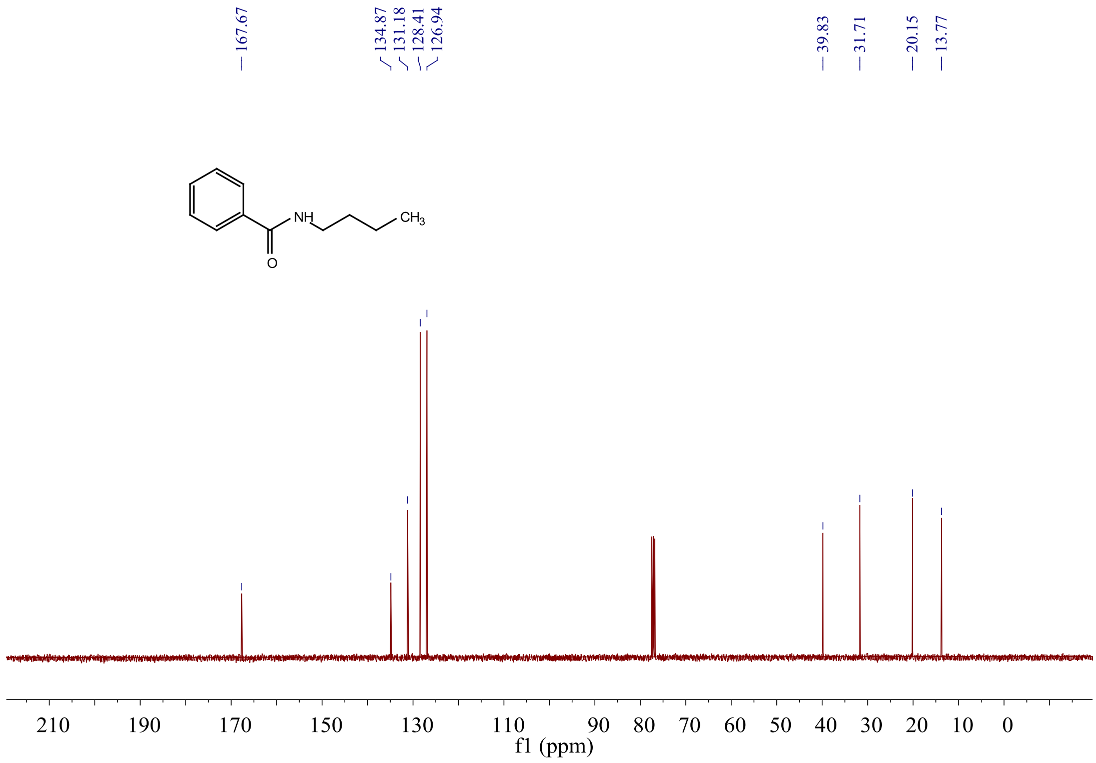

^{1}H NMR spectrum (400 MHz, $CDCl_3$) of **1z**

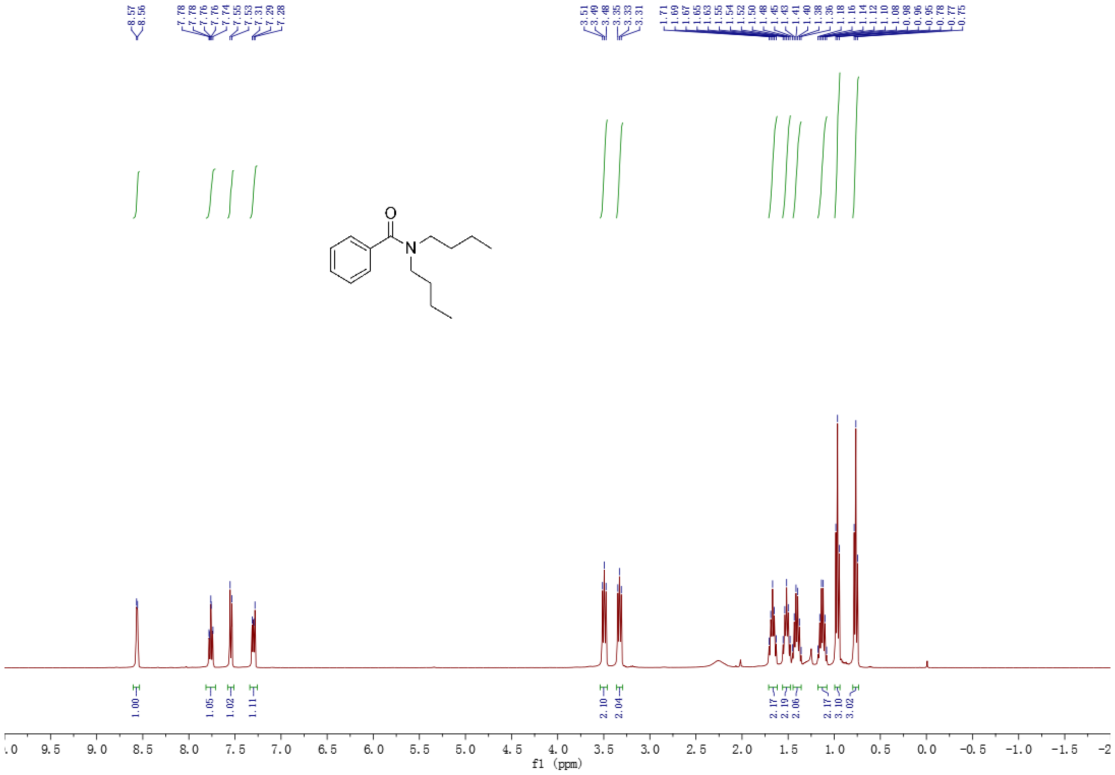

^{13}C NMR spectrum (100 MHz, $CDCl_3$) of **1z**

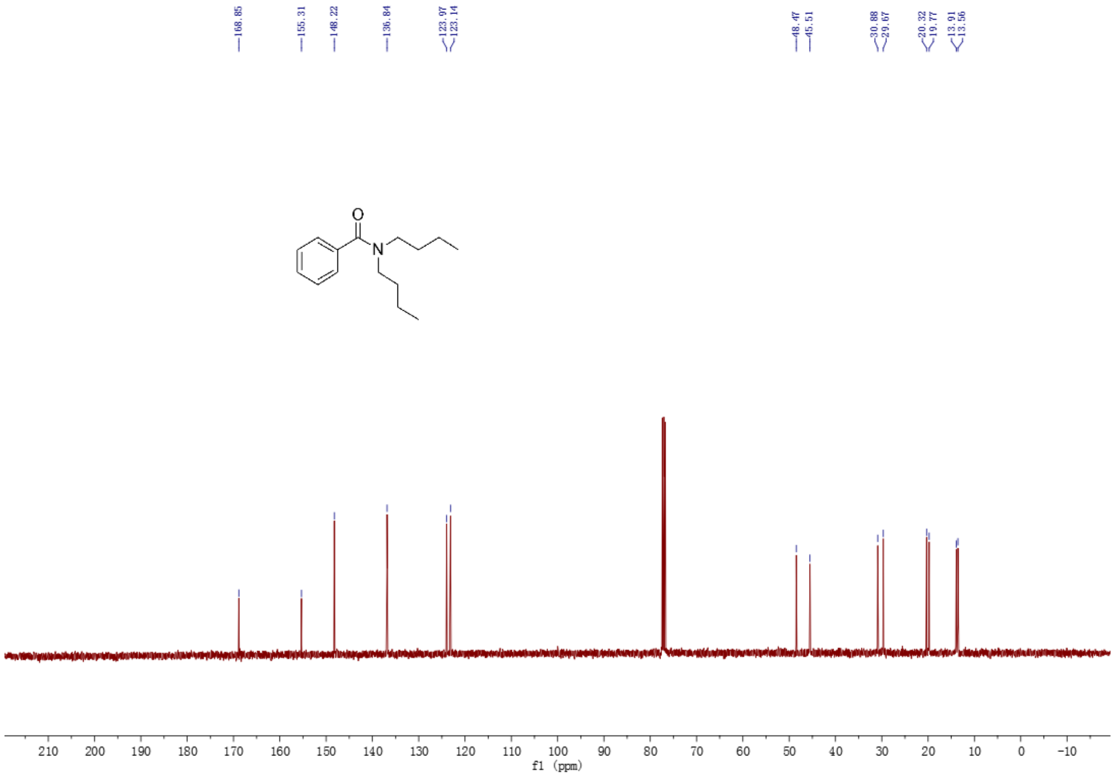

^{1}H NMR spectrum (400 MHz, $CDCl_3$) of *d*-**1m**

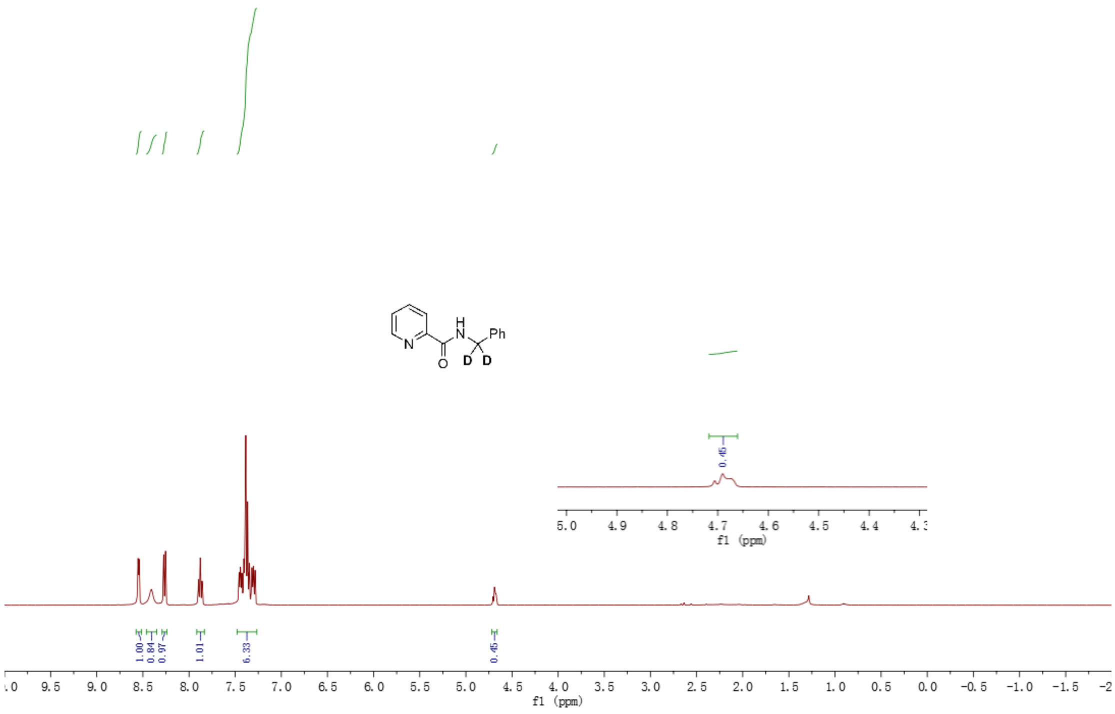

^{13}C NMR spectrum (100 MHz, $CDCl_3$) of *d*-**1m**

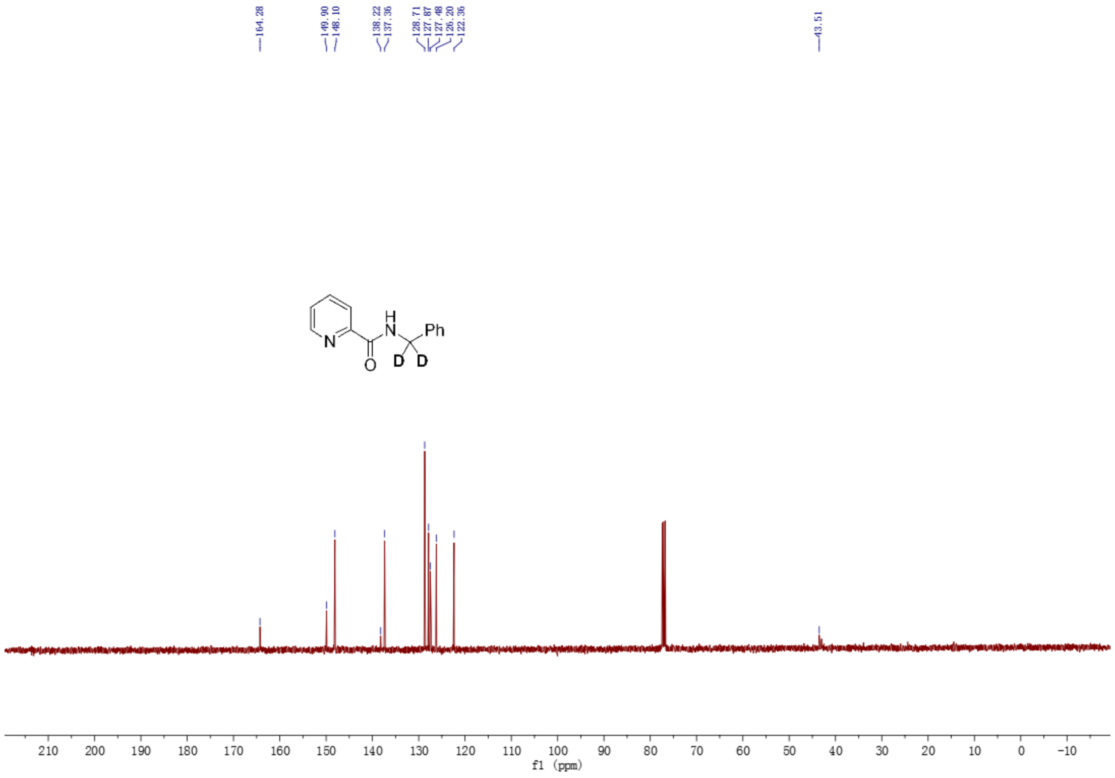

^{1}H NMR spectrum (400 MHz, $CDCl_3$) of **3a**

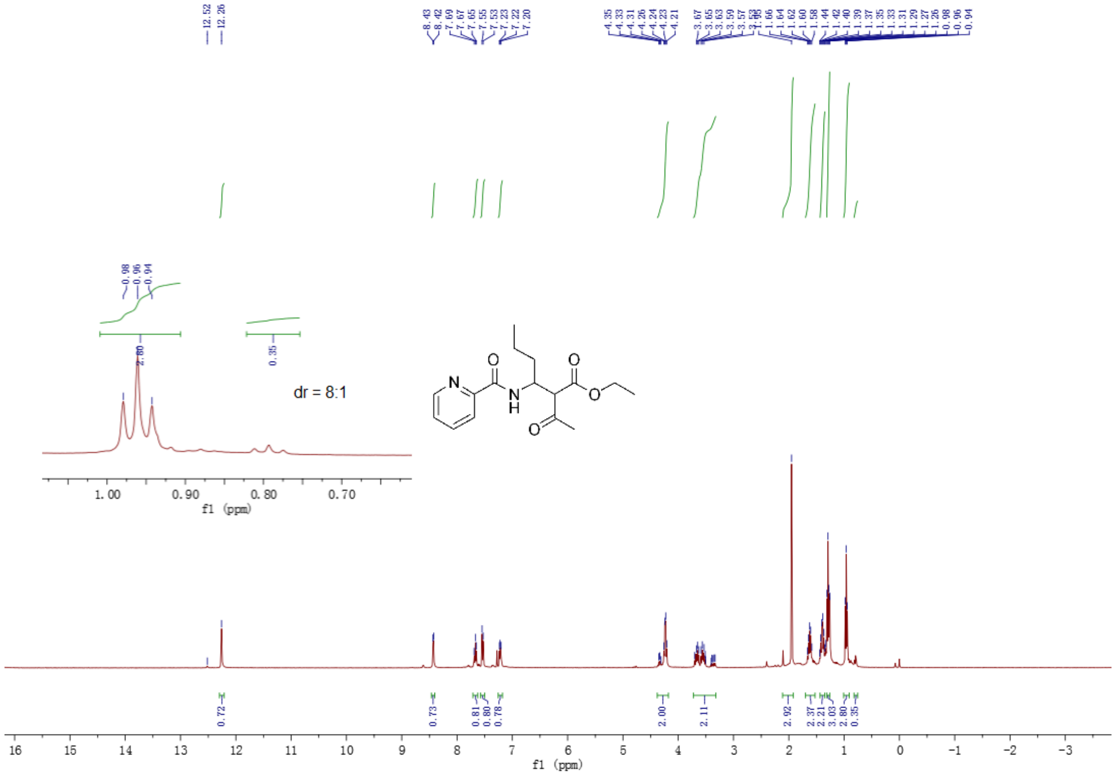

^{13}C NMR spectrum (100 MHz, $CDCl_3$) of **3a**

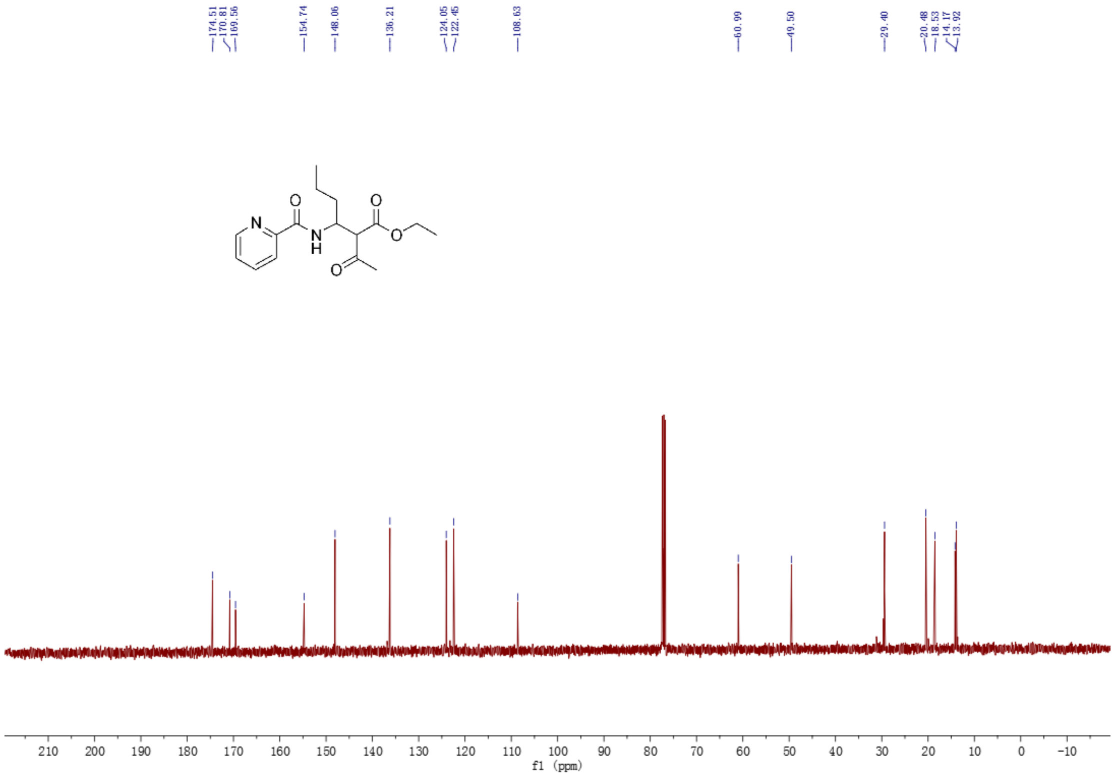

^{1}H NMR spectrum (400 MHz, $CDCl_3$) of **3b**

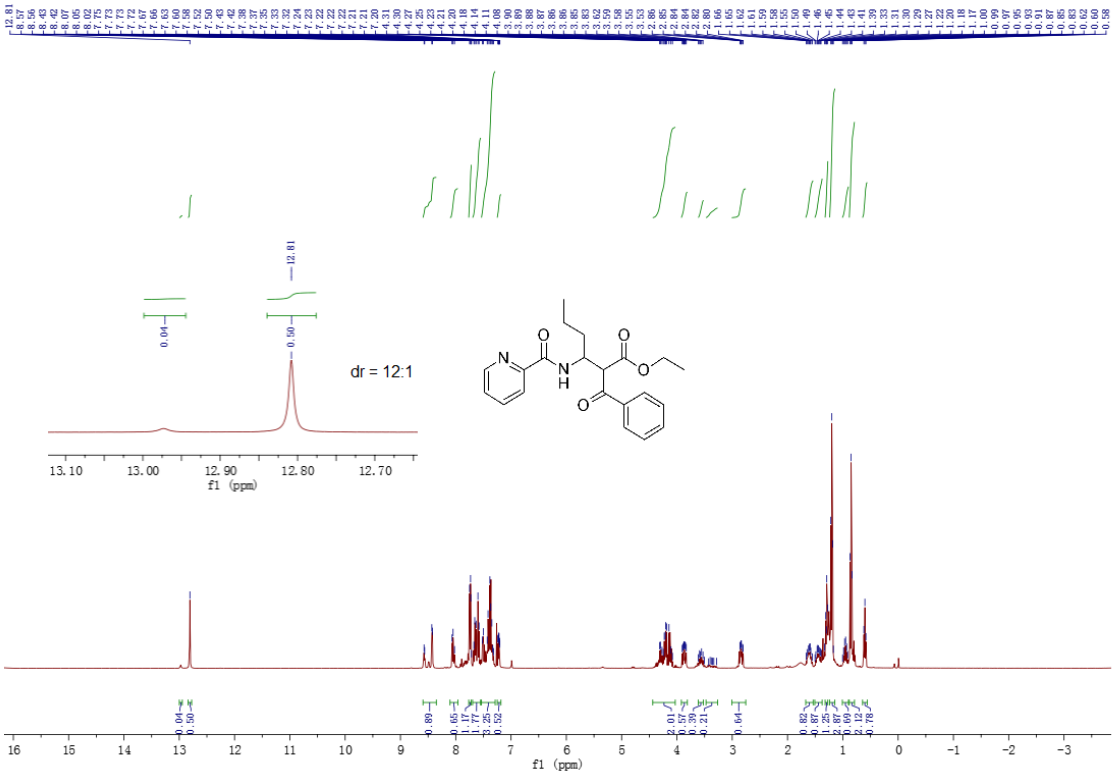

^{13}C NMR spectrum (100 MHz, $CDCl_3$) of **3b**

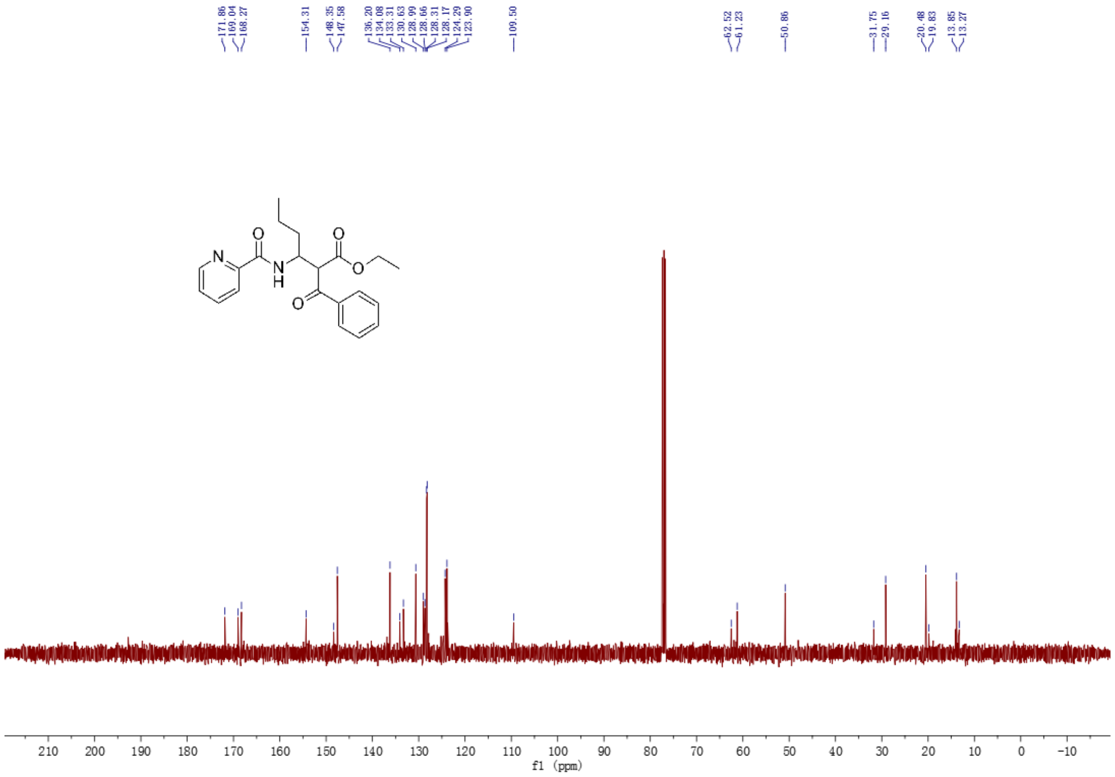

^{1}H NMR spectrum (400 MHz, $CDCl_3$) of **3c**

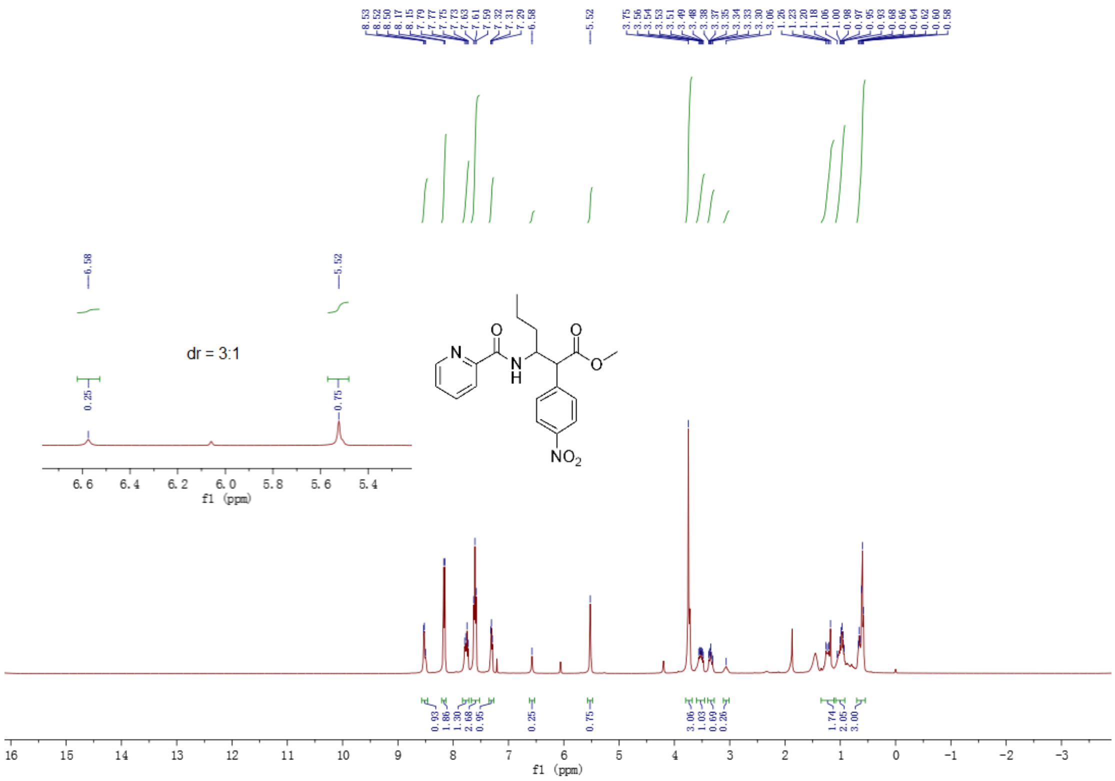

^{13}C NMR spectrum (100 MHz, $CDCl_3$) of **3c**

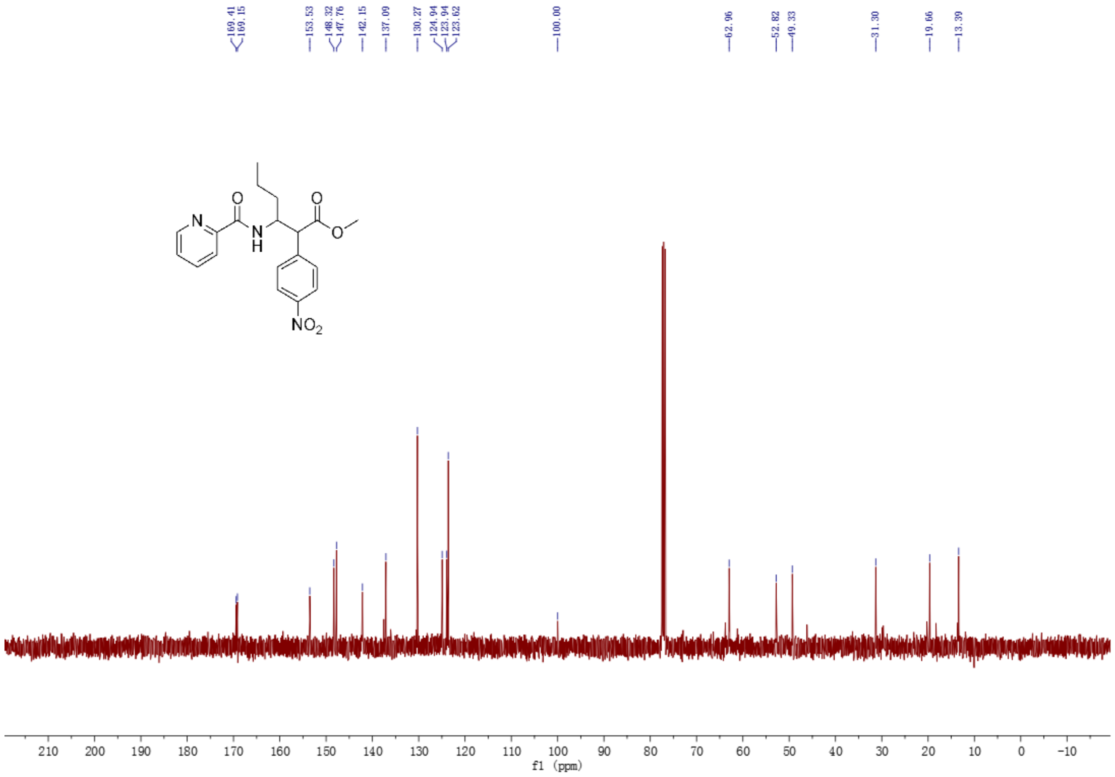

^{1}H NMR spectrum (400 MHz, $CDCl_3$) of **3d**

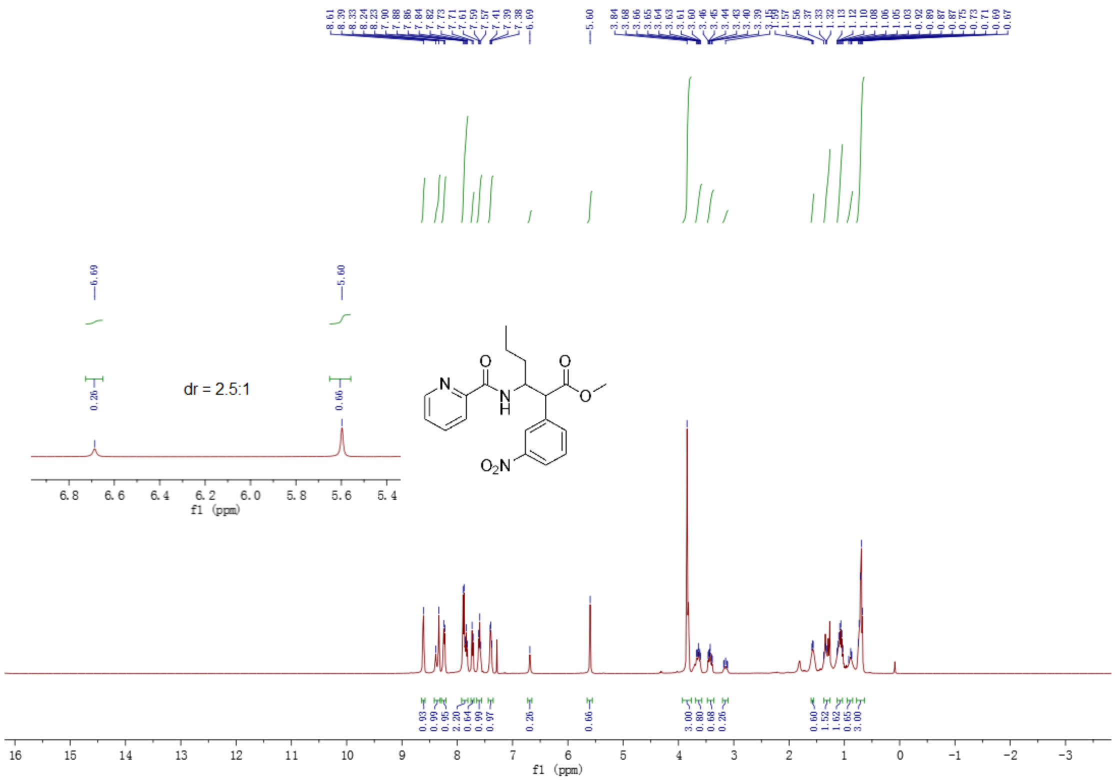

^{13}C NMR spectrum (100 MHz, $CDCl_3$) of **3d**

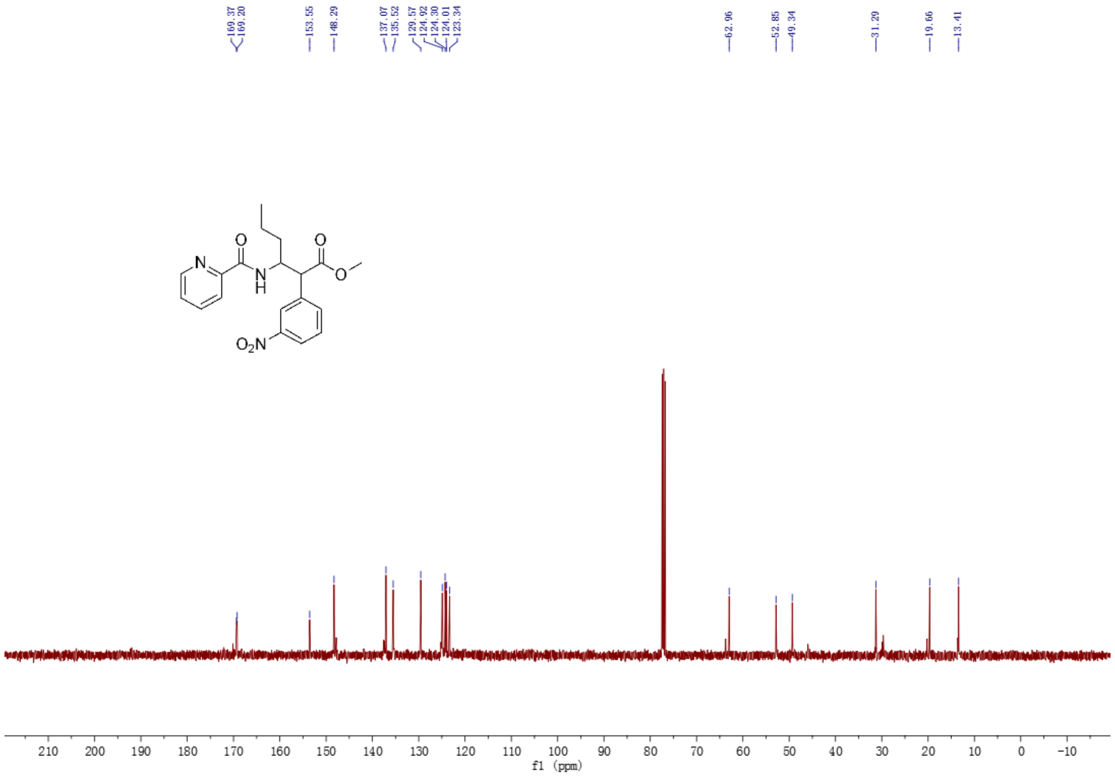

^{1}H NMR spectrum (400 MHz, $CDCl_3$) of **3e**

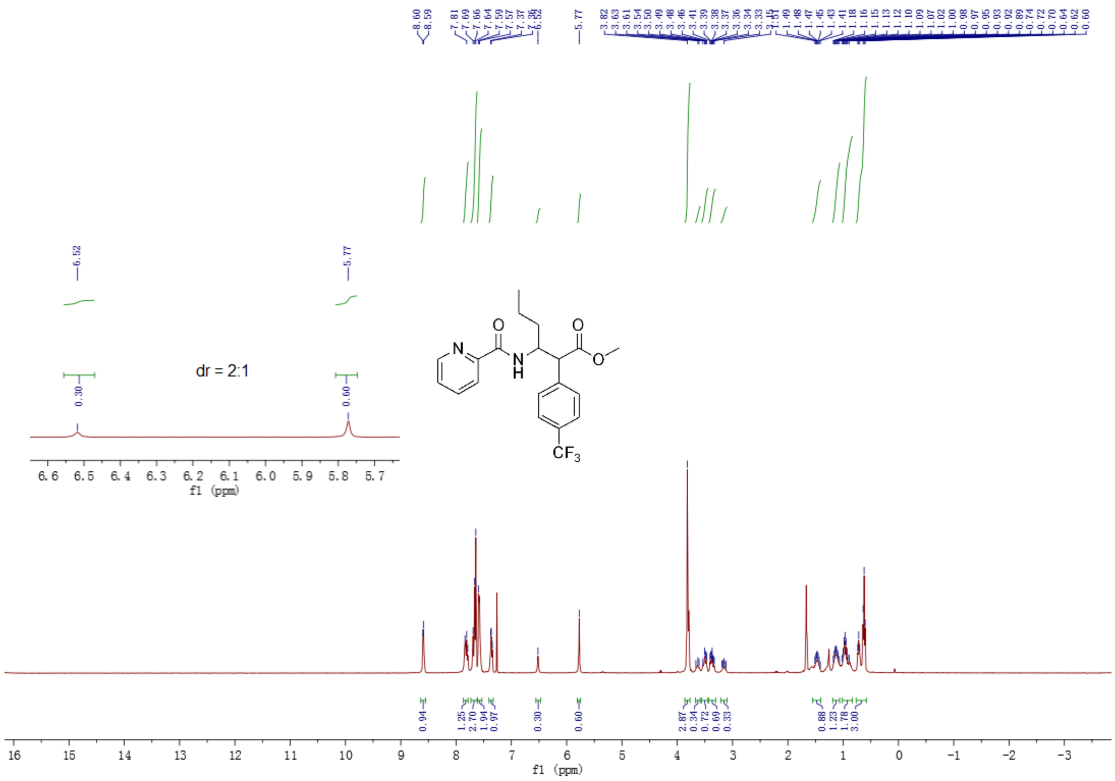

^{13}C NMR spectrum (100 MHz, $CDCl_3$) of **3e**

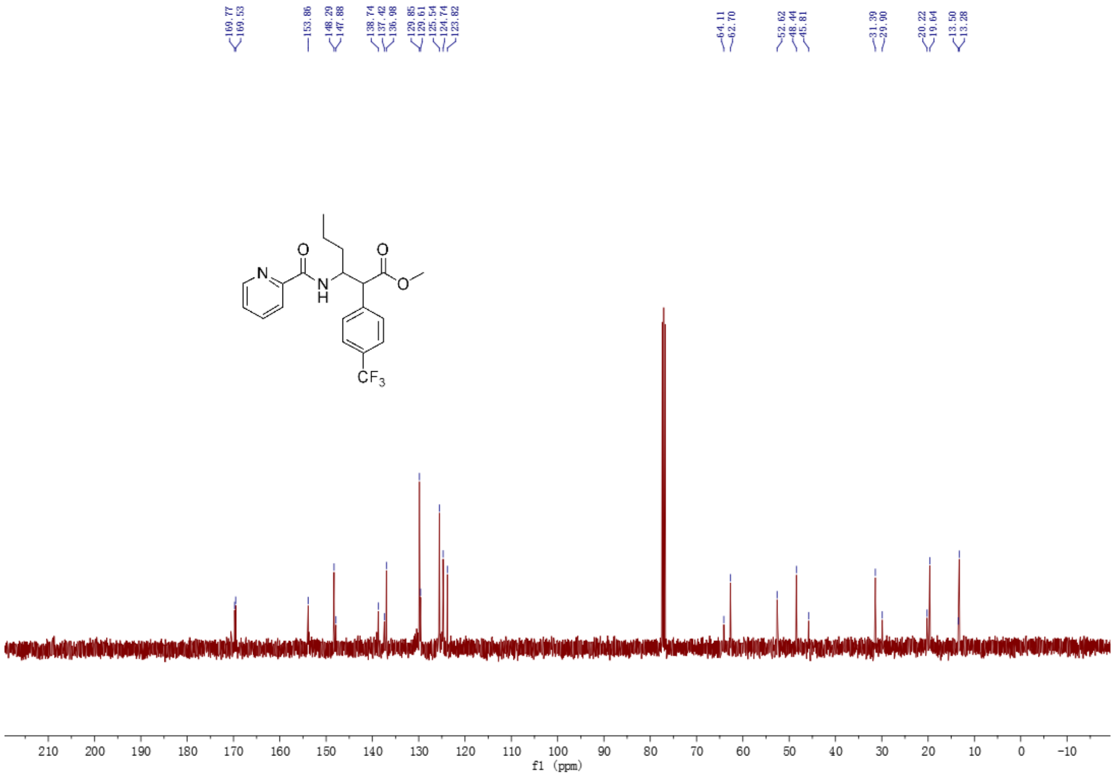

^{1}H NMR spectrum (400 MHz, $CDCl_3$) of **3f**

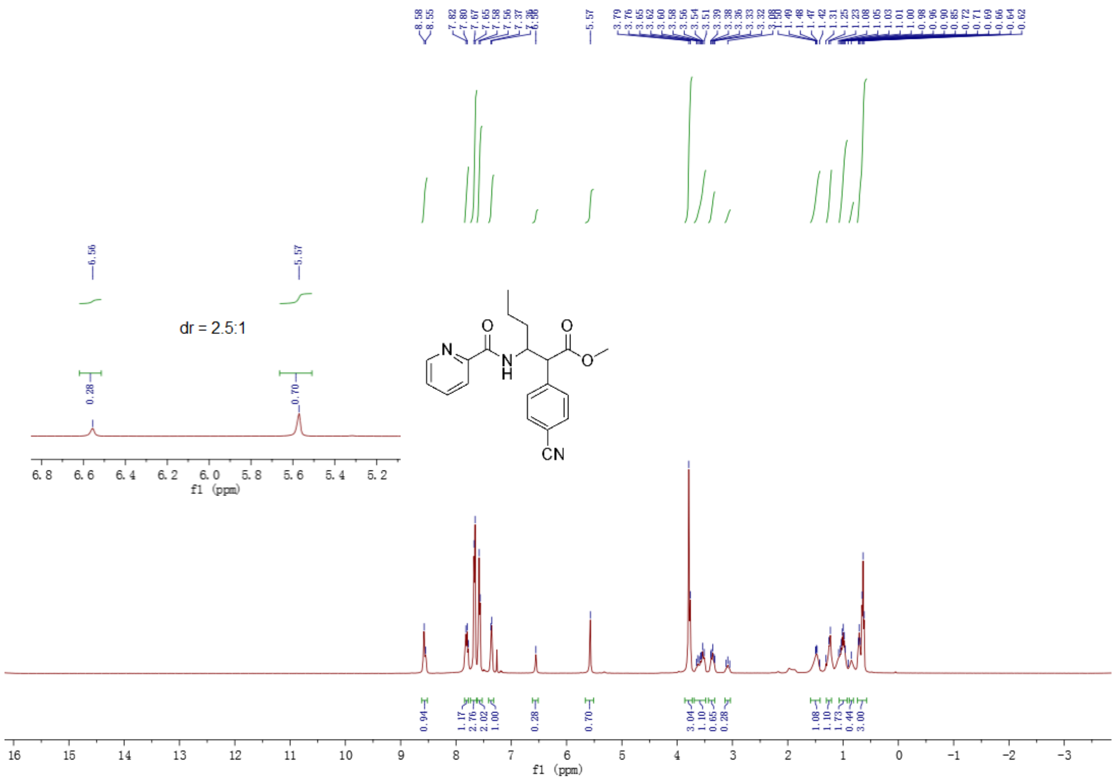

^{13}C NMR spectrum (100 MHz, $CDCl_3$) of **3f**

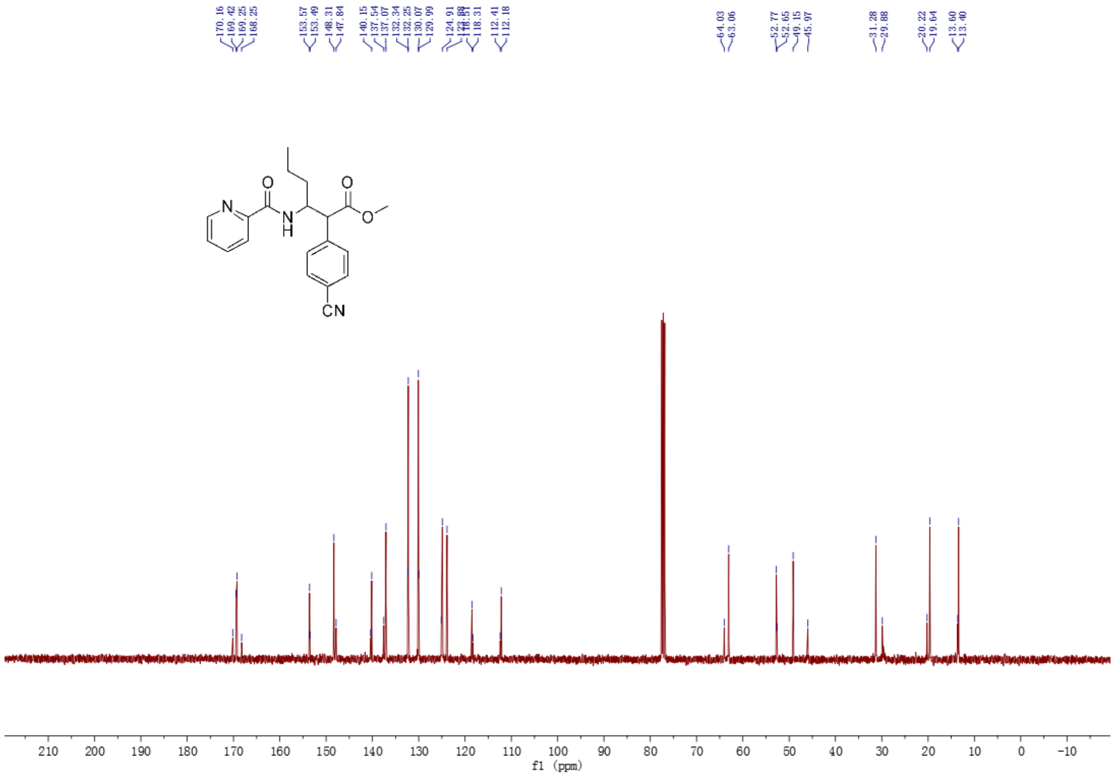

^{1}H NMR spectrum (400 MHz, $CDCl_3$) of **3g**

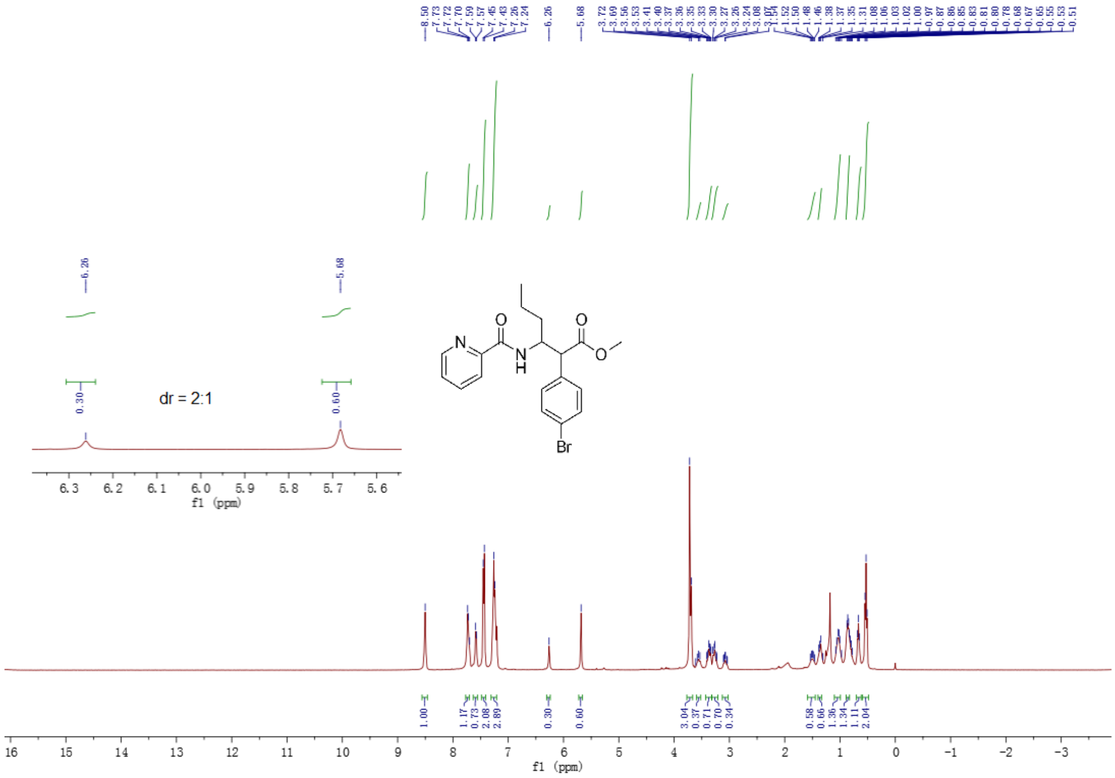

^{13}C NMR spectrum (100 MHz, $CDCl_3$) of **3g**

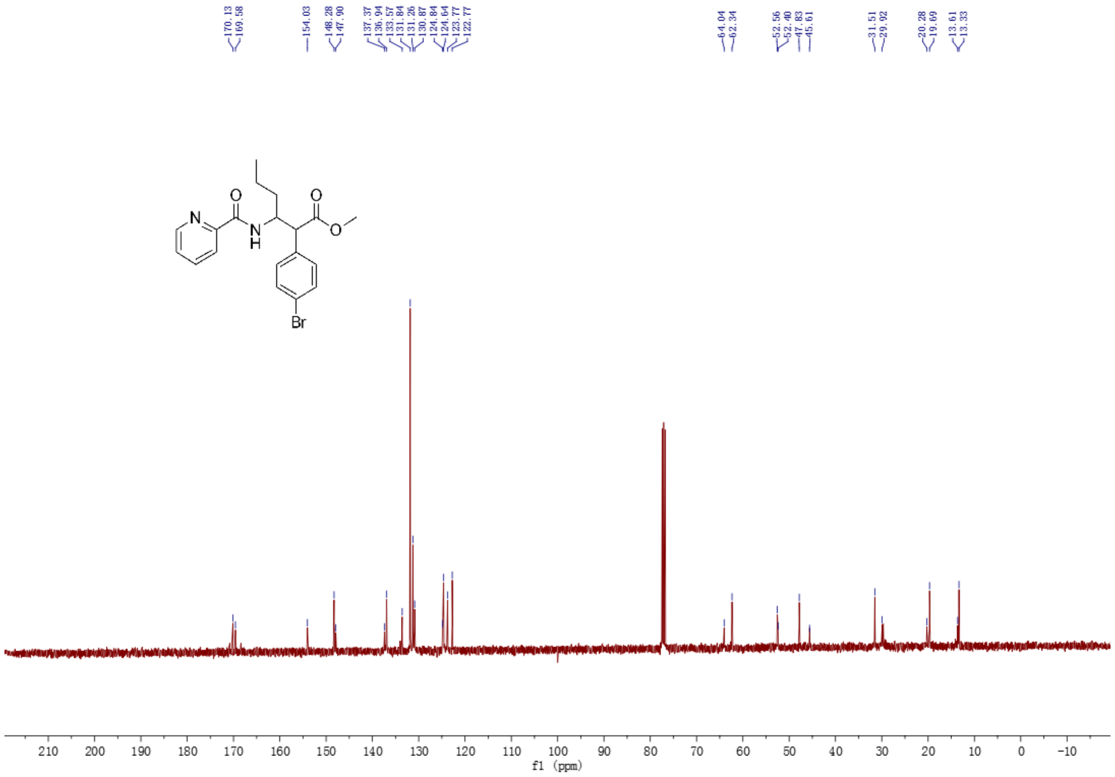

^{1}H NMR spectrum (400 MHz, $CDCl_3$) of **3h**

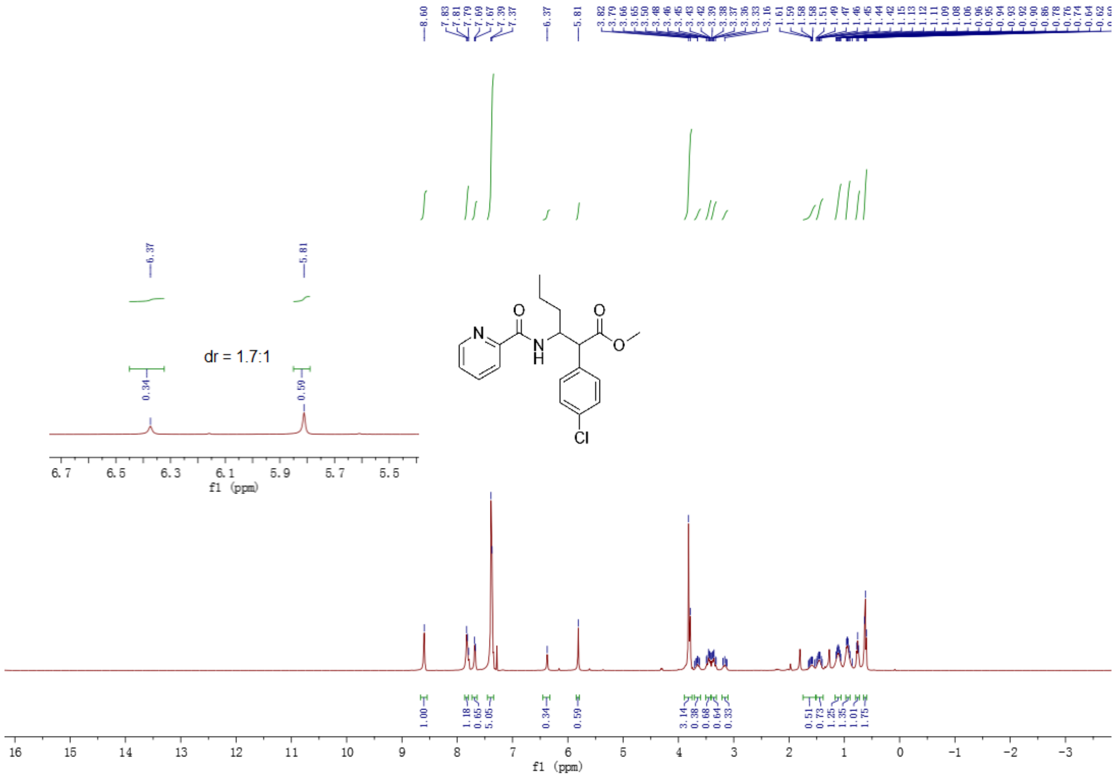

^{13}C NMR spectrum (100 MHz, $CDCl_3$) of **3h**

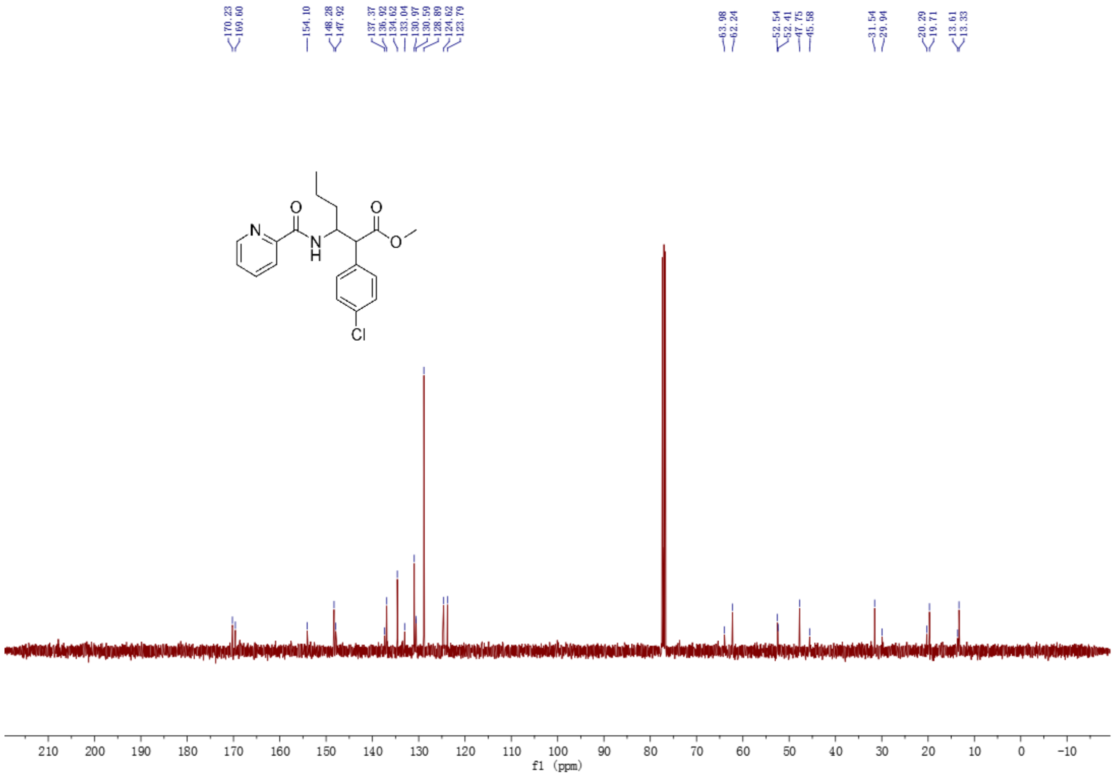

^{1}H NMR spectrum (400 MHz, $CDCl_3$) of **3i**

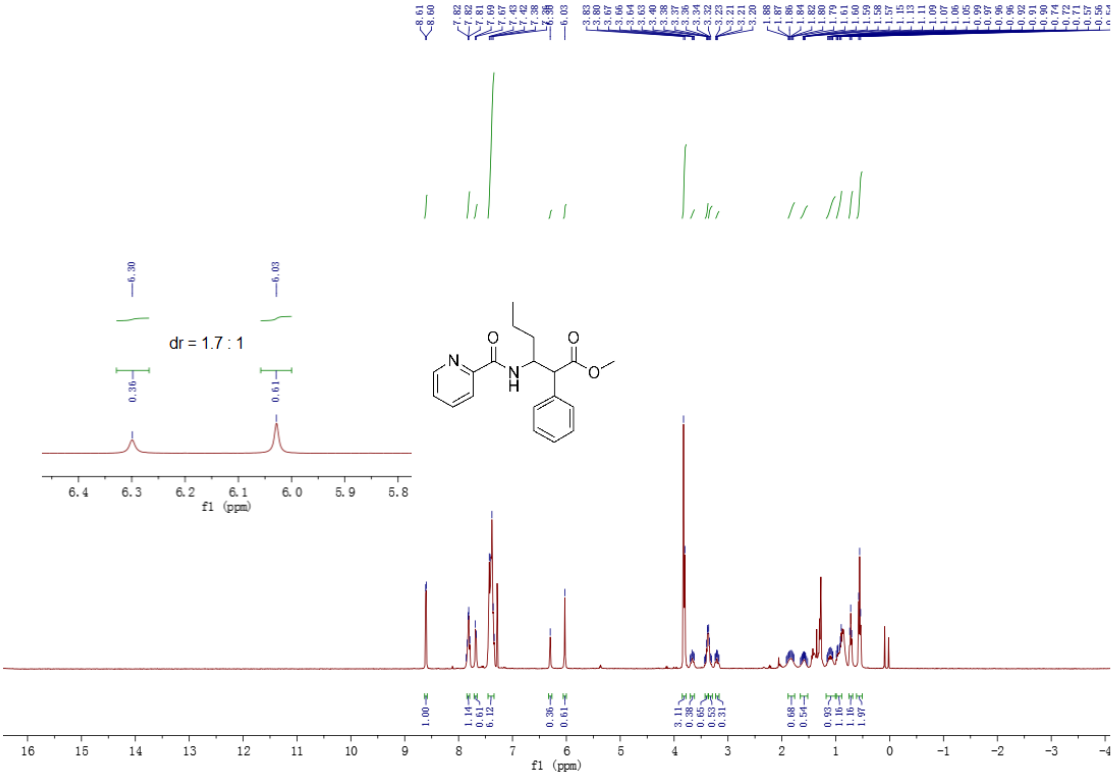

^{13}C NMR spectrum (100 MHz, $CDCl_3$) of **3i**

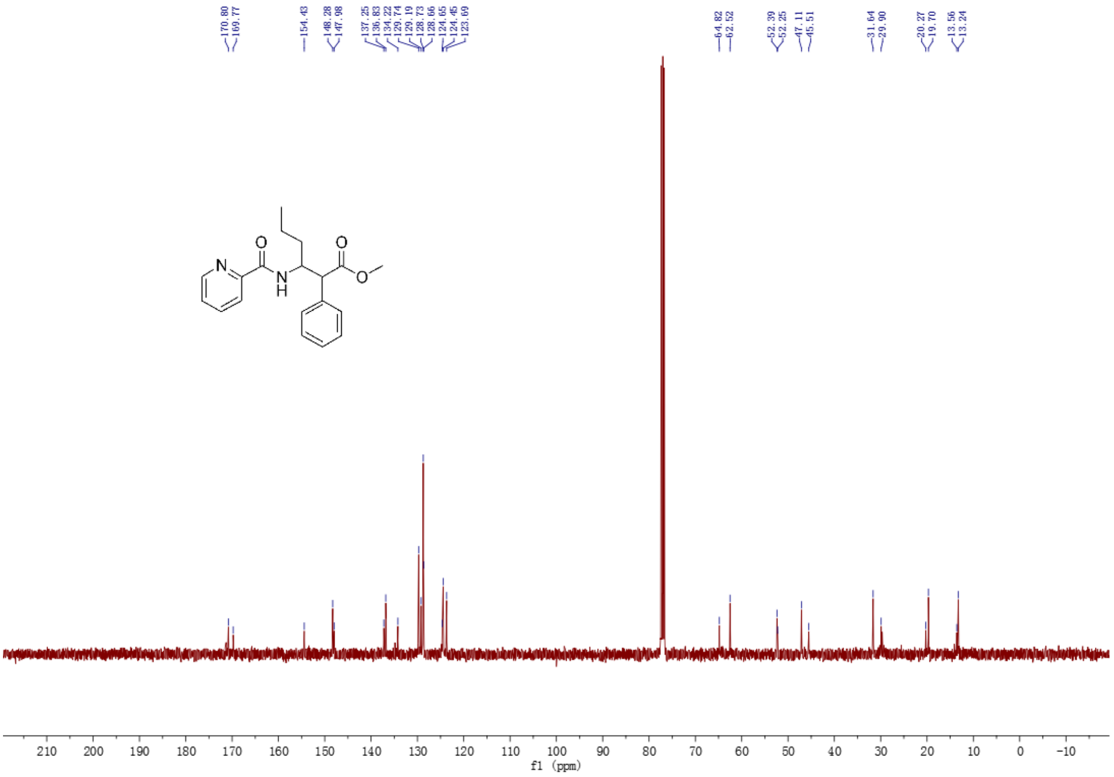

^{1}H NMR spectrum (400 MHz, $CDCl_3$) of **3k** (***Z***)

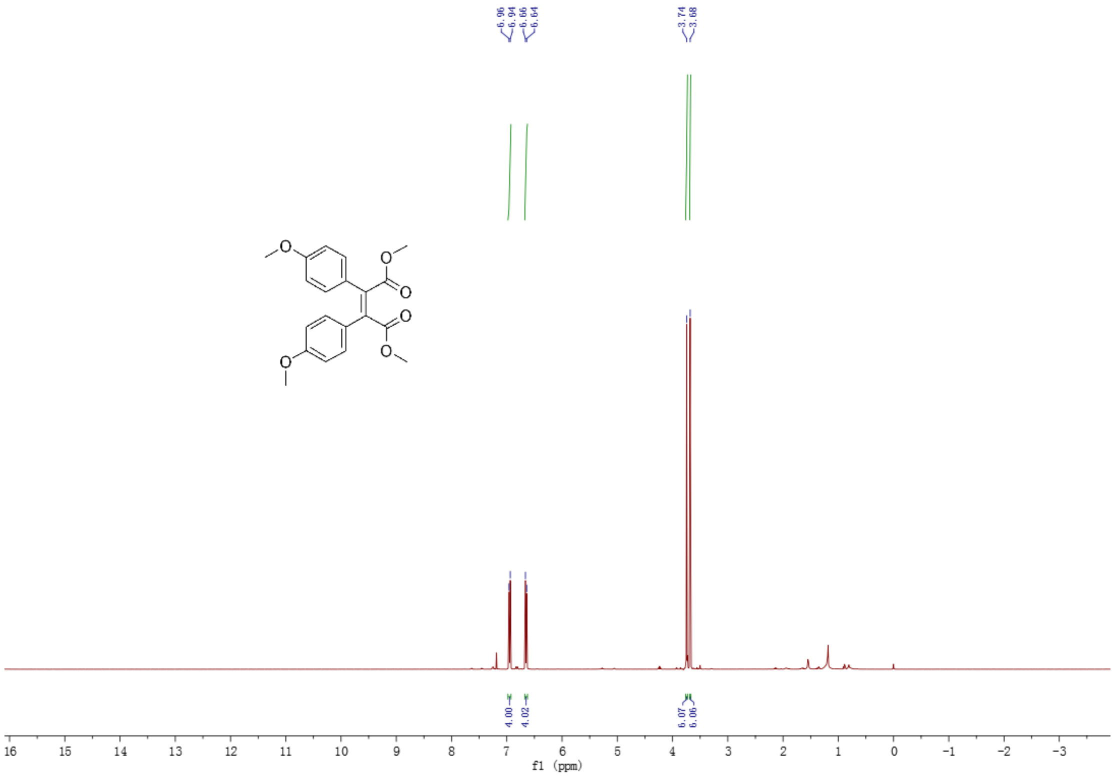

^{1}H NMR spectrum (400 MHz, $CDCl_3$) of **3k** (**E**)

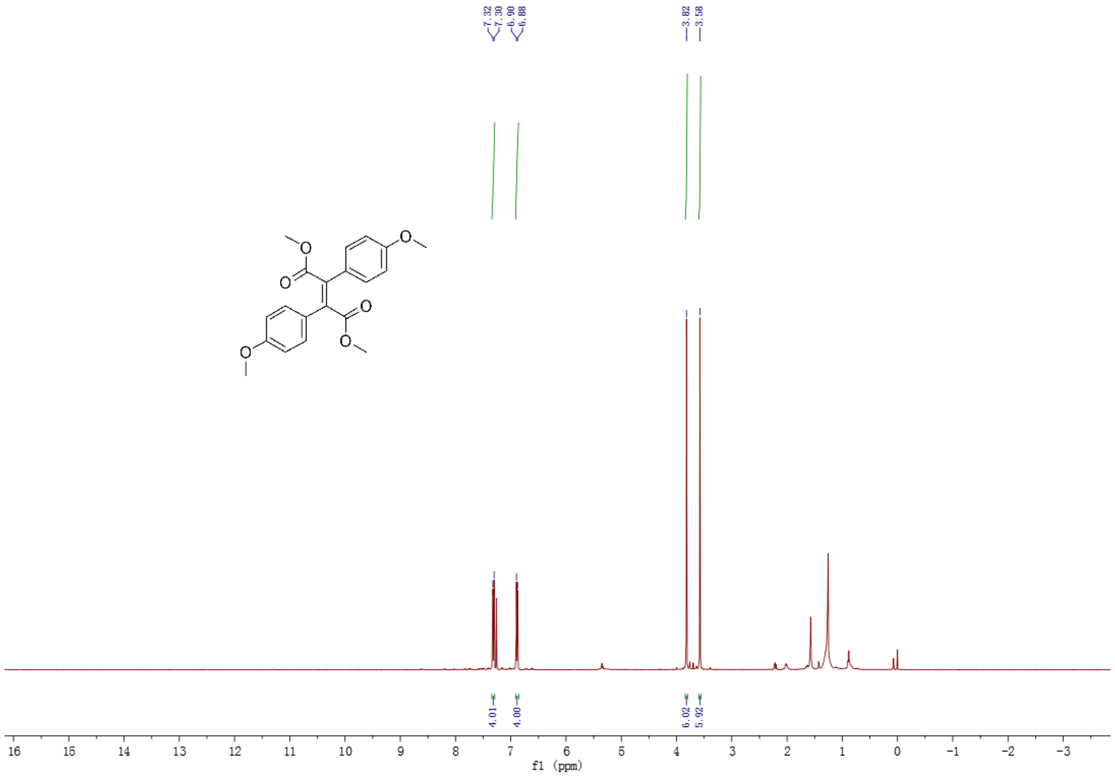

^{1}H NMR spectrum (400 MHz, $CDCl_3$) of **3l**

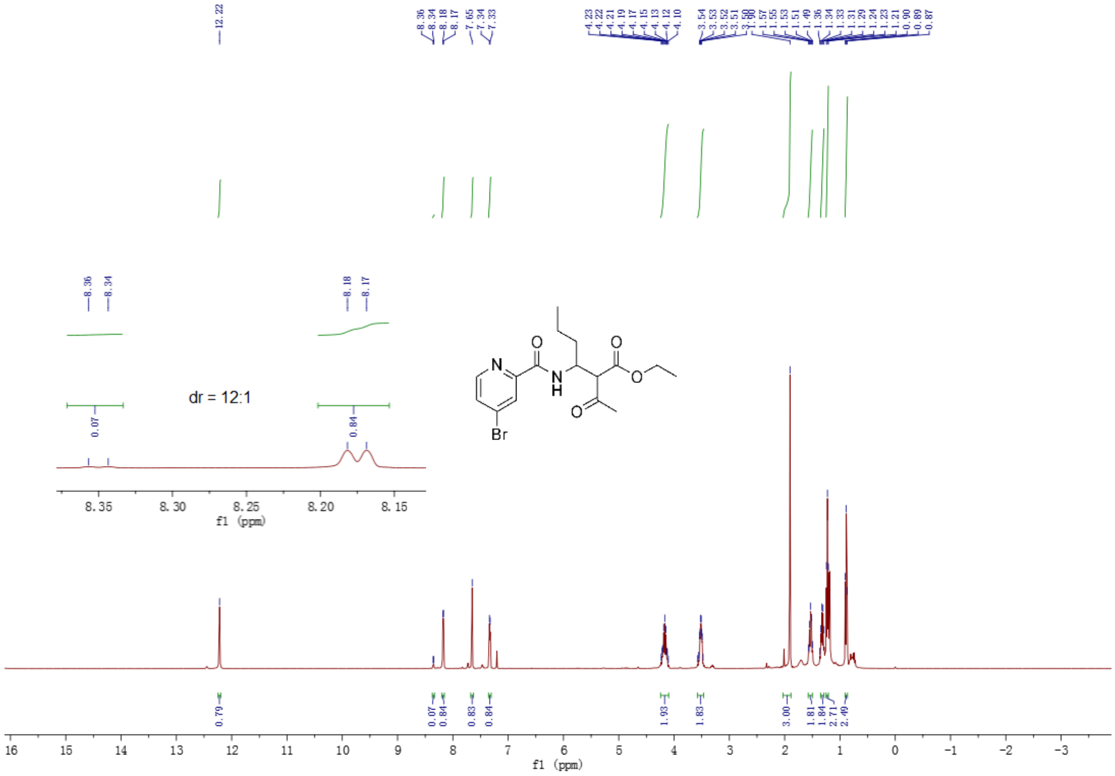

^{13}C NMR spectrum (1010 MHz, $CDCl_3$) of **3l**

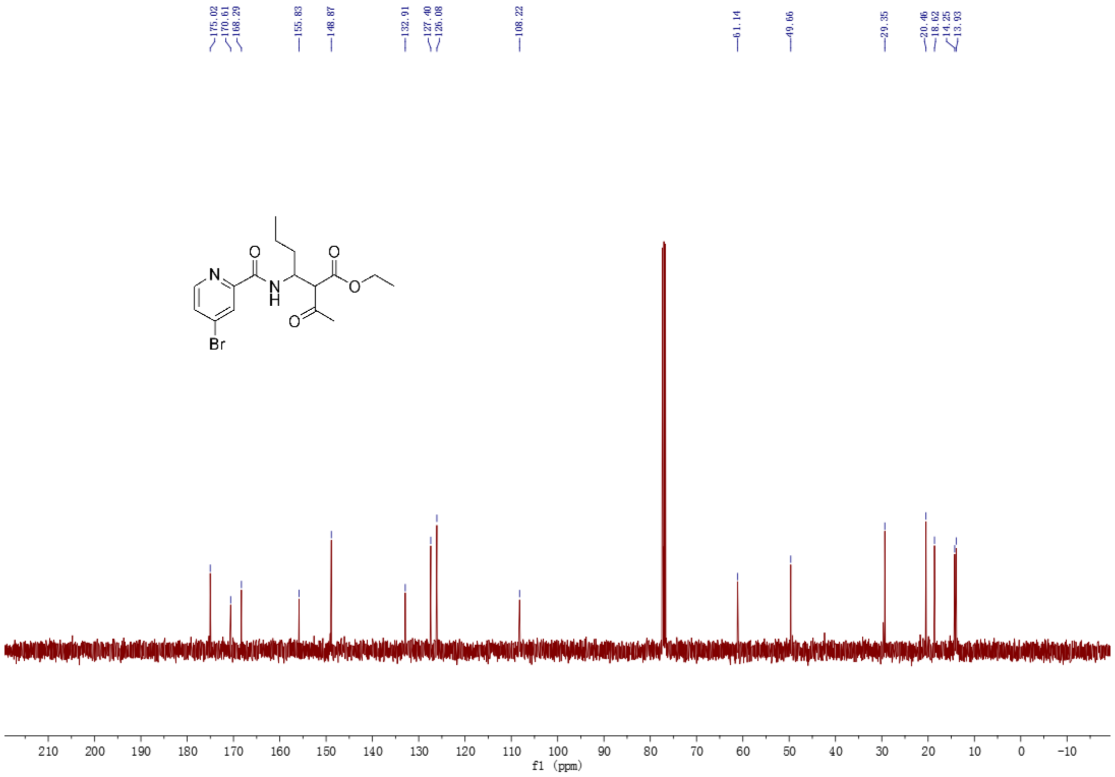

^{1}H NMR spectrum (400 MHz, $CDCl_3$) of **3n**

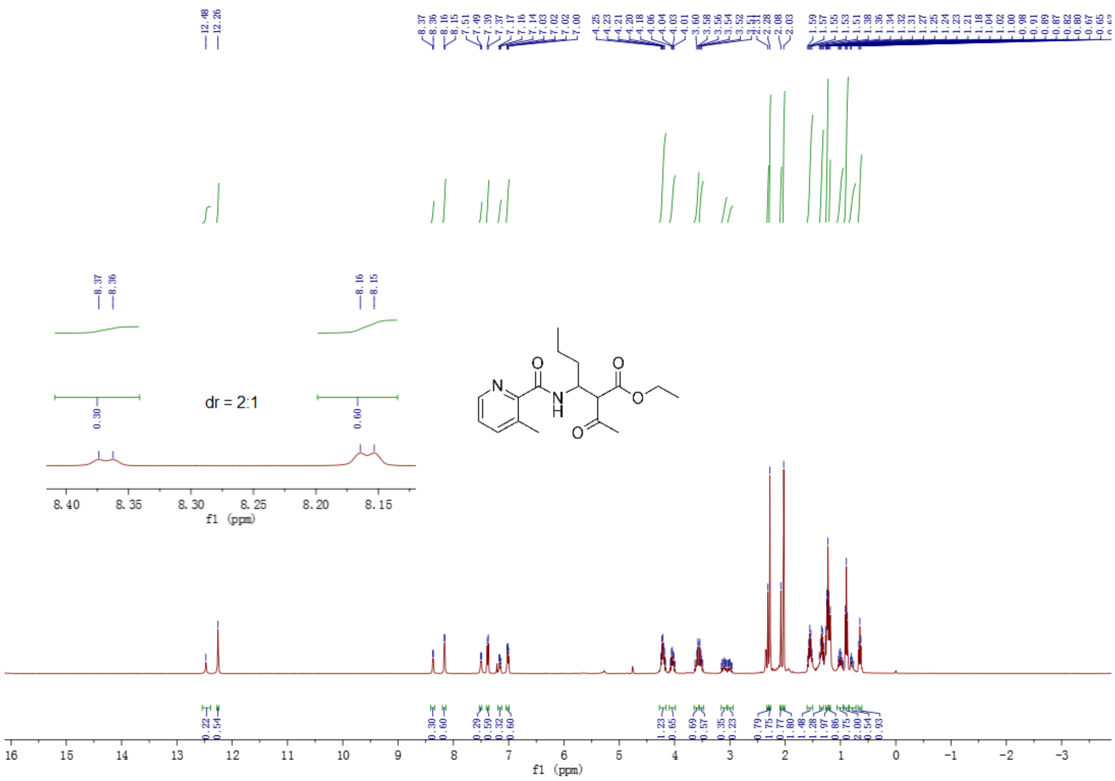

^{13}C NMR spectrum (100 MHz, $CDCl_3$) of **3n**

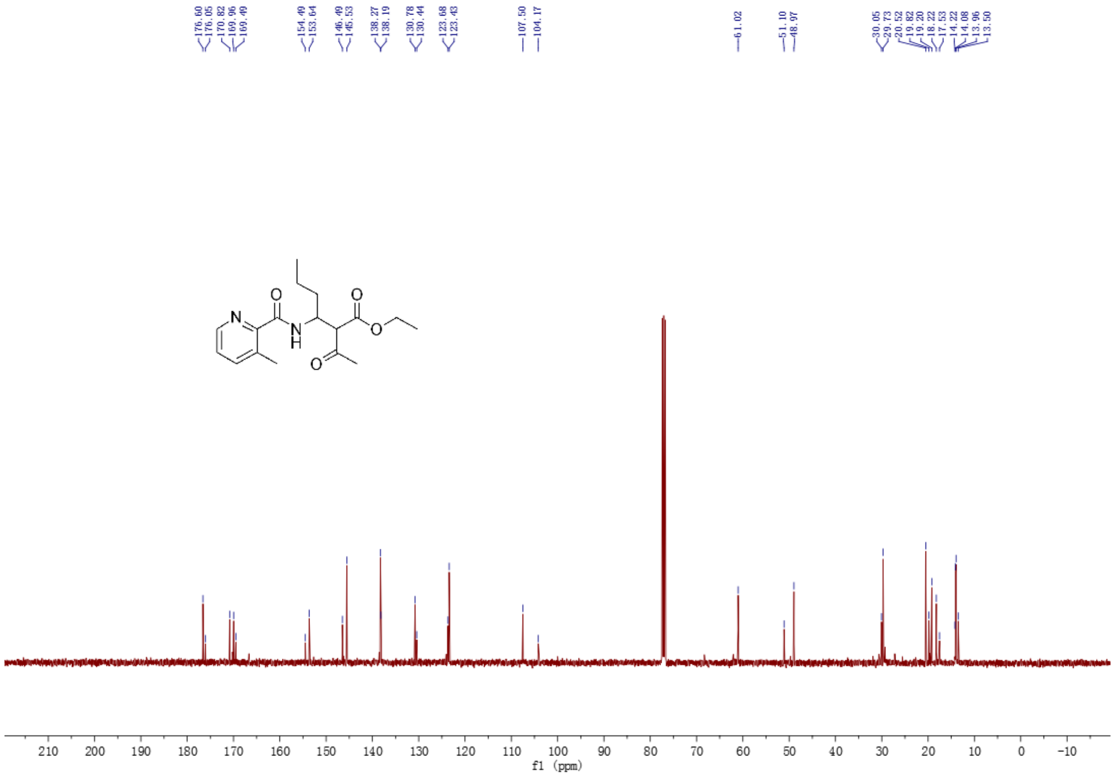

^{1}H NMR spectrum (400 MHz, $CDCl_3$) of **3o**

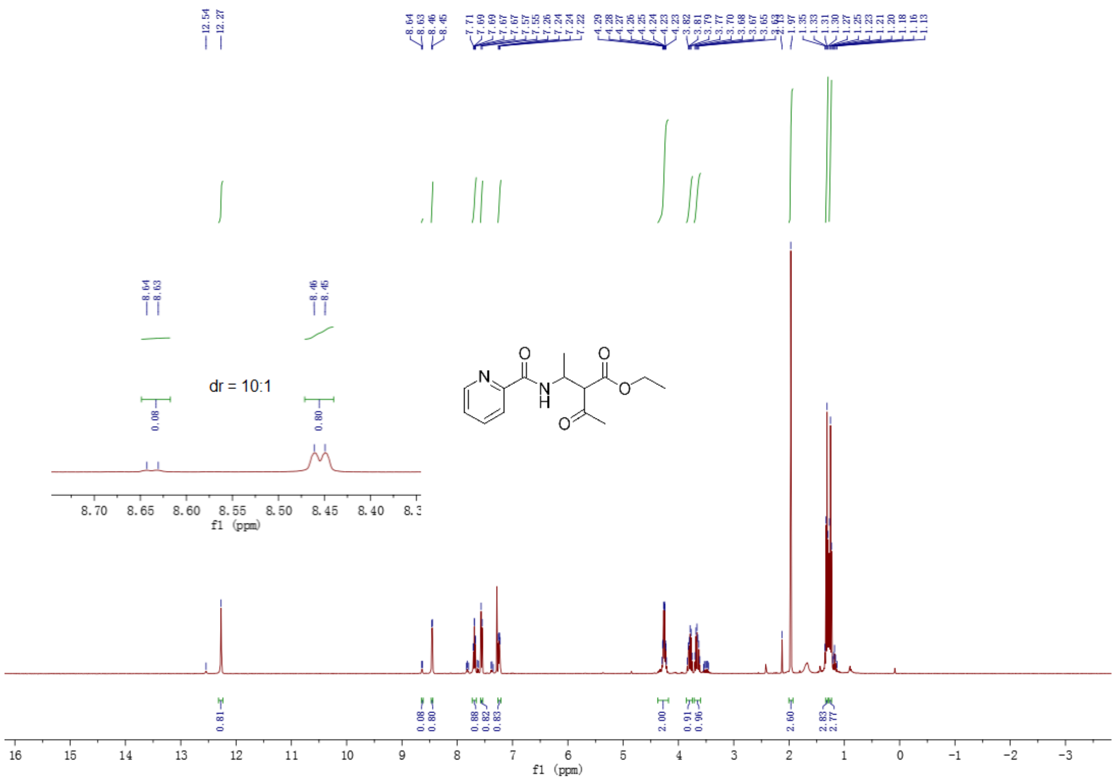

^{13}C NMR spectrum (100 MHz, $CDCl_3$) of **3o**

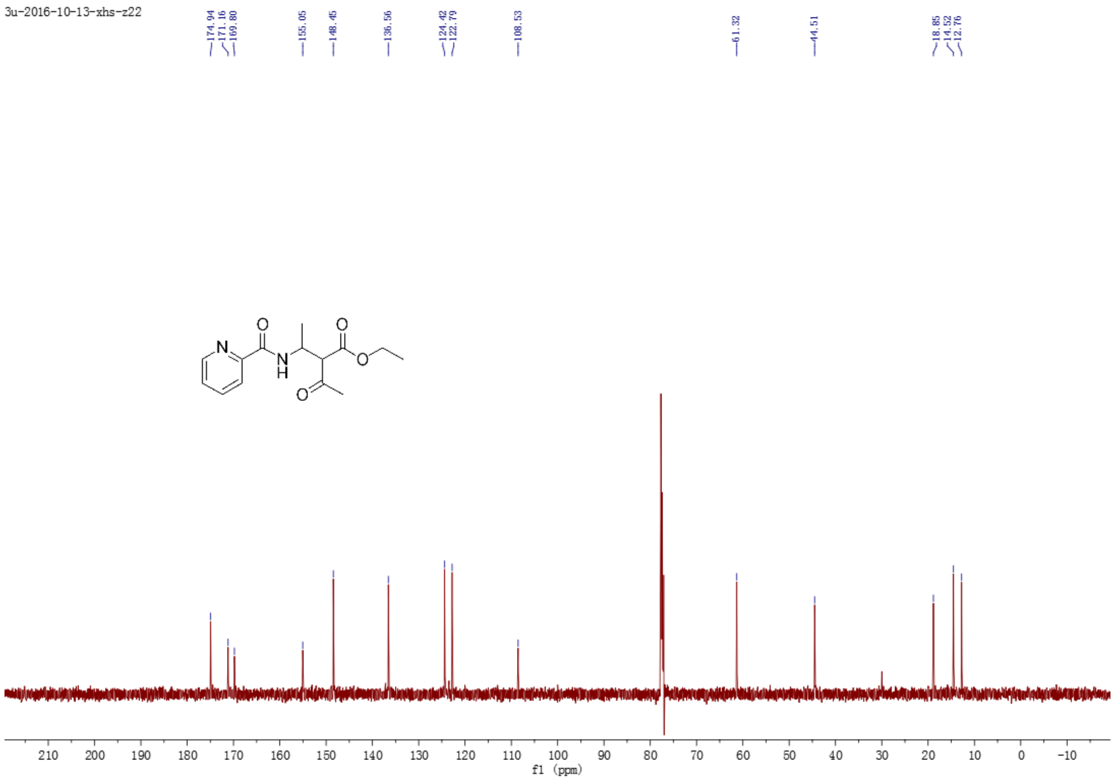

^{1}H NMR spectrum (400 MHz, $CDCl_3$) of **3p**

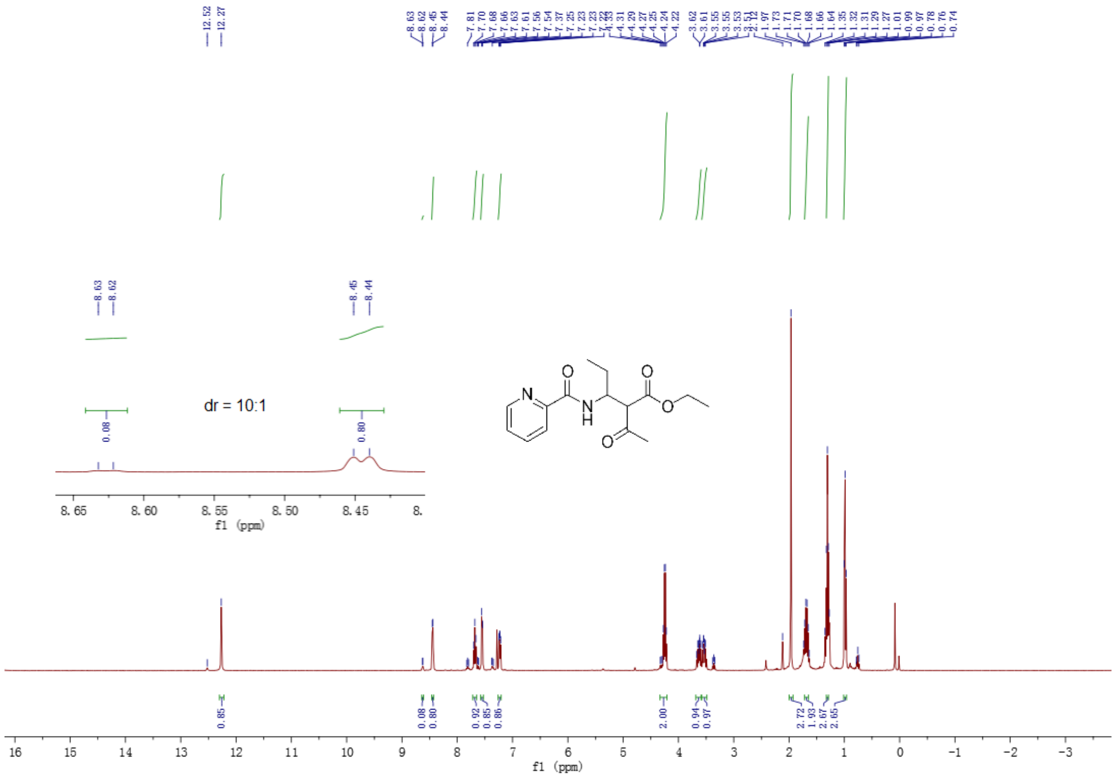

^{13}C NMR spectrum (100 MHz, $CDCl_3$) of **3p**

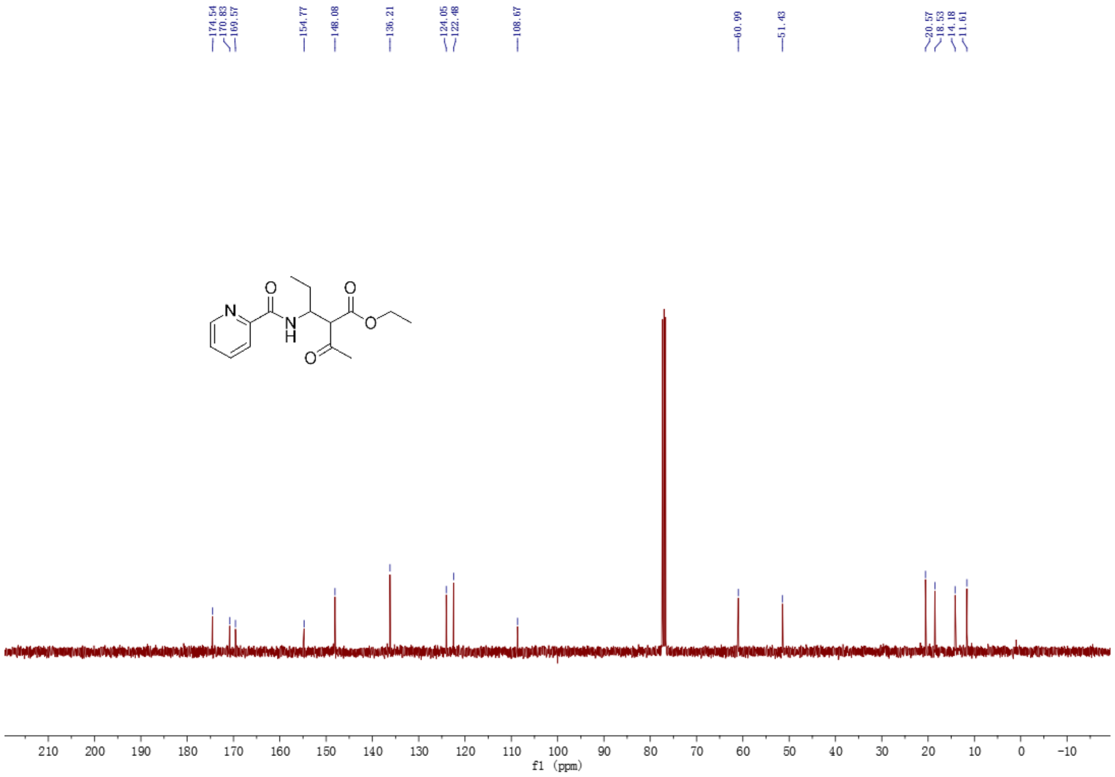

^{1}H NMR spectrum (400 MHz, CDCl$_3$) of **3q**

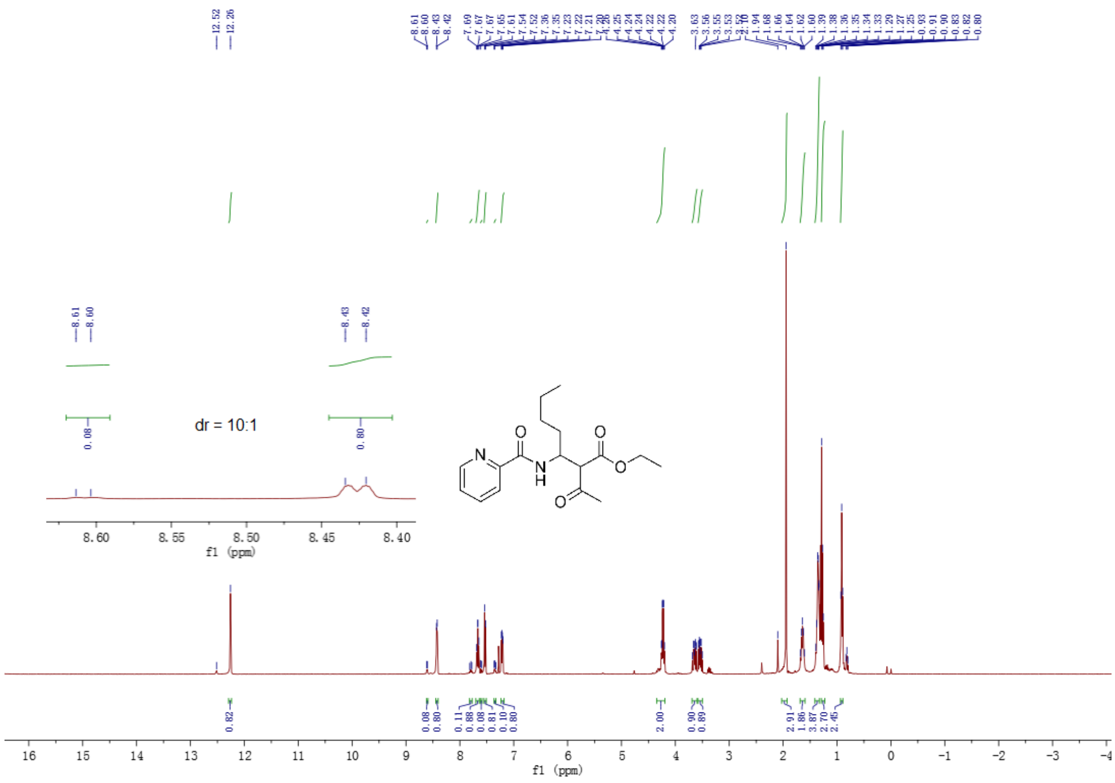

^{13}C NMR spectrum (100 MHz, CDCl$_3$) of **3q**

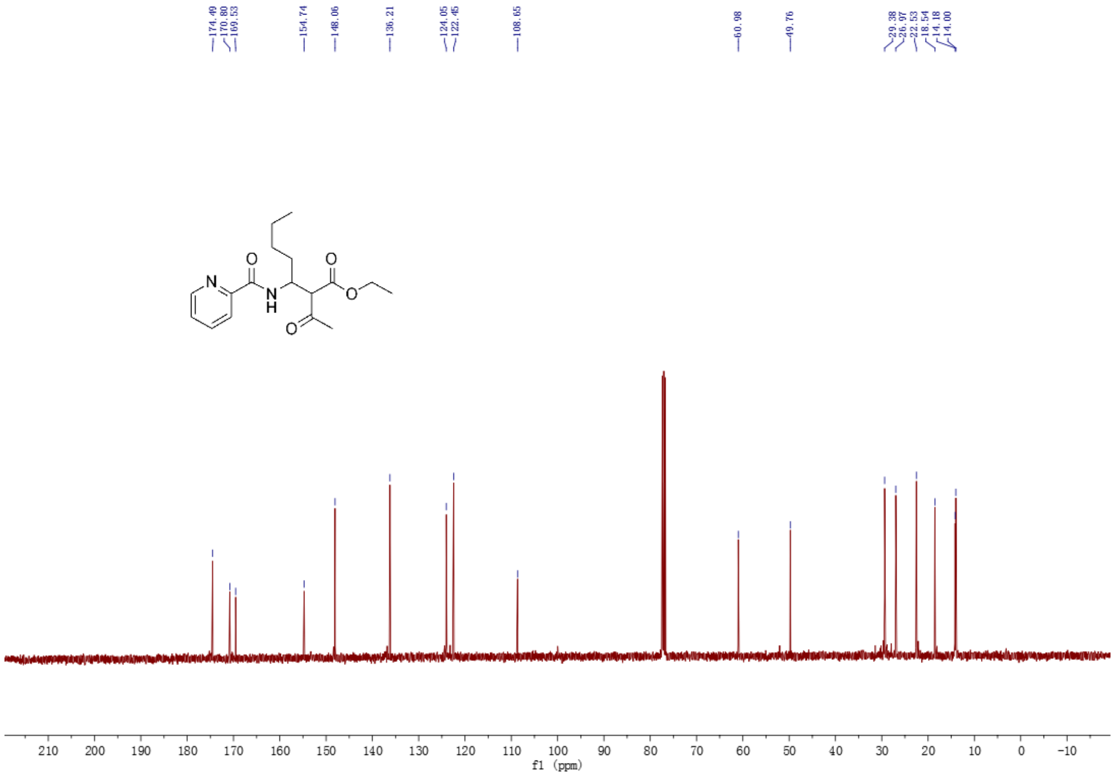

^{1}H NMR spectrum (400 MHz, $CDCl_3$) of **3r**

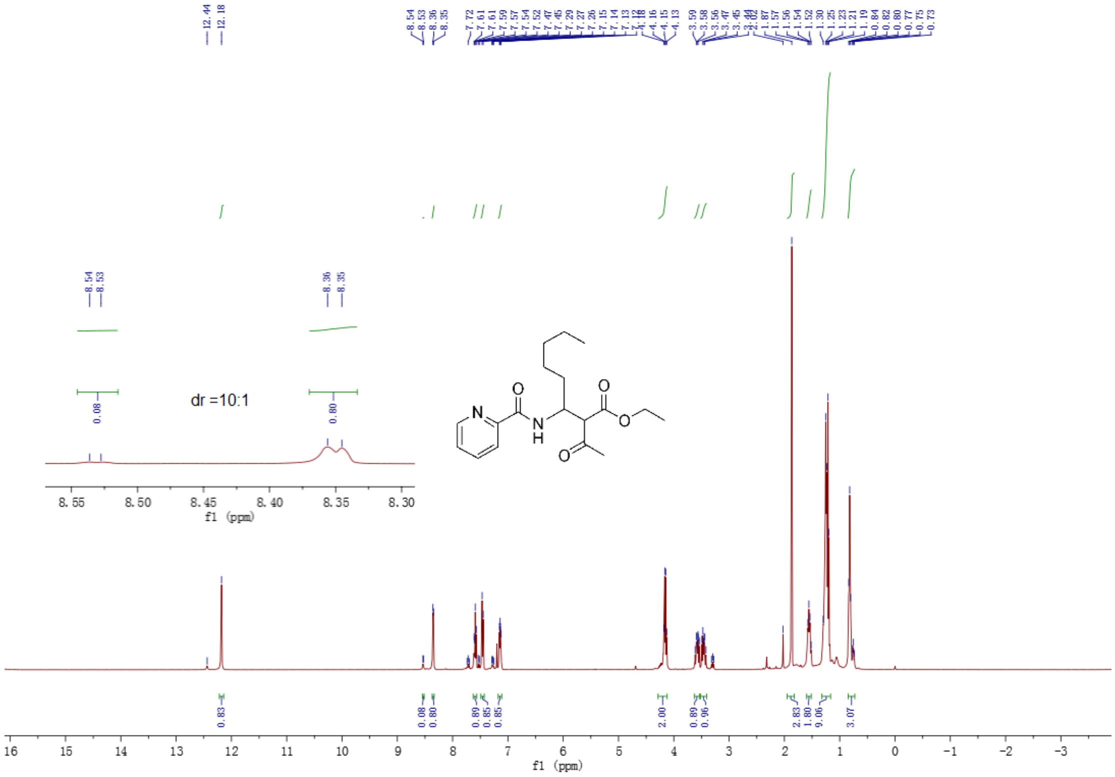

^{13}C NMR spectrum (100 MHz, $CDCl_3$) of **3r**

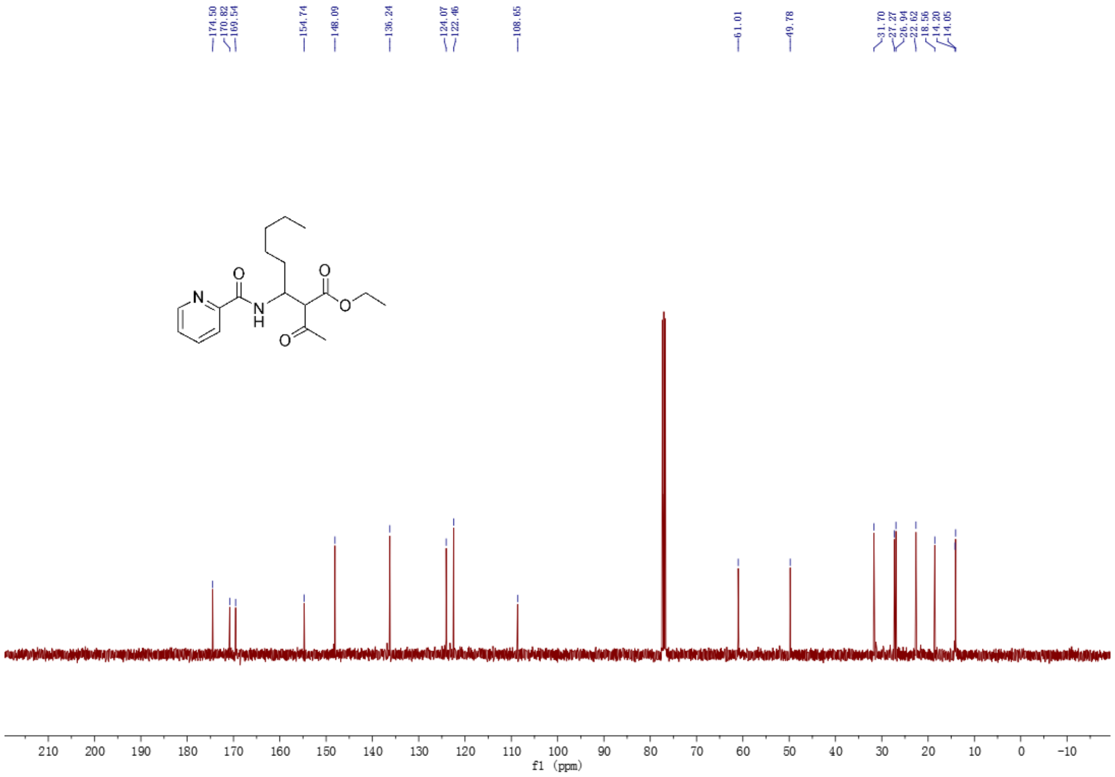

^{1}H NMR spectrum (400 MHz, $CDCl_3$) of **3s**

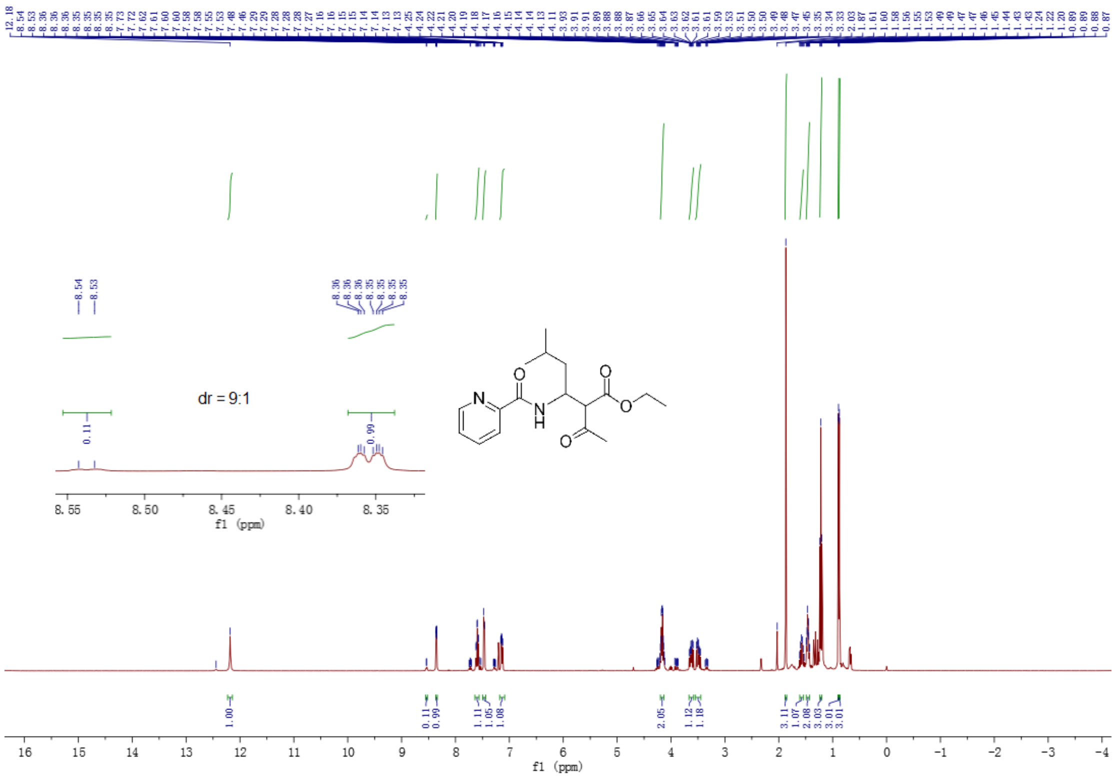

^{13}C NMR spectrum (100 MHz, $CDCl_3$) of **3s**

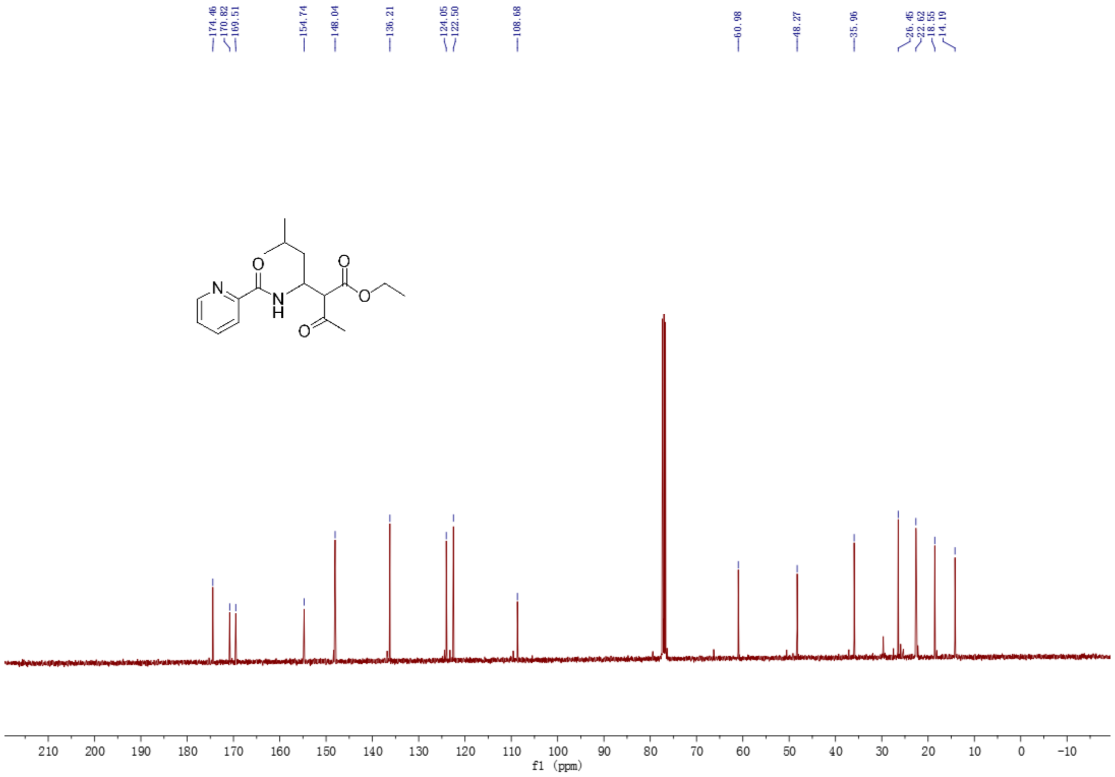

^{1}H NMR spectrum (400 MHz, $CDCl_3$) of **3t**

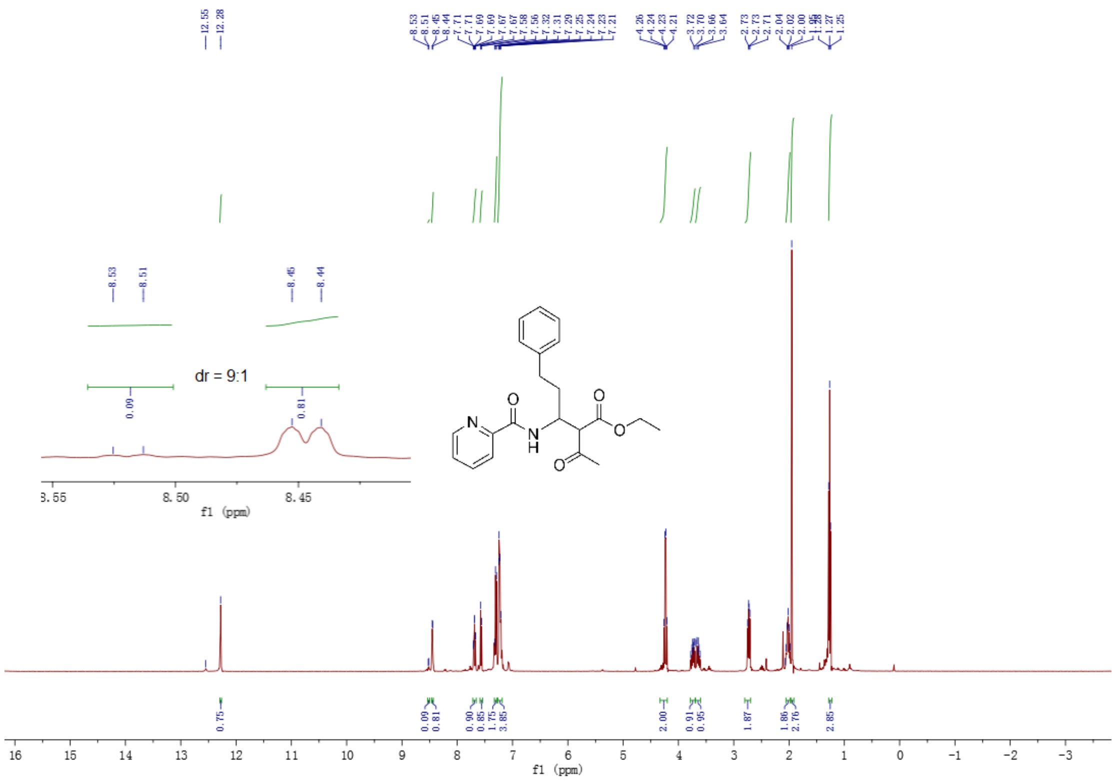

^{13}C NMR spectrum (100 MHz, $CDCl_3$) of **3t**

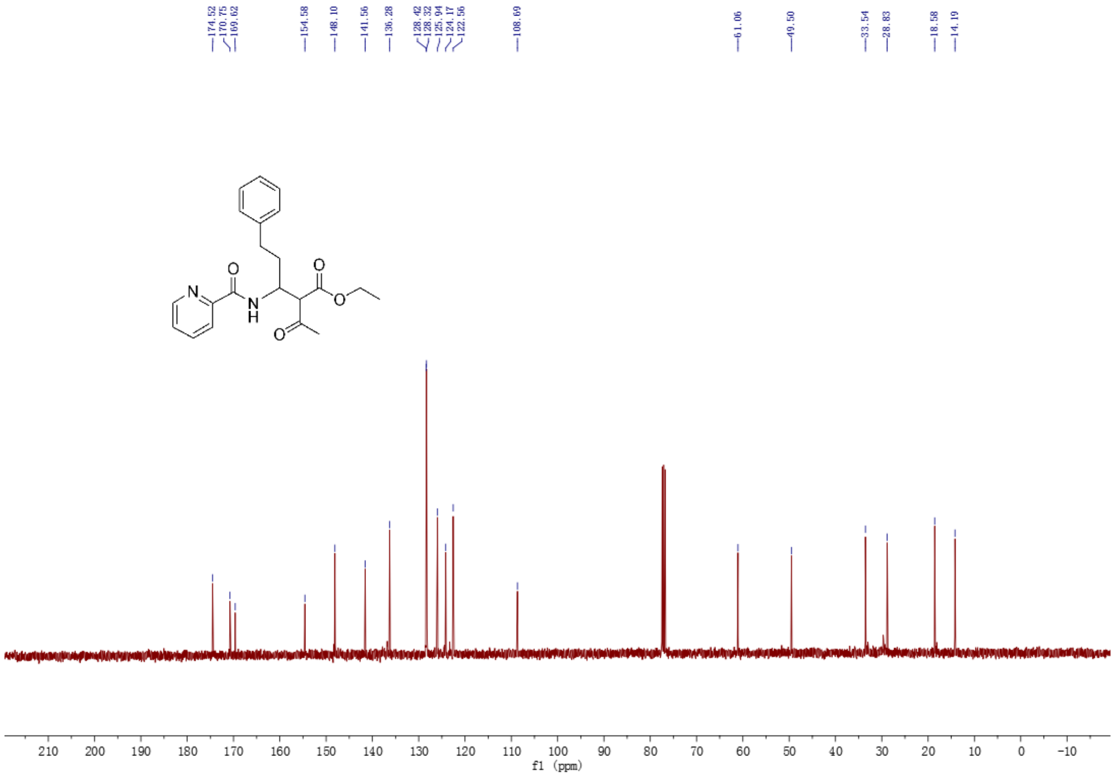

^{1}H NMR spectrum (400 MHz, $CDCl_3$) of **3u**

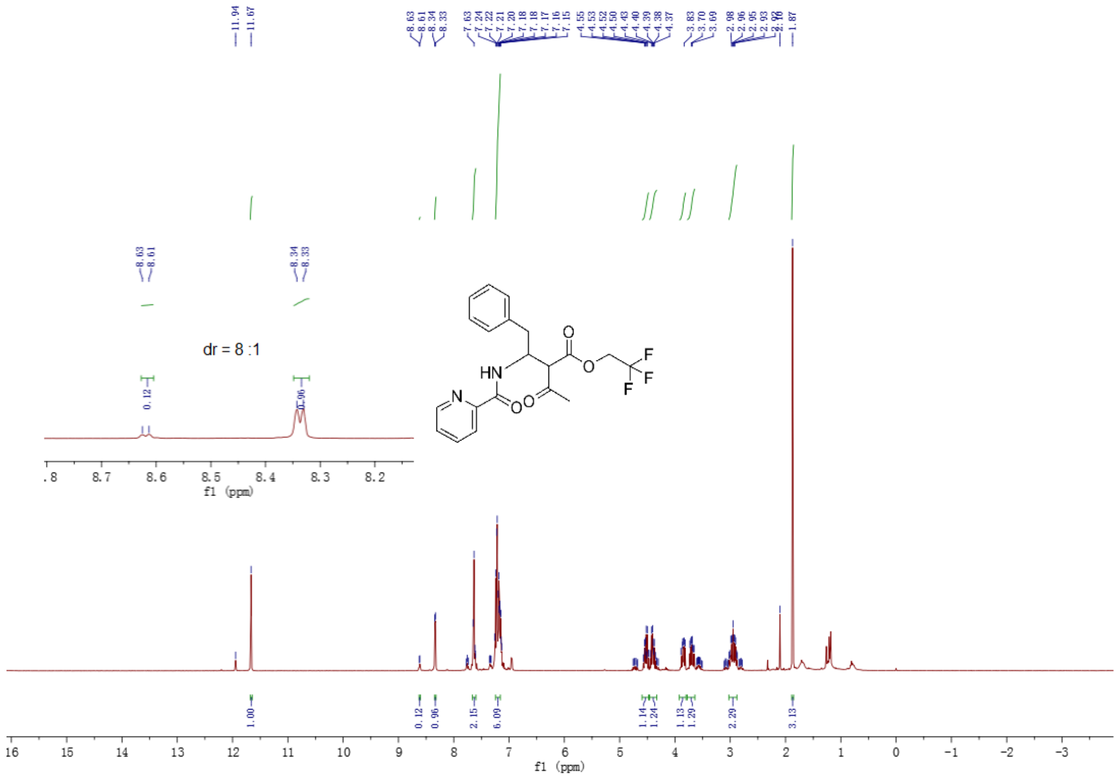

^{13}C NMR spectrum (100 MHz, $CDCl_3$) of **3u**

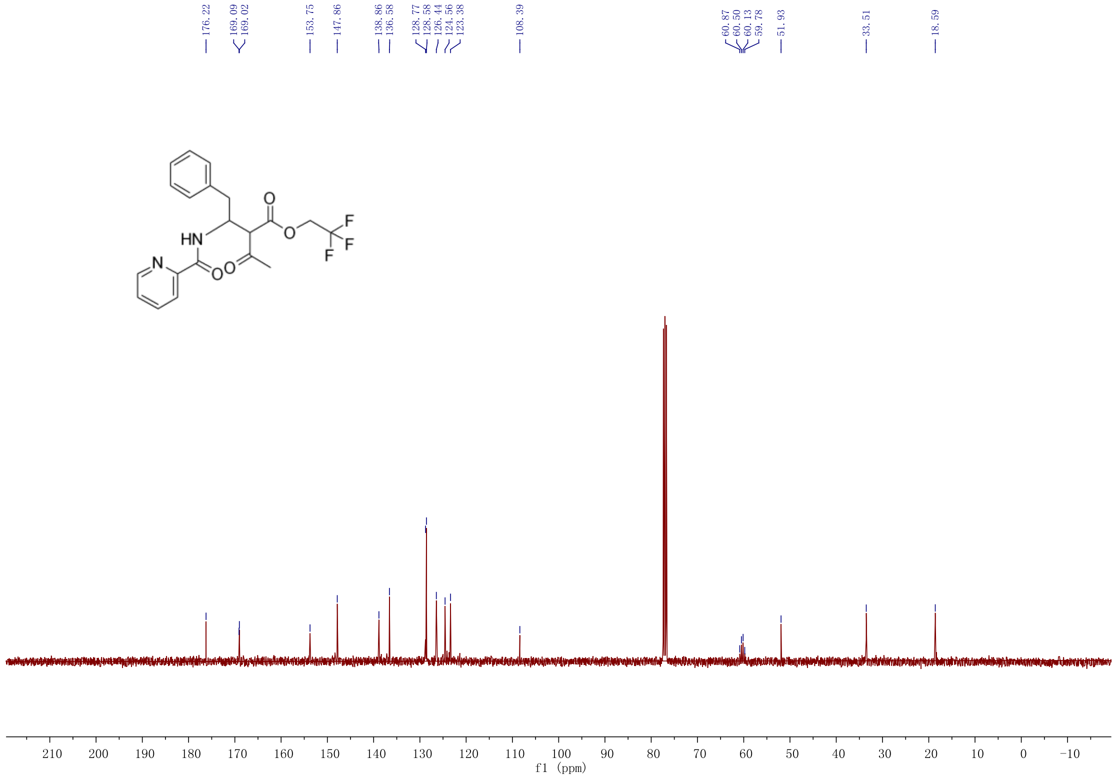

^{19}F NMR spectrum (376 MHz, $CDCl_3$) of **3u**

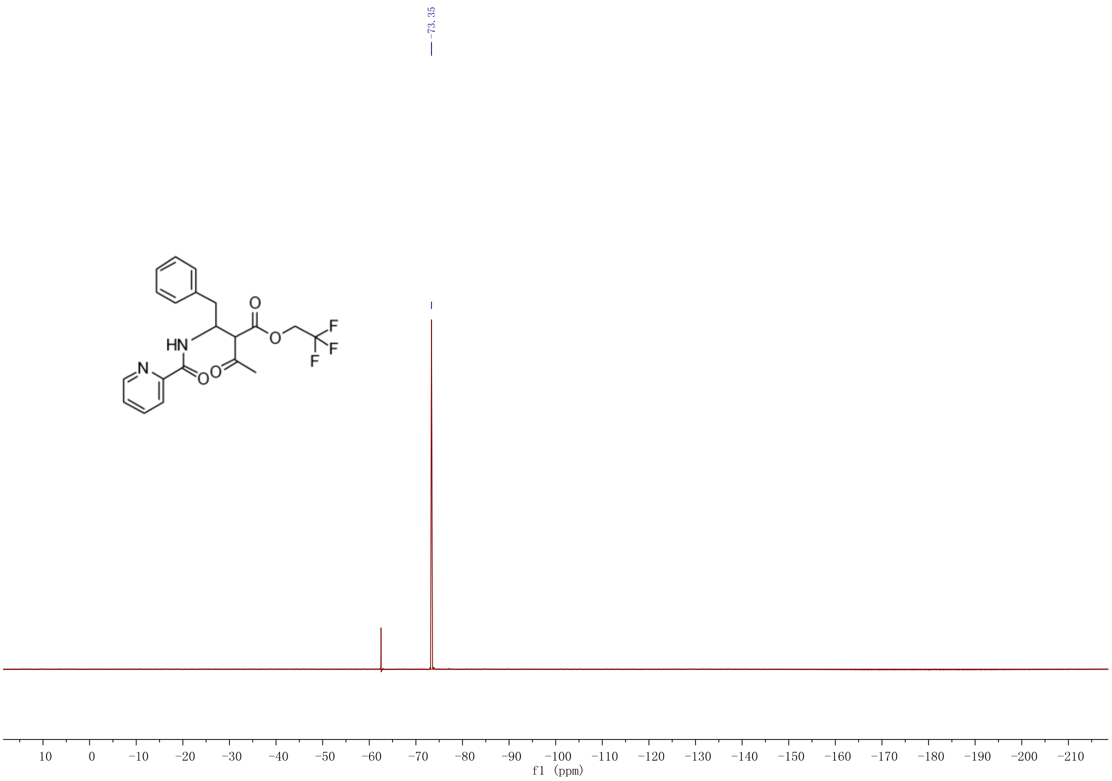

^{1}H NMR spectrum (400 MHz, $CDCl_3$) of **3v**

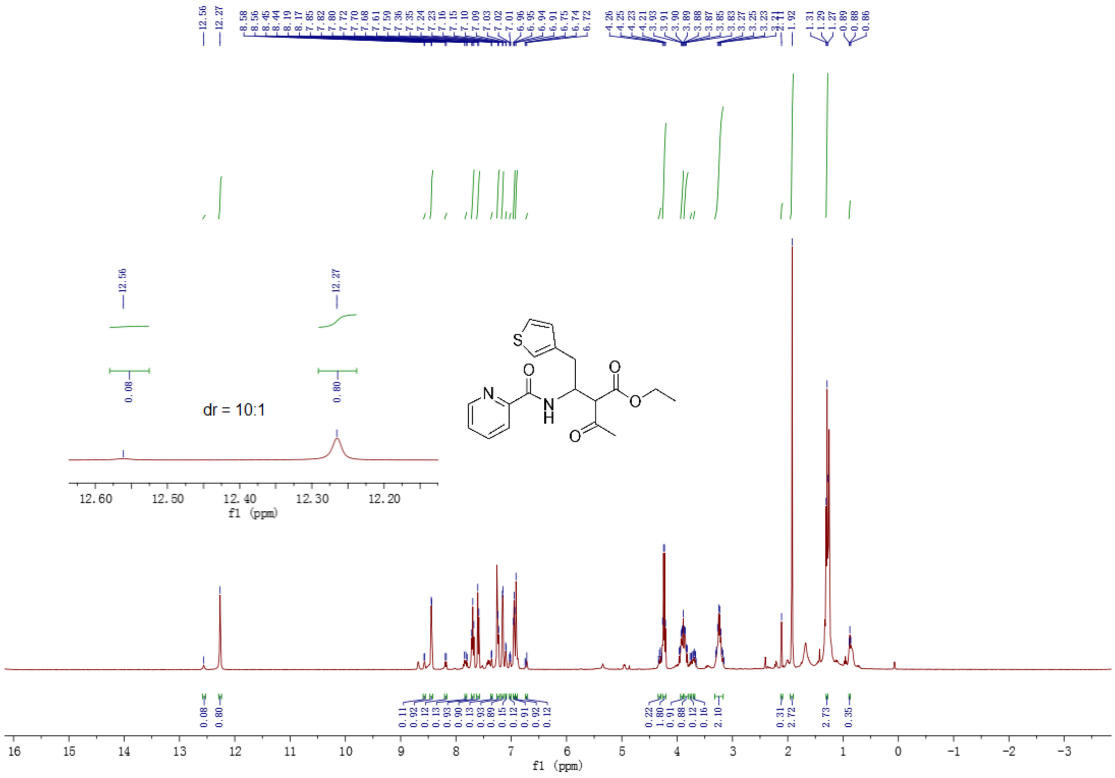

^{13}C NMR spectrum (100 MHz, $CDCl_3$) of **3v**

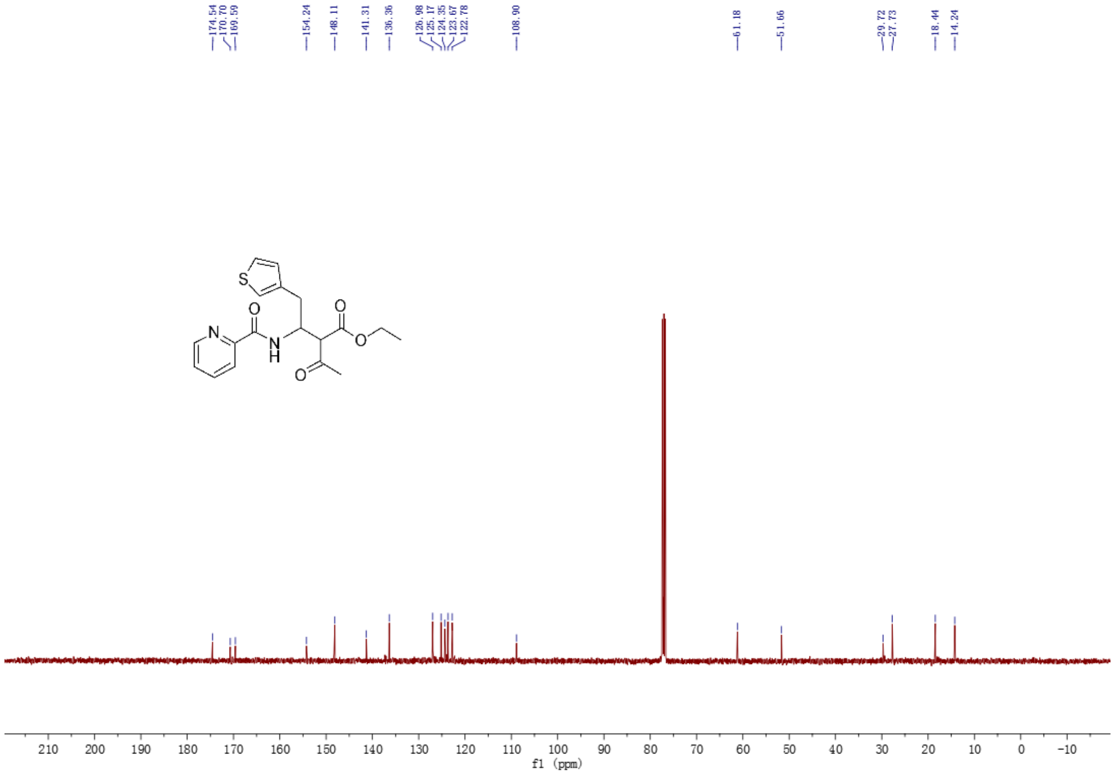

^{1}H NMR spectrum (400 MHz, $CDCl_3$) of **3w**

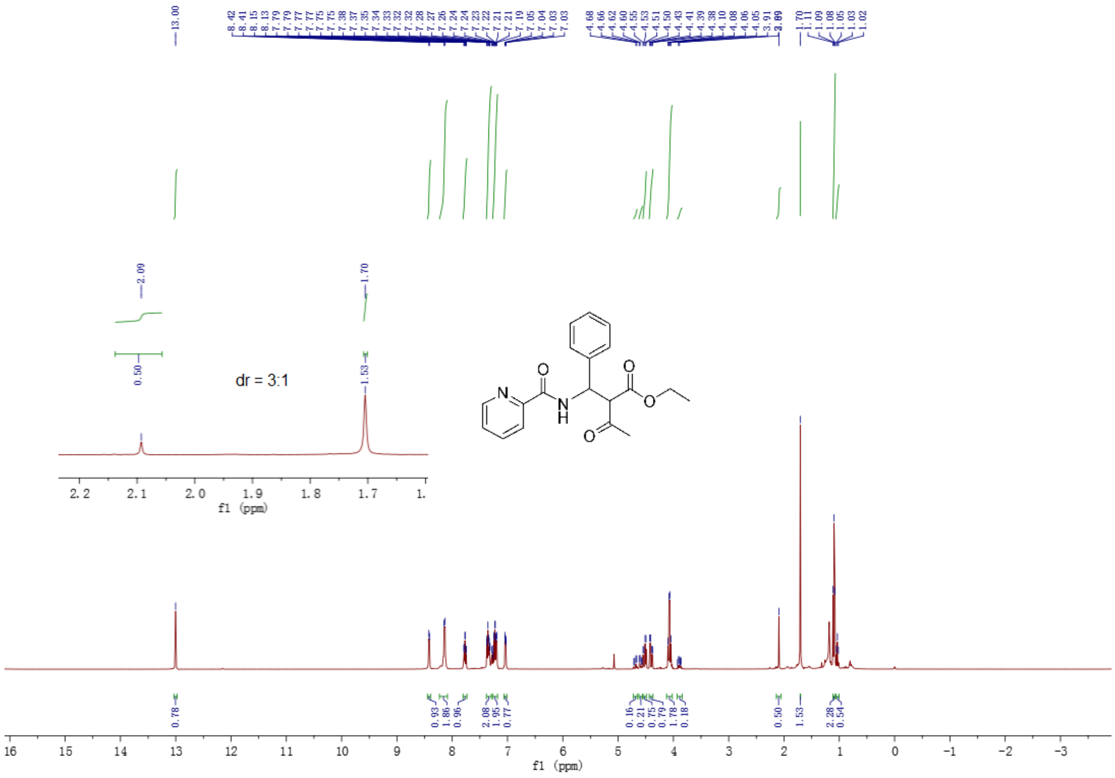

^{13}C NMR spectrum (100 MHz, $CDCl_3$) of **3w**

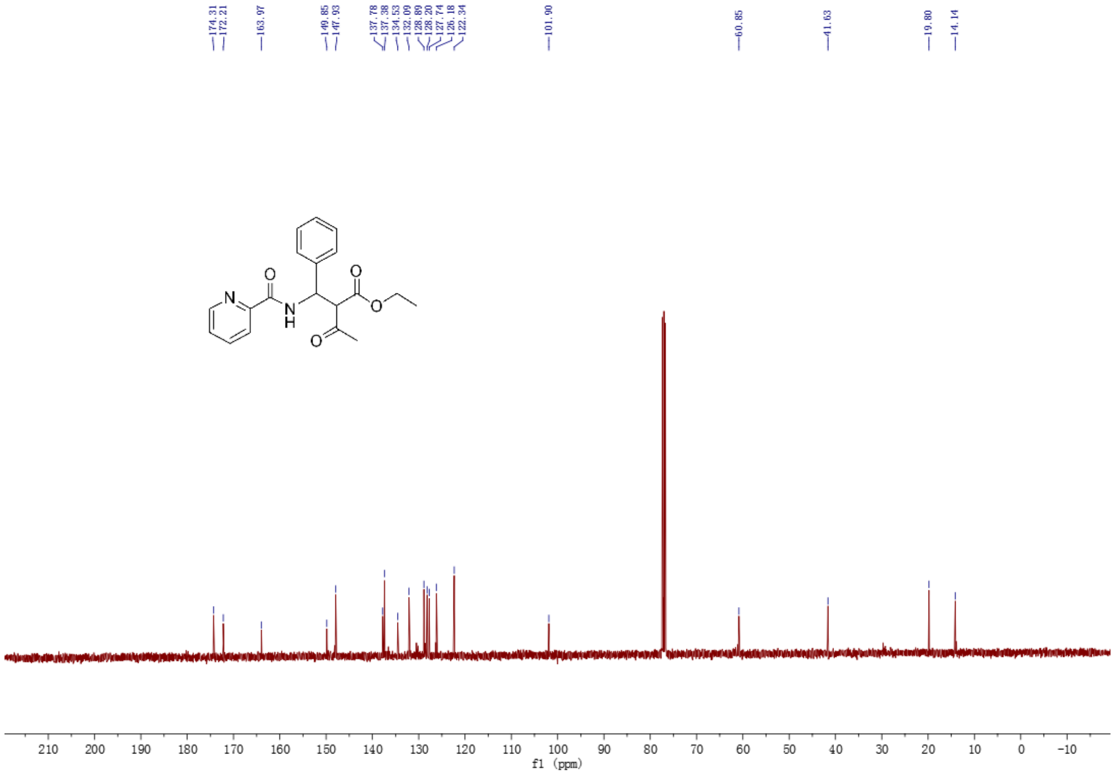

^{1}H NMR spectrum (400 MHz, $CDCl_3$) of **3x**

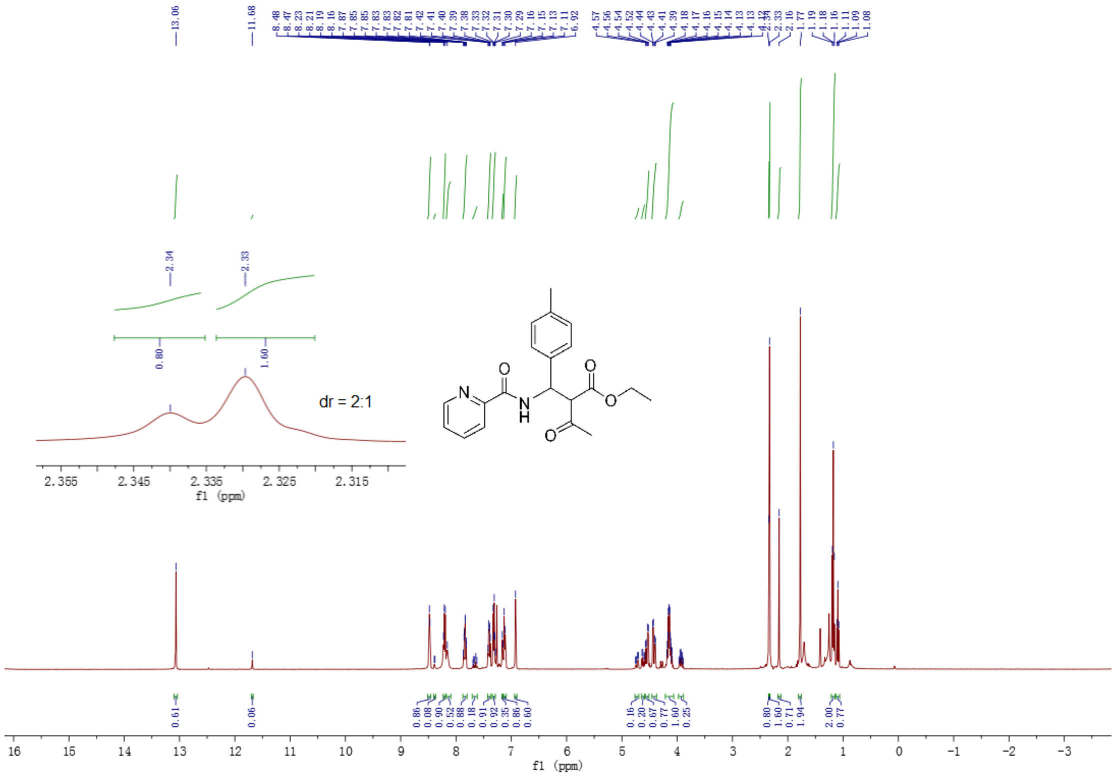

^{13}C NMR spectrum (100 MHz, $CDCl_3$) of **3x**

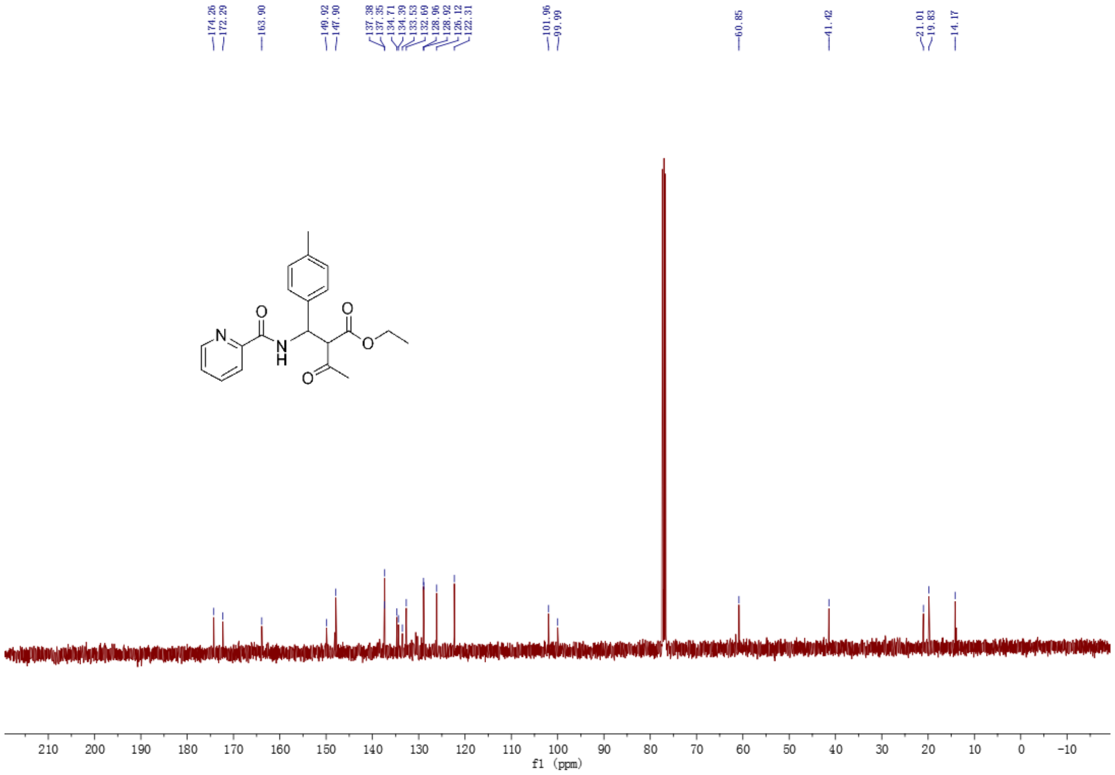

^{1}H NMR spectrum (400 MHz, $CDCl_3$) of **3y**

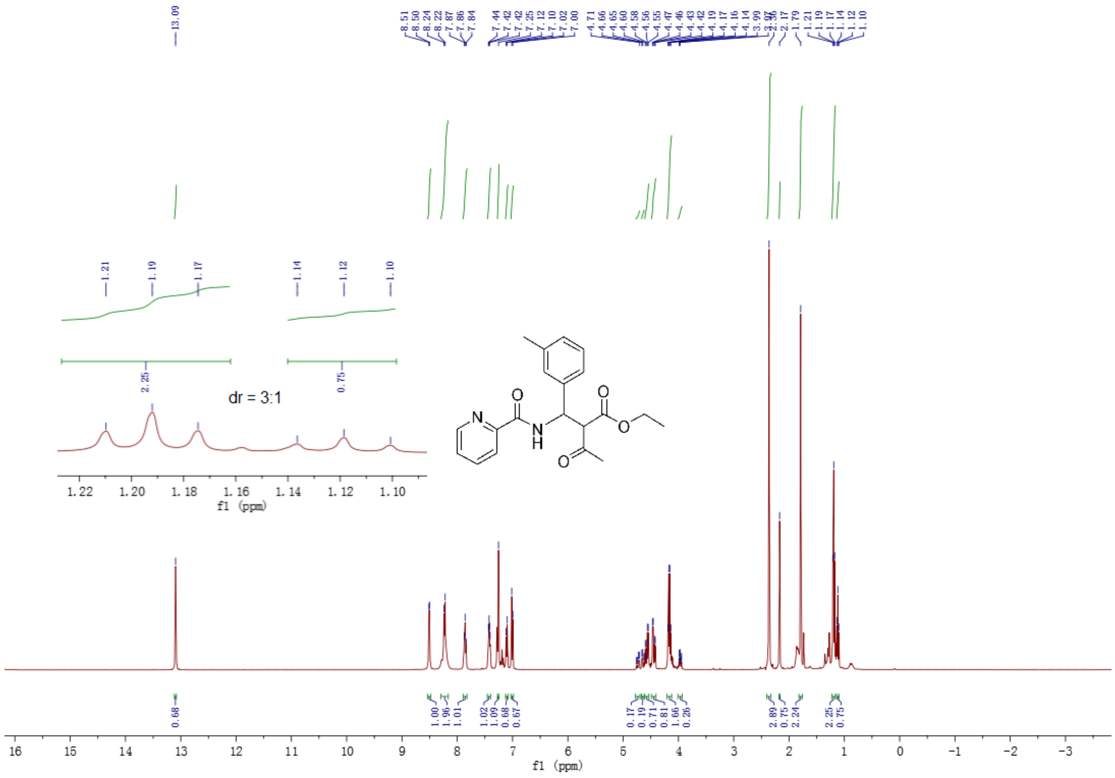

^{13}C NMR spectrum (100 MHz, $CDCl_3$) of **3y**

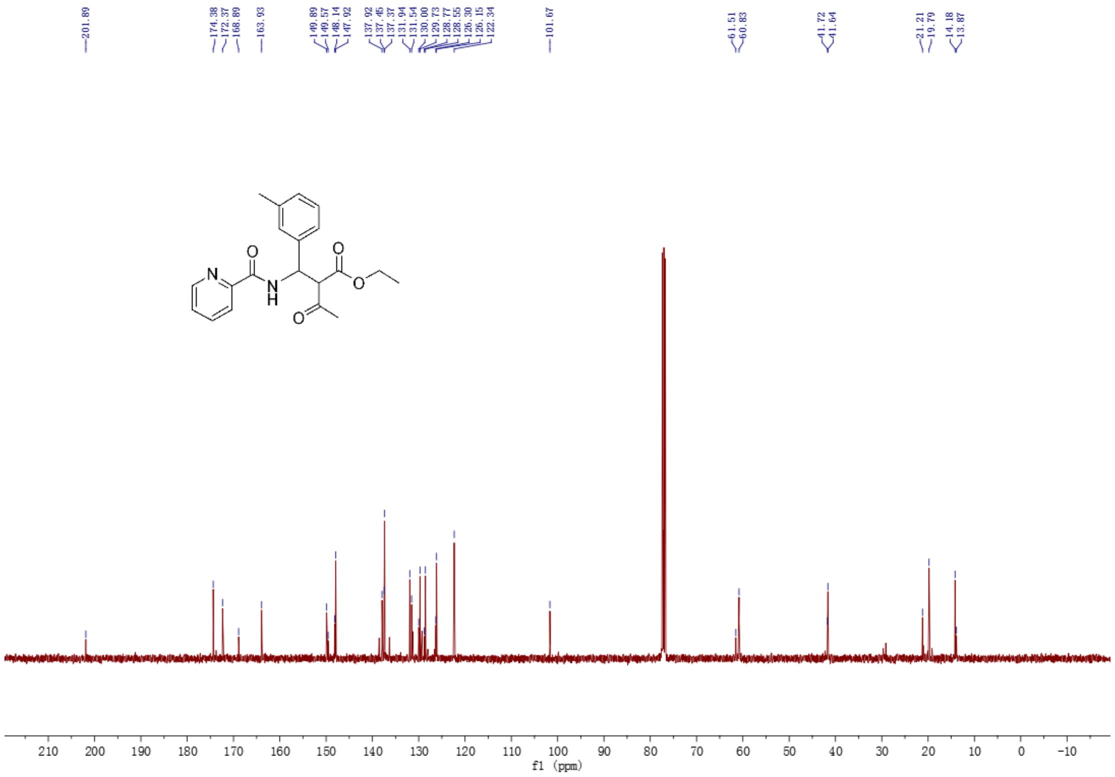

^{1}H NMR spectrum (400 MHz, $CDCl_3$) of **3z**

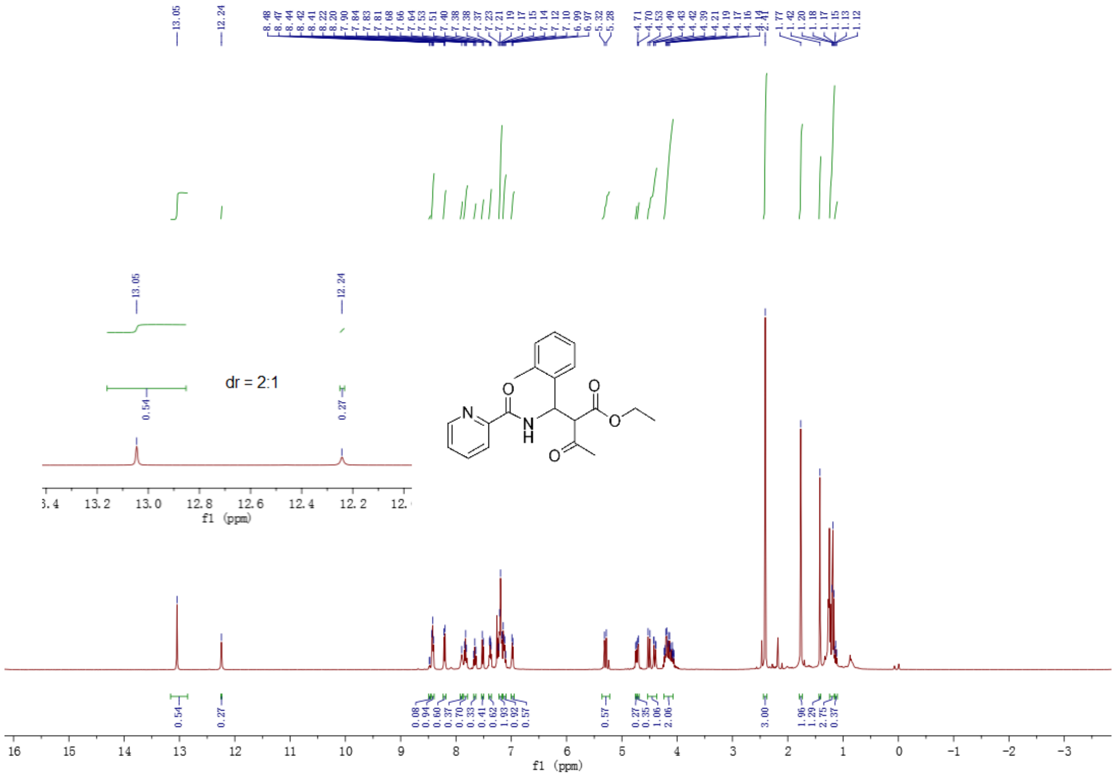

^{13}C NMR spectrum (100 MHz, $CDCl_3$) of **3z**

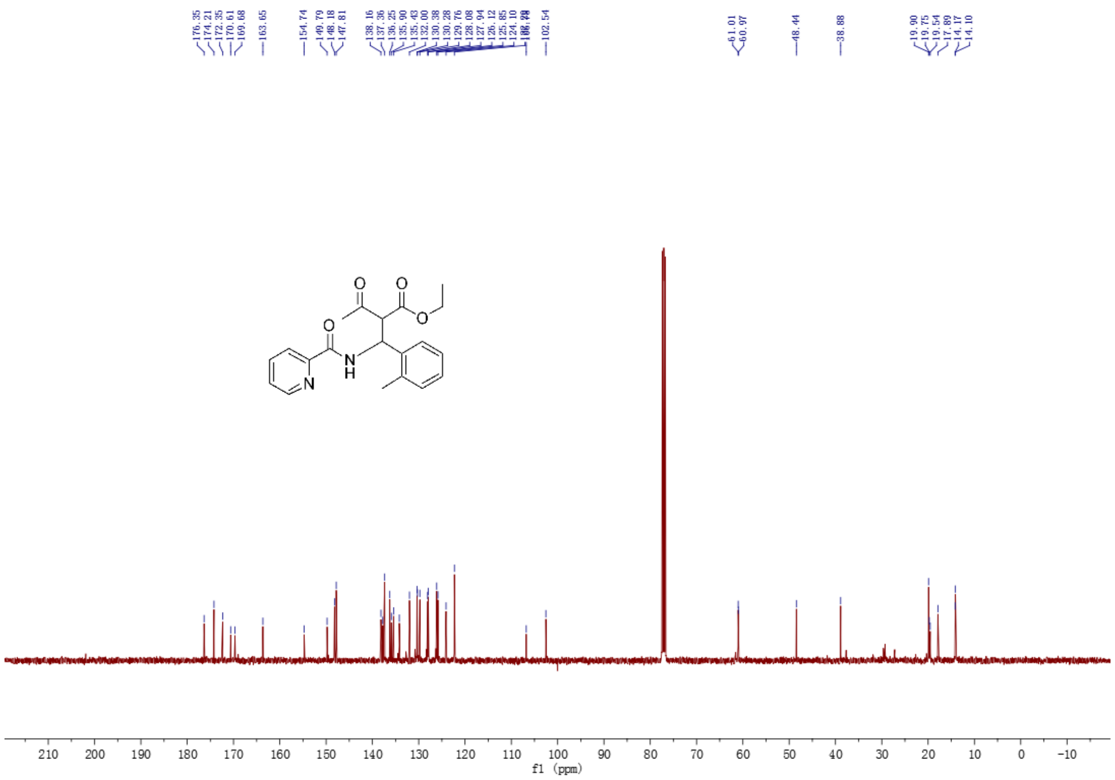

^{1}H NMR spectrum (400 MHz, $CDCl_3$) of **3-1a**

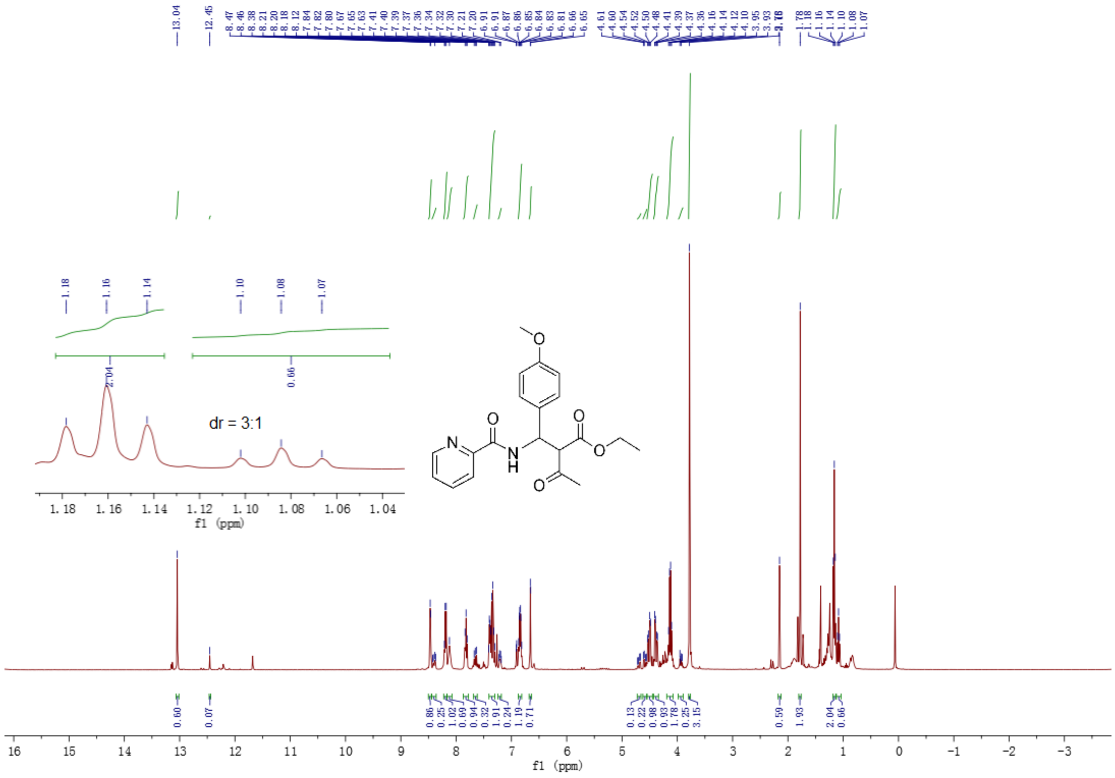

^{13}C NMR spectrum (100 MHz, $CDCl_3$) of **3-1a**

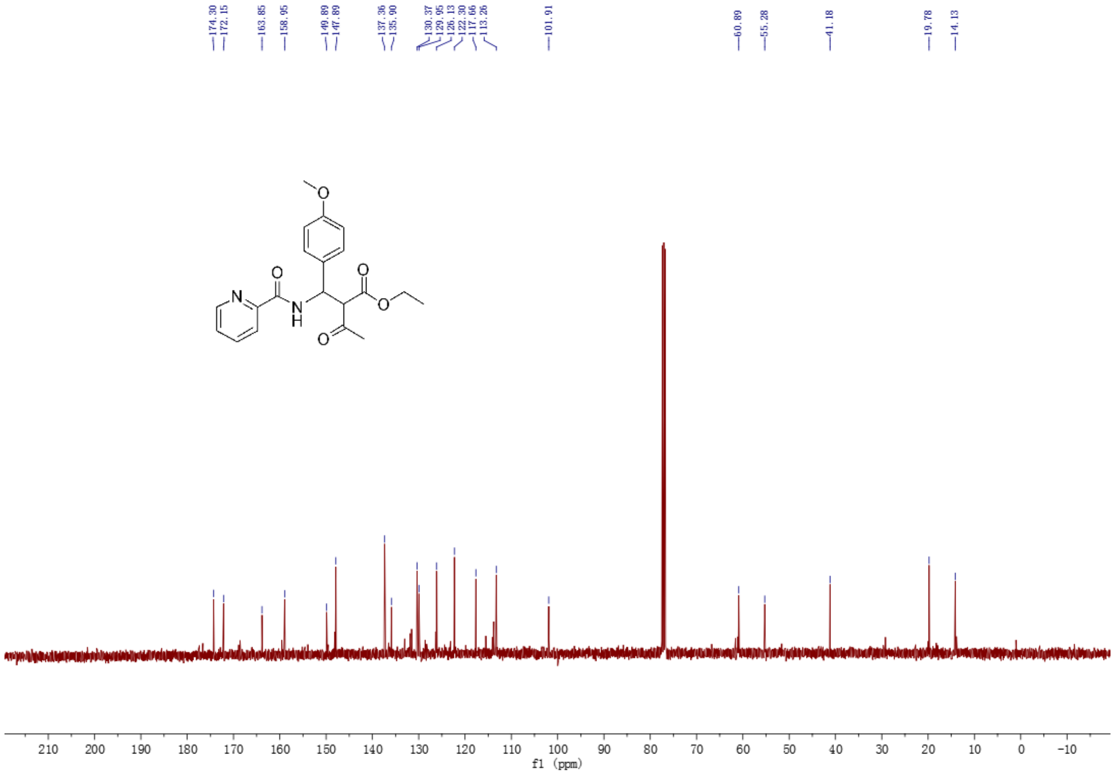

^{1}H NMR spectrum (400 MHz, $CDCl_3$) of **3-1b**

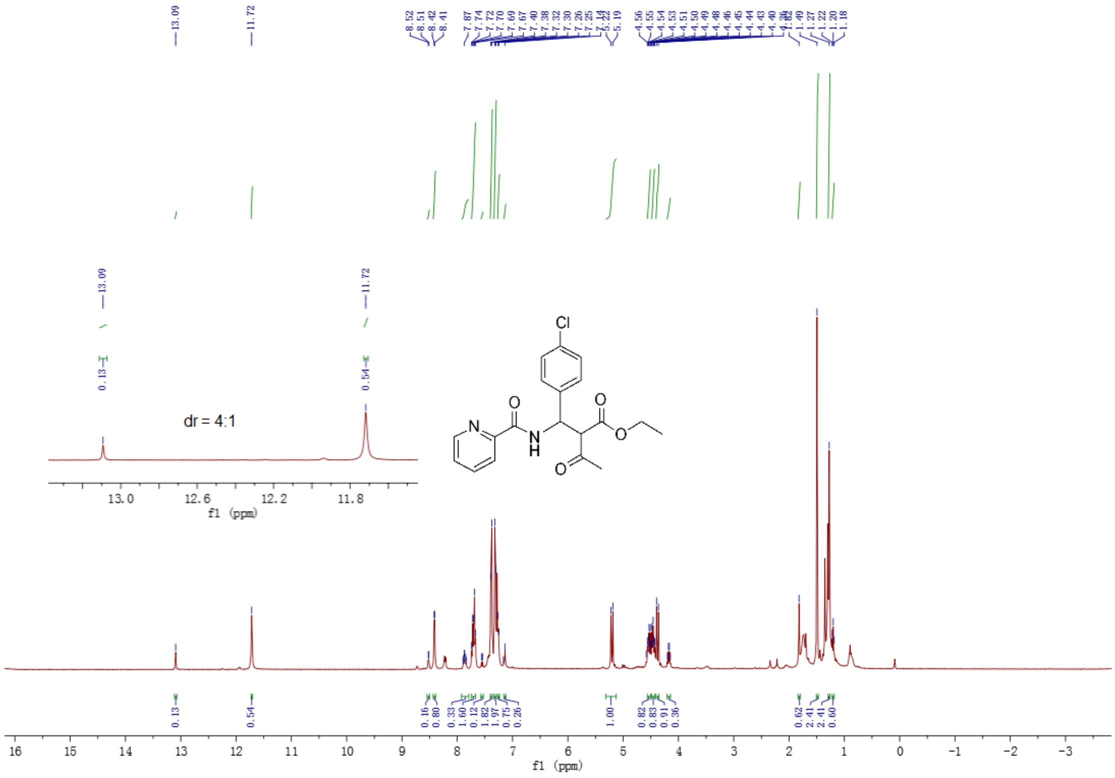

^{13}C NMR spectrum (100 MHz, $CDCl_3$) of **3-1b**

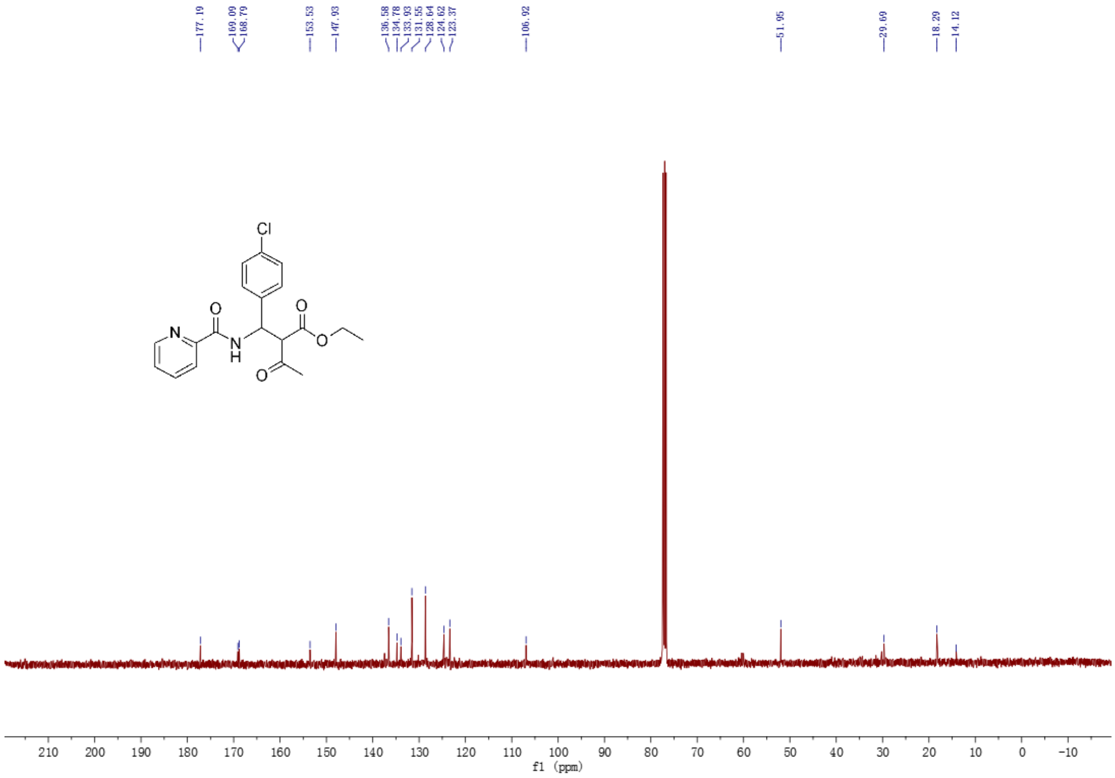

^{1}H NMR spectrum (400 MHz, $CDCl_3$) of **3-1c**

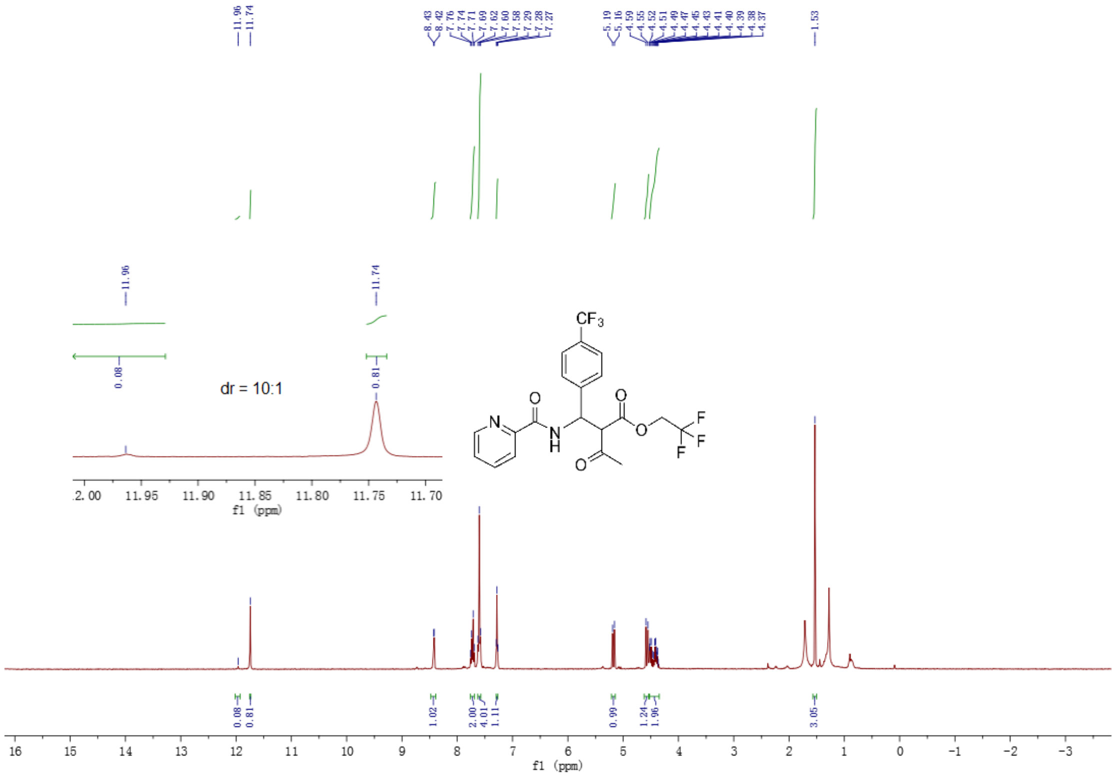

^{13}C NMR spectrum (100 MHz, $CDCl_3$) of **3-1c**

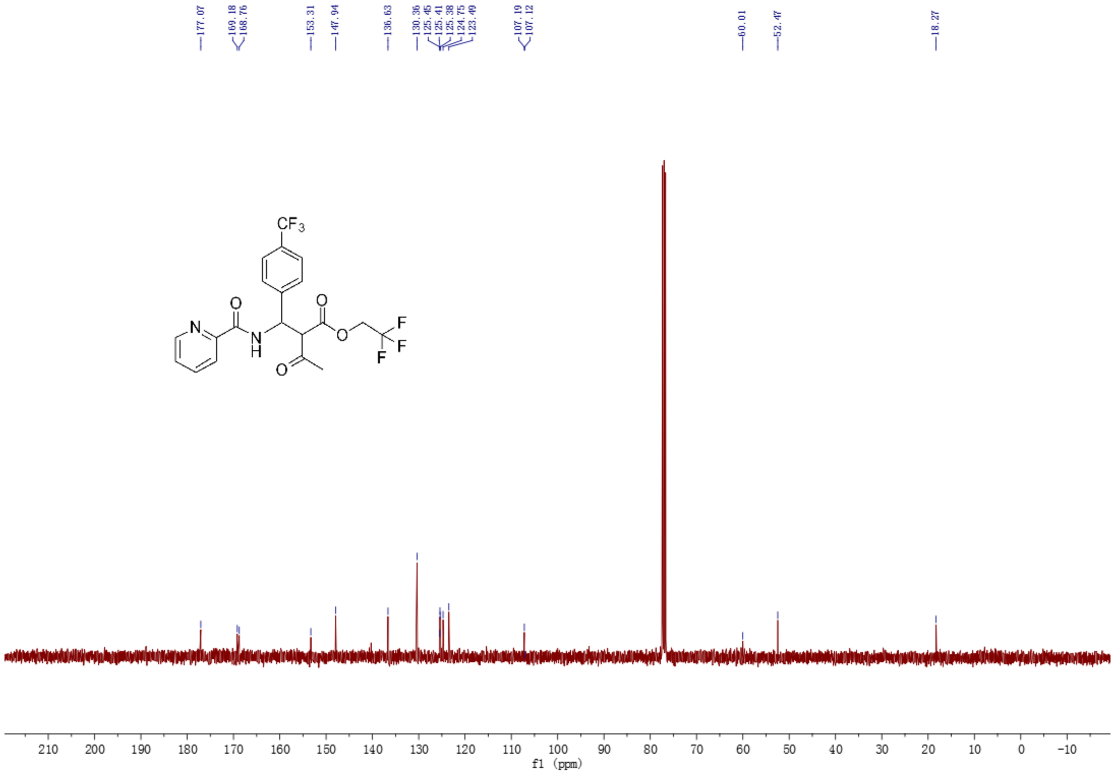

^{19}F NMR spectrum (376 MHz, $CDCl_3$) of **3-1c**

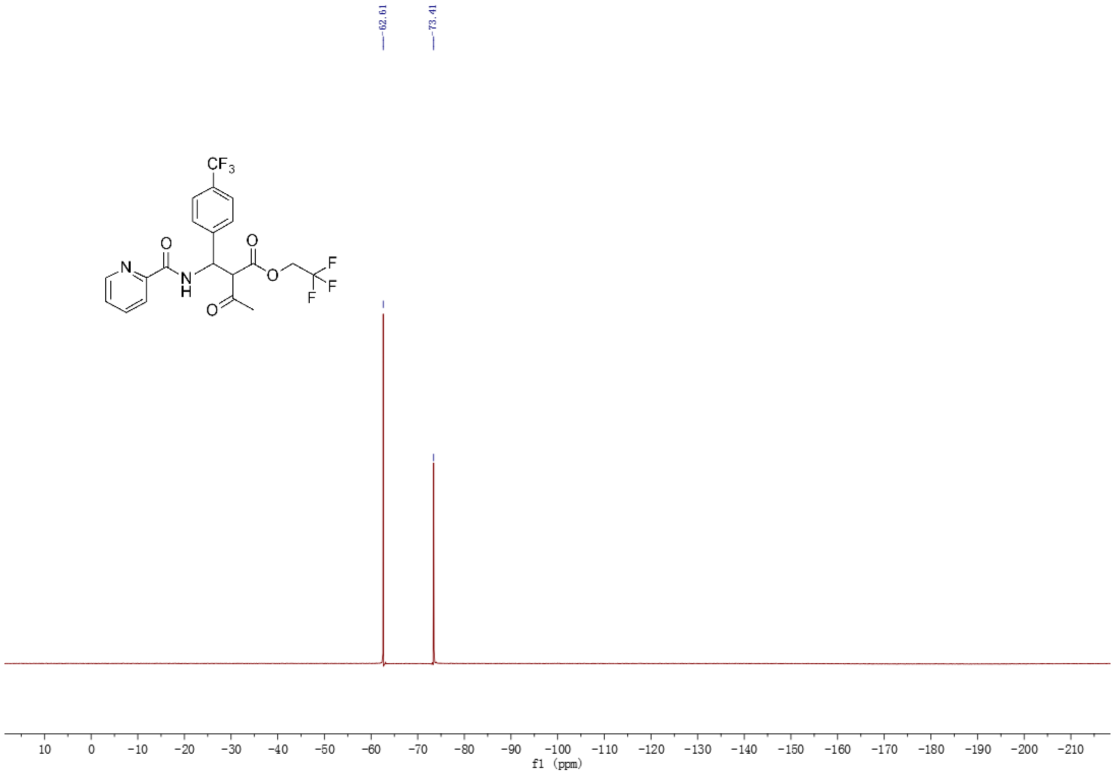

^{1}H NMR spectrum (400 MHz, $CDCl_3$) of **3-1d**

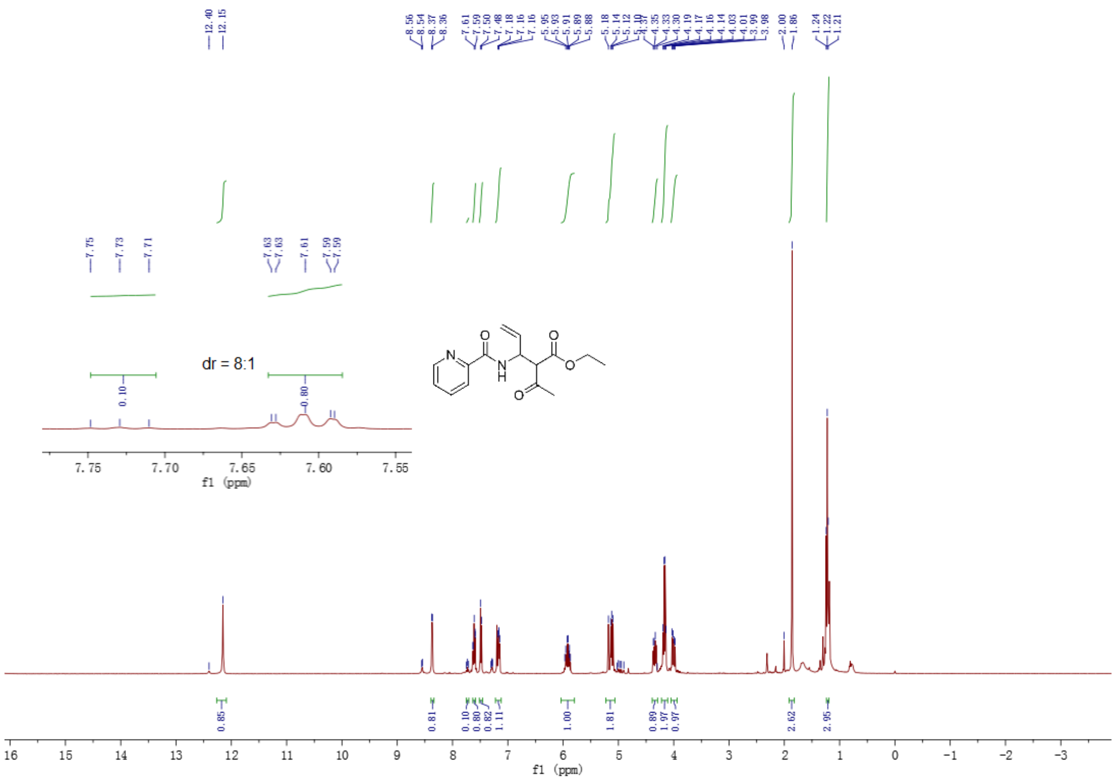

^{13}C NMR spectrum (100 MHz, $CDCl_3$) of **3-1d**

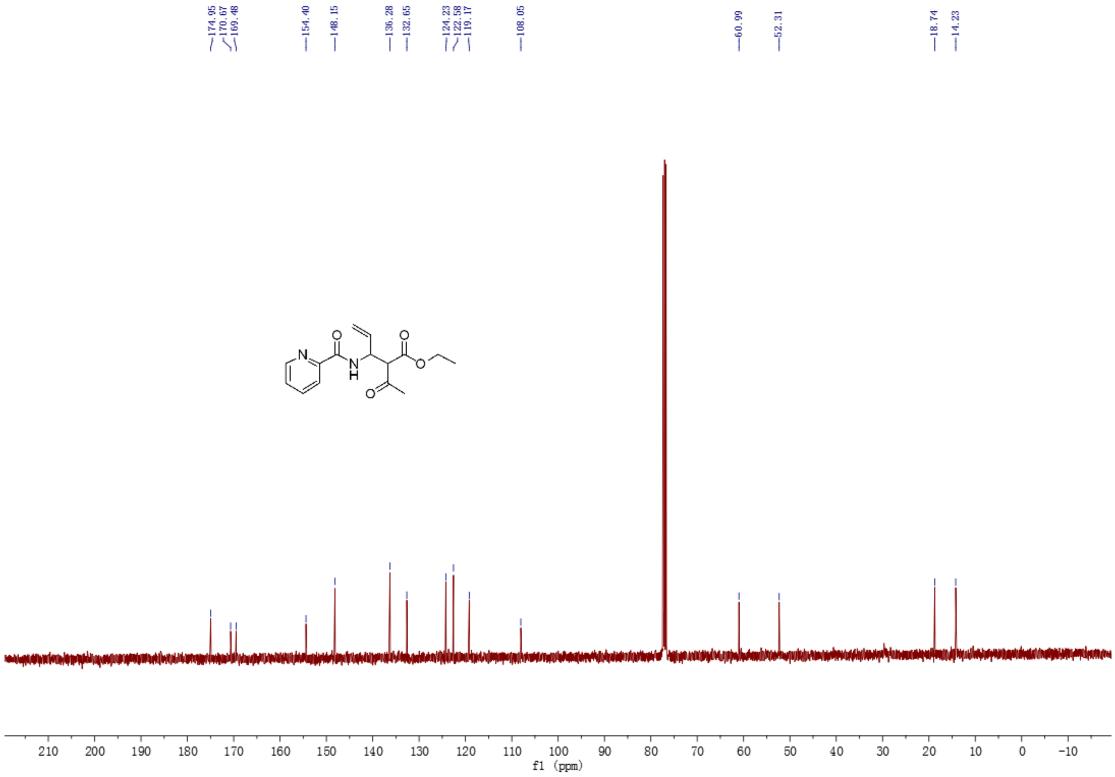

^{1}H NMR spectrum (400 MHz, $CDCl_3$) of **3-1e**

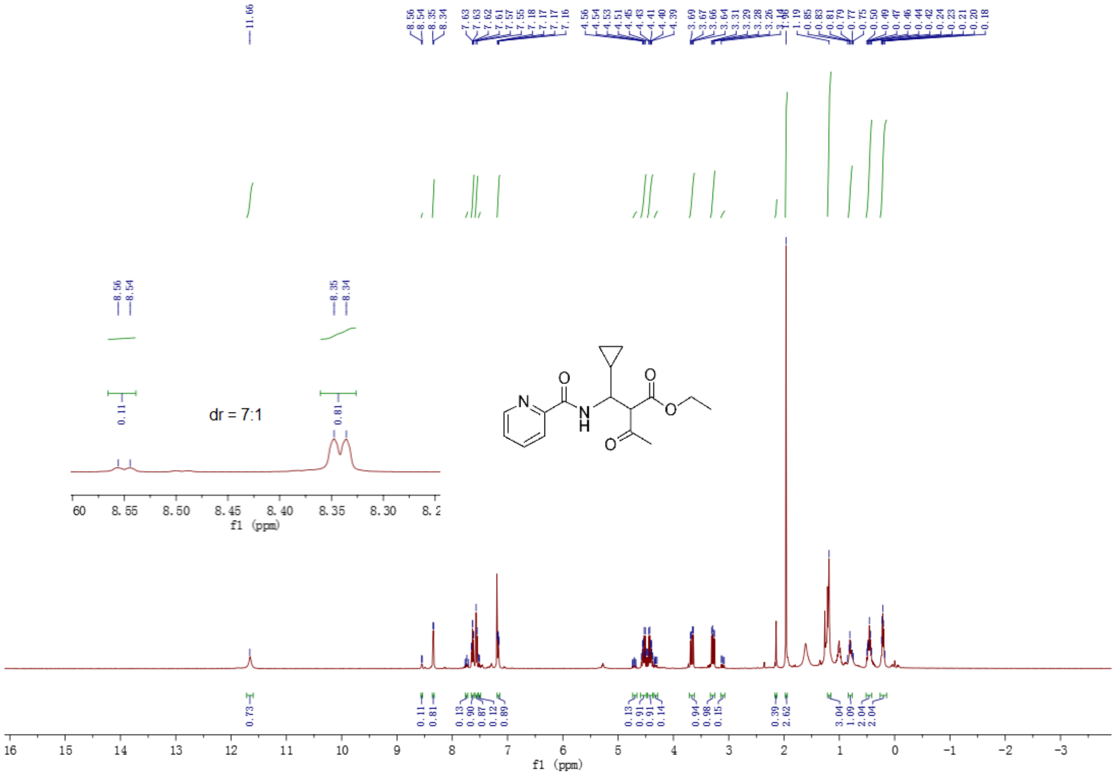

^{13}C NMR spectrum (100 MHz, $CDCl_3$) of **3-1e**

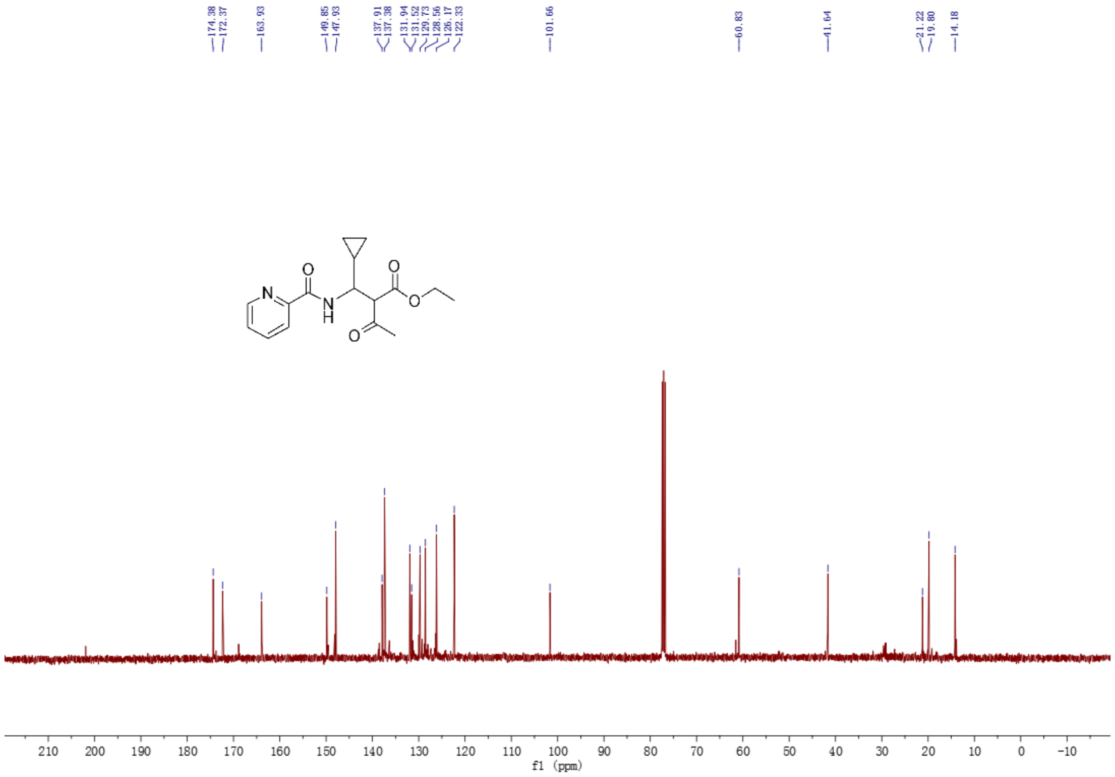

^{1}H NMR spectrum (400 MHz, $CDCl_3$) of **3-1f**

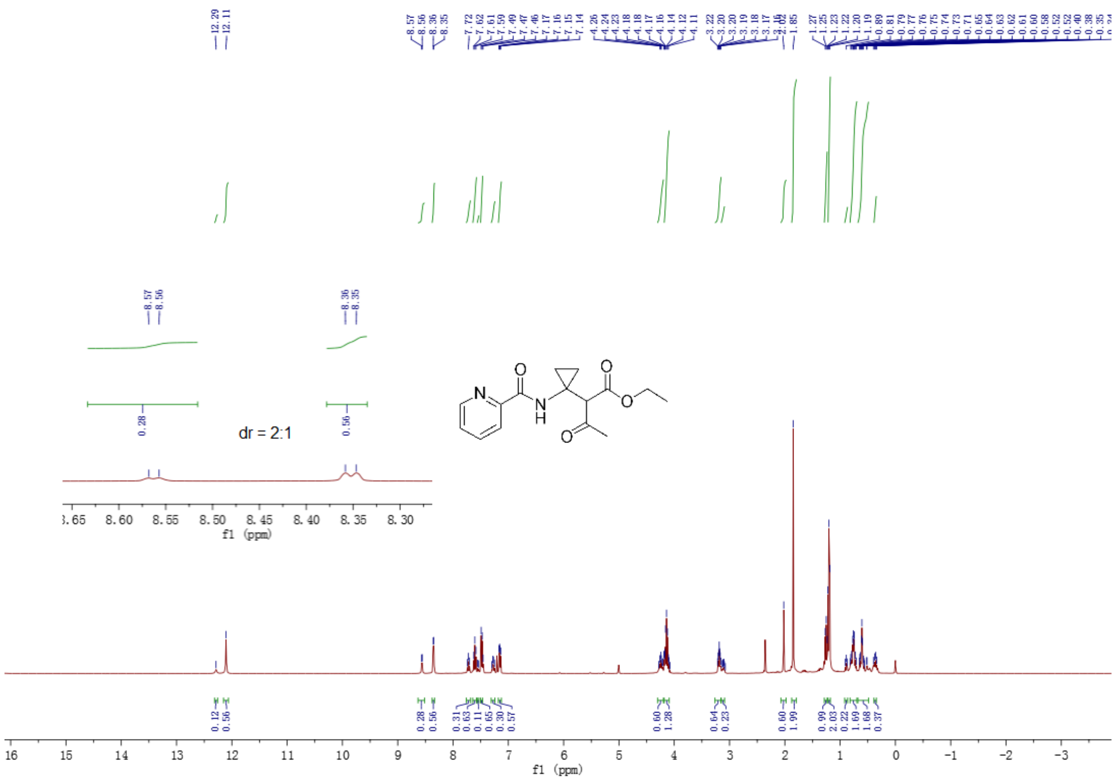

^{13}C NMR spectrum (100 MHz, $CDCl_3$) of **3-1f**

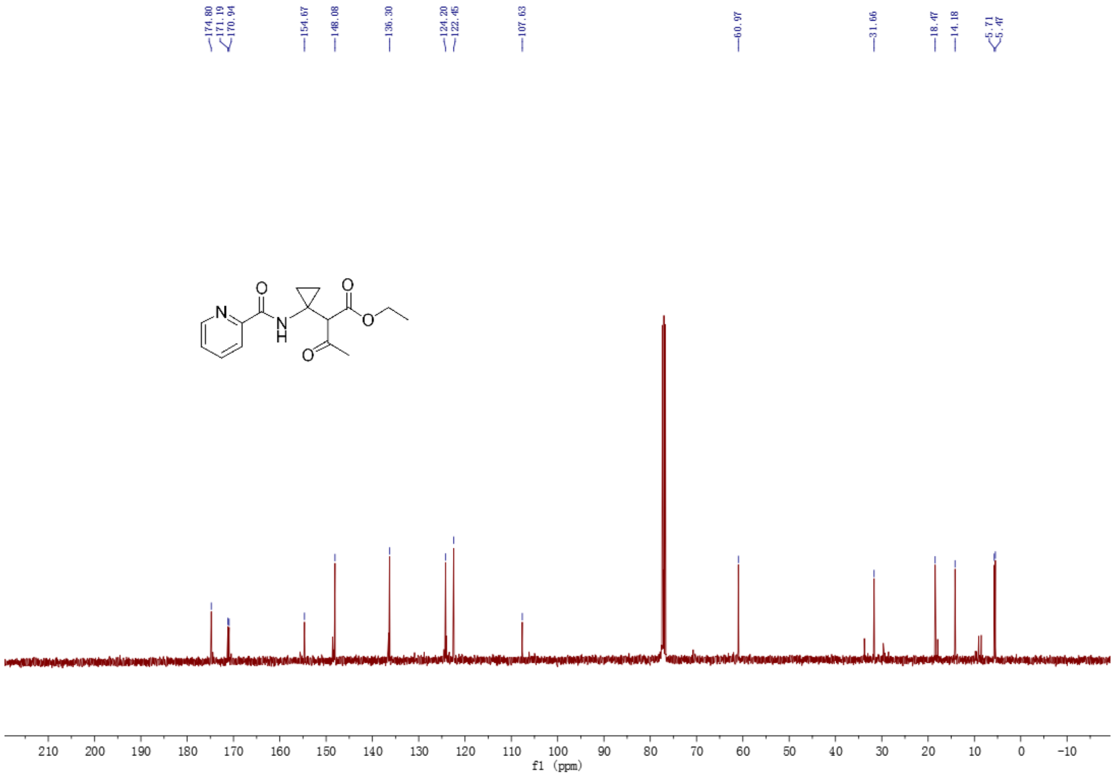

^{1}H NMR spectrum (400 MHz, DMSO/CF_3CO_2H, 20:1) of **4b**

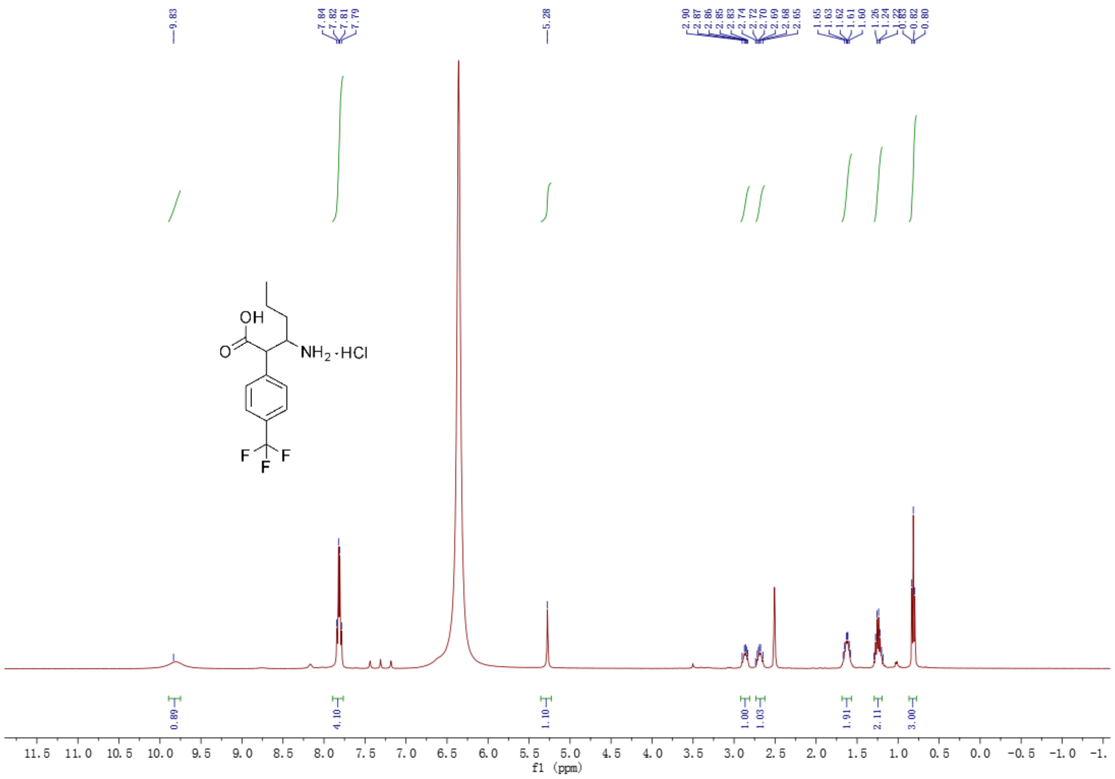

^{13}C NMR spectrum (100 MHz, DMSO/CF_3COOH, 20:1) of **4b**

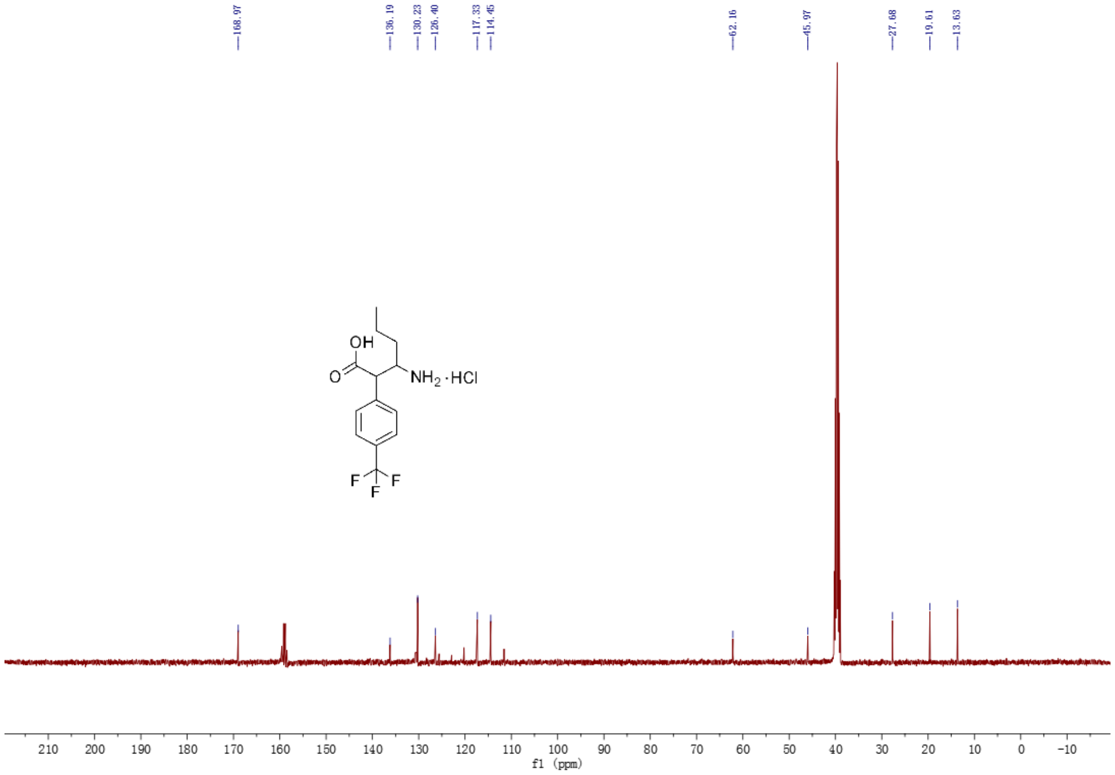

^{19}F NMR spectrum (376 MHz, $CDCl_3$) of **4b**

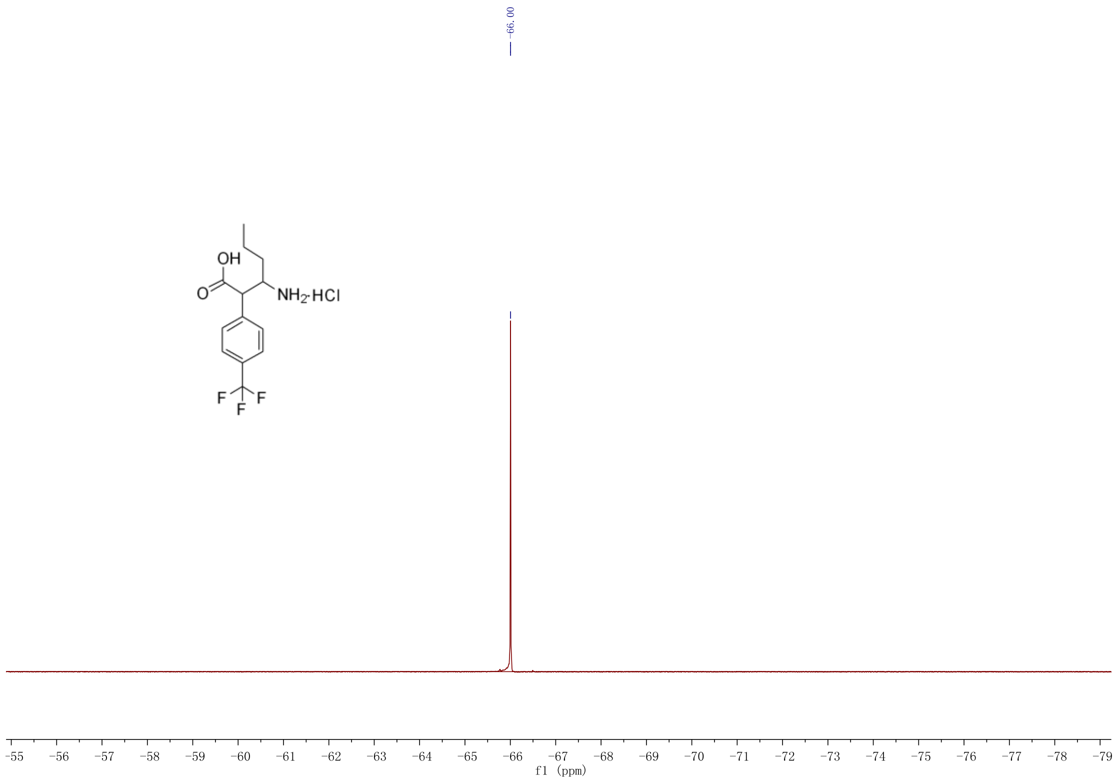

Supplement: Supplementary file 1 [file SC-009-C7SC03802J-s001.pdf]
